# Human adult and pediatric multimodal transcriptomic atlas of progression from pressure-loaded to failing right ventricle

**DOI:** 10.1038/s44161-026-00885-5

**Published:** 2026-10-09

**Authors:** Ivan A. Kuznetsov, Kristina Li, Yasmine Guedira, Bridget Simonson, Mark Chaffin, Yonathan T. Aberra, Kenneth C. Bedi, Trace Thome, Yijun Yang, Kirsten Branch, Wencao Zhao, Wenkai Zhu, Wei Zhou, Farid F. Kadyrov, Junedh M. Amrute, Ling Lai, Joanna Griffin, Li Li, Jian Li, Shelley D. Miyamoto, Patrick Ellinor, Kenneth B. Margulies, Kory J. Lavine, Zoltan Arany, Jonathan J. Edwards

**Affiliations:** 1 Cardiovascular Institute, University of Pennsylvania, Philadelphia, PA USA; 2 Department of Bioengineering, University of Pennsylvania, Philadelphia, PA USA; 3 Cardiovascular Disease Initiative, The Broad Institute, Cambridge, MA USA; 4 Cardiovascular Institute, Children’s Hospital of Philadelphia, Philadelphia, PA USA; 5 Center for Cardiovascular Research, Department of Medicine, Cardiovascular Division, Washington University School of Medicine, St. Louis, MO USA; 6 Department of Pediatrics, University of Colorado Anschutz Medical Campus, Children’s Hospital Colorado, Aurora, CO USA

**Keywords:** Cardiovascular biology, Cell biology

## Abstract

Right ventricular failure (RVF) independently predicts mortality in heart failure, yet its pathophysiology is poorly understood. Here we present a multi-modal atlas of human right ventricular pressure loading and failure, including bulk (*n* = 142), single-nucleus (*n* = 11) and Xenium spatial (*n* = 9) transcriptomics in adults, plus pediatric (*n* = 14) and mouse pulmonary artery banding cohorts. Subclustering resolved 34 cell subtypes across 12 lineages along a two-phase trajectory. Phase 1 (non-failing to pressure-loaded) accounts for most of the transcriptional change detectable by bulk and single-nucleus RNA sequencing and is marked by loss of resident macrophage identity, fibroblast activation, endothelial expansion and erosion of tissue-protective programs. Phase 2 (pressure-loaded to RVF) produces little change in bulk RNA sequencing but is resolved by spatial transcriptomics, engaging multi-lineage fibrotic, endothelial-activation and cardiomyocyte-reactivation programs, with a gain of extracellular matrix (collagen, laminin, thrombospondin) signaling over a stable cell contact adhesion baseline. Mitochondrial respirometry revealed respiratory dysfunction in adult and mouse RVF but not pediatric RVF. Multi-lineage remodeling of the cardiac microenvironment emerges as the central molecular program of RVF, meriting further study as possible disease-modifying targets.

## Abstract (web summary)

Kuznetsov et al. present a multi-modal transcriptomic atlas of the human right ventricle, revealing a two-phase progression from pressure loading to failure, with erosion of tissue-protective programs preceding focal fibrotic and vascular remodeling.

## Main

The normal right ventricle (RV) is morphologically and embryologically unique from the left ventricle (LV), precluding the categorical extrapolation of our understanding of left-side pathophysiology to the right heart^1^. Historically, cardiac research has focused on left ventricular failure (LVF), delivering multiple approved therapies, with less attention to RVF^2^. Although the most common cause of RVF is pressure overload secondary to LVF, RVF can also arise from diverse other causes^3–5^. Independent of etiology, RV dysfunction portends a poor prognosis: patients with LVF and concomitant RV dysfunction have approximately a two-fold increased mortality risk^6–8^.

Advances in LVF therapy have been driven by targeting critical pathways such as the renin−angiotensin−aldosterone system (RAAS)^9^. Attempts to generalize these therapies to the RV have been clinically unsuccessful. Neurohormonal blockade, a cornerstone of LVF therapy, has little efficacy in RVF^9^. There is, therefore, a need for exploratory research to identify RVF-specific therapeutic targets.

We conducted a multi-platform transcriptomic study of failing and non-failing human RVs, integrating bulk RNA sequencing (RNA-seq) (142 individuals), single-nucleus RNA sequencing (snRNA-seq) (11 individuals) and Xenium spatial transcriptomics (nine sections), with mitochondrial respirometry, flow cytometry and histological validation. We placed these findings in broader context by comparing to a mouse pulmonary artery banding (PAB) model, pediatric single-ventricle RVs, LVF and a published pulmonary arterial hypertension (PAH) RV transcriptomic dataset. Recent human cardiac snRNA-seq atlases remain LV centric^10–14^ or include limited RV in genetically defined cohorts^15^. Although some include non-failing (NF) RV tissue^11,14,16^, none has stratified pressure-overload RVF progression or integrated bulk, single-nucleus and spatial modalities on matched patient tissue. Here we present, to our knowledge, the first such integrated atlas and propose a two-phase model in which multi-lineage erosion of protective programs occurs in various cell types during the transition from NF to preserved RV in the context of LVF (NF → pRV) and is followed by a pRV → RVF transition that is comparatively muted in bulk RNA expression changes but engages a broader multi-lineage signal elicited in Xenium spatial pseudobulk analyses.

## Results

### Cohort and multi-platform approach

**Fig. 1:**
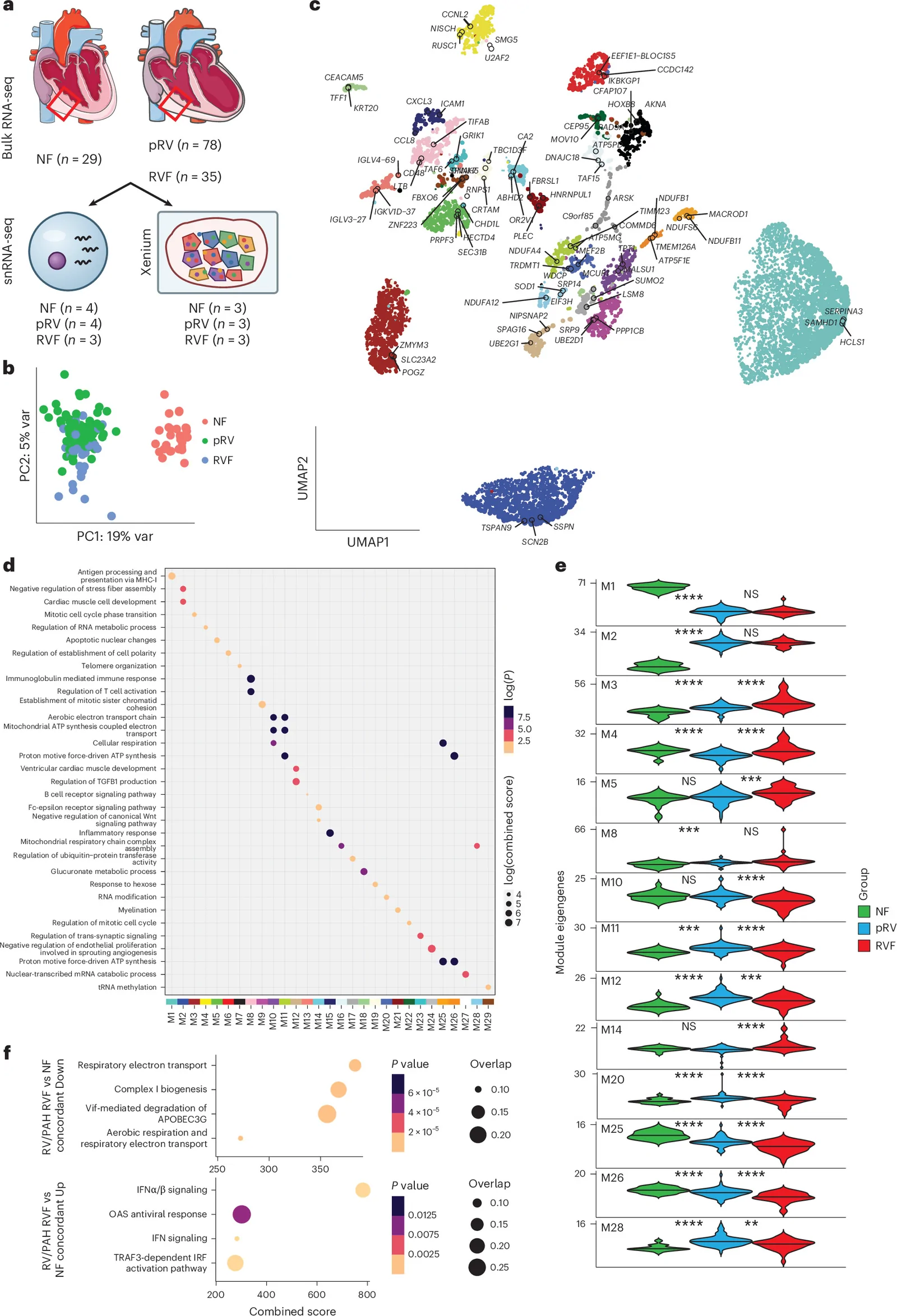
Study overview and bulk RNA-seq of human RVF. **a**, Cohort schematic across three platforms: bulk RNA-seq (NF *n* = 29, pRV *n* = 78, RVF *n* = 35), snRNA-seq (NF *n* = 4, pRV *n* = 4, RVF *n* = 3) and Xenium spatial transcriptomics (NF *n* = 3, pRV *n* = 3, RVF *n* = 3). **b**, PCA embeddings of normalized and transformed counts from RV samples show prominent differences between NF and pRV/RVF samples but relative similarity of pRV and RVF samples. **c**, UMAP for dimension reduction of WGCNA results. Points correspond to genes. Distance metric computed from WGCNA topological overlap matrix. WGCNA identifies 29 gene expression modules within bulk data. Top-5 hub genes corresponding to the given module are labeled. Gene Ontology (Biological Processes) enrichments for top-100 hub genes are overlaid for key modules. **d**, Gene Ontology enrichments for WGCNA modules. Point size corresponds to Enrichr combined scores, and color corresponds to multiple comparison-adjusted *P* value. Enrichment was tested with Fisherʼs exact test. Gene sets are the top-100 hub genes per module ranked by kME. **e**, WGCNA module score violin plots across NF/pRV/RVF for the 14 of 29 bulk modules with a significant module eigengene shift in at least one adjacent transition. Left-sided comparison corresponds to NF versus pRV, and right-sided comparison corresponds to pRV versus RVF. The NF versus RVF comparison is not shown for simplicity. Exact Bonferroni-adjusted *P* values for the significant contrasts are M1 NF → pRV 6.7 × 10^−14^; M2 NF → pRV 6.7 × 10^−14^; M3 NF → pRV 4.9 × 10^−8^, pRV → RVF 5.3 × 10^−5^; M4 NF → pRV 1.3 × 10^−5^, pRV → RVF 1.7 × 10^−4^; M5 pRV → RVF 0.027; M8 NF → pRV 0.0062; M10 pRV → RVF 5.5 × 10^−5^; M11 NF → pRV 2.7 × 10^−6^, pRV → RVF 0.010; M12 NF → pRV 2.0 × 10^−11^, pRV → RVF 0.0091; M14 pRV → RVF 2.4 × 10^−4^; M20 NF → pRV 8.1 × 10^−7^, pRV → RVF 1.3 × 10^−4^; M25 NF → pRV 1.6 × 10^−8^, pRV → RVF 9.2 × 10^−5^; M26 NF → pRV 0.024, pRV → RVF 1.9 × 10^−4^; M28 NF → pRV 1.2 × 10^−9^. All other displayed contrasts are non-significant (adjusted *P* ≥ 0.05). Module eigengene values are shown per bulk RNA-seq sample. Module eigengenes were compared between groups with a two-sided Wilcoxon rank-sum test with Bonferroni adjustment across the modules. Significance brackets on the panel denote the Bonferroni-adjusted *P* value: NS, not significant (*P* ≥ 0.05); *P < 0.05; **P < 0.01; ***P < 0.001; ****P < 0.0001. The 15 modules with no significant shift in either adjacent transition are not displayed. The full per-module table is provided in Source Data Fig. 1. **f**, Cross-cohort concordance dot plots between RV failure (RVF versus NF) and PAH for concordantly downregulated and upregulated pathways. Concordant gene sets were defined as genes changing in the same direction in both cohorts at DESeq2 Benjamini−Hochberg *P*_adj_ < 0.1 and |log_2_FC| > 0.1. Pathway enrichment used one-sided Fisherʼs exact test with Benjamini−Hochberg adjustment. Down, downregulated; OAS, oligoadenylate synthetase; *P*_adj_, adjusted *P* value; PC, principal component; Up, upregulated; var, variance explained. Source data

From the Penn Human Heart Tissue Bank, we collected transmural RV samples from adult NF organ donors and LVF transplant recipients, the latter substratified^17,18^ into pressure-loaded but preserved systolic function (pRV) and failing (RVF) groups (Fig. 1a and Supplementary Tables 1–3). We applied bulk RNA-seq (*n* = 142), snRNA-seq (*n* = 11; 61,398 nuclei) and Xenium spatial transcriptomics (*n* = 9 sections; 625,305 cells; 477-gene panel); a mouse PAB model at 2-week and 2-month timepoints; a pediatric snRNA-seq cohort (*n* = 14; 10 hypoplastic left heart syndrome (HLHS)-palliated RVs stratified by normal function or RVF and four controls); and a cross-etiology comparison with a published PAH RVF bulk dataset^19^.

### Bulk RNA-seq reveals the transcriptomic landscape of RV failure progression

**Extended Data Fig. 1:**
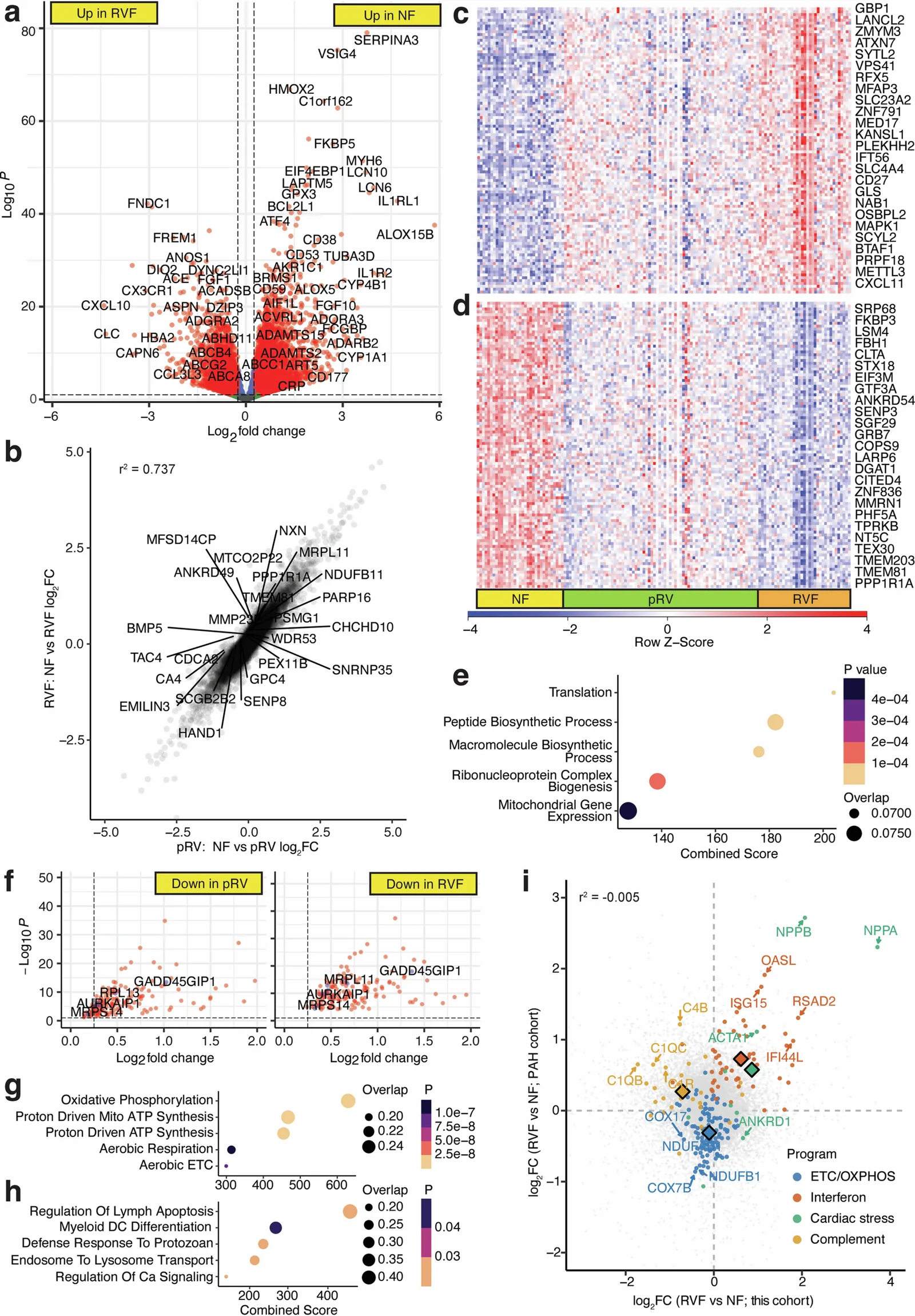
Bulk RNA-seq supplemental analyses. (**a**) Volcano plot showing DEGs for the RVF vs NF comparison from bulk RNA-sequencing data. (**b**) Scatter plot of log_2_fold changes for the NF vs RVF vs pRV vs NF comparison by gene. Fold changes are well correlated. Outliers are labeled. (**c**) Heatmap of per sample gene expression for genes which show increasing expression from NF to pRV to RVF. Color corresponds to z-score of expression. For visual simplicity only a subset of genes are labeled. (**d**) Heatmap of per sample gene expression for genes which show decreasing expression from NF to pRV to RVF. Color corresponds to z-score of expression. For visual simplicity only a subset of genes are labeled. (**e**) Top 5 gene ontology biological processes enrichments for stepwise decreasing genes. These genes primarily belong to M1, M25, and M26 and enrich for translational machinery, primarily mitochondrial translational machinery. (**f**) Volcano plots showing progressively downregulated DEGs for the NF vs pRV comparison (left) and NF vs RVF comparison (right). Highlighted are *GADD45GIP1*, *MRPL11*, *MRPS14*, which are components of the mitochondrial ribosome. (**g**) Top 5 gene ontology biological processes enrichments for pRV upregulated genes in the pRV vs RVF comparison. (**h**) Top 5 gene ontology biological processes enrichments for RVF upregulated genes in the pRV vs RVF comparison. (**i**) Cross-cohort comparison of RV-failure transcriptomes. Each gene is plotted by its log_2_fold change (RVF vs NF) in our RV cohort versus a PAH-associated RV-failure cohort (GSE240941), colored by membership in four programs (ETC/OXPHOS, interferon, cardiac stress, complement). Diamonds mark program centroids. Interferon and cardiac-stress programs are concordantly up-regulated and ETC/OXPHOS concordantly down-regulated across both cohorts, whereas complement is divergent (down in our RVF, up in PAH), indicating etiology-specific responses. Program gene membership is provided in Supplementary Data 5. Source data

Principal component analysis (PCA) of bulk RNA-seq separated NF from pRV/RVF but poorly separated pRV from RVF (Fig. 1b). Differential expression (DE) yielded many NF versus pRV/RVF differentially expressed genes (DEGs) with strong NF → pRV and NF → RVF fold change (FC) correlation (Extended Data Fig. 1a,b and Supplementary Data 1). Prominent DEGs common to pRV and RVF included cardiac stress (*NPPA*, *NPPB*), chemokines (*CXCL8*–*11*) and extracellular matrix (ECM)/angiogenic genes. Pathway enrichment highlighted immune, complement and inflammatory terms (Supplementary Data 2), suggesting that immune populations dominate the bulk signal. Many DEGs exhibited stepwise changes from NF → pRV → RVF, with 115 stepwise-increasing genes enriched for T cell chemotaxis, and 161 stepwise-decreasing genes enriched for mitochondrial ribosomal components (Extended Data Fig. 1c–f and Supplementary Data 3). Fewer significant DEGs were identified from pRV → RVF analysis, indicating that most transcriptional remodeling precedes functional decompensation. pRV-upregulated genes enriched for oxidative phosphorylation (OXPHOS); RVF-upregulated genes were immune dominated (Extended Data Fig. 1g,h and Supplementary Data 2).

We also evaluated DE by weighted gene correlation network analysis (WGCNA)^20^ to capture broader co-expression signals, resolving 29 gene modules, almost all of which were differentially expressed across states (Fig. 1c–e and Supplementary Data 4). Two OXPHOS modules (M25 and M26) declined monotonically across NF → pRV → RVF at the module eigengene level, whereas the large resident macrophage module M1 (*CD163*, *MRC1*, *VSIG4*) was highest in NF and fell sharply from NF to pRV before plateauing. In the opposing direction, an adaptive immune module (M8; T/B cell and major histocompatibility complex class II (MHC-II) genes) rose significantly from NF to pRV (Fig. 1d,e and Supplementary Data 4).

To test cross-etiology generalizability, we compared our bulk data to a published PAH-associated RVF transcriptome^19^. RVF versus NF yielded 235 concordantly downregulated genes (respiratory electron transport (ETC), complex I biogenesis) and 214 concordantly upregulated genes (interferon (IFN) α/β, antiviral signaling), establishing mitochondrial decline and IFN activation as etiology shared (Fig. 1f). By contrast, the complement cascade was divergent between cohorts, downregulated in our RVF but upregulated in PAH RVF, identifying complement activation as an etiology-specific response (Extended Data Fig. 1i and Supplementary Data 5).

### Single-nucleus transcriptomics resolves 34 cell subtypes across 12 lineages

**Fig. 2:**
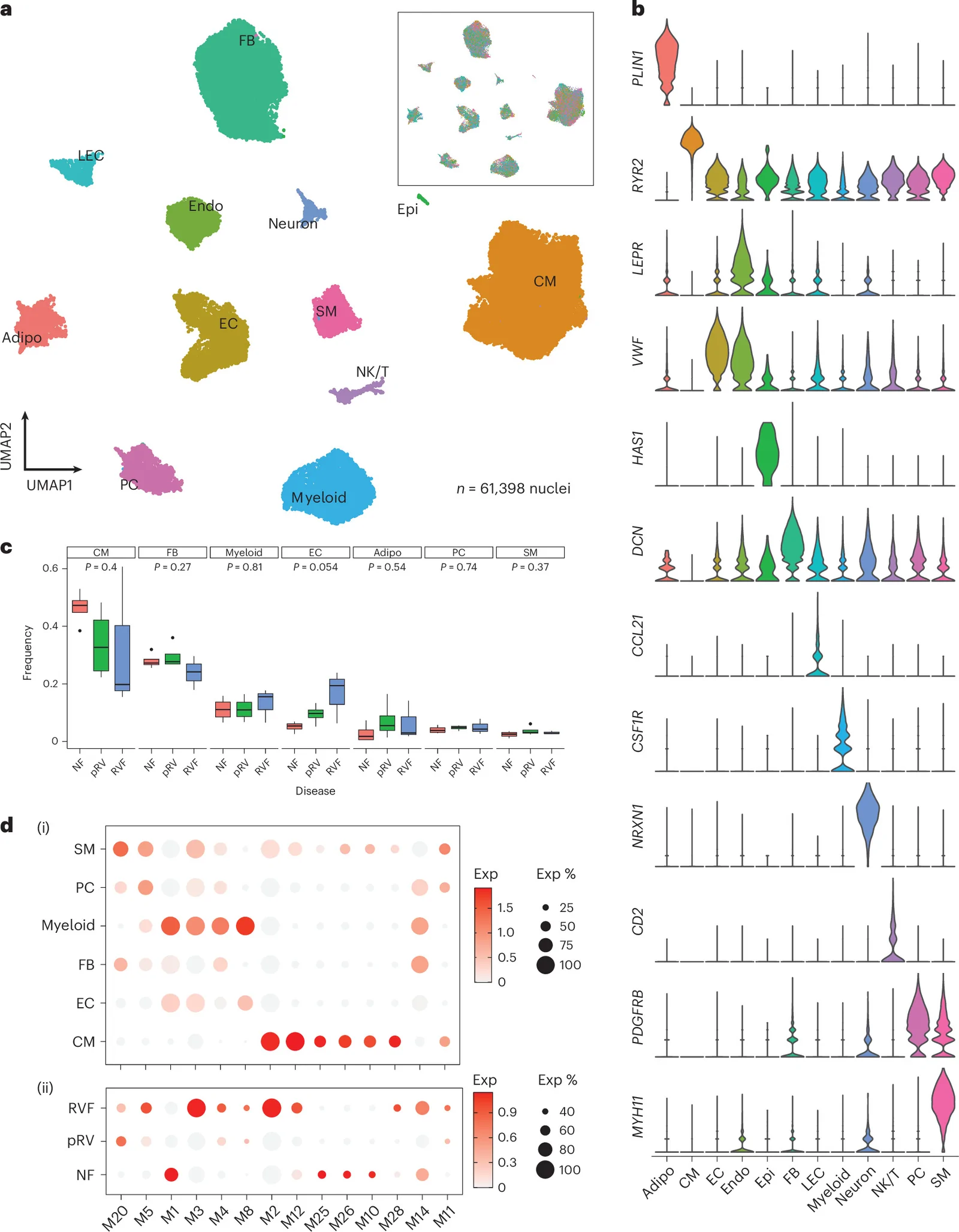
Single-nucleus transcriptomic atlas of the RV. **a**, UMAP of 61,398 nuclei colored by 12 major lineage clusters (CM, FB, EC, Endo, SM, Myeloid, NK/T, PC, Adipo, Epi, LEC and Neuron); inset shows the same UMAP colored by patient (*n* = 11) to illustrate donor-consistent integration. **b**, Stacked violin plots of canonical lineage markers. **c**, Per-lineage frequency box plots across NF, pRV and RVF (*n* = 4/4/3) with ANOVA *P* values, capturing progressive EC expansion and CM contraction. Each point is one biologically independent donor. Box plots show the median (center line), the 25th and 75th percentiles (box bounds) and whiskers extending to the most extreme observation within 1.5× the IQR of the box (minima and maxima). Points beyond that range are plotted individually as outliers. Frequencies are the per-patient proportion of nuclei assigned to each lineage. *P* values are one-way ANOVA (two-sided *F*-test) per lineage, unadjusted across lineages. *F*, d.f. and exact *P* values per lineage are given in Source Data Fig. 2. **d**, Two-row dot plot of bulk WGCNA module scores projected onto the snRNA-seq atlas: (i) module score by lineage (CM, EC, FB, Myeloid, PC, SM); (ii) module score by disease group. Adipo, adipocyte; CM, cardiomyocyte; d.f., degrees of freedom; Endo, endocardial; Epi, epicardial; Exp, normalized *z*-score of module score; Exp %, percent of nuclei expressing the given module with *z*-score > 0; FB, fibroblast; LEC, lymphatic endothelial cell; NK/T, natural killer/T; PC, pericyte; SM, smooth muscle. Source data

**Extended Data Fig. 2:**
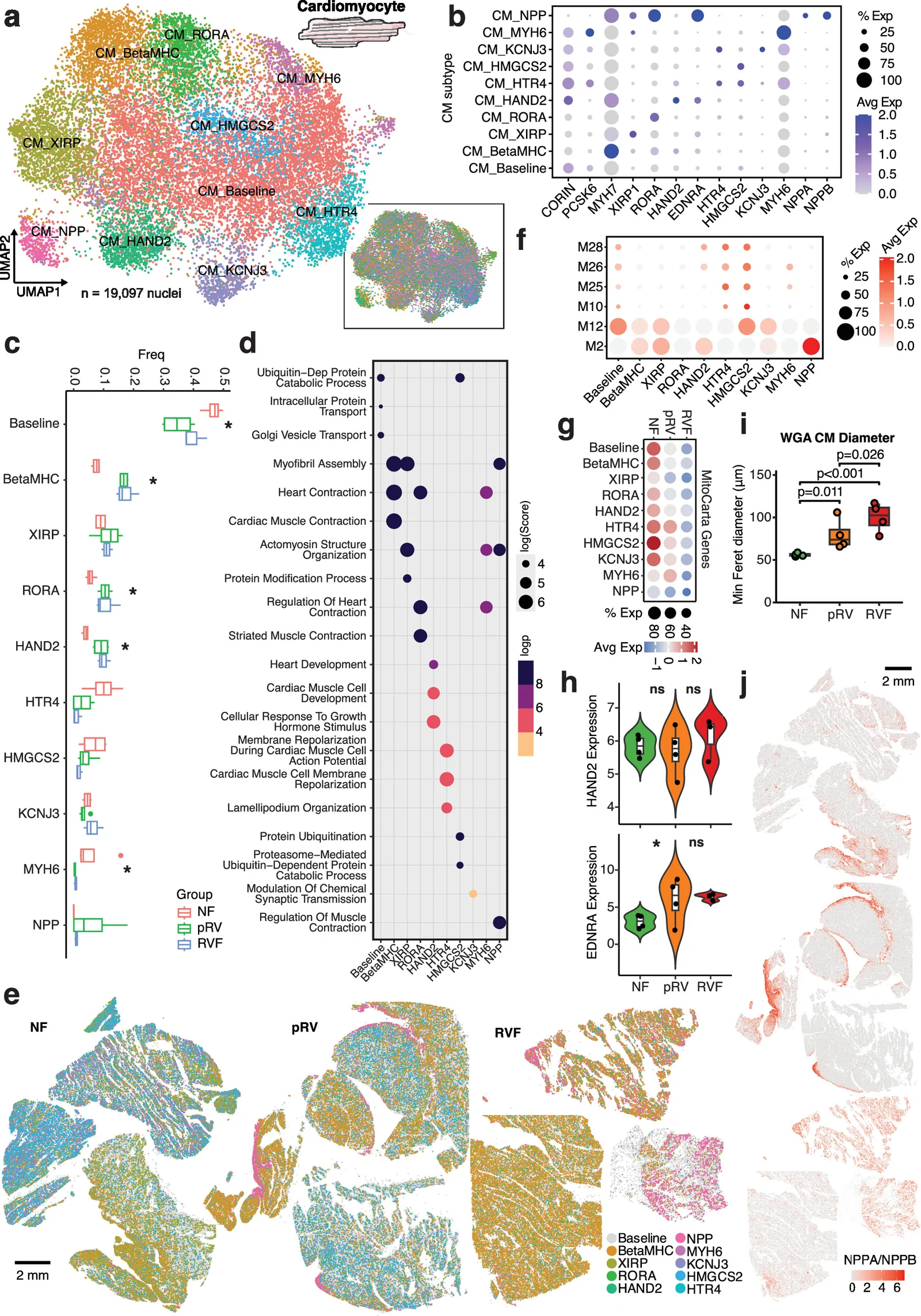
Cardiomyocyte subclustering and spatial validation. (**a**) UMAP showing subclustering of cardiomyocytes into 10 subpopulations. Inset shows the same UMAP coloured by patient (n = 11) to illustrate donor-consistent integration of cardiomyocyte subclusters. (**b**) Dot plot of marker genes for each cardiomyocyte subtype. (**c**) Cluster frequency, split by disease state, as proportion of total cells. * indicates p < 0.05 for one-way Kruskal–Wallis. (**d**) Top 3 gene ontology biological processes enrichments for marker genes for each cardiomyocyte cluster. Only enrichments with an adjusted p-value < 0.05 are shown. Point size corresponds to Enrichr combined scores and color corresponds to multiple comparison-adjusted p value. (**e**) Spatial distribution of cardiomyocyte subclusters, as visualized on the Xenium spatial data. (**f**) Dot plot showing Seurat-computed module score for given WGCNA modules by cardiomyocyte cluster. Exp – normalized z-score of module score; Exp % – Percent of nuclei expressing given module. (**g**) Dot plot of MitoCarta3.0 mitochondrial genes across CM subtypes × disease state, highlighting coordinated suppression of nuclear-encoded mitochondrial transcripts in pRV/RVF cardiomyocytes. Mean expression (color) and percent expressing (size). (**h**) snRNA-seq pseudobulk violin plot of *HAND2* (RV-specifying transcription factor) and *EDNRA* (endothelin-1 receptor) expression across disease states. Pairwise Wilcoxon shown. (**i**) Cardiomyocyte minimum Feret diameter (proxy for CM cross-sectional area) measured by wheat-germ agglutinin (WGA) staining of human RV sections (NF n = 4, pRV n = 4, RVF n = 4; 17,864 CMs total). Boxplot of per-patient median minimum Feret; each dot is one patient. Pairwise contrasts from a cell-level linear mixed model (random intercept by patient), BH-adjusted. (**j**) Per-patient rasterized spatial tiles of minimum-restricted *NPPA*/*NPPB* combined expression score on Xenium (mean of *NPPA* + *NPPB* log-normalised expression; continuous gray→red color scale), illustrating endocardial enrichment of *NPPA*/*NPPB*-expressing cardiomyocytes. Source data

**Extended Data Fig. 3:**
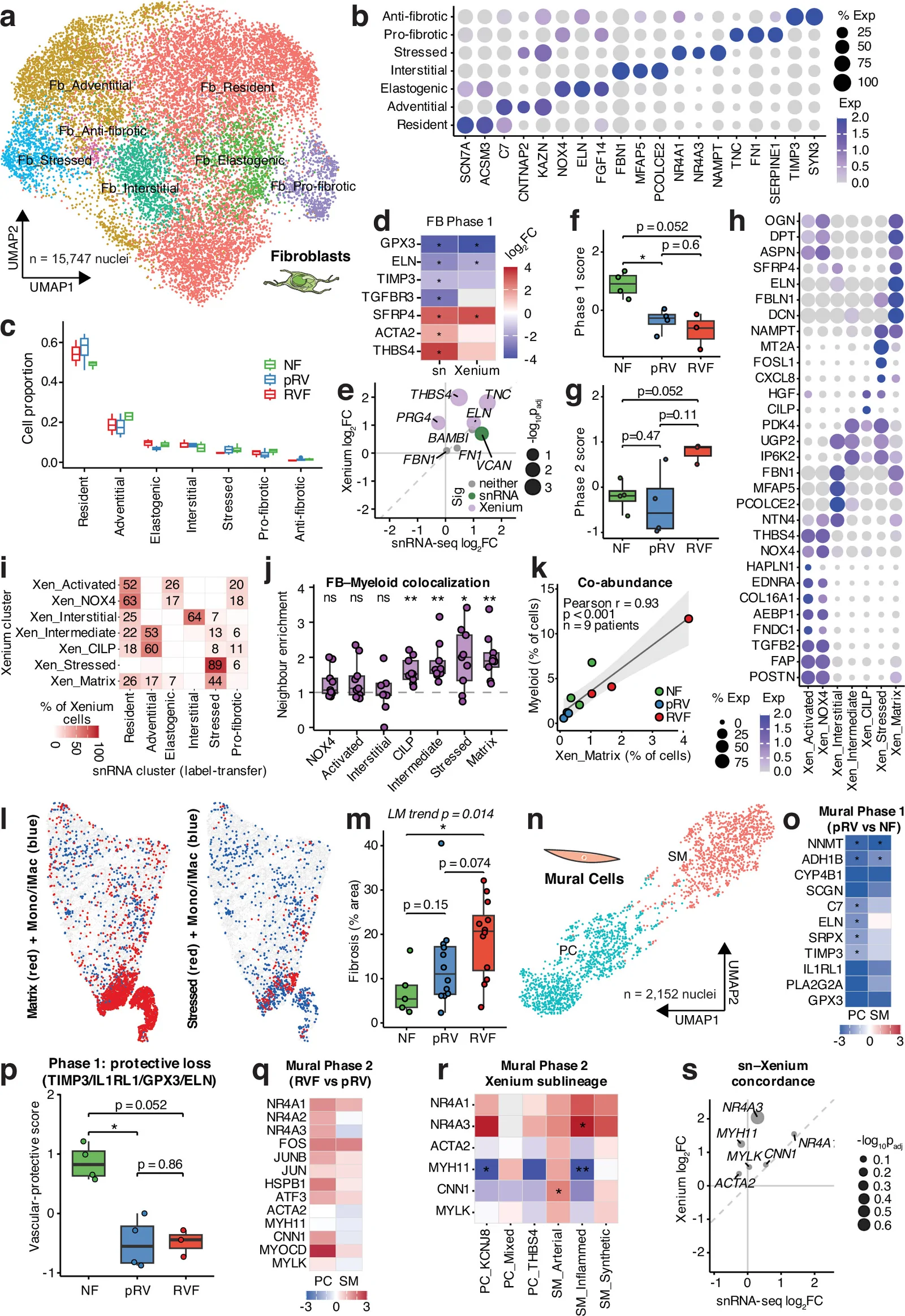
Fibroblast and mural cell subclustering and spatial validation. (**a**) UMAP showing subclustering of fibroblasts into 7 subpopulations. (**b**) Dot plot of FB subtype marker genes. (**c**) Per-patient FB subtype proportions across disease state (NF/pRV/RVF) shown as boxplots with per-patient dots. (**d**) FB Phase 1 (NF→pRV) two-platform concordance heatmap (snRNA-seq vs Xenium FB-lineage pseudobulk). Asterisks: p_adj_<0.05, |log_2_FC | >0.5, baseMean>50; grey tiles = off the 477-gene Xenium panel. (**e**) FB Phase 2 (pRV→RVF) sn-vs-Xenium concordance scatter at the FB lineage. Point size scales with –log_10_p_adj_. Color marks significance using a hybrid criterion: snRNA at FB lineage (sn p_adj_<0.05) or Xenium at FB sublineage (p_adj_<0.05 and |log_2_FC | >0.5). (**f**) “Erosion of FB identity” Phase 1 pseudobulk module score per patient using the Koenig et al. 2022 donor-FB signature (*GPX3*, *PID1*, *TGFBR3*, *ACSM3*, *APOD*; FB lineage; log-CPM, gene-wise z-scored). (**g**) Mirror-image “matrifibrocyte commitment” Phase 2 pseudobulk module score using the Fu et al. 2018 tendon/cartilage-like mature-scar signature (*COMP*, *CILP*, *CHAD*). (**h**) Xenium FB subtype dot plot using literature-supported cluster-defining markers. SpaGE-imputed expression is used where genes are off the 477-gene panel. (**i**) Xenium ↔ snRNA FB cluster similarity heatmap (Seurat label transfer). Each row = a Xenium FB subtype; each column = the % of cells in that row that mapped to the indicated snRNA Fb cluster. (**j**) FB–myeloid spatial colocalization in the Xenium data (K = 15 nearest-neighbor analysis; n = 9 patients). Per-FB-subtype median enrichment (observed myeloid neighbour fraction / patient-matched expected); reference line at 1.0; per-subtype Wilcoxon vs 1.0. (**k**) Per-patient co-abundance scatter (Xenium): Xen FB Matrix density (% cells) on x, total myeloid density (% cells) on y, dots colored by group. (**l**) Spatial vignettes in patient 1632 (RVF): all baseline cells grey, iMac + Monocyte highlighted in blue, and either Xen FB Stressed (top) or Xen FB Matrix (bottom) highlighted in red. (**m**) Adult RV trichrome / Sirius red fibrosis (% area) by disease state (n = 5 NF, 12 pRV, 12 RVF) boxplot with pairwise Wilcoxon brackets. (**n**) UMAP of mural cell subclustering (n = 2,152 nuclei: pericytes Pc=1,141, smooth muscle Sm=1,011). (**o**) Mural Phase 1 (pRV vs NF) snRNA sublineage heatmap. (**p**) Mural Phase 1 vascular-protective pseudobulk module score (PC + SM pooled). Pairwise Wilcoxon shown, (**q**) Mural Phase 2 (RVF vs pRV) snRNA sublineage heatmap. (**r**) Mural Phase 2 Xenium sublineage heatmap restricted to on-panel genes only and the 6 specific Xenium PC/VSMC subsets. (**s**) Mural Phase 2 sn-vs-Xenium concordance scatter at the mural lineage level (PC + SM pooled per platform). Same display conventions as panel (**e**). Source data

Single-nucleus RNA-seq of 11 RV samples (Supplementary Tables 4 and 5) yielded 61,398 nuclei (2,912 genes per nucleus) and 12 major clusters (Fig. 2a,b and Supplementary Data 6). Composition shifted progressively with disease: loss of cardiomyocyte nuclei and expansion of endothelial cell (EC) nuclei from NF → pRV → RVF (Fig. 2c). This compositional shift parallels reports from LVF snRNA-seq atlases, although, notably, the non-myocyte expansion here is endothelial predominant without the fibroblast expansion typical of LVF^12^. Bulk WGCNA modules were assigned to dominant lineages (Fig. 2d). Single-nucleus RNA-seq subclustering resolved 34 subtypes across 12 lineages: 10 cardiomyocyte, seven fibroblast, five EC, six myeloid and six lineages retained at lineage level (pericyte, smooth muscle, adipocyte, epicardial, neuronal/glial and natural killer/T) due to low counts (markers in Figs. 6b and 7b, Extended Data Figs. 2b and 3b and Supplementary Data 7). Pseudobulk DESeq2 within each subtype across three pairwise comparisons (Supplementary Data 8 and 9) identified fibroblasts as most transcriptionally responsive (1,083 DEGs pRV versus NF; 806 DEGs RVF versus NF), followed by cardiomyocytes (248; 464). This difference partially reflects lower inter-patient dispersion among fibroblasts.

### Spatial transcriptomics extends and validates single-nucleus findings

**Fig. 3:**
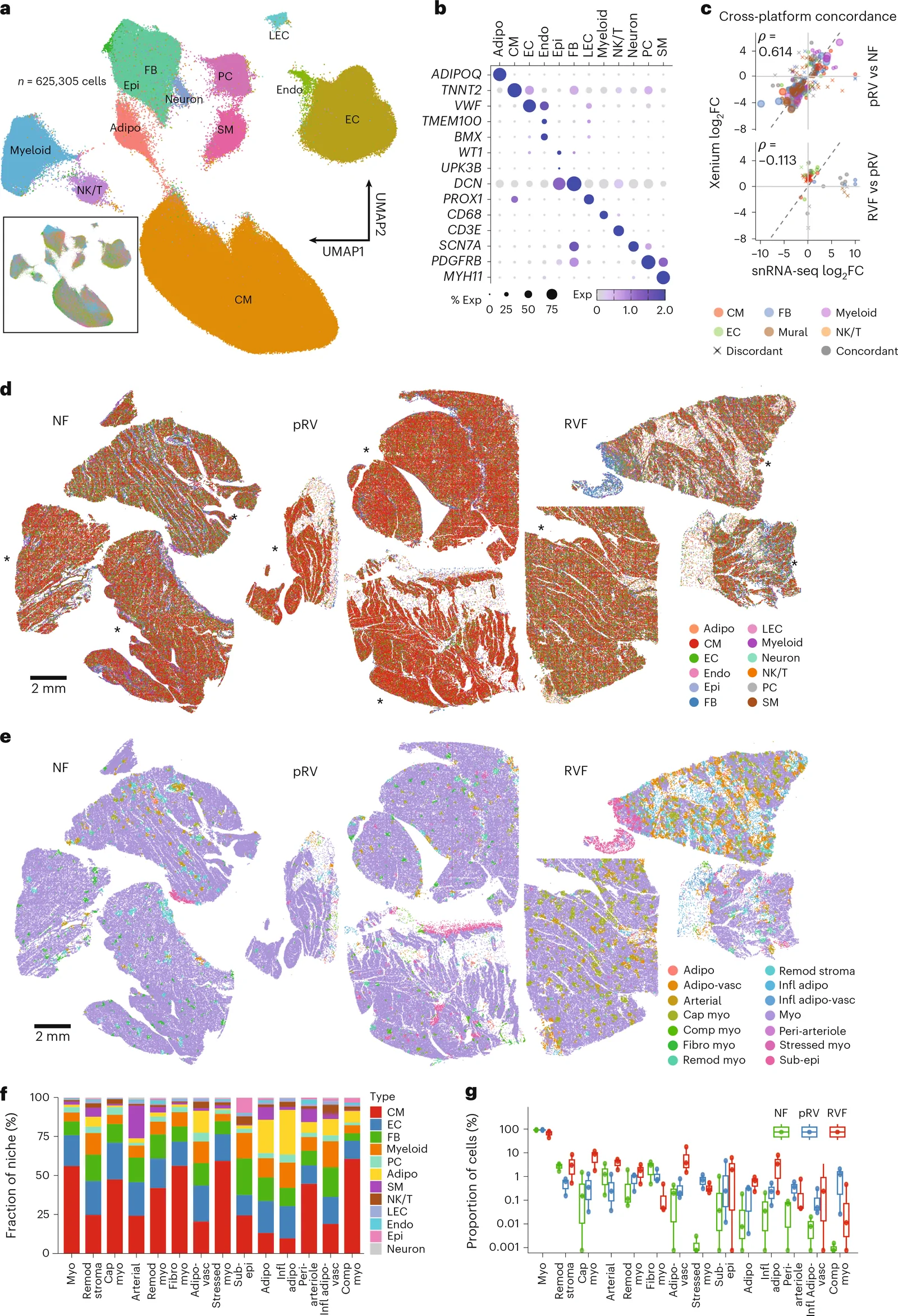
Xenium spatial transcriptomic atlas of the RV. **a**, Xenium UMAP of 625,305 cells colored by 12 lineage clusters. Inset shows the same UMAP colored by patient (*n* = 9) to illustrate donor-consistent integration. **b**, Dot plot of canonical lineage markers across the 12 Xenium lineages. Dot color encodes mean expression, and dot size encodes the percentage of expressing cells. **c**, Cross-platform concordance scatter of pseudobulk log_2_FC: snRNA-seq (*x* axis) versus Xenium (*y* axis) for matched lineages and contrasts, with points colored by lineage and symbols separating concordant from discordant calls. Spearmanʼs *ρ* is reported per comparison. **d**, Spatial maps of all NF, pRV and RVF sections after Proseg resegmentation and RCTD annotation, colored by lineage. Endocardial border is indicated with asterisk. Scale bar is shown. **e**, Spatial maps of the same sections colored by spatial niche assignment, illustrating the spatial organization of the 14 microenvironments resolved by BuildNicheAssay (*k* = 100, CLR transformed). Niche labels are abbreviated as follows: adipo (adipocyte-rich); adipo-vasc (adipo-vascular); arterial; cap myo (capillary myocardium); comp myo (compact myocardium); fibro myo (fibrotic myocardium); infl adipo (inflammatory adipocyte); infl adipo-vasc (inflammatory adipo-vascular); myo (myocardium); peri-arteriole; stressed myo (stressed myocardium); remod myo (remodeling myocardium); remod stroma (remodeling stroma); sub-epi (sub-epicardial). **f**, Stacked bar chart of niche composition by lineage (fraction-of-niche), summarizing the cellular identity of each microenvironment. **g**, Niche frequency box plots across NF, pRV and RVF; *y* axis is shown on a log scale. Niche frequencies are compositional: each segmented cell is assigned to exactly one of the 14 niches, so niche fractions sum to 1 within a sample, and a fall in the share of one niche can reflect expansion of others rather than loss of that tissue type. No niche abundance shift survived multiple-comparison correction at *n* = 3 per group. Each point is one biologically independent donor (*n* = 9: NF 3, pRV 3, RVF 3). Box plots are defined as in Fig. 2c, with every replicate overlaid. *P* values are two-sided Kruskal−Wallis per niche, Benjamini−Hochberg adjusted across the 14 niches. Unadjusted and adjusted *P* values per niche are given in Source Data Fig. 3. Exp, mean expression; % Exp, percentage of cells expressing the marker gene. Source data

We profiled nine transmural RV sections (one per patient; 625,305 cells; Supplementary Table 6) on 10x Xenium (477-gene panel; Supplementary Data 10). Annotation against the snRNA-seq reference produced concordant cell type assignments (Fig. 3a,b). Pseudobulk DESeq2 (Supplementary Data 11) broadly corroborated snRNA-seq at the cross-platform FC level (Fig. 3c), with spatial maps confirming consistent assignment across all sections (Fig. 3d). Normalized to panel size, Xeniumʼs phase 2 DEG detection rate was substantially higher than snRNA-seq (approximately 18% panel-significant versus approximately 0.4% genome-wide), consistent with higher Xenium cell counts.

Spatial Gene Expression (SpaGE)^21^ imputation enabled subclustering beyond the 477-gene panel, resolving 58 Xenium subtypes across the 12 Xenium lineages, including refinements across myeloid, EC, fibroblast, cardiomyocyte, mural and epicardial lineages (Supplementary Data 12). Spatial niche analysis yielded 14 niches (Fig. 3e–g and Supplementary Data 13). Niche abundance shifts were modest but limited by statistical power at *n* = 3 per group: one niche reached nominal significance (stressed myocardium; pRV/RVF enriched). Six additional niches showed trends: the adipose-vascular, capillary-rich myocardium, arterial and inflamed adipose stroma niches were enriched in RVF, whereas compact myocardium was highest in pRV, and fibrotic myocardium was lowest in RVF. No niche survived multiple-comparison correction, so these findings require larger-cohort validation. Niche density differences between groups reflect both biological remodeling (cell population shifts during disease progression) and technical factors (variation in tissue section area and cell density across patients) and should be interpreted accordingly.

### A two-phase trajectory of RV failure progression

**Fig. 4:**
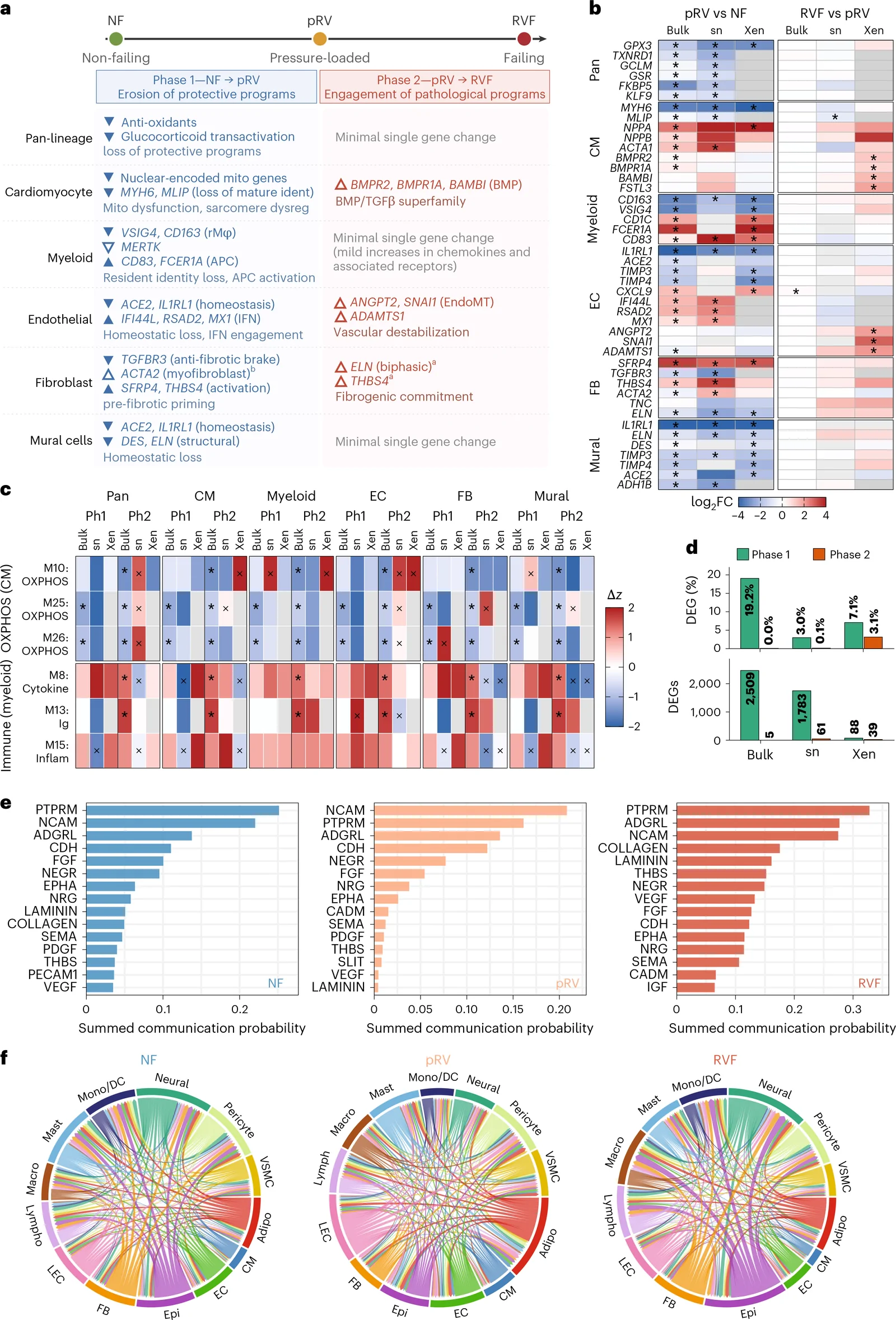
Two-phase transcriptional trajectory of RV failure. **a**, Two-phase model schematic of the NF → pRV (phase 1) and pRV → RVF (phase 2) transitions. Filled triangles mark genes detected with concordance across two or more platforms; unfilled triangles mark genes detected only on one platform. ^a^Significant in phase 2 only within Xenium fibroblast subtypes (*THBS4*, Xen FB Activated and Xen FB NOX4; *ELN*, Xen FB Interstitial) and not at lineage level. ^b^*ACTA2* is significantly increased in snRNA-seq fibroblasts but significantly decreased in bulk RNA-seq, which may reflect *ACTA2* expression by non-fibroblast cells in whole tissue. **b**, Lineage-level key gene heatmaps across bulk, snRNA-seq pseudobulk and Xenium panel-filtered pseudobulk for phase 1 (pRV versus NF log_2_FC) and phase 2 (RVF versus pRV log_2_FC); gray cells, gene not on Xenium 477-gene panel. Asterisks mark genes that are significant on that platform for the indicated contrast (two-sided DESeq2 Wald test, Benjamini–Hochberg-adjusted *P* < 0.05). In the Pan rows, the snRNA-seq and Xenium cells show the mean log_2_FC across the five non-cardiomyocyte lineages (myeloid cells, ECs, fibroblasts, pericytes and smooth muscle cells), and an asterisk marks adjusted *P* < 0.05 in at least two of these lineages. **c**, Within-lineage activity of cardiomyocyte OXPHOS (M25/M26/M10) and myeloid immune (M15/M8/M13) modules across NF → pRV → RVF. Module score, mean of per-gene *z*-scored expression on sample-level pseudobulk (bulk, *n* = 142; per-patient snRNA-seq and Xenium within each lineage). Bulk *P < 0.05; crosses indicates that snRNA/Xenium direction differs from bulk; gray, Xenium score omitted (module has <5 genes measured on the 477-gene panel). Xenium module scores were computed on the imputed Xenium object. Asterisks mark a two-sided Welch *t*-test on sample-level module means (pRV versus NF and RVF versus pRV; *n* = 142), unadjusted across modules. snRNA-seq and Xenium columns are per-patient pseudobulk module scores shown for direction only, not tested. **d**, Phase 1/phase 2 DEG asymmetry: top, significant unique gene symbols per contrast (*P*_adj_ < 0.05, |log_2_FC| > 0.5, baseMean > 50) across bulk, snRNA-seq and Xenium; bottom, significance rate per platform. The underlying DE calls are two-sided DESeq2 Wald tests with Benjamini−Hochberg adjustment. The significance rate is the proportion of tested genes meeting those thresholds on each platform. **e**, CellChat top ligand−receptor pairs across NF, pRV and RVF. **f**, Chord diagrams: cell type communication networks across NF, pRV and RVF. DC, dendritic cell; dysreg, dysregulation; Ig, immunoglobulin; ident, identity; Inflam, inflammatory; Lympho, lymphocyte; Macro, macrophage; mito, mitochondrial; Mono, monocyte; sn, single-nucleus; VSMC, vascular smooth muscle cell; Xen, Xenium. Source data

We organized cell type results around two phases (Fig. 4a): phase 1 (NF → pRV) revealed tissue-wide erosion of protective programs, and phase 2 (pRV → RVF) showed quantitatively fewer changes, resolved as focal cell-type-specific programs (cardiomyocyte oxidative stress/BMP and EC EndoMT/angiogenic activation) best captured by spatial transcriptomics. Indeed, integrating pseudobulk DESeq2 across platforms (Methods) showed phase 1 ≫ phase 2 across all three assays: NF → pRV yielded 2,509 (bulk)/1,783 (snRNA-seq)/88 (Xenium) unique symbol lineage-level DEGs compared to 5/61/39 for pRV → RVF.

#### Phase 1 is multi-lineage and cross-platform concordant

Phase 1 (Fig. 4b) is marked by non-cardiomyocyte loss of genes associated with glucocorticoid receptor (GR) transactivation, notably the GR co-chaperone *FKBP5* (ref. ^22^) (significant in 11/24 non-cardiomyocyte subtypes) alongside the canonical GR targets *ZBTB16* (ref. ^23^) and *KLF9* (ref. ^24^). Cardiomyocytes instead show downregulation of α-myosin heavy chain (*MYH6*) and the lamin-associated gene *MLIP*, with reactivation of the fetal gene program (*NPPA*, *NPPB*, *ACTA1*) evident at the bulk tissue level. More broadly, cardiomyocytes coordinately downregulate nuclear-encoded mitochondrial genes spanning fatty acid oxidation (FAO), the tricarboxylic acid (TCA) cycle, OXPHOS subunits and the mitoribosome as well as the biogenesis regulators PGC-1α (*PPARGC1A*) and ERRα (*ESRRA*). In non-cardiomyocyte lineages, glutathione/thioredoxin redox-buffering genes (*GCLC*, *GCLM*, *GSR*, *TXNRD1*, *SRXN1*, *SLC7A11*) are coordinately downregulated, as is the secreted glutathione peroxidase *GPX3*, which has been causally linked to RVF^25^. The myeloid pool downregulates homeostatic receptors (*CD163*, *VSIG4*; *MERTK* at trend) while inducing conventional dendritic cell markers *CD83*, *CD1C* and *FCER1A*. There is also downregulation of the TGFβ co-receptor *TGFBR3* (ref. ^26^) across all seven fibroblast subtypes and fibroblast activation (*SFRP4* (ref. ^27^), *THBS4* (ref. ^28^)). In ECs, pRV was already marked by an erosion of homeostatic, anti-oxidant and vasoprotective programs—notably, the IL-33 receptor *IL1RL1/ST2* and the matrix metalloprotease inhibitors *TIMP3* and *TIMP4*, with the counter-regulatory RAAS enzyme *ACE2* declining in parallel. snRNA-seq additionally revealed a type I IFN response (*IFI44/IFI44L*, *MX1/MX2*, *OAS3*, *RSAD2*, *STAT1*) that the Xenium panel cannot resolve as the IFN-stimulated genes (ISGs) are off-panel. The one represented chemokine, *CXCL9*, was concordantly induced. Mural cells added early destabilization to the shared vascular-protective loss. We noted downregulation of the structural genes *ELN* (elastin) and *DES* (desmin), the resting stromal metabolic enzyme *ADH1B* and the genes *TIMP3*, *TIMP4*, *IL1RL1*/*ST2* and *ACE2*, with snRNA-seq adding loss of *SMAD3*.

#### Phase 2 is focal and cell type specific, not platform driven

Phase 2 lineage-level significant genes are 39 in Xenium, 61 in snRNA-seq and five in bulk (unique gene symbols per contrast), concordant in direction across platforms where detectable (Fig. 4b). The signal concentrates in two lineages. In cardiomyocytes, Xenium reveals a BMP/TGFβ superfamily signaling program (*BMPR2*, *BMPR1A, FSTL3*, *BAMBI* up). This simultaneous upregulation of receptors and their inhibitors is cardiomyocyte restricted and opposite to the receptor loss of function of heritable PAH. In ECs, phase 2 resolves as an EndoMT/angiogenic destabilization program (*SNAI1*, *ADAMTS1*, *ANGPT2* up; directionally concordant in snRNA-seq). CCR2^−^ macrophage remodeling remains graded rather than a discrete phase 2 step. Module-level analysis (Fig. 4c) resolves two cross-platform concordant programs. A cardiomyocyte OXPHOS module set (M10, M25, M26) is progressively downregulated, whereas myeloid inflammatory, cytokine and immunoglobulin modules (M8, M13, M15) are coordinately induced. Both programs agree in direction between bulk and snRNA-seq when scored within their cell type of origin. The lineage-level DEG count asymmetry is summarized in Fig. 4d.

### Spatially constrained cell−cell communication reveals stage-specific signaling

Cell−cell communication inference (CellChat^29^, applied to per-patient snRNA-imputed Xenium expression under Xenium-derived spatial constraints; Supplementary Data 14) revealed a progressive reorganization of myocardial signaling across the NF → pRV → RVF axis (Fig. 4e,f). In every condition, the highest-probability pathways were contact-mediated adhesion programs (PTPRM, NCAM, ADGRL, CDH), consistent with the dense cellular packing of myocardium and serving as a stable baseline rather than a disease feature. Layered on this baseline, RVF showed a selective gain of ECM signaling: COLLAGEN, LAMININ and THBS, which were low or absent among top-ranked interactions in NF and pRV, rose to prominence specifically in RVF. This matrix signaling gain is concordant with the emergence of the matrifibrocyte-like fibroblast state and frames fibroblast-to-stroma communication as a defining feature of RVF.

### Cardiomyocytes demonstrate mitochondrial dysfunction in RV failure

Subclustering snRNA-seq resolved 10 cardiomyocyte subtypes (Extended Data Fig. 2a,b and Supplementary Data 7), which are defined by distinct functional gene programs by subtype Gene Ontology enrichment and exhibit opposing proportional changes in NF compared to disease states, with subcluster distribution similar between pRV/RVF (Extended Data Fig. 2c,d). SpaGE-imputed label transfer corroborated subtype mapping at single-cell spatial resolution (Extended Data Fig. 2e). Three subtypes expanded in diseased RVs: CM BetaMHC (fetal sarcomere), CM HAND2 (RV-specifier *HAND2* and endothelin receptor *EDNRA*), and CM RORA, with a trend towards expansion in CM NPP (*NPPA*/*NPPB*). This represents reactivation of fetal, developmental and stretch-stress programs. In parallel, ketogenic (CM HMGCS2 (refs. ^30,31^); trend), *MYH6*^+^ baseline (CM MYH6) and atrial-like (CM HTR4; trend) subtypes were all NF enriched and lost in pRV/RVF. *HMGCS2* was suppressed in 12 of 12 Xenium cardiomyocyte subtypes (pRV versus NF). snRNA-seq additionally showed pan-cardiomyocyte *MYH6* downregulation (7/7), with *MLIP* loss (2/7) and *STAT2*/ISG induction in three subtypes, indicating cardiomyocyte-intrinsic innate immune activation.

**Fig. 5:**
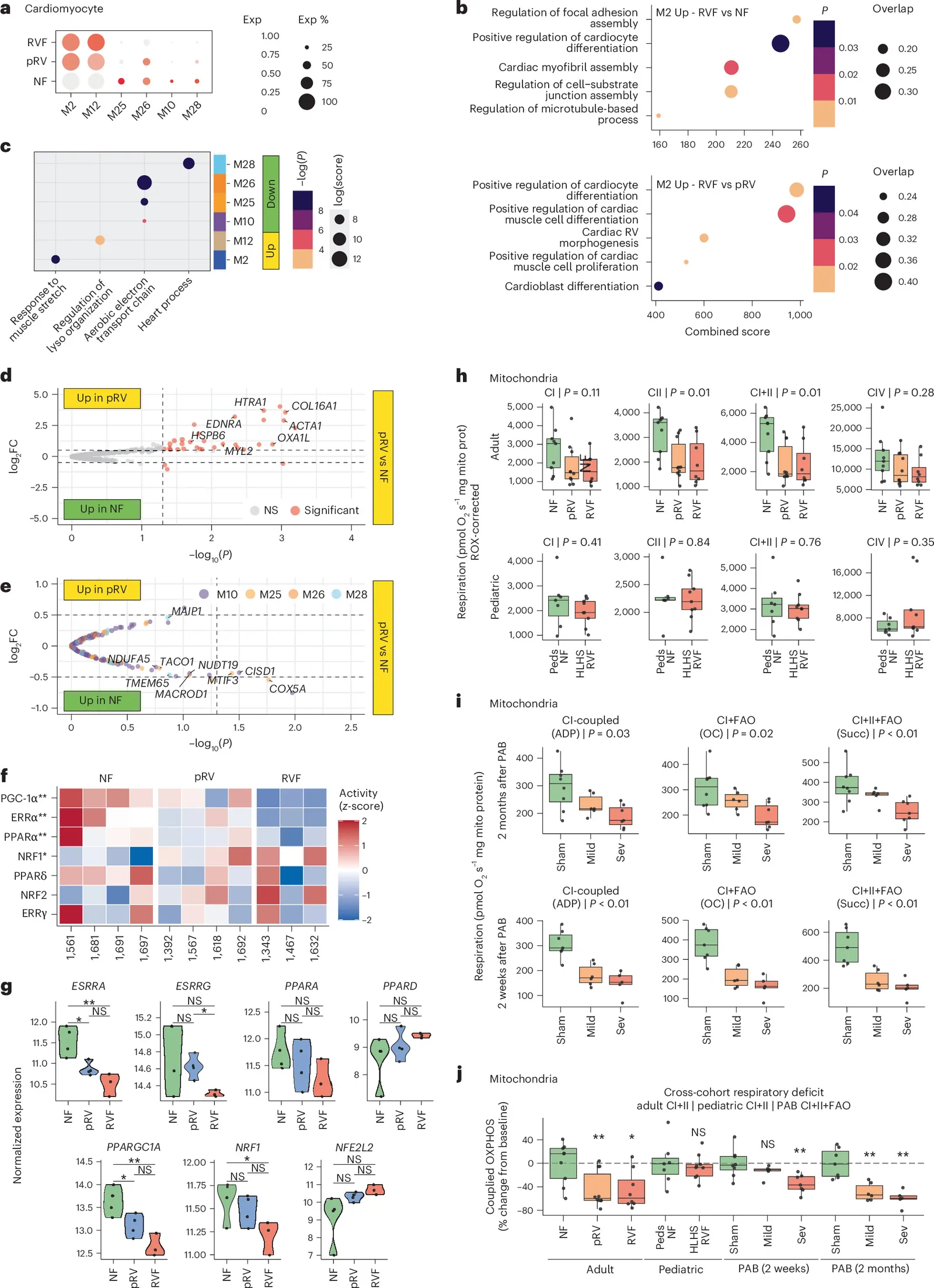
RV cardiomyocyte transcriptional and metabolic remodeling. **a**, Dot plot of CM-enriched bulk WGCNA module scores by CM subcluster and disease state. **b**, Gene Ontology enrichment for CM-specific modules after subsetting to exclusively genes that are both expressed in the given module and differentially enriched between RVF and NF cardiomyocytes. **c**, EnrichR Gene Ontology pathway analysis for M2 module genes. **d**, Volcano plot of M2 module DEGs (CM pseudobulk, pRV versus NF; sample-level DESeq2 with ashr shrinkage). Points are colored gray (NS) or red (significant at *P*_adj_ < 0.05, |log_2_FC| > 0.5). **e**, Module volcano plots for mitochondrial modules (M10, M25, M26, M28). Enrichment in **b** and **c** uses one-sided Fisherʼs exact test, Benjamini−Hochberg adjusted, on module genes also differentially expressed between the contrasted groups. DE in **d** and **e** is per-patient pseudobulk DESeq2 (two-sided Wald test, Benjamini−Hochberg adjustment) on *n* = 11 biologically independent donors (NF *n* = 4, pRV *n* = 4, RVF *n* = 3), with ashr log_2_FC shrinkage for display only. Significance thresholds are *P*_adj_ < 0.05 and |log_2_FC| > 0.5 applied to unshrunken statistics. **f**, CollecTRI/decoupleR transcription factor activity for mitochondrial regulators inferred from CM snRNA-seq. Statistical testing with Jonckheere−Terpstra ordered trend. *q < 0.05; **q < 0.01. PGC-1α *q* = 0.0032, ERRα *q* = 0.0044, PPARα *q* = 0.0079, NRF1 *q* = 0.035. **g**, snRNA-seq pseudobulk violin plots of *ESRRA*, *ESRRG*, *PPARA*, *PPARD*, *PPARGC1A*, *NRF1* and *NFE2L2* expression in CMs. Each point is one biologically independent donor (*n* = 11: NF 4, pRV 4, RVF 3). Values are per-patient DESeq2 variance-stabilized pseudobulk expression. Violins show the full distribution. Brackets show two-sided Welchʼs *t*-tests, unadjusted (NS, *P* ≥ 0.05; *P < 0.05, **P < 0.01): *ESRRA* NF versus pRV *P* = 0.025, NF versus RVF *P* = 0.008; *PPARGC1A* NF versus pRV *P* = 0.038, NF versus RVF *P* = 0.0062; *ESRRG* pRV versus RVF *P* = 0.014; *NRF1* NF versus RVF *P* = 0.048; all other comparisons *P* > 0.05. Exact *P* values for all comparisons are given in Source Data Fig. 5. **h**, Oroboros O2K high-resolution respirometry on freeze/thaw-isolated RV mitochondria, ROX corrected and normalized to starting mitochondrial protein mass (pmol O_2 _s^−1^ per mg mitochondrial protein). Each dot is one patient. Adult cohort (top): NF *n* = 9, pRV *n* = 10, RVF *n* = 8; Kruskal−Wallis *P* values are shown. Pediatric cohort (bottom): pediatric NF (*n* = 7) versus HLHS-palliated failing (*n* = 9); Mann−Whitney pairwise *P* values. Box plots are defined as in Fig. 2c. Each dot is one biologically independent patient. Kruskal−Wallis and Mann−Whitney tests are two-sided, unadjusted across respiratory states. **i**, Respirometry in PAB model at 2-month (top) and 2-week (bottom) timepoints versus sham, using a substrate addition protocol (PM → ADP → Cyt C → OC → Succ) on tissue homogenate. Complex I + II + FAO maximal coupled respiration (Succ-driven) is the headline measure. Two-week: sham *n* = 8, mild *n* = 6, severe *n* = 7. Two-month: sham *n* = 7, mild *n* = 6, severe *n* = 5. Each dot is one animal; Kruskal−Wallis *P* values are shown. Box plots are defined as in **h**, and each dot is one biologically independent animal. Kruskal−Wallis tests are two-sided and unadjusted across respiratory states. **j**, Cross-cohort summary of RV mitochondrial OXPHOS, normalized to within-cohort controls. Maximal coupled respiration is reduced in adult RVF and mouse PAB but preserved in pediatric HLHS-palliated failing RV. Each point is one biologically independent patient or animal, normalized to the within-cohort control mean. Box plots are defined as in **h**. Succ-driven maximal coupled respiration versus the within-cohort reference, two-sided Mann−Whitney, unadjusted: adult pRV versus NF −42.4%, *P* = 0.0030; adult RVF versus NF −40.4%, *P* = 0.015; PAB mild versus sham −13.8%, *P* = 0.18 (2-week) and −49.0%, *P* = 0.0023 (2-month); PAB severe versus sham −34.7%, *P* = 0.0037 (2-week) and −59.6%, *P* = 0.0025 (2-month); pediatric HLHS-palliated failing versus pediatric NF −2.3%, *P* = 0.84. *P < 0.05 and **P < 0.01. Asc/TMPD, ascorbate/tetramethyl-*p*-phenylenediamine; CI/CII/CIV, mitochondrial respiratory complexes I, II, IV; CI + II, coupled complex I + II respiration; Exp, normalized *z*-score of module score; Exp %, percent of nuclei expressing given module; mito prot, mitochondrial protein; OC, octanoyl-carnitine; Peds, pediatric; PM, pyruvate + malate; Sham, sham-operated control mice; Sev, severe; Succ, succinate. Source data

Cardiomyocyte WGCNA modules segregated NF from pRV/RVF (Fig. 5a,b and Extended Data Fig. 2f). pRV/RVF-elevated M2 enriched for contractile/focal adhesion and structural/ECM genes (Fig. 5c,d). M12 contained *TNNI3*/*TNNI3K* (phase 1 bulk DEGs) plus *BMPR2*/*MYH7* (subthreshold). Suppression of M10/M25/M26 (Fig. 5e) across cardiomyocyte subtypes and cardiomyocyte pseudobulk DESeq2 analysis both demonstrate a modest (median log_2_FC ≅ −0.22) but broadly consistent transcriptional downregulation of nuclear-encoded mitochondrial components (for example, *NDUF*, *SDHC*, *COX* and *ATP5* subunits) without bias toward any single complex. The aggregate MitoCarta3.0 module score declined across cardiomyocyte subclusters and disease stages (Extended Data Fig. 2g). Among mitochondrial biogenesis regulators, *ESRRA* (ERRα) was the standout, concordantly suppressed across modalities with reduced transcript and inferred regulon activity in cardiomyocytes (Fig. 5f,g) and significant suppression in bulk RV. *PPARGC1A* (PGC-1α) was suppressed in cardiomyocytes at both the transcript and activity level. *PPARA* showed reduced inferred activity with a concordant but non-significant expression trend. For *NRF1* and *PPARD*, inferred activity and measured expression diverged. Because CollecTRI/decoupleR activity is inferred from target gene expression rather than the regulatorʼs own transcript and is sparsely parameterized for ligand-regulated and cofactor-regulated metabolic regulators, we weight the concordant evidence most heavily: the suppression of ERRα and PGC-1α and the coordinated decline of their nuclear-encoded mitochondrial targets.

Respirometry on isolated RV mitochondria (Fig. 5h, top panels, and Supplementary Data 15) demonstrated coordinated ETC suppression in RVF consistent with previous literature^32,33^: maximal OXPHOS capacity (complex I + II) declined similarly in pRV and RVF relative to NF (pRV −42%, RVF −40%), with parallel approximately 35−37% loss of complex II-driven respiration (Kruskal−Wallis significant). Complex I (−32% to −39%) and complex IV (−22% to −28%) showed non-significant trends down, and non-mitochondrial respiration was unchanged. The largest change in respirometry capacity occurring at NF → pRV mirrors the dominant mitochondrial transcriptional changes occurring at this stage.

The CM HAND2 population expanded in pRV/RVF (Extended Data Fig. 2c), but per-patient expression of *HAND2* was not significantly different, whereas *EDNRA* was induced in pRV and RVF (Extended Data Fig. 2h). Wheat germ agglutinin (WGA) cross-sectional area (Extended Data Fig. 2i and Supplementary Data 16) confirmed progressive cardiomyocyte hypertrophy across NF → pRV → RVF (median minimum Feret diameters 55.8 → 73.9 → 102.5 µm). Xenium localized *NPPA*/*NPPB* induction to the endocardial border (Extended Data Fig. 2j).

### Fibroblasts activate early in RV failure

Fibroblasts were the most transcriptionally responsive snRNA-seq population (1,083 pRV versus NF DEGs; 806 RVF versus NF DEGs). Subclustering snRNA-seq resolved seven literature-grounded subtypes (Extended Data Fig. 3a,b): Fb Resident, Adventitial, Elastogenic, Interstitial, Stressed, Pro-fibrotic and Anti-fibrotic, with fibroblast subtype proportions generally stable across disease states (Extended Data Fig. 3c). The snRNA-seq trajectory was front loaded and consistent with Xenium (Extended Data Fig. 3d): in phase 1, there was downregulation of the TGFβ co-receptor *TGFBR3* along with *GPX3* and the matrix/elastogenic genes *ELN* and *TIMP3*. Simultaneously, the canonical myofibroblast/matricellular markers *THBS4* (ref. ^34^) and *ACTA2* (ref. ^35^) and the activated fibroblast marker *SFRP4* were induced.

**Extended Data Fig. 4:**
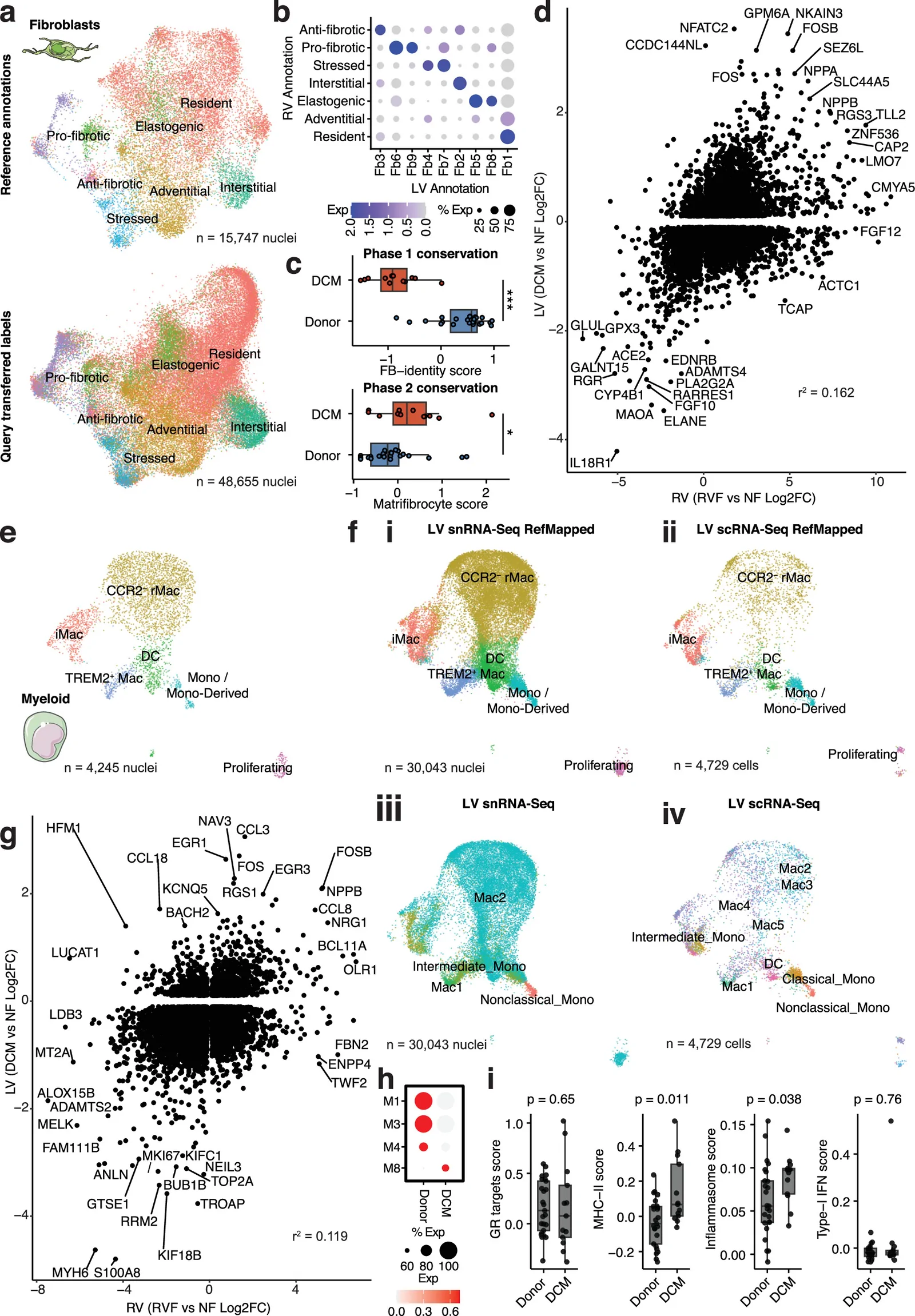
Left ventricular failure (DCM) comparison. (**a**) UMAP of integrated human RV fibroblast lineages and cell sub-types (top), as well as reference mapped LV data (bottom). (**b**) Fibroblast subtype concordance between ventricles. (**c**) Conservation of fibroblast Phase 1 and Phase 2 programs in LV snRNA-seq (calculated from Koenig et al. 2022; Donor vs DCM, per-patient pseudobulk; CPM, log1p, per-gene z-scored across patients, mean across genes in module). (top) Phase 1: Koenig donor-FB identity score (*GPX3*, *PID1*, *TGFBR3*, *ACSM3*, *APOD*; expected to erode, that is down in DCM). (bottom) Phase 2: Fu 2018 matrifibrocyte commitment score (*COMP*, *CILP*, *CHAD*; expected to rise, that is up in DCM). Each dot is one patient; box = IQR, whiskers = 1.5× IQR. Donor vs DCM tested by Wilcoxon rank-sum. * p < 0.05; ** p < 0.01; *** p < 0.001. (**d**) Scatter plot of log2 fold changes for the human LV fibroblast (DCM vs NF) vs human RV fibroblast (RVF vs NF) comparison by gene. (**e**) UMAP of integrated human RV myeloid lineages and cell sub-types. (**f**) Reference mapping of single-nuclei (**i**) and single-cell (**ii**) LV myeloid dataset onto human RV dataset with corresponding RV annotations. (**g**) Cross-chamber concordance scatter for the four myeloid bulk-WGCNA modules (M1, M3, M4, M8). For each module, per-gene log2FC was computed by Seurat FindMarkers in the RV myeloid object and in the LV (Koenig 2022) myeloid object, restricted to genes detected on both platforms. Notable genes are labeled. (**h**) Bulk-WGCNA myeloid modules (M1, M3, M4, M8) projected onto the LV (Koenig 2022) myeloid atlas via Seurat AddModuleScore. Dot plot of mean per-cell module score (color) and percent expressing (size) by LV myeloid subtype × disease state (Donor vs DCM), allowing the four RV-derived myeloid programs to be assessed for cross-chamber conservation. (**i**) Per-patient pseudobulk module-score box plots in LV myeloid cells snRNA-seq for the four RV-derived myeloid programs: GR transactivation, MHC-II/antigen presentation, NLRP3 inflammasome, and type-I interferon. Each dot = one patient (per-patient mean module score over LV myeloid nuclei). Donor vs DCM tested by Wilcoxon rank-sum. Source data

None of the snRNA-seq, bulk or panel-filtered Xenium data yielded significant fibrogenic DEGs in RVF versus pRV at the lineage level. Xenium fibroblast subtype resolution exposed a focal phase 2 program restricted to activated/matricellular subsets involving *THBS4*, *TNC* and *PRG4* (snRNA-seq directions concordant, not significant) (Extended Data Fig. 3e). Two literature-defined module scores anchored the two-phase split: the Koenig et al.^10^ donor fibroblast identity signature collapsed sharply at NF → pRV with no further drop at decompensation (Extended Data Fig. 3f), whereas the Fu et al.^34^ matrifibrocyte commitment signature was flat at NF → pRV and trended upward only at pRV → RVF (Extended Data Fig. 3g). This suggests that fibroblast identity erosion is phase 1 specific, whereas fibrogenic commitment is phase 2 specific. Cross-platform similarity (Extended Data Fig. 3h,i) was strongest for Stressed and Interstitial subtypes. Spatial neighborhood analysis (Extended Data Fig. 3j–l) placed FB Stressed, Matrix, Intermediate and CILP in myeloid-enriched niches (Xen FB Matrix–myeloid Pearsonʼs *r* = 0.93). Massonʼs trichrome/Sirius Red histology confirmed progressive fibrosis (+5.9% area per stage; Extended Data Fig. 3m). In RVF, the fibrotic myocardium niche occupies a smaller fraction of section area (Fig. 3g). This does not necessarily represent less fibrosis, as niche proportions are compositional. They describe how section area is partitioned between neighborhoods, not how much matrix each neighborhood contains. The rising histologic collagen burden (Extended Data Fig. 3m), the pRV → RVF gain of ECM signaling (Fig. 4e) and the matrifibrocyte commitment trend (Extended Data Fig. 3g) all measure matrix content or fibroblast state and are compatible with fibrotic remodeling intensifying within existing neighborhoods while the stressed myocardium and vascular niches expand their share of the section. Fibroblast subclusters identified from previous LV studies were similarly recovered (Extended Data Fig. 4a–d), indicating conserved fibroblast biology across ventricles.

### Mural cells lose vasoprotective programs in RV failure

Mural snRNA-seq subclustering (Extended Data Fig. 3n) separates pericytes from smooth muscle. Pericytes undergo phase 1 loss of vascular-protective programs spanning metabolic, homeostatic, anti-protease, anti-inflammatory and anti-oxidant functions (gene lists in Extended Data Fig. 3o and Supplementary Data 8 and 9), concordant across bulk, snRNA-seq and Xenium pseudobulk. The loss is largely pericyte restricted. Pericyte snRNA-seq additionally showed downregulation of the TGFβ co-receptor *TGFBR3* and the hypoxia pathway gene *HIF3A*. We quantify the protective program loss by a curated pseudobulk module score (*TIMP3*, *IL1RL1*, *GPX3*, *ELN*; Extended Data Fig. 3p), whose NF/pRV/RVF trajectory parallelled the fibroblast module. At the lineage level, mural cells did not demonstrate significant phase 2 changes, whereas sublineage analyses by snRNA-seq pseudobulk reveal IEG/AP-1 stress and partial contractile reversion as the dominant gene groupings (heatmap in Extended Data Fig. 3q). Xenium subtype resolution (Extended Data Fig. 3r) resolves focal subset-restricted hits: *MYH11* loss in Pericyte_KCNJ8, Pericyte_THBS4 and VSMC_Inflamed; *CNN1* induction in VSMC_Arterial; and *NR4A3* induction in VSMC_Inflamed (cross-platform concordance in Extended Data Fig. 3s), supporting a focal phase 2 program rather than a pan-mural shift.

### The immune compartment remodels in RV failure with resident macrophage identity loss and cumulative inflammatory transition

**Fig. 6:**
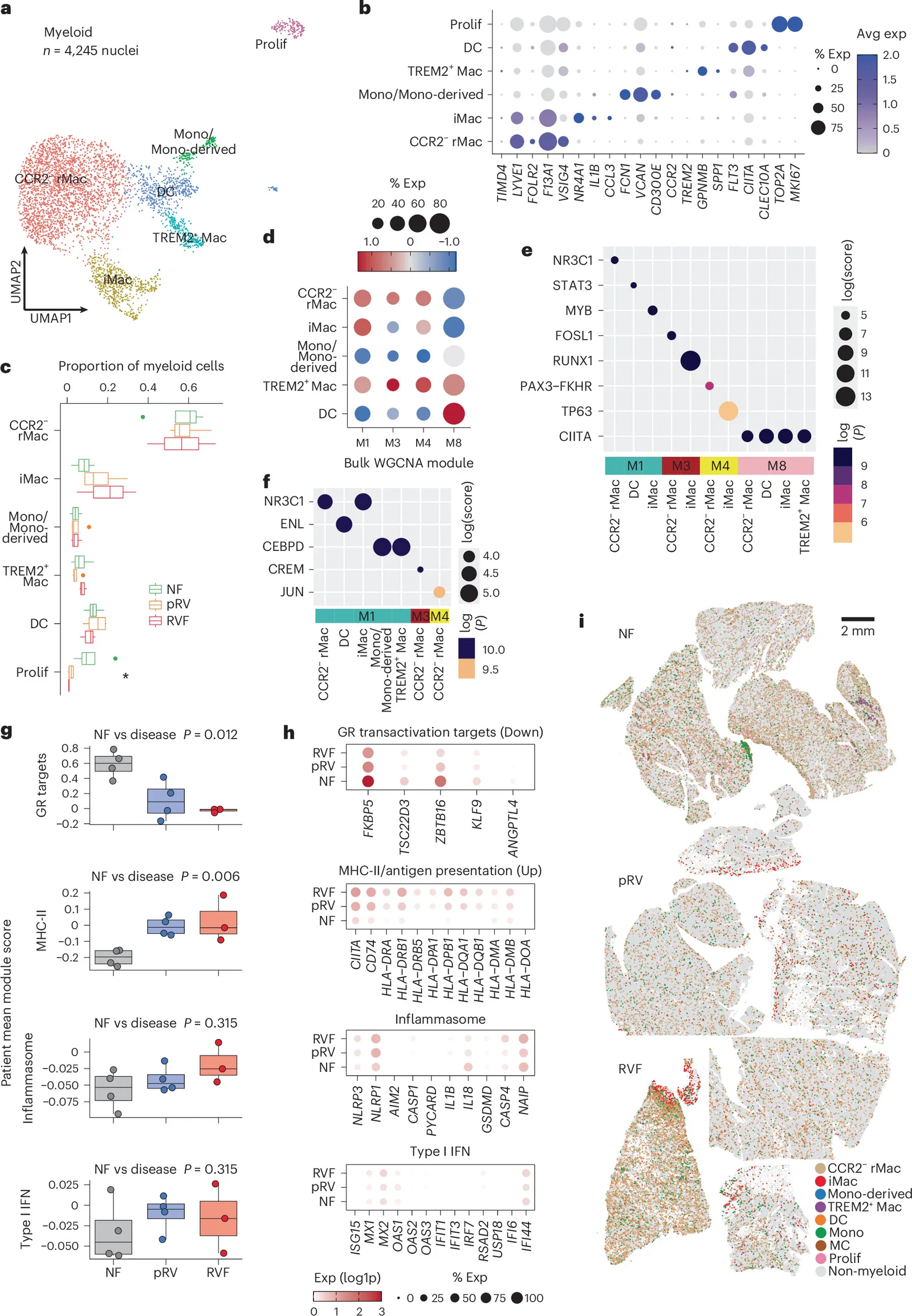
Subclustering and analysis of myeloid populations in RV failure. **a**, Myeloid subclustering UMAP with six subtypes: CCR2^−^ resident macrophage (rMac), inflammatory macrophage (iMac), monocyte and monocyte-derived macrophage (Mono/Mono-derived), TREM2^+^ macrophage, DC and proliferating (prolif) myeloid. **b**, Dot plot of canonical marker genes per myeloid subtype. Mean expression (color) and percent of cells expressing (size). **c**, Cell type frequency across disease states (NF/pRV/RVF; *n* = 4/4/3). Box plots are defined as in Fig. 2c. The asterisk marks two-sided Kruskal−Wallis *P* < 0.05 per subtype, uncorrected across the six subtypes. Exact *P* values are prolif myeloid *P* = 0.024, TREM2^+^ macrophage *P* = 0.38, iMac *P* = 0.41, DC *P* = 0.56, Mono/Mono-derived *P* = 0.97 and CCR2^−^ rMac *P* = 0.98. **d**, Module expression dot plot of the four myeloid-relevant bulk WGCNA modules (M1, M3, M4, M8) by myeloid cell type. **e**, ChEA transcription factor enrichment for module genes upregulated in RVF versus NF. **f**, ChEA transcription factor enrichment for module genes downregulated in RVF versus NF. Transcription factor enrichment in **e** and **f** used one-sided Fisherʼs exact test, Benjamini−Hochberg adjusted on module genes upregulated or downregulated in RVF versus NF. **g**, Pseudobulk module score violin plots for the four myeloid programs across NF/pRV/RVF (*n* = 4/4/3): GR transactivation, MHC-II/antigen presentation, NLRP3 inflammasome and type I IFN across all myeloid subtypes. Pairwise Wilcoxon and pooled NF versus disease (pRV + RVF) *P* values are shown. Values are per-patient pseudobulk module scores across all myeloid subtypes. Box plots are defined as in Fig. 2c. Pairwise and pooled comparisons are two-sided Wilcoxon rank-sum tests, unadjusted across the four programs. Exact *P* values are given in Source Data Fig. 6. **h**, Companion dot plots of canonical genes from each of the four programs shown across NF/pRV/RVF. Shared color (expression, white → red) and size (% expressed) legends. Color is mean expression; size is the percentage of expressing cells. **i**, Spatial localization of myeloid subtypes in the full Xenium dataset (625,305 cells). Non-myeloid cells are shown in light gray; CCR2^−^ rMac and macrophage C1q are combined. Avg exp, average expression; ChEA, ChIP-X Enrichment Analysis; Exp, normalized *z*-score of module score; % Exp, percentage of nuclei expressing the gene (**b**,**h**) or expressing the given module with *z*-score > 0 (**d**). Source data

Myeloid subclustering yielded six snRNA-seq populations (Fig. 6a–c and Supplementary Data 7): CCR2^−^ resident macrophage, inflammatory macrophage (iMac), monocyte/monocyte-derived, TREM2^+^, dendritic and proliferating; mast cells were only identified by Xenium. CCR2^−^ resident macrophages expressed the conserved TLF framework (*TIMD4*, *LYVE1*, *FOLR2*)^36^. iMac cells retained resident markers while strongly expressing immediate-early genes (*NR4A1/NR4A2/NR4A3*, *FOS*, *EGR1*) and, more modestly, NF-κB target cytokines (*CCL3*, *CCL4*, *IL1B*) while also lacking monocyte lineage markers, reflecting an activated-resident rather than a recruited-monocyte state. TREM2^+^ cells expressed a lipid-associated signature^37^ implicated in phagocytosis of cardiomyocyte-ejected dysfunctional mitochondria^38,39^.

Pseudobulk DESeq2 identified CCR2^−^ resident macrophages as the most responsive myeloid population (169 pRV versus NF snRNA-seq DEGs; 10 Xenium). Phase 1 featured cross-platform loss of resident/scavenger markers (*VSIG4*, *CD163*). No individual significant DEG emerged from snRNAseq analysis of pRV → RVF, but the inflammatory transition appeared cumulative from NF → pRV → RVF. From NF → RVF, resident macrophages induced the NR4A immediate-early family, the activation marker *CD83* and coordinated MHC-II induction (*HLA-DRB1* significant), supporting increased antigen presentation capacity. Myeloid WGCNA (Fig. 6d–f) recapitulated this pattern: RVF-upregulated M8 enriched for MHC-II master *CIITA*^40^, whereas the RVF-downregulated *NR3C1* target-enriched M1 module captured the same coordinated resident identity loss. After co-embedding RV and LV (from Koenig et al.^10^) myeloid cells (Extended Data Fig. 4e,f), the same resident/scavenger markers, *VSIG4* and *CD163*, fell in both ventricles (Extended Data Fig. 4g,h), identifying erosion of resident macrophage identity as a shared, chamber-independent feature of heart failure.

Within the myeloid compartment, the dominant transcriptional change was a coordinated, pan-myeloid downregulation of GR transactivation targets. The canonical GR transactivation targets *FKBP5*, *TSC22D3*, *ZBTB16* and *KLF9*— together with *ANGPTL4*—were concordantly downregulated as an early stepwise shift^23^, established at the pre-failure (pRV) stage and sustained in RVF (Fig. 6g,h and Supplementary Data 17), and this decline was consistent across myeloid subtypes rather than confined to a single population. In parallel, MHC-II/antigen presentation genes, led by the master regulator *CIITA*, were induced with the same early kinetics. Critically, this rewiring was program specific rather than a generalized inflammatory activation, and two innate immune axes that commonly accompany myocardial stress were explicitly unchanged across disease stage. The NLRP3 inflammasome, a canonical driver of heart failure inflammation, showed minimal induction of its sensors, adaptor, caspases or effector cytokines (*IL1B*, *IL18*)^41,42^. Likewise, the type I IFN/antiviral ISG response, increasingly reported in failing and inflamed myocardium, remained flat, arguing against an IFN-driven component to the myeloid response^43,44^. Thus, the failing RV myeloid compartment shows a focused, coordinated shift in GR target gene expression, in the absence of inflammasome or type I IFN activation. Comparison with failing LV (dilated cardiomyopathy (DCM)) myeloid cells shows that this configuration is only partly conserved across ventricles: MHC-II/antigen presentation induction is shared, and the type I IFN response stays low in both, whereas the loss of GR target gene expression and the absence of inflammasome activation are RV specific. LV DCM myeloid cells instead retain GR target expression and show inflammasome induction (Extended Data Fig. 4i).

Spatial Xenium refined myeloid taxonomy into eight subpopulations, adding Mac C1q and Mast Cell. Residents distributed broadly, and iMacs co-localized with monocytes at the tissue periphery—a spatial pattern that cannot by itself distinguish phenotypic transition of resident macrophages from monocyte-derived recruitment (Fig. 6i). TLF marker retention in iMacs, iMac non-expansion (not significant by Kruskal−Wallis) and CCR2^−^ contraction are consistent with phenotypic transition, but the iMac/monocyte spatial co-occurrence and absence of imaging-based lineage tracing leave the relative contribution of resident cell transition versus monocyte recruitment unresolved.

### ECs suppress anti-oxidant programs and activate in RV failure

**Fig. 7:**
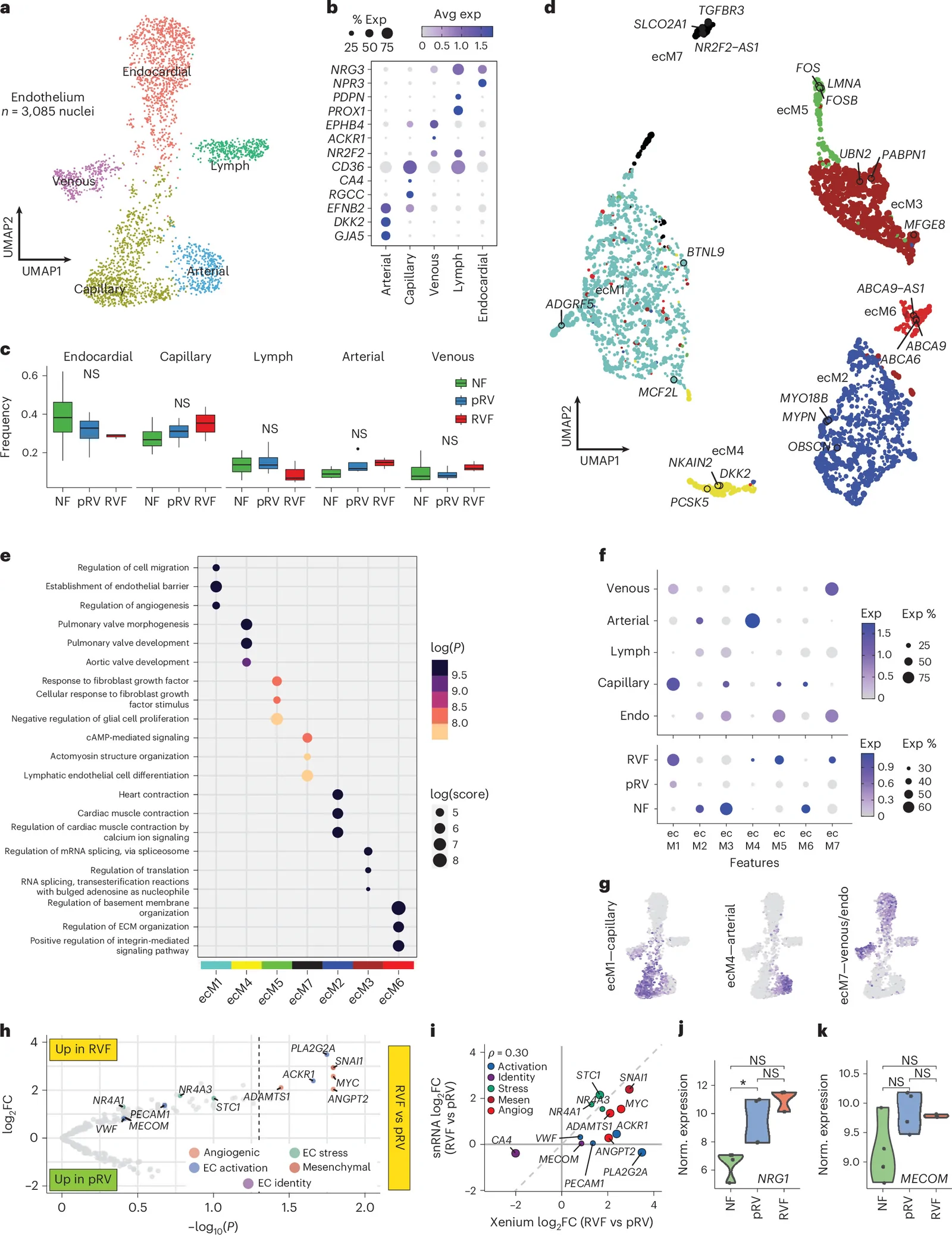
Subclustering and analysis of endothelial populations in RV failure. **a**, EC subclustering UMAP, five subtypes (arterial, capillary, venous, lymph and endocardial). **b**, Dot plot of curated canonical EC subtype marker genes. **c**, Per-subtype frequency box plots across NF, pRV, RVF (ANOVA; *n* = 4/4/3). Box plots are defined as in Fig. 2c. *P* values are one-way ANOVA across the three disease groups computed independently per EC subtype, unadjusted across subtypes. **d**, hdWGCNA on EC snRNA-seq resolved seven modules. Per-module sizes, color labels and hub genes are detailed in text and Supplementary Data 18. **e**, Gene Ontology enrichment for capillary core (ecM1), arterial (ecM4) and venous/inflamed (ecM7) modules. Enrichment used one-sided Fisherʼs exact test, Benjamini−Hochberg adjusted, on module hub genes. **f**, EC module score dot plot by subtype × disease group. **g**, FeaturePlot of ecM1, ecM4 and ecM7 on the EC UMAP. **h**, Xenium pan-EC pseudobulk volcano (RVF versus pRV) highlighting curated phase 2 candidates by program. DE is Xenium pan-EC per-patient pseudobulk DESeq2 (two-sided Wald test, Benjamini−Hochberg adjusted), restricted to the 477-gene panel. **i**, Cross-platform concordance scatter for phase 2 candidate genes: Xenium pan-EC log_2_FC (*x*) versus snRNA-seq pan-EC log_2_FC (*y*), RVF versus pRV; Spearmanʼs rank correlation is reported. **j**, *NRG1* expression in endocardial EC across NF/pRV/RVF (pseudobulk DESeq2-VST per donor; pairwise Wilcoxon). NF versus pRV *P* = 0.029, NF versus RVF *P* = 0.057, pRV versus RVF *P* = 0.23. **k**, *MECOM* expression in arterial EC across NF/pRV/RVF (pseudobulk DESeq2-VST per donor; pairwise Wilcoxon). NF versus pRV *P* = 0.11, NF versus RVF *P* = 0.40, pRV versus RVF *P* = 1.0. Angiog, angiogenic; Exp, normalized *z*-score of module score; Exp %, percent of nuclei expressing the given module with *z*-score > 0; Mesen, mesenchymal; Norm., normalized. Source data

EC snRNA-seq subclustering yielded five clusters (Fig. 7a,b and Supplementary Data 7), including an endocardial population (*LEPR*, *NPR3*). snRNA-seq showed EC expansion in pRV/RVF driven by capillary and arterial ECs, with relative contraction of endocardial and lymphatic ECs (Fig. 7c). Xenium favored arterial expansion with stable capillary proportions, reflecting platform-specific recovery biases. High-dimensional WGCNA (hdWGCNA)^45^ resolved seven EC modules (catalog in Methods and Supplementary Data 18), with ecM1/ecM4/ecM7 (capillary/arterial/venous EC programs) being upregulated in RVF (Fig. 7d–g).

Phase 1 EC resolved into two cross-platform programs (Fig. 4a,b). Phase 1 loss of homeostatic features included suppression of vasoprotective genes (*IL1RL1*, the dominant anti-oxidant *GPX3*, *ACE2* and *EDN1*). ISGs rose in parallel, with concordant induction of *IFI44L*, *RSAD2*, *IFI44*, *OAS1*/*OAS2*/*OAS3*, *MX1*, and *STAT1* by snRNA-seq and bulk (not included in the Xenium 477-gene panel). We curated 13 phase 2 EC candidate genes spanning five functional programs: angiogenic destabilization (*ANGPT2*, *MYC*, *ADAMTS1*), EC stress (*STC1*, *NR4A1*, *NR4A3*), EC activation (*ACKR1*, *PLA2G2A*), mesenchymal (*SNAI1*), and EC identity (*VWF*, *PECAM1*, *MECOM*, *CA4*), and we tested cross-platform concordance in EC pan-pseudobulk (RVF versus pRV; Fig. 7h,i). The functional phase 2 program validated cleanly: angiogenic destabilization (3/3 Xenium-sig + sn-concordant), the isolated *SNAI1* event, *ACKR1* biphasic reduction in phase 1 and recovery in phase 2 and *CA4* capillary identity erosion; EC stress IFN effector genes (IEGs) were direction concordant but false discovery rate (FDR) modest at the lineage level. Three EC identity/activation candidates (*VWF*, *MECOM*, *PLA2G2A*) showed Xenium signal without snRNA-seq direction concordance.

At the sublineage level, cardioprotective *NRG1* was strongly induced in endocardial ECs in phase 1 (Fig. 7j). The arterial specifier *MECOM*^46,47^ visually trended up in arterial ECs snRNA-seq in phase 1 (Fig. 7k; notably, phase 2 in Xenium); because *MECOM* regulates arteriovenous specification in human cardiac endothelium, adult *MECOM* induction may explain capillary/arterial-biased expansion.

### A mouse PAB model recapitulates some but not all aspects of human RVF

**Extended Data Fig. 5:**
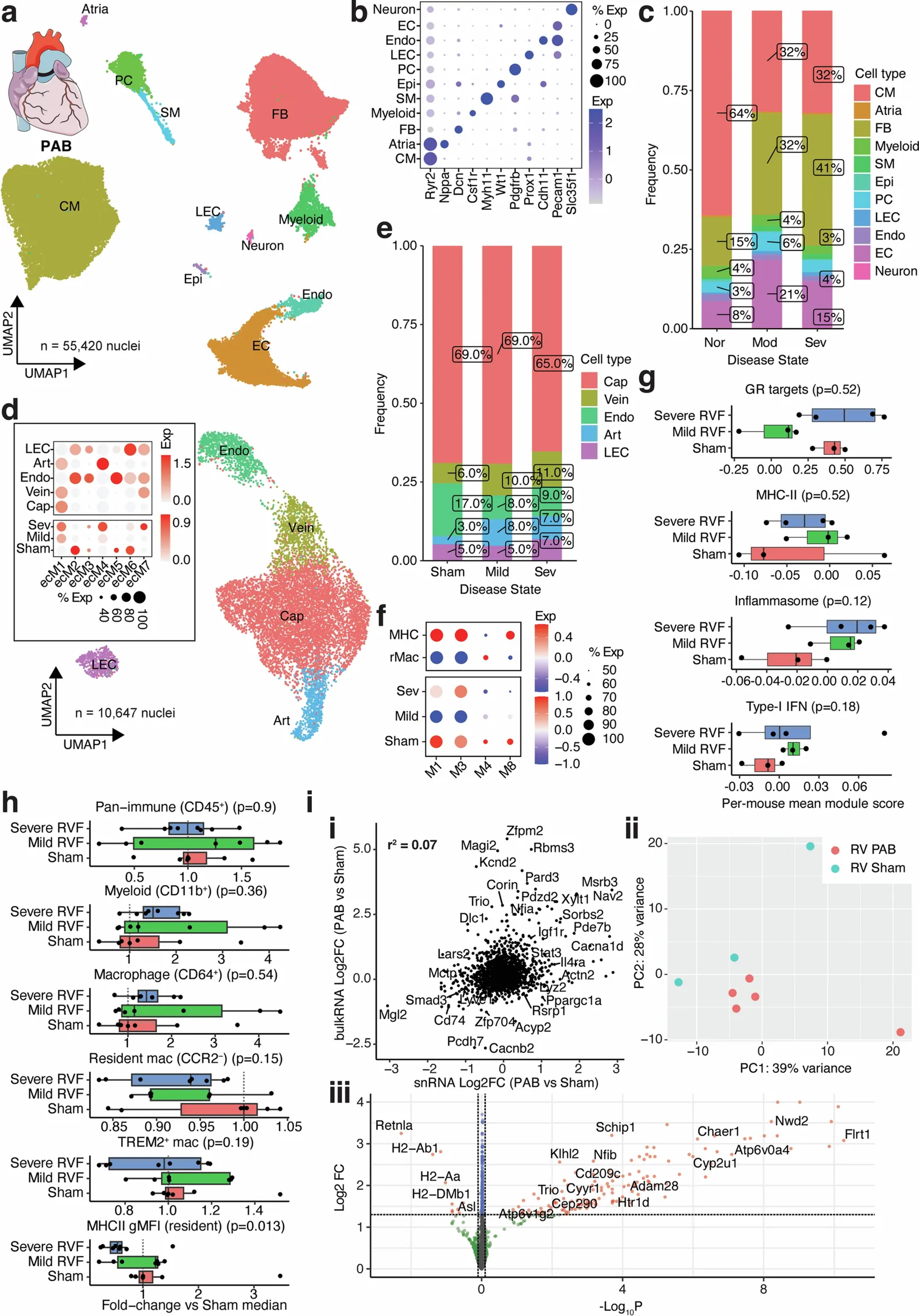
Mouse PAB model: immune and vascular characterization. (**a**) UMAP of mouse PAB dataset nuclei (n = 55,420) shows 11 different cell types present in mouse RVs. (**b**) Annotation of cluster identity using marker genes for unique cell types. (**c**) Stacked barplot showing cell type proportion by disease state, normalized at the subject level. (**d**) UMAP of mouse PAB endothelial and endocardial populations, showing recovery of similar cell types in the mouse dataset as the adult one. The inset demonstrates normalized Seurat-computed module expression scores of previously identified human EC hdWGCNA modules in the mouse dataset, split by cell-type (top) and disease state (bottom). (**e**) Stacked barplot showing cell type proportion by disease state of mouse ECs, normalized at the subject level. (**f**) Normalized Seurat-computed module expression scores of previously identified human myeloid WGCNA modules in the mouse dataset, split by cell-type (top) and disease state (bottom). (**g**) Cross-species myeloid programs in the mouse PAB model. Per-mouse pseudobulk module scores for the four corrected human-RVF Phase-1 myeloid programs (n = 10 mice). GR targets, MHC-II, inflammasome, and type-I IFN are scored across pooled myeloid. NF (Sham) vs disease (Mild + Severe RVF pooled) Wilcoxon p shown. (**h**) Flow-cytometry validation of cardiac immune populations in the mouse PAB model (Sham/Mild RVF/Severe RVF). Per-mouse fold-change relative to the within experiment Sham median for leukocytes (CD45^+^ events as a fraction of all recorded events), myeloid cells (CD11b^+^Ly-6G^−^), macrophages (CD64^+^), CCR2^−^ resident macrophages, TREM2^+^ macrophages, and MHC-II geometric mean fluorescence intensity (gMFI) on CCR2^−^ residents; n = 7/7/8 mice (1 sham excluded due to quality control). Log-scale linear-trend p across severity is shown. (**i**) Orthogonal validation of myeloid single-nuclei mouse results by bulk RNA-sequencing of flow sorted mouse cardiac macrophages. (**i**) Transcriptional changes between PAB and sham RV macrophages are similar in the bulk RNA-sequencing and single-nuclei datasets. (**ii**) In the bulk RNA-sequencing data PAB RV macrophages cluster separately from sham RV macrophages. (**iii**) PAB is associated with downregulation of MHCII-related transcripts. Source data

PAB isolates the RV-specific consequence of sustained pressure overload, the hemodynamic insult shared with pulmonary hypertension and other right-sided afterload diseases, in the absence of pulmonary vascular and systemic confounders or LV failure. We profiled snRNA-seq of sham (*n* = 3) and PAB with mild or severe RVF (*n* = 3, *n* = 4; stratified by RV dilation (RV end-diastolic area); Supplementary Table 7, Extended Data Fig. 5a,b and Supplementary Data 19). Most cell populations were recovered except adipocytes^48^, with EC expansion and cardiomyocyte contraction across disease states paralleling the human dataset (Extended Data Fig. 5c). EC expansion was largely arterial in mice versus capillary/arterial in humans (Extended Data Fig. 5d,e), and human EC hdWGCNA modules embedded consistently. Myeloid cells (<2% of total cells) separated into MHC-II*-*high and resident macrophages. PAB-induced shifts in RV myeloid cells were broadly discordant with human RVF. Across single-nucleus myeloid cells, PAB did not reproduce the human antigen presentation response: the M8 antigen-presenting module was not induced (Extended Data Fig. 5f), and the pooled MHC-II module score was unchanged across severity (Extended Data Fig. 5g). Also, *NR3C1* (GR) target genes showed no repression (Extended Data Fig. 5g). Because antigen-presenting macrophages are a minor fraction of RV myeloid cells, we tested MHC-II directly in residents with two orthogonal assays, both of which showed active suppression in PAB RV resident macrophages. Flow cytometry showed that surface MHC-II (I-A/I-E geometric mean fluorescence intensity) on CCR2^−^ resident macrophages declined in a severity-dependent manner (*P* = 0.013) even as leukocyte, myeloid, macrophage and resident macrophage frequencies remained unchanged across severity (Extended Data Fig. 5h). Genetic pulse−chase fate mapping (*Cx3cr1*-CreER;*Rosa26*-tdTomato) with flow-sorted bulk RNA-seq of long-lived resident macrophages confirmed MHC-II suppression in PAB RV resident macrophages (Extended Data Fig. 5i and Supplementary Data 20)^49^. Thus, the antigen presentation/MHC-II program induced in human RVF resident macrophages is instead suppressed in the mouse PAB RV, establishing inter-species divergence of the RVF myeloid signature.

**Extended Data Fig. 6:**
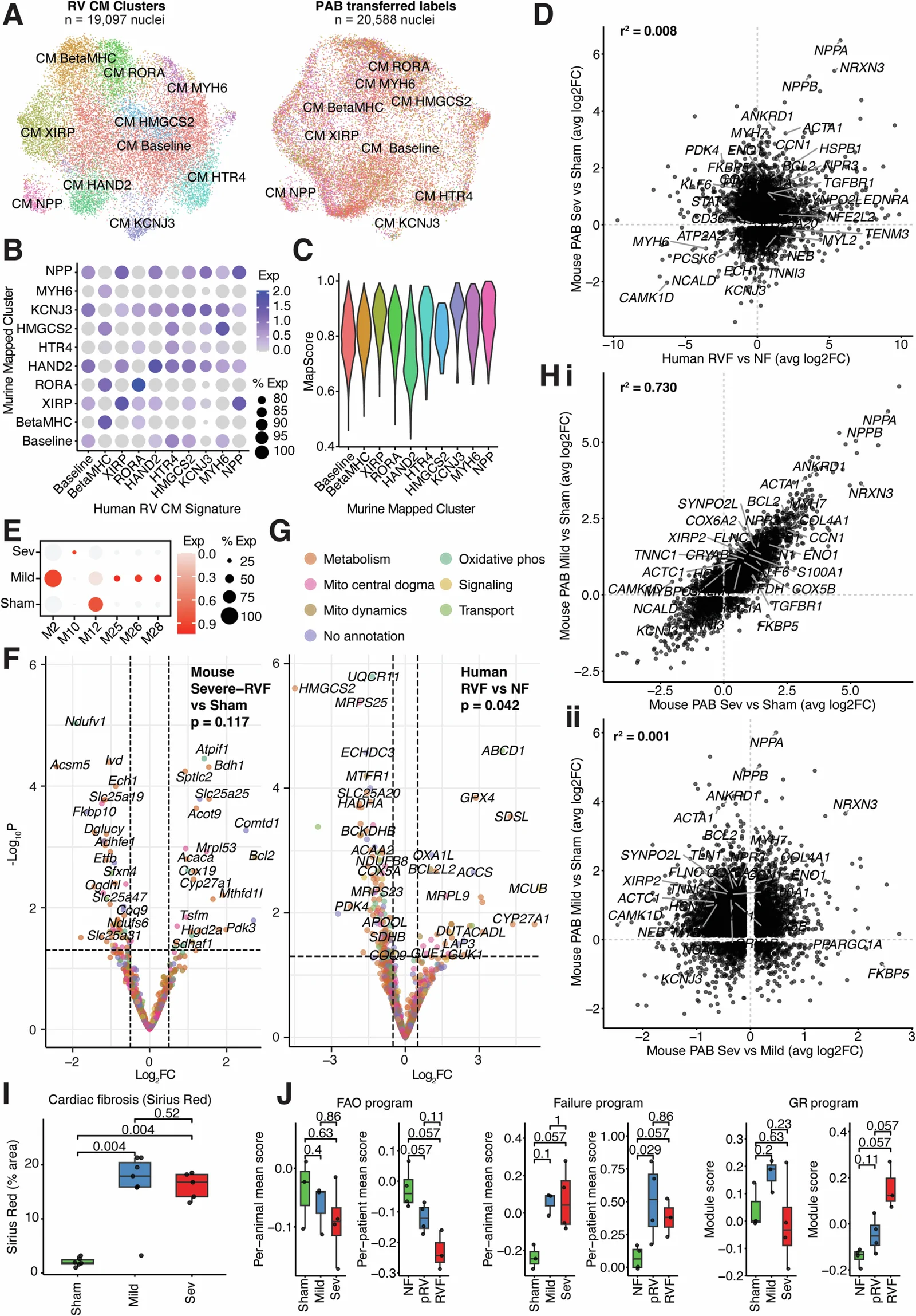
Mouse PAB cardiomyocyte comparison with human RV failure. (**a**) Reference mapping of mouse PAB cardiomyocytes to human RV cardiomyocytes shows partial confirmation of cell sub-types. (**b**) Seurat-calculated expression score of human RV cardiomyocyte sub-cluster markers in the corresponding mouse PAB cardiomyocyte sub-cluster. (**c**) Seurat-calculated mapping score for each individual sub-cluster into the mouse PAB dataset. (**d**) Scatter plot of log2 fold changes for the human RV cardiomyocyte RVF vs NF vs mouse PAB RV cardiomyocyte severe RVF vs sham comparison by gene. Genes with log2 fold changes < 0.1 are not shown. (**e**) Dot plot of normalized Seurat-calculated expression score of corresponding WGCNA module genes in the mouse PAB cardiomyocytes by disease state. (**f**) Per-mouse pseudobulk DESeq2 volcano of MitoCarta3.0 genes in mouse RV CMs, severe-RVF vs sham (n = 4 vs 3) with apeglm log2 fold-change shrinkage. Points colored by MitoCarta3.0 top-level pathway. Per-mouse Wilcoxon p-value is shown for the sham vs (mild-RVF and severe-RVF) comparison. (**g**) Per-patient pseudobulk DESeq2 volcano of MitoCarta3.0 genes in human RV CMs, RVF vs NF (n = 3 vs 4) with apeglm log2 fold-change shrinkage; points coloured by MitoCarta3.0 top-level pathway. Per-subject Wilcoxon p-value is shown for the NF vs (pRV and RVF) comparison. (**h**) Within-mouse fold-change trajectories: (**i**) mouse Severe RVF vs Sham against Mild RVF vs Sham (internal-consistency control sharing the Sham reference; r^2^ = 0.73), and (**ii**) mouse Severe RVF vs Mild RVF against Mild RVF vs Sham, orthogonal trajectories (r^2^ = 0.001) consistent with mild RVF representing a transcriptionally distinct, transient state rather than a linear intermediate to severe RVF. Cardiac/heart-failure-relevant genes ( ≥ 5% CM expression, |log_2_FC | > 1) labelled across all FC scatters. (**i**) Cardiac fibrosis quantified as Sirius Red percent area per animal across PAB stages (Sham n = 6, Mild n = 7, Sev n = 5; Wilcoxon). (**j**) Cross-species concordance of three cardiomyocyte programs (per-subject mean module scores, mouse PAB and human RV shown side-by-side): fatty-acid oxidation (*HMGCS2*, *HADHA*, *HADHB*, *ACADM*, *ACADVL*, *CPT1A/B*, *CPT2*, *ACOX1*, *ECH1*, *ECHS1*, *ETFDH*, *SLC25A20*), failure/fetal program (*NPPA*, *NPPB*, *ANKRD1*, *MYH7*, *XIRP1*, *ACTA1*, *BAG3*), and glucocorticoid receptor (*NR3C1*). The failure program rises in both species while fatty acid oxidation declines, but the glucocorticoid axis shows opposite directionality. Source data

Mouse cardiomyocyte subclusters mapped only partially to human (Extended Data Fig. 6a–d). *NPPA*/*NPPB* rose in both species, but human mitochondrial modules showed inconsistent mouse trends (Extended Data Fig. 6e). Human mitochondrial module score trends were not recapitulated in the mouse at the per-animal level (Extended Data Fig. 6e). However, at the gene level, mouse PAB cardiomyocytes showed coordinated suppression of mitochondrial transcripts (MitoCarta gene set enrichment analysis (GSEA) normalized enrichment score (NES) = −1.8, FDR < 10^−^^17^; Extended Data Fig. 6f), concordant in direction with adult human RVF (Extended Data Fig. 6g) although of smaller magnitude and not significant at the per-animal level. Interestingly, NF → mild-RVF and mild → severe-RVF correlated poorly, suggesting that mild-RVF is a distinct state rather than a linear intermediate (Extended Data Fig. 6h), consistent with differences in severity and chronicity of pressure overload rather than a discrete protective mechanism. PAB induced progressive fibrosis on Sirius Red histology (Extended Data Fig. 6i), and a focused cross-species comparison resolved program-specific concordance: FAO and failure/fetal programs were cross-species concordant while the glucocorticoid axis diverged (Extended Data Fig. 6j).

Echocardiography at 2 weeks and 2 months confirmed progressive pressure overload (reduced tricuspid annular plane systolic excursion (TAPSE), fractional area change (FAC) and systolic decline; Supplementary Table 8). Consistent with a decreased transcriptional level of mitochondrial genes, respirometry (Fig. 5i and Supplementary Data 15) showed reduced respiratory capacity at both timepoints: at 2 weeks, the deficit was concentrated in severely affected mice (severe-RVF, approximately 35% reduction in coupled complex I + complex II + FAO respiration versus sham); by 2 months, it penetrated mild-RVF and intensified in severe-RVF (approximately 50−60% reduction). Mitochondrial respiratory dysfunction is, thus, conserved between adult human and PAB hearts.

In summary, comparing human adult RVF to mouse PAB-induced RVF, we found:

- Conservation of cardiac cell types, with the notable absence of adipose cells in mice
- Concordant expansion of ECs with RVF, largely arterial in mice versus capillary in humans
- Discordance in MHC-II downregulation in mouse myeloid cells versus induction in human RVF
- Concordant cardiomyocyte mitochondrial transcript suppression in both species (significant by gene set enrichment but more modest in mouse than the clear suppression in human RVF)
- Markedly reduced mitochondrial respiratory capacity, with the functional deficit exceeding the transcriptional change

### Pediatric RVF shows similar transcriptional responses as adult RVF

**Fig. 8:**
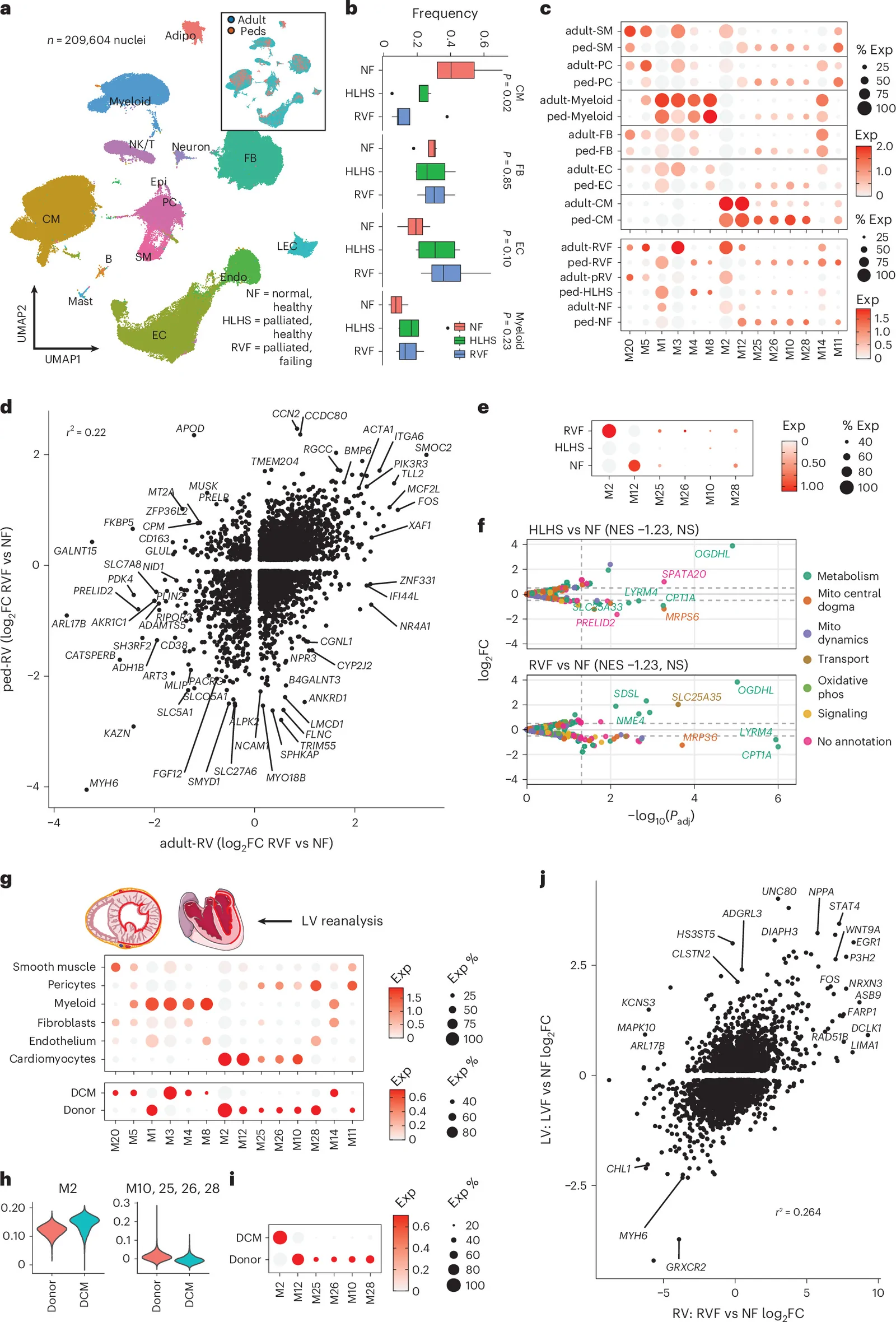
Cross-species, cross-age and cross-etiology comparisons. **a**, UMAP showing co-embedding of HLHS (*n* = 148,206 nuclei) and adult RV (*n* = 61,398 nuclei) datasets. Note the presence of shared cell types. Inset UMAP is colored by nuclei origin. **b**, Cell type abundance by disease state for HLHS dataset. Statistical comparison was done via one-way ANOVA (ped-NF *n* = 4; ped-HLHS *n* = 5; ped-RVF *n* = 5). Box plots are defined as in Fig. 2c. ANOVA is a two-sided *F*-test computed independently per cell type, unadjusted across cell types. **c**, Bulk WGCNA module expression by cell type (top) and disease state (bottom) for HLHS and adult RV dataset. **d**, Scatter plot showing pseudobulked log_2_FCs between adult-RVF versus adult-NF RV and HLHS ped-RVF versus ped-NF samples. Genes not present in both datasets were removed. Low-abundance genes (expressed in <5% of nuclei in both datasets) were also removed. **e**, Cardiomyocyte-specific module expression by disease state in HLHS dataset. **f**, MitoCarta3.0 gene-level volcano plots for pediatric cardiomyocytes: ped-HLHS (top) and ped-RVF (bottom) versus ped-NF. Per-patient pseudobulk DESeq2 (apeglm log_2_FC shrinkage, FDR from the unshrunken Wald test; *n* = 4 ped-NF, *n* = 5 ped-HLHS, *n* = 5 ped-RVF). Each point is a MitoCarta3.0 gene colored by top-level MitoPathway category. Gene set enrichment over the full ranking: ped-HLHS NES = −1.23, ped-RVF NES = −1.23. The shift is not significant at the patient level. Gene set enrichment used fgsea (10,000 permutations) against the MitoCarta3.0 nuclear-encoded set. **g**, Seurat-computed WGCNA module scores by cell type (top) and disease state (bottom). Note the general conservation of module cell type specificity and disease state specificity between RV and LV. **h**, Per-nucleus violin plots of Seurat-computed WGCNA module scores for M2 expression (left) and pooled M10, M25, M26 and M28 expression (right) for NF versus DCM LV cardiomyocytes. **i**, Dot plot of Seurat-computed WGCNA module scores over exclusively LV cardiomyocytes split by disease state. Just as in the RV, M2 increased with failing hearts while M10, M25, M26 and M28 decreased. **j**, Scatter plot of log_2_FCs for the LV (DCM versus NF) versus RV (RVF versus NF) comparison by gene. FCs are well correlated between ventricles except for notable labeled outliers. B, B cell; Exp, normalized *z*-score of module score; Exp %, percent of nuclei expressing given module; phos, phosphorylation. Source data

We also profiled RVs from patients with HLHS after single-ventricle palliation (*n* = 5 Fontan without RVF; *n* = 4 Fontan with RVF; *n* = 1 Glenn with RVF; palliated without RVF called ped-HLHS and with RVF called ped-RVF) plus pediatric controls (*n* = 4; ped-NF). Details of this pediatric dataset can be found in Lavine et al.^50^. Single-nucleus RNA-seq recovered similar cell populations as in adults, with concordant EC expansion and cardiomyocyte contraction in ped-HLHS/RVF (Fig. 8a,b and Supplementary Data 21). Cross-age WGCNA modules and global RVF transcriptional changes correlated well (Fig. 8c,d and Supplementary Data 22). At the mitochondrial level, however, the pediatric RV diverged from the adult and PAB cohorts: cardiomyocyte mitochondrial modules were not clearly induced (Fig. 8e), and mitochondrial transcripts showed only modest, non‑significant downregulation (Fig. 8f), directionally consistent with, but far milder than, the coordinated nuclear‑encoded mitochondrial gene loss of adult RVF. Concordantly, within‑pediatric respirometry (ped‑NF versus ped‑RVF; Fig. 5h) revealed no significant deficit in ETC complex activity (complex I −9.7%, complex I + II −2.3%, complex II +4.1%, complex IV +42.4%; all Mann−Whitney not significant), unlike the marked complex‑level deficits of adult RVF and PAB. Notably, preserved maximal ETC capacity does not exclude the substrate‑level bioenergetic impairment, reduced carnitine palmitoyltransferase (CPT) activity and altered TCA cycle flux, reported in explanted failing single‑ventricle hearts by Garcia et al.^51^, who also found that NF single‑ventricle hearts already adopt an intermediate metabolic phenotype. Our data, therefore, indicate that the severe, coordinated ETC transcriptional and functional collapse characteristic of adult RVF is attenuated in pediatric HLHS‑palliated RVs rather than implying globally normal pediatric mitochondrial metabolism. Given the small, palliation‑heterogeneous cohort, these comparisons are underpowered and should be read as a relative, not absolute, divergence from adult RVF.

**Extended Data Fig. 7:**
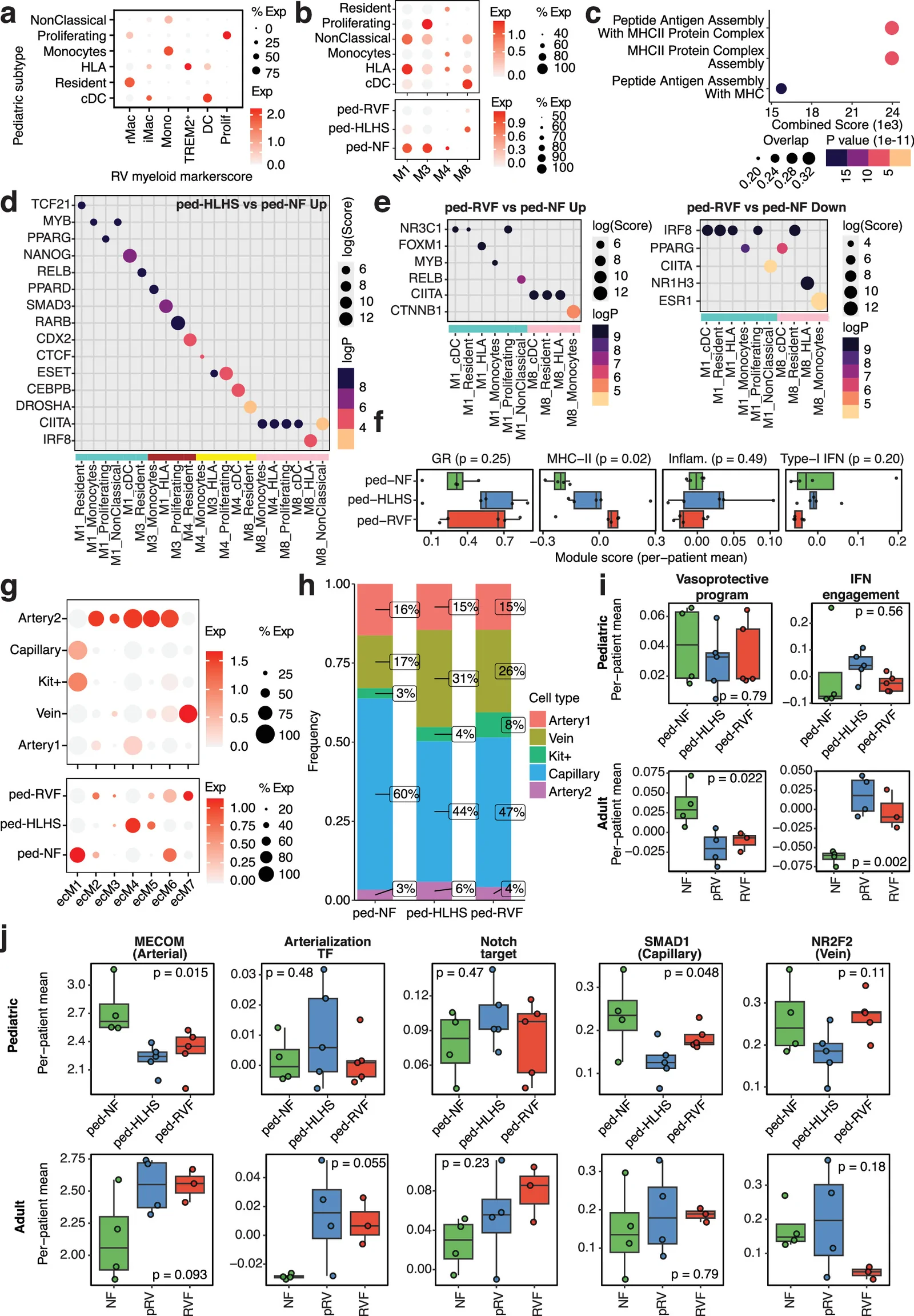
Pediatric snRNA-seq myeloid and endothelial comparison with adult RV. (**a**) Dotplot of normalized Seurat-calculated expression score of cell type marker genes from adult RV dataset for annotated myeloid cell types in HLHS dataset. (**b**) Dotplot of normalized Seurat-calculated expression score of bulk WGCNA modules by cell type and disease state in myeloid subclustering of the HLHS dataset. (**c**) Gene ontology biological process enrichment for upregulated M8 DEGs for failing single-ventricle vs NF bi-ventricle samples. (**d**) ChEA enriched transcription factors for upregulated DEGs for NF single-ventricle samples vs NF bi-ventricle samples. (**e**) ChEA enriched transcription factors for upregulated DEGs for failing single-ventricle samples vs NF bi-ventricle samples (left) and for downregulated DEGs (right), subsetting on exclusively M1 and M8 genes. (**f**) Per-patient myeloid program scores (glucocorticoid transactivation, MHC-II/antigen presentation, inflammasome, type-I interferon signalling) across ped-NF/ped-HLHS/ped-RVF (n = 4/5/5; Jonckheere-Terpstra test p value shown). (**g**) Dotplot of normalized Seurat-calculated expression scores for adult RV endothelial hdWGCNA modules in endothelial subclustering of the HLHS dataset by cell type (top) and disease state (bottom). Similar to the adult RV dataset, M1 is primarily capillary-expressed, M4 is primarily arterial-expressed, and M7 is primarily venous-expressed. (**h**) Stacked barplot showing EC type proportion in HLHS dataset by disease state. Note prominent venous expansion. (**i**) Phase 1 EC vasoprotective program score (pooled antioxidant, RAAS/vasoreactive, anti-inflammatory, ECM-stabilization, EC-quiescence categories) and IFN engagement score, per-patient pseudobulk; pediatric (top) and adult (bottom) ECs; one-way ANOVA per cohort. (**j**) Subtype-restricted Phase-2 readouts in pediatric (top) vs adult (bottom) ECs: *MECOM* in arterial EC, arterialization-TF score (pan-EC), Notch-target score (pan-EC), *SMAD1* in capillary EC, *NR2F2* in venous EC; per-patient pseudobulk means; one-way ANOVA per facet. Adult *MECOM* induction contrasts with pediatric *MECOM* reduction, paralleling adult capillary/arterial expansion vs pediatric venous expansion. Source data

Myeloid lineages were largely concordant: M1/M3/M4 enriched in ped-NF; M8 enriched in ped-RVF; and *MHC*-*II* induction paralleled adults (Extended Data Fig. 7a–e); *IRF8* targets were additionally depressed across CCR2^−^ resident macrophage, human leukocyte antigen (HLA) and conventional dendritic cell (cDC) clusters (underpowered for FDR), with concordant loss of canonical *IRF8*-maintained genes (Extended Data Fig. 7e). Of four adult-derived myeloid programs, MHC-II induction and lack of increase in inflammasome or type I IFN signaling recapitulated, but we did not detect a downregulation of GR transactivation (Extended Data Fig. 7f). The dominant cross-group difference for every program was the ped-NF → ped-HLHS jump rather than ped-HLHS → ped-RVF, indicating that pressure-loaded, systemic single-RV physiology itself (not failure onset) drives most of the myeloid program shift. EC lineages diverged substantially: ped-RVF EC expansion was venous dominated; *MECOM* was reduced (versus induced in adults); and arterialization^52^, Notch, *SMAD1* and *NR2F2* all differed (Extended Data Fig. 7g–j).

**Extended Data Fig. 8:**
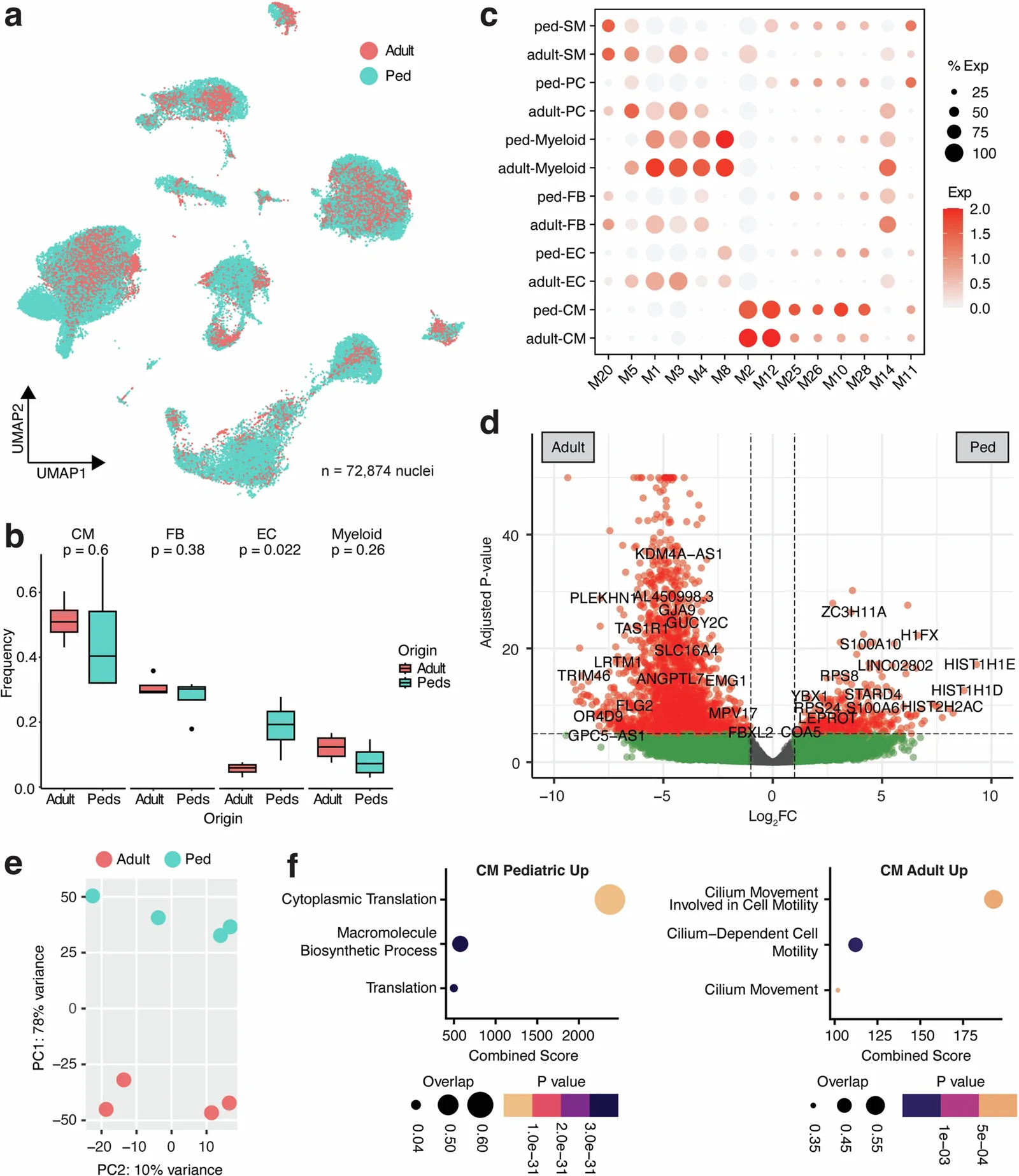
Adult versus pediatric non-failing RV comparison. (**a**) UMAP showing co-embedding of healthy pediatric and adult RV datasets. (**b**) Cell type abundance by dataset origin. Statistical comparison done via one-way ANOVA. (**c**) Bulk WGCNA module expression by cell type and dataset origin. Volcano plot (**d**), PCA embedding (**e**), and gene ontology enrichments (**f**) of cardiomyocyte DEGs for pseudobulk RNAseq data from healthy donor adult versus pediatric cardiomyocytes. Source data

To anchor the cross-age comparison developmentally, we directly compared healthy adult versus healthy pediatric NF RVs (Extended Data Fig. 8). Cell types co-embedded with conserved assignments (Extended Data Fig. 8a) but showed age-dependent abundance shifts (Extended Data Fig. 8b) and lineage-specific differences in bulk WGCNA module expression (Extended Data Fig. 8c). Cardiomyocyte pseudobulk DESeq2 (Extended Data Fig. 8d) and PCA (Extended Data Fig. 8e) confirmed substantial transcriptional divergence by age. Gene Ontology enrichment of cardiomyocyte DEGs comparing pediatric to adult cells (Extended Data Fig. 8f) identified translational machinery in pediatric cardiomyocytes versus potassium channel and cadherin-mediated junctional programs in adult cardiomyocytes. This developmental baseline frames the cross-age disease comparisons above: a portion of apparent pediatric-versus-adult RVF differences may reflect developmental setpoint divergence rather than disease-specific adaptation.

In summary, comparing pediatric to adult RVF, we found that:

- Gene expression modules are largely concordant between adult and pediatric RVs.
- Overall, there is substantial, but not perfect, concordance in the transcriptional changes during RVF.
- Endothelial expansion is seen in both cases of RVF, although it appears driven by venous ECs in pediatric hearts versus capillary ECs in adult hearts.
- Mitochondrial gene expression is mostly unchanged in pediatric RVF, unlike adult RVF. Consistent with this, mitochondrial respiration also appears unchanged.
- Myeloid MHC-II expression is induced in both cases.

### RVF and LVF share core transcriptional programs

Cell-type-resolved LV concordances have already emerged from the fibroblast (Extended Data Fig. 4a–c) and myeloid (Extended Data Fig. 4d–i) analyses above. To consolidate that picture and extend it to the bulk module and transcriptome scale, we leveraged the same Koenig et al.^10^ human LV snRNA-seq dataset across the full RV WGCNA module set, which showed that RV modules were broadly conserved in LV with concordant NF-versus-DCM trends (Fig. 8g). Findings that were common to RVF and LVF included induction of the contractile/cytoskeletal/focal adhesion module M2 (Fig. 8h); repression of the nuclear-encoded ETC mitochondrial modules M10, M25, M26 and M28 (Fig. 8i); and NF-versus-failing pseudobulk cardiomyocyte FCs (Fig. 8j). One chamber-specific divergence emerged from the myeloid comparison: the RV iMac population uniquely retained TLF residency markers that were not reported in the Koenig et al. LV inflammatory macrophages. This is consistent with, although it does not prove, an RV iMac hybrid resident-like state.

Thus, the core heart failure programs (mitochondrial suppression, inflammatory activation and myofibroblast activation) are shared between RVF and LVF.

## Discussion

We integrated bulk, single-nucleus and spatial transcriptomics with orthogonal mitochondrial respirometry, flow cytometry and histology to construct a multi-lineage atlas of human RVF, from which a two-phase model emerges. Phase 1 (NF → pRV) carries the dominant transcriptional burden in bulk and snRNA-seq pseudobulk and features tissue-wide erosion of protective programs, resident macrophage identity loss, fibroblast activation, endothelial expansion and anti-oxidant suppression and pan-tissue *FKBP5* loss. The compensated-to-decompensated (pRV → RVF) transition is best framed as a transcriptional set-point shift in phase 1 (approximately 95% of platform-resolved DEGs) followed by qualitatively distinct, lineage-specific phase 2 programs that include cardiomyocyte oxidative stress, EC *ANGPT2*/*ADAMTS1* induction consistent with vascular remodeling and fibroblast matricellular activation.

A central and unanticipated finding is that the pediatric single-ventricle RV resists the mitochondrial failure that characterizes adult and mouse pressure overload. In adult RVF, a coordinated decline in nuclear-encoded mitochondrial transcripts, accompanied by suppression of the ERRα/PGC-1α biogenesis program, parallel impaired complex I + II respiratory function. The mouse PAB RV showed the same concordant decline: cardiomyocyte mitochondrial transcripts were coordinately downregulated (gene set enrichment, consistent with the decreased ETC gene expression reported by Hwang et al.^53^) alongside reduced respiratory capacity, although the per-animal transcriptional effect was modest relative to the functional deficit. By contrast, pediatric HLHS-palliated failing RVs were metabolically spared. Mitochondrial transcripts were neither induced nor significantly suppressed, and respiratory capacity was preserved (Fig. 5h). The cross-cohort respiratory pattern (Fig. 5j), with deficits in adult and mouse but not pediatric single-ventricle RVF, together with the transcriptional data, suggests that the adult and mouse pressure-overloaded RVs undergo coupled mitochondrial transcriptional and functional decline. Conversely, the developing single-ventricle heart sustains respiratory function.

A notable feature of the RV myeloid compartment was a macrophage population marked by an immediate-early gene program, including *NR4A1*, *NR4A3*, *DUSP2*, *FOS* and *JUN*, which we designate as iMac. Because induction of these transcription factors is a recognized consequence of enzymatic tissue dissociation in single-cell RNA-seq, such signatures are often dismissed as technical artifact^54^. Several lines of evidence argue against that interpretation here. The program was defined in snRNA-seq, which omits the warm enzymatic dissociation step responsible for the artifact, and was independently recovered by in situ spatial transcriptomics (Xenium), in which transcripts are detected on fixed tissue without any dissociation. Furthermore, the signature was confined to a single macrophage subset and showed reproducible spatial organization, co-localizing with monocytes within discrete tissue foci—a distribution incompatible with a uniform, handling-induced stress response, which would be expected across all cell types irrespective of identity. The iMacs additionally retained resident macrophage markers that were not observed in the inflammatory macrophages of the Koenig et al.^10^ left ventricular atlas, suggesting that the RV harbors an activated, resident-like macrophage state localized to sites of monocyte accrual. Whether this state reflects local activation of resident macrophages or in situ adaptation of recruited cells cannot be resolved from cross-sectional human data and will require lineage-resolved models. Regardless of origin, its presence points to a chamber-specific organization of the myeloid response in right ventricular failure.

We found evidence for disruption in GR signaling, with tissue-wide loss of the GR co-chaperone *FKBP5* and a coordinated, pan-myeloid suppression of *NR3C1* transactivation targets, although this evidence is limited to transcriptional state. Because the GR can bind both positive and negative glucocorticoid response elements (GREs) and drive either induction or repression accordingly, downregulation of GRE-associated transcripts does not by itself imply reduced GR activity. Therefore, the direction of GR signaling cannot be inferred from target gene expression alone, and functional validation of altered glucocorticoid signaling will require dedicated studies. However, the PAB model only partially reproduces this human signature (*NR3C1* targets unchanged), limiting its utility for studying RV glucocorticoid biology. Because *FKBP5* loss is observed across both tissue-resident and circulating-origin lineages, the underlying signal could reflect either local microenvironmental modulation of glucocorticoid responsiveness or systemic axis perturbation. Distinguishing these possibilities will require dedicated experimental designs.

The endothelial expansion seen in pRV/RVF across adult human, pediatric human and mice is somewhat unusual among heart failure transcriptomic studies but has been reported in the context of ischemic left ventricular cardiomyopathy^11^. We hypothesize that this reflects coronary microvascular adaptation to RV-specific hemodynamics (a thin-walled chamber perfused throughout systole as well as diastole), driving compensatory capillary/arterial expansion to maintain oxygen delivery in the setting of pressure loading, with adult *MECOM*^+^ arterial induction and pediatric venous-dominated expansion reflecting different developmental contexts. Phase 2 angiopoietin axis destabilization (*ANGPT2*, *MYC*, *ADAMTS1*) suggests that this initially compensatory vascular response transitions toward destabilization at decompensation. Functional validation will require perfusion imaging, vascular casting or interventional EC studies.

More broadly, mouse PAB reproduces the hemodynamic burden of human RVF and converges with human RVF in terms of metabolic and structural programs: suppression of nuclear-encoded mitochondrial genes, reduced respiratory capacity, cardiomyocyte fetal gene reactivation, endothelial expansion and progressive fibrosis. However, it diverges in immune and hormonal programs, most notably myeloid antigen presentation (MHC-II/*CIITA*) and glucocorticoid signaling and in finer features such as the arterial-biased endothelial expansion seen in mice. The model is, therefore, well suited to studying RV metabolic and fibrotic remodeling but should be applied cautiously to immune or glucocorticoid pathways.

Our atlas can be placed directly against previous cross-chamber and cross-etiology studies. In the NF human heart, the right and left ventricles are nearly transcriptionally identical. The four-chamber atlas of Tucker et al.^16^ reported only approximately 24 genes differentially expressed between the ventricles, versus more than 1,000 for each atrial−ventricular contrast, implying that the RV-specific programs we resolve in failure are disease emergent rather than reflections of baseline chamber identity. Against the matched failing-LV/DCM atlas of Koenig et al.^10^, the failing RV and LV share a substantial core transcriptional program (mitochondrial suppression, cytoskeletal remodeling and immune activation)^10,12,15^ with RV-specific divergence in focal programs such as glucocorticoid axis loss and reduced inflammasome induction. A rat pulmonary-artery-banding (RV) versus aortic-banding (LV) model likewise shows broadly overlapping ventricular transcriptomes at baseline (approximately 91% shared) but a distinct, stronger RV disease response, together with a conserved secreted/matrix right-heart-failure signature of approximately 50 genes spanning rat PAB and human chronic thromboembolic pulmonary hypertension (CTEPH)^55^. Notably, isolated afterload modeling argues that RVF and LVF are largely distinct: only 2.2% of human LVF genes overlap the rat PAB regulated set^55^. Our predominantly LVF secondary cohort is the informative exception: its RVF shares the broad cardiomyopathic core of the failing LV, which we attribute to the common myocardial and systemic substrate of LVF-associated RVF rather than to the isolated afterload that drives the RV-versus-LV divergence seen in the banding model.

These comparisons carry translational implications. The shared core molecular pathology between LVF-associated RVF and LVF suggests that the clinical failure of LV-directed guideline-directed medical therapy (GDMT) in isolated RVF^9^ reflects engagement of non-cardiac targets, biomechanical context, chamber geometry or upstream hemodynamic triggers rather than fundamentally divergent molecular pathways. Combined with a parallel plasma LTBP-2 biomarker study^56^, the fibrosis-related RV transcripts identified here may eventually serve as biomarkers of RV remodeling. Overall, the multi-lineage microenvironmental remodeling that we identify nominates anti-fibrotic strategies (notably, TGFBR3), immunomodulatory approaches and direct mitochondrial functional support as plausible RV-specific therapeutic avenues.

Our study has limitations. The snRNA-seq cohort (*n* = 11), although among the largest for human RV, remains modest. We address this through the larger bulk cohort (*n* = 142) and cross-platform concordance for key claims. Xenium was available for *n* = 3 per group, limiting CellChat inference, and is restricted to the 477-gene panel, so off-panel claims (notably, *POSTN*, *FKBP5*, *TGFBR3* and *MLIP*) rest on bulk and snRNA-seq alone. Also, the phase 2 signal is quantitatively limited across platforms. Endothelial EndoMT and cardiomyocyte reactivation programs will require targeted functional validation.

## Methods

### Ethical compliance

This study complies with all relevant ethical regulations. Studies involving human tissue were approved by the University of Pennsylvania Institutional Review Board (study number 802781), the Childrenʼs Hospital of Philadelphia Institutional Review Board (study number 22-020464) and the Gift-of-Life Donor Program. All animal studies were approved by the University of Pennsylvania Institutional Animal Care and Use Committee (protocols 805255 and 805309).

#### Experimental model and subject details

##### Mouse models

All animal studies were approved by the University of Pennsylvania Institutional Animal Care and Use Committee (protocols 805255 and 805309). Mice were housed in the University of Pennsylvania vivarium on a 12-hour light/dark cycle (lights on 07:00, off 19:00), at an ambient temperature of 20−23 °C and 30−70% relative humidity, with constant access to normal chow and water. Experiments were initiated on mice 6−10 weeks (typically 7−8 weeks) of age, unless otherwise stated. All experiments were performed with littermate controls. Only male mice were used in order to remove sex as a biological variable. PAB and sham surgery were performed on 9-week-old male C57BL/6J mice (The Jackson Laboratory, 000664); resident macrophage bulk RNA-seq used 8-week-old male heterozygous *Cx3cr1*-CreER;*Rosa26*-tdTomato mice. Per-experiment group sizes are given in Supplementary Tables 7 and 8. The mouse studies described here involved heterozygous *Cx3cr1*-CreER;*Rosa26*-tdTomato mice, which were bred from *Cx3cr1*-CreER (The Jackson Laboratory, 020940) and *Rosa26*-tdTomato (The Jackson Laboratory, 007914) mice.

#### Method details

##### Statement on human specimens

This study complies with all relevant ethical regulations and was approved by the University of Pennsylvania Institutional Review Board (study number 802781), the Childrenʼs Hospital of Philadelphia Institutional Review Board (study number 22-020464) and the Gift-of-Life Donor Program. Prospective informed and written consent for research use of surgically explanted tissue was obtained from the transplant recipients or from the next-of-kin in the case of deceased organ donors. The consent obtained covers publication of the resulting data: participants, or next-of-kin for deceased organ donors, were informed that the results of the research may be presented at scientific meetings or in publications, with identity not disclosed and only coded identifiers used. No compensation was provided for participation^57^. All sequencing data were deidentified prior to deposition. Heart tissue was procured according to a previously described standard protocol^57^. In brief, all hearts underwent in situ cardioplegia and were kept in cold cardioplegia or saline until transmural samples were snap frozen in liquid nitrogen (within 4 hours) and stored at −80 °C until processing for mRNA analysis.

### Participant demographics

Bulk RNA-seq included 142 participants: NF *n* = 29 (11 male, 18 female; mean age 51.8 ± 16.1 years, range 18−75), pressure-loaded *n* = 78 (61 male, 17 female; mean age 53.6 ± 11.0 years, range 20−69) and RV failure *n* = 35 (21 male, 14 female; mean age 55.0 ± 8.7 years, range 27−69). Single-nucleus RNA-seq included 11 participants: NF *n* = 4 (two male, two female; mean age 55.5 ± 12.1 years, range 39−65), pressure-loaded *n* = 4 (two male, two female; mean age 54.2 ± 1.3 years, range 53−56) and RV failure *n* = 3 (two male, one female; mean age 47.7 ± 20.0 years, range 27−67). Xenium spatial transcriptomics included nine participants: NF *n* = 3 (one male, two female; mean age 61.0 ± 6.1 years, range 54−65), pressure-loaded *n* = 3 (two male, one female; mean age 54.3 ± 1.5 years, range 53−56) and RV failure *n* = 3 (two male, one female; mean age 47.7 ± 20.0 years, range 27−67). Per-participant sex, age and underlying diagnosis are given in Supplementary Tables 1, 4 and 6. The pediatric single-nucleus cohort comprised 14 participants from two institutions (Colorado *n* = 9, Washington University in St. Louis *n* = 5): NF donors *n* = 4 (two male, two female; mean age 4.9 ± 3.3 years, range 1.3−8.0), single-ventricle participants with protein-losing enteropathy or plastic bronchitis *n* = 5 (three male, two female; mean age 9.6 ± 5.1 years, range 4.8−16.0) and single-ventricle participants with systolic heart failure *n* = 5 (four male, one female; mean age 10.5 ± 8.3 years, range 3.8−23.0). One participant in the systolic heart failure group was 23 years old at collection. All others were under 18.

### Bulk RNA-seq: sample stratification

Diseased RVs were stratified into two groups: pRV (right atrial pressure and right atrial to pulmonary capillary wedge pressure ratio—that is, RA ≤ 8 mmHg, RA:PCWP ≤ 0.37) and RVF (RA ≥ 9 mmHg, RA:PCWP ≥ 0.63) based on validated clinical predictors of RVF or death after left ventricular assist device placement^17,18^.

### Bulk RNA-seq: human sample preparation

Snap-frozen cardiac tissues from the RVs of explanted donors were pulverized in a liquid nitrogen-cooled cryomill. Total RNA extraction was performed using RNeasy Mini Kit (Qiagen) with poly(A) selection. RNA quality was assessed using RNA 6000 Nano Kit (Agilent Technologies) and 2100 Bioanalyzer (Agilent Technologies), targeting an RNA integrity number (RIN) score >7.0. RNA-seq libraries were prepared using TruSeq mRNA Stranded Kit (Illumina) according to the manufacturerʼs recommended protocol.

### Bulk RNA-seq: transcriptome pseudo-alignment

Libraries were sequenced to a depth of more than 20 million reads per sample. Reads were trimmed using Trimmomatic^58^. Trimmed reads were pseudo-aligned to the human transcriptome (GRCh38) using Kallisto^59^. Transcript-level abundances were converted to gene-level counts with tximport, which weights transcripts by length to correct for the length dependence of read counts under the 2 × 150 sequencing configuration^60^. The resultant gene-level count matrices were then used for downstream analysis.

### Bulk RNA-seq: differential gene expression

DEGs were detected using DESeq2 (ref. ^61^). To account for inter-subject differences including sex, race, other clinical variables and batch effects, SVA^62^ was used to estimate 21 surrogate variables and remove sequencing batch effects—that is, with the model Expression ~ Group + SV1 +…+ SV21, where the tilde represents ‘distributed as’. A joint model was fit over all contrasts, and then pairwise comparisons (for example, NF versus pRV) were conducted for disease states using the Wald test at an *α* value of 0.1. The surrogate variables were fit from demographic and clinical covariates (sex, age, race, body surface area, height, weight, body mass index, thyroid disease, pacemaker status and sequencing and library preparation batch). Those covariates are not released, as they are indirect identifiers. The fitted surrogate variables are deposited with the count matrix so that the published models can be reproduced without patient-level characteristics.

### Bulk RNA-seq: WGCNA

The R packages WGCNA^20^ and hdWGCNA^45^ were used for all WGCNA analysis. Only genes expressed in more than 5% of subjects (or nuclei for EC snRNA-seq) were retained for analysis. For bulk analysis, WGCNA was run on DESeq2 normalized counts and was used to identify gene modules that co-varied among subjects, agnostic of disease state.

The key of EC hdWGCNA module identities is as follows: ecM1 core/capillary identity (*n* = 1,139 genes); ecM2 sarcomeric/cardiomyocyte ambient signature (*n* = 836); ecM3 constitutive RNA splicing/processing (*n* = 647); ecM4 arterial identity (*n* = 125); ecM5 AP-1/NR4A immediate-early stress (*n* = 120); ecM6 peri-vascular/mural-like ECM (*n* = 115); ecM7 venous/inflamed program (*n* = 91). Hubs are in Supplementary Data 18.

For the bulk WGCNA module catalog (nomenclature), the module identities used were as follows (29 in total; full hub gene lists in Supplementary Data 4): M1 resident-macrophage identity (hubs *VSIG4*, *CD163*, *MRC1*, *LAPTM5*, *HCLS1*, *FCN3*); M2 cytoskeletal/focal adhesion + structural/ECM (contractile, multi-lineage); M3 late chromatin/epitranscriptomic (*METTL3*, *INO80*, *KANSL1*, *PHF8*, *MBD1*); M4 myeloid/T cell antigen presentation; M7 polycomb/developmental (*EZH2*, *HOXB8*, *ITGA4*, *POLE2*); M8 MHC-II master, *CIITA*-enriched; M10/M25/M26 nuclear-encoded ETC/OXPHOS (M10/M26 also complex III/ATP synthase/*MCUR1*); M12 cardiomyocyte (*TNNI3*, *TNNI3K*, *BMPR2*, *MYH7*); M13 immunoglobulin repertoire (*IGHG/IGKC*); M14 cardiac transcriptional regulators (*CASZ1*, *RARA*, *KDM5A*, *MED13L*, *TLE4*, *DDR1*); M15 endothelial activation (95 genes, chemokine signaling); M21 TGFβ/Notch/Wnt suppressors (*SKI*, *AXIN1*, *MIB2*, *CTBP1*, *MTA1*, *ALKBH5*); M24 complex I/IV hubs; M27 splicing/RNA processing (*SRSF7*, *SF3B1*); and M28 mtDNA-encoded ETC (*MT-ATP6*, *MT-CO2*, *MT-CO3*, *MT-CYB*, *SOD1*). Modules M5, M6 and M16 are referenced descriptively without standalone identities.

### Single-nucleus RNA-seq: sample selection

Diseased hearts were selected from patients with DCM who had no high-impact variants identified on whole-exome sequencing. To maximize the probability of capturing transcriptional changes, the RVF samples were enriched for severity, possessing decreased diastolic function (RA ≥ 14 mmHg, RA:PCWP ≥ 0.7), decreased systolic function (TAPSE ≤ 1.7) and at least moderate tricuspid regurgitation; pRV samples were comparatively enriched for normal diastolic function (RA ≤ 4 mmHg, RA:PCWP ≤ 0.33), systolic function (TAPSE ≥ 2.2) and trivial or less tricuspid regurgitation. RVF and pRV samples were otherwise well balanced for other clinical and demographic variables (Supplementary Tables 4 and 5). For the pediatric HLHS cohort, Fontan/Glenn single-ventricle physiology differs fundamentally from adult LVF secondary pressure overload in onset timing, ventricular morphology and load history; pediatric comparisons, therefore, test molecular convergence rather than direct replication.

### Single-nucleus RNA-seq: human sample preparation

Samples were prepared as described previously^12^. In brief, frozen cardiac tissues from the RV of explanted donors (approximately 100 mg) were sectioned to 100 µm (Leica, CM1950 cryostat) and then transferred into a 2-ml Dounce homogenizer containing 1 ml of chilled lysis buffer (10 mM Tris-HCl, pH 8.0, 250 mM sucrose, 25 mM KCl, 3 mM MgCl_2_, 1 µM DTT and 0.05% IGEPAL-630 in nuclease-free water). Samples were homogenized with 10 passes of a coarse pestle and then 10 passes of a fine pestle and then incubated on ice for 15 minutes. Large debris was removed by centrifugation at 40*g* for 1 minute (Beckman Coulter, Allegra X-15R swinging bucket centrifuge) before sequentially being filtered through a 50-µm and then a 10-µm filter (puriSelect Life Science) into a 50-ml conical tube. The filters were washed with 6 ml of wash buffer (0.01% BSA, 5 mM MgCl_2_, PBS), and the resultant suspension was pelleted at 550*g* for 5 minutes at 4 °C. The pelleted nuclei were resuspended in 150 µl of cold nuclei resuspension buffer (0.01% BSA, 5 mM MgCl_2_, 0.4 U µl^−1^ murine RNase inhibitor (New England Biolabs), PBS) and counted on a hemocytometer before dilution to 1,000 nuclei per microliter.

### Single-nucleus RNA-seq: mouse sample preparation

Samples were prepared similarly as above for the human samples, with the notable exception of an additional fluorescence-activated cell sorting (FACS) step at the end. DAPI (1 µg ml^−1^) was added into the lysis buffer. Instead of resuspension in nuclei resuspension buffer, nuclei were resuspended in FACS buffer (1% BSA, 0.4 U µl^−1^ murine RNase inhibitor (New England Biolabs), PBS). Nuclei were flowed at the lowest flow rate on a FACSAria III (BD Biosciences) with a 100-µm nozzle. In total, 100,000 DAPI^+^ singlets were collected. Nuclei were recovered by spinning at 500*g* for 5 minutes and then resuspended in nuclei resuspension buffer before quantification and dilution to 1,000 nuclei per microliter.

### Single-nucleus RNA-seq: 10x Chromium single-cell gene expression

Nuclei were loaded onto the 10x Genomics Chromium microfluidic platform (Single Cell 3′ solution, v3) for an estimated recovery of 6,000 cells per device. Processing of libraries was performed according to the manufacturerʼs instructions.

### Single-nucleus RNA-seq: sequencing and alignment

Complementary DNA libraries were multiplexed and sequenced on a NovaSeq 6000 S4 chip (Azenta) to a goal of approximately 75,000−100,000 reads per nucleus. Raw base call files for each sample were demultiplexed and converted to FASTQ files using the 10x Genomics toolkit Cell Ranger 7.1.0 (cellranger mkfastq)^63^. FASTQ files were then uploaded to the 10x Genomic Cloud where Cell Ranger Count 7.1.0 was run on each sample to map them to the human (GRCh38) 2020-A transcriptome or the mouse (GRCm39) 2020-A transcriptome with the include-introns flag set to true. Resultant raw feature matrices were then used for downstream analysis.

### Single-nucleus RNA-seq: ambient RNA and noise removal

To remove ambient and background RNA contamination, we used CellBender^64^, running remove-background with hyperparameter tuning (primarily of learning rate) to ensure smooth convergence during model training. CellBender-denoised count matrices were then further processed with DecontX^65^ to estimate and subtract residual per-cell ambient contamination, and the DecontX-corrected counts were carried forward for all downstream analyses. Some residual cytoplasmic RNA contamination during nuclear isolation can nevertheless produce low-level off-target detection of canonical cell type markers (for example, *RYR2* in non-cardiomyocyte populations); cell type assignment, therefore, relies on relative enrichment over a panel of cluster-defining markers rather than presence/absence of any single gene.

### Single-nucleus RNA-seq: filtering and quality control

All further analysis was conducted in Seurat^66^. Nuclei with more than 5% mitochondrial transcripts, with fewer than 200 transcripts or with more than 100,000 transcripts were discarded. For each nucleus, the entropy (using the Python package ndd and its entropy function) and the exonic percentage (using scR-Invex) were calculated. Nuclei with entropy below Q1 − 1.5×IQR (first quartile (Q1), interquartile range (IQR)), exon percentage above Q3 + 1.5×IQR (third quartile (Q3)) or mitochondrial read percentage above Q3 + 1.5×IQR were flagged as outliers and removed. For secondary outlier detection, nuclei were then clustered using Seuratʼs built-in graph-based clustering approach (FindNeighbors and FindClusters). Each cluster was fit with an ellipsoid using the sklearn EllipticEnvelope function, and the 5% of nuclei farthest from the cluster centroid were labeled as outliers and also removed. For doublet removal, we elected to utilize a semi-supervised approach, as has been done previously^11^, which was based upon identifying nuclei that simultaneously had gene expression signatures consistent with two (or more) cell populations^11^. Practically, this was done by iteratively clustering nuclei at high resolution, generating *z*-score expression profiles for each expected cell population and visualizing the nuclei clusters colored by *z*-score using uniform manifold approximation and projection (UMAP) embedding. Clusters that demonstrated high expression profiles for multiple cell types were deleted, and then the entire above process was repeated until no further clusters were removed (six rounds).

### Single-nucleus RNA-seq: myeloid subtype marker rationale

Myeloid subtype assignments were supported by markers with established functional roles: *F13A1* (coagulation factor XIII) is required for post-infarction cardiac integrity^67^. *MARCO* and *HPGDS* define scavenger populations implicated in non-inflammatory clearance of damage-associated molecular patterns and prostaglandin D_2_-mediated inflammation resolution, respectively^36^. *VSIG4*, *CD163* and *LYVE1* mark self-renewing tissue-resident macrophage subsets associated with complement-mediated immune quiescence^36^ and hyaluronan-dependent maintenance of arterial tone^68^. Initial high-resolution subclustering of CCR2^−^ resident macrophages identified three transcriptionally distinguishable subsets (enriched for *HPGDS*, a broader *F13A1*/*CD209*/*VSIG4* signature or *RBPJ*), but these subsets showed no disease-specific expansion (all differences <10% of total resident macrophages) and were combined into a single CCR2^−^ resident macrophage population for downstream analyses. *CCR2* transcript itself is sparsely detected in droplet-based snRNA-seq of human heart^10^, a well-described dropout effect that disproportionately affects modestly expressed transcripts. Populations were, therefore, defined by combinatorial marker expression rather than by *CCR2* expression alone.

### Single-nucleus RNA-seq: sample integration

The 11 single-nucleus datasets were integrated using the R Harmony package^69^. Specifically, the 11 preprocessed (as above) and normalized/scaled (via SCTransform) datasets were first combined via Seurat merge, and then the RunHarmony command was utilized to generate batch-corrected PCA components (50 dimensions). These Harmony-corrected dimensions (rather than raw PCA) were used for all downstream neighbor graph construction (FindNeighbors), clustering (FindClusters) and UMAP embedding (RunUMAP).

### Single-nucleus RNA-seq: reference mapping

Reference mapping was conducted using the Seurat version 4 reference mapping pipeline. In brief, both the reference and query datasets were initially preprocessed via SCTransform. The reference dataset was then integrated using canonical correlation analysis (CCA) integration rather than Harmony integration as used elsewhere. Anchors between the reference and query dataset were then identified and used to map query data to the integrated reference dataset.

### Single-nucleus RNA-seq: differential gene expression

Differential gene expression at the single-nucleus level was performed using the FindMarkers function. The default Wilcoxon rank-sum method was used for statistical testing. Only genes with log_2_FC > 0.1 were retained, and those with adjusted *P* < 0.05 were considered significant.

### Single-nucleus RNA-seq: pseudobulk differential gene expression

Pseudobulk dataset generation was done by summing counts (from the ‘counts’ slot of the SCTransform assay) for each major cell type using the AggregateExpression function. Differential gene expression analysis was then conducted via DESeq2. For both snRNA-seq and Xenium pseudobulk analyses, multiple-testing correction was performed using Benjamini−Hochberg FDR within each DESeq2 run (per subtype × contrast); no global correction was applied across the full subtype × contrast matrix, in keeping with standard pseudobulk practice where each cell type is treated as a distinct experiment. DEG counts reported throughout this paper use the unified threshold adjusted *P* < 0.05, |log_2_FC| > 0.5 and baseMean > 50 unless otherwise stated.

### Single-nucleus RNA-seq: enrichment analysis

Pathway enrichment analysis was conducted using the web interface for Enrichr^70^ and the R enrichR package. Inputs were genes upregulated or downregulated in the cluster of interest (log_2_FC > 0.1, adjusted *P* < 0.05). Combined score was calculated as *c* = log(*p*) × *z*, where *c* is the combined score, *p* is Fisherʼs exact test *P* value and *z* is *z*-score for deviation from expected rank.

### Bulk RNA-seq: PAH comparison

To compare RVF transcriptional programs across etiologies, we reanalyzed the published PAH RV bulk RNA-seq dataset from Khassafi et al.^19^. Raw count matrices were obtained from the Gene Expression Omnibus (GSE240941) and processed using the same DESeq2 pipeline as our adult RV bulk data. DEGs from PAH RVF versus PAH NF were compared with our RVF versus NF DEGs to identify concordant and discordant genes. Pathway enrichment analysis (EnrichR) was performed separately on concordant genes (same direction in both datasets) and discordant genes (opposite direction).

### Single-nucleus RNA-seq: mitochondrial transcription factor analysis

We assessed mitochondrial biogenesis transcriptional regulators across three analytic axes (per-patient pseudobulk DESeq2 expression, CollecTRI/decoupleR^71^ transcription factor activity inference and MitoCarta3.0 (ref. ^72^) module scoring) to disentangle transcription factor transcript level, downstream regulon activity and aggregate mitochondrial program output.

#### Mitochondrial transcription factor panel

A literature-curated panel of nine mitochondrial biogenesis regulators was analyzed: *ESRRA*, *ESRRG*, *PPARA*, *PPARGC1A* (PGC-1α), *NRF1*, *NFE2L2* (NRF2), *TFAM*, *POLRMT* and *TFB2M*, covering the PGC-1α/ERR/PPAR/*NRF1*/*TFAM* axis governing nuclear-encoded mitochondrial gene expression and mtDNA transcription.

#### Per-patient pseudobulk expression

For each subtype and lineage, raw RNA counts were aggregated per patient × disease state with Seurat AggregateExpression; counts were then modeled by DESeq2 (design = ~group) with apeglm log_2_FC shrinkage and Benjamini−Hochberg correction. Pairwise contrasts (NF versus pRV, pRV versus RVF, NF versus RVF) used the Wald test. Per-patient violin plots show pseudobulk variance-stabilized transformation (VST) expression, with pairwise Wilcoxon rank-sum *P* values reported between disease states. Pseudobulk treats each patient as the unit of replication, avoiding the inflated significance produced by treating individual nuclei as independent observations.

#### Transcription factor activity inference via CollecTRI/decoupleR

Transcription factor activities were inferred from the cardiomyocyte snRNA-seq using the decoupleR R package with the CollecTRI regulon network (decoupleR::get_collectri, organism = ‘human’, split_complexes = FALSE). Per-cell transcription factor activity was estimated by univariate linear modeling (decoupleR::run_ulm) on SCTransform-normalized expression with default minimum regulon size. Per-patient mean activity per disease group was computed and visualized as a heatmap for the curated mitochondrial transcription factor panel; this approach captures downstream regulon-level activation independent of transcription factor transcript abundance. Pairwise group differences were tested by Wilcoxon rank-sum on per-patient mean activities.

#### MitoCarta3.0 module score

The MitoCarta3.0 inventory of human mitochondrial-localized genes was used to score aggregate mitochondrial program output. Seurat AddModuleScore was applied to the full MitoCarta3.0 gene set on the SCTransform-normalized snRNA-seq cardiomyocyte data; per-cell scores were aggregated per cardiomyocyte subcluster × disease state.

#### Bulk RNA-seq cross-validation

DESeq2-VST expression values for the same nine-transcription-factor panel were extracted from the bulk RNA-seq cohort (*n* = 142 patients). Disease-state differences were tested by Wilcoxon rank-sum.

### Spatial transcriptomics: 10x Xenium

The Xenium protocol was completed using the Xenium Slides and Sample Prep Reagents Kit (10x Genomics, PN-1000460) and associated consumables. The procedure itself was done using the manufacturerʼs suggested protocol (CG000578; 10x Genomics) without modifications. In brief, 5-µm sections of formalin-fixed paraffin-embedded (FFPE) RV samples were mounted onto a Xenium slide (10x Genomics) prior to deparaffinization, decrosslinking, fixation and permeabilization. We specifically chose nine RV samples (three NF, three pRV and three RVF) based on RNA quality and sample geometry (variable sizes but typically 10 × 10 mm) for Xenium analysis. Probes were hybridized, washed, ligated and amplified prior to autofluorescence quenching and nuclei staining. Custom probes were designed based on a manually curated list of 100 cluster-specific marker genes from the snRNA-seq data (using the Seurat FindAllMarkers function). The prepared slides were then loaded onto the Xenium Analyzer (10x Genomics), which was run following the recommended protocol. A total of 477 target genes (377 predesigned from the Human Multi-Tissue and Cancer Panel and 100 custom genes) plus 64 negative control probes (541 total) were included on the panel (design ID: 44Z7X8; Supplementary Data 10). Output was visualized via Xenium Explorer (10x Genomics). Default Xenium segmentation recovered fewer cells than transcript-level methods, and Baysor^73^ produced visually apparent over-segmentation of large cardiomyocytes. Proseg^74^ (version 0.3.0) was, therefore, used for resegmentation with the ‘–xenium’ preset, which leverages nucleus boundary overlap for transcript-to-cell assignment. Transcripts with quality value <20 were excluded prior to resegmentation. Each tissue section was divided into overlapping spatial patches using SpatialData/Sopa^75^, and Proseg was run independently on each patch with default parameters (10 components, initial voxel size 10 µm). Patch-level results were merged with global cell ID remapping to avoid collisions across patches. Cell type annotation was performed using RCTD^76^ (spacexr R package) in full mode, using our snRNA-seq dataset as the reference. The snRNA-seq reference was constructed from the 12 major cell type annotations with downsampling to a maximum of 500 nuclei per cell type. RCTD was run with UMI_min = 10 and UMI_min_sigma = 100. RCTD weights were used to assign each spatially resolved cell to its most likely cell type. SPLIT^77^ ambient RNA correction was applied to the Proseg-resegmented count matrices. To validate cell type frequency estimates against segmentation-dependent cell size variation, nucleus-level proportions were computed by mapping Xenium DAPI nucleus boundaries to Proseg cell boundaries using polygon intersection, with each nucleus inheriting the RCTD annotation of its maximally overlapping Proseg cell. This provides an orthogonal frequency estimate where each nucleus counts as one unit regardless of cell body size. SpaGE^21^ imputation was used to impute the expression of genes not included in the Xenium panel but present in the snRNA-seq reference. Subclustering of spatially resolved cells was performed within each of the 12 major cell types using Seurat (version 5). For each cell type, the SPLIT-corrected count matrix (supplemented with SpaGE-imputed genes) was normalized via SCTransform, reduced to 30 principal components and clustered using the Louvain algorithm at a resolution selected to maximize biological interpretability while minimizing over-splitting. Subtype annotations were manually assigned based on marker gene expression and concordance with snRNA-seq subtypes. Pseudobulk differential gene expression analysis was performed within each spatial cell type using DESeq2. To preserve biologically meaningful transcript abundance differences that SPLIT over-corrects, pseudobulk aggregation was performed on the raw, uncorrected Proseg-resegmented count matrix. Genes with |log_2_FC| > 0.5 and adjusted *P* < 0.05 were considered significant. Spatial niche identification was performed using Seurat’s BuildNicheAssay function with *k* = 100 nearest spatial neighbors. To prevent patient-specific cardiomyocyte subtype variation from dominating niche definitions, all cardiomyocyte subtypes were collapsed into a single label prior to niche computation. Centered log ratio (CLR) transformation was applied to niche proportion vectors to amplify differences in rare cell type fractions, followed by *z*-scoring. Mini-batch *k*-means clustering (*k* = 15) was performed on the CLR-transformed vectors. Clusters sharing similar compositions were merged into 14 final niches. Niche proportions were compared across disease groups (NF, pRV, RVF; *n* = 3 per group) using Kruskal−Wallis tests with Benjamini−Hochberg correction.

### Spatial transcriptomics: CellChat cell−cell communication analysis

To infer intercellular signaling within RV myocardium while retaining spatial context, we combined single-nucleus expression with Xenium spatial coordinates. Because the Xenium panel measured only 477 genes, single-nucleus expression was first imputed onto Xenium cells per patient, using each patientʼs own matched snRNA-seq dataset as the reference, so no expression information was shared across individuals. Cell type annotations were consolidated into 13 broad groups from subclustered labels: ECs, cardiomyocytes, fibroblasts, macrophages, pericytes, vascular smooth muscle cells, lymphocytes, myeloid cells (monocytes, dendritic cells and proliferating myeloid cells), adipocytes, epicardial cells, neural cells, lymphatic endothelial cells and mast cells. CellChat^29^ was run separately for each patient. For each sample, the imputed expression matrix, lineage annotations and Xenium cell centroids were used to construct a spatial CellChat object with spatial scale factors derived from the Xenium coordinate system. Ligand−receptor interactions were drawn from CellChatDB.human. Overexpressed genes and interactions were identified, and communication probabilities were computed under spatial constraints using the truncated mean method (trim = 0.25). Interactions supported by fewer than five cells were removed. Pathway-level probabilities were aggregated across constituent ligand−receptor pairs. For aggregate network visualization, per-patient CellChat objects restricted to the myocardium niche were averaged across patients within each condition (NF, *n* = 3; pRV, *n* = 3; RVF, *n* = 3) to prevent dominance by any single individual. Edge weights were normalized by the product of sender and receiver cell type proportions to control for compositional differences across conditions. For pathway ranking, summed communication probability across all ligand−receptor pairs was computed per pathway per condition and normalized to sum to 1 within each condition. Analyses were performed in R with CellChat version 3.

### PAB procedure

All animal studies were approved by the University of Pennsylvania Institutional Animal Care and Use Committee (protocols 805255 and 805309). Mice were housed on a normal light/dark cycle (light on from 7:00 to 19:00) with constant access to normal chow and water. PAB was performed in 9-week-old C57BL/6J mice. The anesthetic procedure included induction with ketamine (100 mg kg^−1^ body weight) and xylazine (10 mg kg^−1^ body weight) cocktail (intraperitoneal injection), tracheal intubation with a 20-gauge needle and ventilation with a Harvard rodent ventilator (Harvard Apparatus) at a rate of 120−180 breaths per minute and tidal volume of 10 µl g^−1^ body weight. The main pulmonary artery trunk was dissected from surrounding tissues and constricted with a 7-0 suture tied against a 25-gauge needle to generate a severe RVF. The same procedure including dissection was performed for sham mice apart from pulmonary artery ligature. Close monitoring was performed during procedure and recovery. Meloxicam was administered preoperatively and postoperatively for 48 hours for analgesia, and mice were evaluated daily for 3 days after surgery. The skin sutures were removed 7−10 days postoperatively to avoid irritation and infection. RV remodeling was confirmed by echocardiography at 2-week and 2-month post-surgery timepoints (Supplementary Table 8).

### PAB resident macrophage bulk RNA-seq

Heterozygous *Cx3cr1*-CreER;*Rosa26*-tdTomato mice were fed tamoxifen chow (Inotiv, TD.130856) starting at 3 weeks of age for 1 week. At 8 weeks of age, mice underwent PAB (or sham surgery). Two weeks after surgery, mice were anesthetized with isoflurane inhalation, and their hearts were perfused with 20 ml of cold PBS. The hearts were then dissected, atria were removed and the left and right ventricles were harvested for single-cell isolation. The ventricles were digested as previously described to generate a single-cell suspension^78,79^. In brief, ventricles were finely minced in ice-cold PBS until a smooth consistency was achieved. Minced tissues were transferred to Eppendorf tubes containing digestion buffer (RPMI supplemented with 450 U ml^−1^ Collagenase I, 60 U ml^−1^ DNase I and 60 U ml^−1^ hyaluronidase enzymes) and digested at 37 °C on a shaker for 45 minutes. After digestion, samples were briefly vortexed and immediately placed on ice to stop the reaction. Then, the cell suspensions were mechanistically dissociated by pipetting with a 1,000-µl pipettor. The digested solution was filtered through a 40-µm cell strainer and rinsed with neutralization buffer (HBSS without Ca^2+^/Mg^2+^, 2% heat-inactivated bovine serum and 0.2% BSA) to obtain a single-cell suspension. Red blood cells were lysed using ACK lysis buffer. Single-cell suspensions were stained for *CD45* (clone 30-F11; BioLegend, 103132), CD11b (clone M1/70; BioLegend, 101223) and *CD64* (clone X54-5/7.1; BioLegend, 139323). Resident macrophages (CD45^+^CD11b^+^CD64^+^tdTomato^+^) were sorted by FACS, pelleted and then sent to Azenta for ultra-low-input RNA-seq.

### Right ventricular cardiomyocyte mitochondrial isolation from frozen tissue

Mitochondria were isolated from flash-frozen tissue as done previously with modifications^80,81^. In brief, frozen right ventricular cardiac muscle was thawed on ice in mitochondrial isolation medium (Buffer A, 50 mM MOPS, 100 mM KCl, 1 mM EGTA, 5 mM MgSO_4_, pH 7.1) for 10 minutes. Thereafter, the cardiac tissue was minced on ice for 3 minutes and then subjected to a 15-minute digestion in Buffer A supplemented with 0.25 mg ml^−1^ Collagenase II (Worthington, LS004176) while rocking at room temperature. After collagenase digestion, cardiac tissue was centrifuged at 300*g* for 5 minutes at 4 °C. The pellet was then resuspended in Buffer B (50 mM MOPS, 100 mM KCl, 1 mM EGTA, 5 mM MgSO_4_, 2 g l^−1^ BSA, pH 7.1) and homogenized via a glass-Teflon homogenizer (Wheaton) for approximately five passes and subsequently centrifuged at 800*g* for 10 minutes at 4 °C. The resulting supernatant containing the mitochondrial fraction was collected in a separate pre-chilled tube and centrifuged at 10,000*g* for 10 minutes at 4 °C to pellet mitochondria. The final mitochondrial pellet was washed with Buffer A to remove damaged mitochondria on the exterior of the pellet and then was resuspended in Buffer A. Protein concentration was determined using a bicinchoninic acid protein assay (Thermo Fisher Scientific, 23225). For mouse PAB and pediatric human samples, mitochondria were similarly isolated from flash-frozen RV tissue using the protocol described above. Pediatric samples were obtained from pediatric patients with HLHS (with and without RVF) and from non-diseased pediatric donors with normal cardiac anatomy.

### Assessment of mitochondrial electron transport capacity using high-resolution respirometry

Mitochondria isolated from freeze/thawed tissue are fully uncoupled and have been suggested to lose components of the TCA cycle throughout processing; however, they still maintain electron transport capacity^80^. Using high-resolution respirometry (Oroboros O2K), oxygen consumption (${J}_{{{\rm{O}}}_{2}}$) was measured in isolated mitochondria from human right ventricular muscle in Buffer D (105 mM K-MES, 30 mM KCl, 1 mM EGTA, 10 mM K_2_HPO_4_, 5 mM MgCl_2_-6H_2_O, 2.5 mg ml^−1^ BSA, pH 7.2) supplemented with 20 mM creatine at 37 °C. Alamethicin (10 µg ml^−1^) was supplemented to Buffer D during the experiment to ensure complete membrane permeabilization and access to substrates. Then, 5−15 mg of right ventricular tissue homogenate was added to the respiratory chambers at an oxygen concentration of approximately 200 µM. Once a baseline steady-state rate was established, substrates were injected as follows: NADH (1 mM) and cytochrome c (Cyt C) (5 µM), succinate (10 mM), rotenone (5 µM), antimycin A (5 µM), N,N,N′,N′-tetramethyl-*p*-phenylenediamine (TMPD, 0.5 mM) and ascorbate (1 mM). The mean ${J}_{{{\rm{O}}}_{2}}$ after each injection was measured and obtained once a steady-state flux was maintained for 5 minutes. Primary measurements included complex I (NADH + Cyt C), complex I + II (after succinate addition; coupled maximal OXPHOS capacity), complex II (rotenone-resistant respiration; succinate-driven with complex I inhibited) and complex IV (ascorbate + TMPD) maximal electron transfer capacity. Residual oxygen consumption (ROX) was defined as the ${J}_{{{\rm{O}}}_{2}}$ remaining after antimycin A addition (complex III inhibitor) and was subtracted from each preceding respiratory state to yield mitochondrial-specific values, in keeping with MITOEAGLE consortium conventions. All respiratory rates were normalized to the mass of starting mitochondrial protein (pmol O_2_ s^−1^ mg^−1^ mitochondrial protein) for cross-cohort consistency. Inter-group differences were assessed by Kruskal−Wallis test with Mann−Whitney pairwise post hoc comparisons (NF versus pRV, NF versus RVF, pRV versus RVF). Non-mitochondrial respiration (ROX) was independently tested across groups to confirm that observed mitochondrial deficits were not confounded by NADPH oxidase (NOX)-mediated changes in non-mitochondrial O_2_ consumption. The same O2K inhibitor titration protocol was applied to pediatric human samples. The pediatric respirometry cohort consisted of pediatric NF controls (non-diseased pediatric donors with normal cardiac anatomy, *n* = 7) and pediatric HLHS-palliated failing RVs (*n* = 9), forming a within-pediatric NF-versus-failing comparison matched for age, ventricular morphology (single-ventricle physiology in the failing arm) and load history.

Mouse PAB respirometry used a different, FAO-focused protocol on intact RV tissue fiber bundle preparations (rather than freeze/thaw-isolated mitochondria), reflecting the smaller available tissue mass and the goal of profiling β-oxidation capacity in the same run. Substrate addition was sequential: pyruvate + malate (complex I leak respiration in the absence of adenosine diphosphate (ADP)), ADP (complex I-driven coupled phosphorylating respiration), Cyt C (outer mitochondrial membrane integrity check; intact outer membrane defined as Cyt C / ADP ratio < 1.10), octanoyl-carnitine (complex I + FAO substrates) and succinate (complex I + II + FAO maximal coupled capacity). Respiratory control ratio (RCR) was calculated as ADP/pyruvate + malate. Inter-group differences (sham versus PAB-mild-RVF versus PAB-severe-RVF) at each timepoint (2 weeks, 2 months) were assessed by Kruskal−Wallis test with Mann−Whitney pairwise post hoc comparisons, in keeping with the adult and pediatric statistical framework.

### Immune repertoire profiling

**Extended Data Fig. 9:**
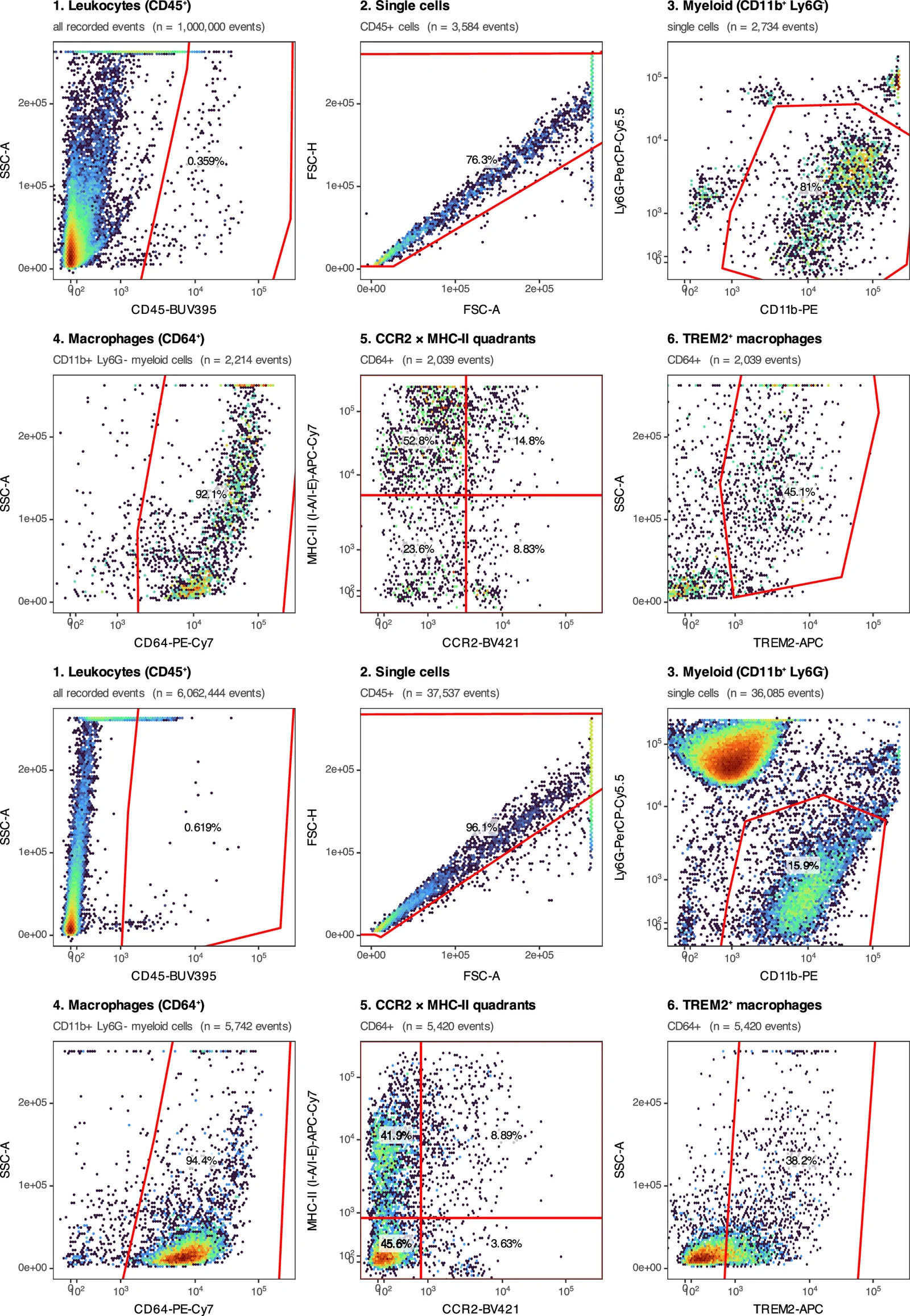
Flow cytometry gating strategy for mouse PAB cardiac macrophages. Complete gating cascade shown for one representative Sham right ventricle from each of the two independent staining cohorts (top, cohort 1; bottom, cohort 2). Plots are pseudocolour density (hexbin, log_10_ event count). Polygon and quadrant gates are those applied in FlowJo, and numbers give the percentage of the parent population indicated in each panel subtitle. Leukocytes were gated as CD45^+^ on all recorded events, followed by an FSC-A/FSC-H singlet gate; myeloid cells as CD11b^+^Ly-6G^−^; macrophages as CD64^+^; resident (CCR2^−^) versus recruited (CCR2^+^) macrophages and their MHC-II status by quadrant gate; and TREM2^+^ macrophages within the macrophage gate. Gates were drawn independently for each cohort on that cohort’s own negative populations, so absolute cut-points differ between the two panels. All analyses use within-experiment Sham-normalized values. Fluorescence axes are displayed on the original biexponential scale. The cascade shown is representative of, and was applied identically to, all n = 23 biologically independent animals (cohort 1, n = 12; cohort 2, n = 11). Per-gate percentages and event counts for every animal are provided in Source Data Extended Data Fig. 9. Source data

Mouse RV cardiac single-cell suspensions were stained with the following anti-mouse antibodies: CD45-BUV395 (clone 30-F11; BioLegend, 103191), CCR2-BV421 (SA203G11; BioLegend, 150605), Ly-6G^−^PerCP-Cy5.5 (1A8; BioLegend, 127615), CD11b-PE (M1/70; BioLegend, 101207), CD64-PE-Cy7 (X54-5/7.1; BioLegend, 139313), TREM2-APC (237920; R&D Systems, FAB17291A) and I-A/I-E (MHC-II)-APC-Cy7 (M5/114.15.2; BioLegend, 107627). Antibodies were used at the manufacturer-recommended amounts in a 100-µl staining volume: CD45-BUV395, CD11b-PE and I-A/I-E (MHC-II)-APC-Cy7 at 0.25 µg per 10^6^ cells; CCR2-BV421, Ly-6G^−^PerCP-Cy5.5 and CD64-PE-Cy7 at 0.5 µg per 10^6^ cells; and TREM2^−^APC at 10 µl per 10^6^ cells. Samples were acquired on an LSRFortessa (five-laser: 355 nm, 405 nm, 488 nm, 561 nm and 640 nm) using FACSDiva version 9.1 (BD Biosciences) and were analysed in FlowJo. Compensation was calculated from single-stain controls acquired in the same run. After exclusion of doublets (forward scatter area (FSC-A)/forward scatter height (FSC-H)), populations were gated as follows: leukocytes (CD45^+^); myeloid cells (CD45^+^CD11b^+^Ly-6G^−^; Ly-6G^−^ excluding neutrophils); and macrophages (CD64^+^ within the myeloid gate). Macrophage residency was defined by CCR2 alone: CCR2^−^ cells as resident macrophages and CCR2^+^ cells as recruited and monocyte derived. TREM2^+^ macrophages were gated within the macrophage gate. Surface MHC-II was quantified as the geometric mean fluorescence intensity (gMFI) of I-A / I-E on the CCR2^−^ resident gate. MHC-II gMFI was analyzed on a log scale (fluorescence intensity is log distributed). Trends across sham, mild-RVF and severe-RVF groups were tested with a batch-adjusted linear model on the per-animal (log transformed for gMFI) values. One sham was excluded for quality control. Gates were drawn separately for each of the two staining cohorts, on the internal negative population of each bivariate plot, and applied identically to every sample within a cohort. The complete gating cascade for a representative animal from each cohort is shown in Extended Data Fig. 9.

### Histology

Full-thickness human RV and mouse hearts were fixed in 4% paraformaldehyde, serially dehydrated in ethanol and paraffin embedded (FFPE). FFPE blocks were sectioned at 10 μm, mounted onto slides and stained with either trichrome or Sirius Red according to the manufacturerʼs instructions. Widefield brightfield microscopy was performed on a Keyence BZ-X810 microscope at ×10 with image stitching using BZ-X800 software. Image processing was performed in Fiji. For mouse slides, a region of interest (ROI) was manually drawn to isolate the RV free wall from the septum. Tissue borders and fibrosis-positive regions were identified using color deconvolution to generate binary masks. Statistical analysis was performed in Prism using Kruskal−Wallis with two-stage linear step-up Benjamini−Krieger−Yekutieli correction for pairwise comparisons.

For cardiomyocyte cross-sectional analyses, slides were stained with Alexa Fluor 488 WGA (Cell Signaling Technology, 1:100) and α-actinin (Abcam, ab68167, 1:200) and DAPI. Donkey anti-rabbit IgG Alexa Fluor 568 was used as secondary antibody (Invitrogen/Thermo Fisher Scientific, A10042, 1:500). Three representative stitched ×40 tile-scan images were acquired per subject in RV regions where cardiomyocytes were predominantly oriented in cross-section. Regions containing predominantly longitudinally oriented cardiomyocytes were manually excluded. Cellpose^82^ was used for cell segmentation on processed WGA images using the following settings: diameter 100, flow threshold 0.5, cell probability threshold 0.0, normalization percentiles 1.0−99.0 and dynamic Niter iterations 200. Generated images were subsequently analyzed in CellProfiler^83^. Cardiomyocytes were identified by filtering segmented objects based on α-actinin intensity, size and eccentricity to exclude non-cardiomyocytes, cell aggregates and obliquely sectioned cells. Minimum Feret diameter (MinFerDia) was extracted for each cardiomyocyte as a proxy for cross-sectional cell size (17,864 cardiomyocytes total across the 12 patients). For visualization (Extended Data Fig. 2i), per-patient median minimum Feret diameter was used. The full per-cell dataset is provided in Supplementary Data 16.

Statistical analyses were performed in R using linear mixed-effects models to account for the hierarchical structure of the data. Experimental group (NF/pRV/RVF for human studies and sham/mild-RVF/severe-RVF for mouse studies) was modeled as a fixed effect. Subject identity was included as a random intercept for all analyses, and slide was additionally included as a random intercept for mouse studies to account for repeated measurements obtained from the same specimen and histologic section. Pairwise group comparisons were performed using estimated marginal means with Benjamini−Hochberg correction for multiple testing.

### Clustering parameters

All clustering analyses used the following parameters unless otherwise specified. For bulk RNA-seq WGCNA, a soft-thresholding power was selected using the pickSoftThreshold function to achieve approximate scale-free topology. For snRNA-seq analysis, SCTransform normalization was applied with mitochondrial read percentage regressed out. PCA was computed using the top 3,000 variable features, and 50 principal components were retained for downstream analysis. Nearest neighbor graphs were constructed using FindNeighbors with *k* = 20. Clustering was performed using FindClusters with the Louvain algorithm. Clustering resolution was selected in a semi-supervised manner: nuclei were clustered at a range of resolutions (0.1−2.0), and the resolution yielding biologically interpretable clusters with distinct marker genes was selected. For the 12 major cell types, the default Seurat resolution of 0.8 was used. For subclustering, a resolution of 0.5 was used for fibroblast, endothelial, myeloid, mural and natural killer/T lineages. Cardiomyocyte subclustering used a resolution of 0.3. For EC hdWGCNA, seven modules were identified using a soft-thresholding power automatically selected via the pickSoftThreshold function to approximate scale-free topology. For Xenium subclustering, 30 principal components were used with resolutions of 0.3 (cardiomyocytes) to 0.5 (all other cell types). UMAP was computed using RunUMAP with n.neighbors = 30 and min.dist = 0.3.

#### Quantification and statistical analysis

Details of specific statistical analyses for each section, sample sizes and statistical tests used are given in the Methods and in the corresponding figure legends. All analysis was done in R using primarily custom-written scripts, which are available with deposited data, and the Seurat package. For pseudobulk DE, DESeq2 was run independently for each cell subtype, and pairwise disease comparison and *P* values were adjusted for multiple testing within each analysis using the Benjamini−Hochberg procedure. Cross-subtype correction was not applied, as each subtype−comparison pair tests an independent biological question.

### Software and versions

Analyses were performed in R version 4.3.3. Bulk RNA-seq processing used Trimmomatic version 0.39, kallisto version 0.51.1, tximport version 1.30.0, DESeq2 version 1.42.1 with apeglm version 1.24.0 shrinkage, sva version 3.50.0, edgeR version 4.0.16 and WGCNA version 1.73. Single-nucleus data were processed with Cell Ranger version 7.1.0, CellBender version 0.3.0 and decontX (celda version 1.18.1) and analyzed with Seurat version 5.1.0 (SeuratObject version 5.0.2, SeuratWrappers version 0.3.5), harmony version 1.2.3, hdWGCNA version 0.3.01, monocle3 version 1.3.7, decoupleR version 2.9.7 with CollecTRI, fgsea version 1.28.0, enrichR version 3.3, presto version 1.0.0 and glmGamPoi version 1.14.3. Xenium data were processed with Proseg version 1.1.9 and Sopa version 2.2.10 and analyzed with spacexr (RCTD) version 2.2.1, SPLIT version 0.1.3, SpaGE (GitHub commit edd0fb70; no tagged releases) and CellChat version 3. Histology was analyzed in Fiji/ImageJ version 1.54p, Cellpose version 3.1.1.2 and CellProfiler version 4.2.8. Flow cytometry was acquired with FACSDiva version 9.1, gated in FlowJo version 11.1.0 and reanalyzed with flowWorkspace version 4.14.3 and ggcyto version 1.30.2. Figures were produced with ggplot2 version 3.5.2, patchwork version 1.3.0, cowplot version 1.1.3, EnhancedVolcano version 1.20.0, Nebulosa version 1.12.1 and scCustomize version 3.0.0. Anthropic Claude was used to assist with the implementation and debugging of analysis code and to improve the clarity and readability of author-written text. All AI-assisted code and textual revisions were reviewed and verified by the authors, who take full responsibility for the final analyses and manuscript.

### Reporting summary

Further information on research design is available in the Nature Portfolio Reporting Summary linked to this article.

## Supplementary information

Reporting Summary

Peer Review File

Supplementary Tables 1−8 Supplementary Table 1: Patient demographics for the adult RV cohort (*n* = 142), including disease state classification (NF, pRV, RVF), underlying diagnosis, age, sex, RIN and platform allocation flags (in_snRNA_cohort, in_Xenium_cohort). Indirect identifiers are limited to three variables (sex, age, underlying diagnosis) to minimize participant reidentification risk. Supplementary Table 2: Hemodynamic parameters for the adult RV cohort (*n* = 142) used for disease-state stratification: right atrial pressure (RAP), pulmonary capillary wedge pressure (PCWP), RA:PCWP ratio (primary stratification variable), pulmonary arterial pressures, cardiac index and pulmonary vascular resistance index (PVRI). Supplementary Table 3: Clinical and demographic characteristics of the adult RV cohort compared between compensated (pRV, *n* = 78) and decompensated (RVF, *n* = 35) disease states. Continuous variables are presented as median (IQR) or mean ± s.d. and categorical variables as percentages. Nominal *P* values were calculated by Mann−Whitney *U*-test or unpaired *t*-test (continuous) and Fisherʼs exact test (categorical). RA, right atrial pressure; PCWP, pulmonary capillary wedge pressure; TAPSE, tricuspid annular plane systolic excursion; GFR, glomerular filtration rate; DCM, dilated cardiomyopathy. Supplementary Table 4: snRNA-seq cohort (*n* = 11): per-patient demographics joined from Supplementary Table 1, plus per-nucleus quality control metrics: nuclei recovered, median UMI count and feature count, mean mitochondrial percent, mean transcriptional entropy and mean intronic read percent. Supplementary Table 5: Clinical and demographic characteristics of the snRNA-seq cohort compared between compensated (pRV, *n* = 4) and decompensated (RVF, *n* = 3) disease states, matching the variable set of Supplementary Table 3. Continuous variables are presented as mean ± s.d. or median (IQR) and categorical variables as percentages. Nominal *P* values were calculated by unpaired *t*-test or Mann−Whitney *U*-test (continuous) and Fisherʼs exact test (categorical). Owing to the small per-group sample size, these comparisons are descriptive. Abbreviations are as in Supplementary Table 3. Supplementary Table 6: Patient demographics for the Xenium spatial transcriptomics cohort (*n* = 9), with tissue section details and per-section quality control metrics (cells captured, median UMI/features per cell, median cell area, section area, cell density). Supplementary Table 7: Mouse PAB snRNA-seq cohort (*n* = 10: 3 sham, 3 mild-RVF, 4 severe-RVF), with nuclei recovered and per-mouse echocardiographic parameters. Column abbreviations: BW, body weight (g); RVAD, RV area at diastole (mm^2^); RVAS, RV area at systole (mm^2^); FAC, fractional area change (%); RVFW, RV free-wall thickness (mm); TAPSE, tricuspid annular plane systolic excursion (mm); TDI S′, tissue-Doppler systolic wave; Pk Grad, peak gradient (mmHg); Pk Vel, peak velocity (mm s^−1^); TR, tricuspid regurgitation. These are distinct animals from the echo/respirometry cohort (Supplementary Table 8) despite shared ID integers. Supplementary Table 8: Mouse PAB 2-week + 2-month (8-week) echocardiography/respirometry cohort—the full echo-phenotyped animal set (distinct mice from the snRNA-seq cohort in Supplementary Table 7), categorized as PAB versus sham (category) and sorted by category. Presented as two vertically stacked sub-tables: week 2 and week 8. ID, category and BW (g) are repeated in each; the week 8 BW (g) is the 8-week surgical body weight. The week 2 group column flags whether a mouse is ‘Week 2 only’ (respirometry-only, no 8-week echo) or ‘Week 8’ (also echoed at 8 weeks); the week 8 sub-table lists exactly the latter. Echo abbreviations match Supplementary Table 7 (BW, body weight (g); RVAD, RV area at diastole (mm^2^); RVAS, RV area at systole (mm^2^); FAC, fractional area change (%); RVFW, RV free-wall thickness (mm); TAPSE, tricuspid annular plane systolic excursion (mm); TDI S′; Pk Grad, peak gradient (mmHg); Pk Vel, peak velocity (mm s^−1^); TR, tricuspid regurgitation); echo values are shown to three significant figures. Week 8 mouse ID 17 died during the echocardiogram, so only the parameters acquired before death are reported (subsequent metrics—TAPSE, TDI S′, Pk Grad, Pk Vel, TR—are shown as NA).

Supplementary Data 1 Bulk RNA-seq DEGs: NF versus pRV, NF versus RVF and pRV versus RVF comparisons, including log_2_FC, *P* value and adjusted *P* value for all tested genes.

Supplementary Data 2 Bulk RNA-seq pathway enrichment results (Reactome, Gene Ontology Biological Processes, GTEx Aging Signatures) for DEGs across all disease-state comparisons.

Supplementary Data 3 Bulk RNA-seq stepwise gene expression trends: genes with significant monotonic increase or decrease from NF to pRV to RVF (*P*_adj_ < 0.1), gene set membership and associated pathway enrichments.

Supplementary Data 4 Bulk WGCNA module gene memberships: module assignments for all genes across 29 co-expression modules, with module−trait correlations and cell type specificity scores.

Supplementary Data 5 PAH comparison: concordant and discordant DEGs between our RV cohort and published PAH-associated RV failure transcriptomic data, with pathway enrichment of concordant (shared mitochondrial suppression/IFN induction) and discordant (complement) gene sets.

Supplementary Data 6 Single-nucleus RNA-seq cell type annotations: cluster assignments for all 61,398 nuclei across 12 major cell types, including UMAP coordinates and quality control metrics.

Supplementary Data 7 Single-nucleus RNA-seq subclustering marker genes: pooled FindMarkers results for all 34 subtypes across all cell lineages (cardiomyocyte, myeloid, endothelial, fibroblast, mural, NK/T, adipocyte, neuronal, epicardial), including log_2_FC, *P* value, adjusted *P* value and percent expression.

Supplementary Data 8 Single-nucleus RNA-seq pseudobulk DESeq2: coarse-grained (12 major cell types) DE results for NF versus pRV, NF versus RVF and pRV versus RVF comparisons, including log_2_FC, *P* value and adjusted *P* value.

Supplementary Data 9 Single-nucleus RNA-seq pseudobulk DESeq2: fine-grained (34 subtypes) DE results for NF versus pRV, NF versus RVF and pRV versus RVF comparisons within each subtype.

Supplementary Data 10 Xenium spatial transcriptomics gene panel and provenance: complete list of 477 target genes (377 predesigned, 100 custom) and 64 negative control probes (541 total) (design ID: 44Z7X8); for each gene used in Xenium analyses, indicates whether it was directly measured on the 477-gene panel or computationally imputed via SpaGE from the snRNA-seq reference. Key DEGs discussed in the main text are annotated with the cell type context in which they appear and whether findings depend on panel genes (independent cross-platform validation) or imputed genes (concordant with, but not independent of, snRNA-seq).

Supplementary Data 11 Xenium pseudobulk DESeq2: DE results for NF versus pRV, NF versus RVF and pRV versus RVF comparisons within each spatially resolved cell type, including cross-platform concordance with snRNA-seq.

Supplementary Data 12 Xenium subclustering: subtype assignments and marker genes for spatially resolved cell populations within each of the 12 major cell types after Proseg resegmentation, RCTD annotation and SPLIT correction.

Supplementary Data 13 Xenium niche composition: cell type proportions within each spatial niche (*k* = 100 nearest neighbors, CLR-transformed, collapsed cardiomyocyte subtypes), niche cluster assignments and niche abundance across NF, pRV and RVF disease states.

Supplementary Data 14 CellChat ligand−receptor interaction results: top-ranked spatially resolved ligand−receptor pairs for each disease state (NF, pRV, RVF).

Supplementary Data 15 Mitochondrial respirometry: Oroboros high-resolution respirometry measurements for adult RVF (NF, pRV, RVF), mouse PAB (sham, 2-week, 2-month), and pediatric RV (ped-NF, ped-RVF) samples, including complex I, complex II, and maximal respiratory capacity.

Supplementary Data 16 WGA cardiomyocyte cross-sectional area measurements: per-cell minimum Feret diameter and area from CellProfiler segmentation of WGA-stained FFPE human RV sections, with patient ID, disease group, image number and per-cell measurements. *n* = 4 patients per group (NF, pRV, RVF); 17,864 cardiomyocytes total.

Supplementary Data 17 Myeloid transcriptional program gene lists: curated gene sets for the four pan-myeloid programs scored in Fig. 6g,h and Extended Data Fig. 7f: GR transactivation targets (*FKBP5*, *TSC22D3*, *ZBTB16*, *KLF9*, *ANGPTL4*), MHC-II/antigen presentation (*CIITA*-driven), the NLRP3 inflammasome and the type I IFN signature.

Supplementary Data 18 Endothelial hdWGCNA module gene memberships: module assignments, hub genes, and module−trait correlations for EC-specific WGCNA.

Supplementary Data 19 Mouse PAB model: cell type annotations, subtype assignments, marker genes, and DEG results for sham versus mild RVF versus severe RVF comparisons across all recovered cell types.

Supplementary Data 20 *Cx3cr1*-tdTomato lineage tracing: bulk RNA-seq of sorted RV and LV macrophages from PAB and sham mice, including DE results.

Supplementary Data 21 Pediatric snRNA-seq cell type annotations: cluster assignments across ped-NF, ped-HLHS, and ped-RVF samples, with UMAP coordinates and quality control metrics.

Supplementary Data 22 Pediatric snRNA-seq DEGs: pseudobulk DE results across pediatric disease states, including comparisons with adult RV DEGs for concordance analysis.

## Source data

Source Data Fig. 1 Statistical source data for the graphs in Fig. 1, one tab per panel.

Source Data Fig. 2 Statistical source data for the graphs in Fig. 2, one tab per panel.

Source Data Fig. 3 Statistical source data for the graphs in Fig. 3, one tab per panel.

Source Data Fig. 4 Statistical source data for the graphs in Fig. 4, one tab per panel.

Source Data Fig. 5 Statistical source data for the graphs in Fig. 5, one tab per panel.

Source Data Fig. 6 Statistical source data for the graphs in Fig. 6, one tab per panel.

Source Data Fig. 7 Statistical source data for the graphs in Fig. 7, one tab per panel.

Source Data Fig. 8 Statistical source data for the graphs in Fig. 8, one tab per panel.

Source Data Extended Data Fig. 1 Statistical source data for the graphs in Extended Data Fig. 1, one tab per panel.

Source Data Extended Data Fig. 2 Statistical source data for the graphs in Extended Data Fig. 2, one tab per panel.

Source Data Extended Data Fig. 3 Statistical source data for the graphs in Extended Data Fig. 3, one tab per panel.

Source Data Extended Data Fig. 4 Statistical source data for the graphs in Extended Data Fig. 4, one tab per panel.

Source Data Extended Data Fig. 5 Statistical source data for the graphs in Extended Data Fig. 5, one tab per panel.

Source Data Extended Data Fig. 6 Statistical source data for the graphs in Extended Data Fig. 6, one tab per panel.

Source Data Extended Data Fig. 7 Statistical source data for the graphs in Extended Data Fig. 7, one tab per panel.

Source Data Extended Data Fig. 8 Statistical source data for the graphs in Extended Data Fig. 8, one tab per panel.

Source Data Extended Data Fig. 9 Source data for Extended Data Fig. 9. Per-gate percentages and raw event counts for every animal in both staining cohorts (*n* = 23; cohort 1 *n* = 12, cohort 2 *n* = 11).

## Data Availability

The bulk RNA-seq, snRNA-seq and Xenium spatial transcriptomics data generated in this study have been deposited in the Gene Expression Omnibus under the following accessions: adult right ventricular bulk RNA-seq, GSE345645; adult right ventricular snRNA-seq, GSE345646; Xenium spatial transcriptomics, GSE345643; *Cx3cr1* lineage-traced cardiac macrophage bulk RNA-seq, GSE345518; and mouse pulmonary artery banding snRNA-seq, GSE345854. The pediatric dataset is available via Lavine et al.^50^. Previously published data reanalyzed here are available under accession GSE240941. Processed data objects underlying the figures are archived at Zenodo (https://doi.org/10.5281/zenodo.20115563)^84^. Source Data are provided with this paper for all main and extended data figures.

## Code availability

The analysis pipeline and all code required to generate the figures in this paper are available at https://github.com/ikuznet1/RV_Atlas.
