# Supplementary material for: Human adult and pediatric multimodal transcriptomic atlas of progression from pressure-loaded to failing right ventricle
Source: Nat Cardiovasc Res. 2026 Oct 9;5(11):1133–58. doi: 10.1038/s44161-026-00885-5 (PMC13753616; doi:10.1038/s44161-026-00885-5)
Supplement: Supplementary file 1 [file 44161_2026_885_MOESM1_ESM.pdf]

nature portfolio

Corresponding author(s): Zoltan Arany

Last updated by author(s): Aug 11, 2026

Reporting Summary

Nature Portfolio wishes to improve the reproducibility of the work that we publish. This form provides structure for consistency and transparency in reporting. For further information on Nature Portfolio policies, see our Editorial Policies and the Editorial Policy Checklist.

Statistics

For all statistical analyses, confirm that the following items are present in the figure legend, table legend, main text, or Methods section.

n/a	Confirmed	
☐	☒	The exact sample size (*n*) for each experimental group/condition, given as a discrete number and unit of measurement
☐	☒	A statement on whether measurements were taken from distinct samples or whether the same sample was measured repeatedly
☐	☒	The statistical test(s) used AND whether they are one- or two-sided<br>*Only common tests should be described solely by name; describe more complex techniques in the Methods section.*
☐	☒	A description of all covariates tested
☐	☒	A description of any assumptions or corrections, such as tests of normality and adjustment for multiple comparisons
☐	☒	A full description of the statistical parameters including central tendency (e.g. means) or other basic estimates (e.g. regression coefficient) AND variation (e.g. standard deviation) or associated estimates of uncertainty (e.g. confidence intervals)
☐	☒	For null hypothesis testing, the test statistic (e.g. *F*, *t*, *r*) with confidence intervals, effect sizes, degrees of freedom and *P* value noted<br>*Give P values as exact values whenever suitable.*
☒	☐	For Bayesian analysis, information on the choice of priors and Markov chain Monte Carlo settings
☐	☒	For hierarchical and complex designs, identification of the appropriate level for tests and full reporting of outcomes
☐	☒	Estimates of effect sizes (e.g. Cohen's *d*, Pearson's *r*), indicating how they were calculated

Our web collection on statistics for biologists contains articles on many of the points above.

Software and code

Policy information about availability of computer code

Data collection	Sequencing: Illumina NovaSeq/HiSeq (Azenta Life Sciences); demultiplexing with bcl2fastq. Single-nucleus libraries: 10x Genomics Chromium; raw reads processed with CellRanger v7.1.0. Spatial transcriptomics: 10x Genomics Xenium Analyzer. Flow cytometry: BD LSRFortessa (five-laser: 355, 405, 488, 561, 640 nm), acquired with BD FACSDiva v9.1. Respirometry: Oroboros Oxygraph-2k with DatLab. Microscopy: Keyence BZ-X810 with BZ-X800 acquisition software. RNA quality: Agilent 2100 Bioanalyzer with 2100 Expert software.
Data analysis	All analysis was performed in R v4.3.3 unless stated otherwise.<br>Bulk RNA-seq: Trimmomatic v0.39, kallisto v0.51.1, tximport v1.30.0, DESeq2 v1.42.1 with apeglm v1.24.0 shrinkage, sva v3.50.0, edgeR v4.0.16, WGCNA v1.73.<br>Single-nucleus RNA-seq: CellRanger v7.1.0, CellBender v0.3.0, decontX (celda v1.18.1), Seurat v5.1.0 with SeuratObject v5.0.2, SeuratWrappers v0.3.5, harmony v1.2.3, hdWGCNA v0.3.01, monocle3 v1.3.7, decoupleR v2.9.7 with CollecTRI, fgsea v1.28.0, enrichR v3.3, presto v1.0.0, glmGamPoi v1.14.3.<br>Spatial transcriptomics (Xenium): Proseg v1.1.9, Sopa v2.2.10, RCTD via spacexr v2.2.1, SPLIT v0.1.3, SpaGE (GitHub commit edd0fb70; no tagged releases), CellChat v3.<br>Histology and imaging: FIJI/ImageJ v1.54p, Cellpose v3.1.1.2, CellProfiler v4.2.8.<br>Flow cytometry: FlowJo v11.1.0, with re-analysis of exported workspaces in flowWorkspace v4.14.3 and ggcyto v1.30.2.<br>Visualisation: ggplot2 v3.5.2, patchwork v1.3.0, cowplot v1.1.3, EnhancedVolcano v1.20.0, Nebulosa v1.12.1, scCustomize v3.0.0.<br>Custom analysis code is available at https://github.com/ikuznet1/RV_Atlas and archived at Zenodo (DOI 10.5281/zenodo.20115563).

For manuscripts utilizing custom algorithms or software that are central to the research but not yet described in published literature, software must be made available to editors and reviewers. We strongly encourage code deposition in a community repository (e.g. GitHub). See the Nature Portfolio guidelines for submitting code & software for further information.

Data

Policy information about availability of data

All manuscripts must include a data availability statement. This statement should provide the following information, where applicable:

- Accession codes, unique identifiers, or web links for publicly available datasets
- A description of any restrictions on data availability
- For clinical datasets or third party data, please ensure that the statement adheres to our policy

The bulk RNA-seq, single-nucleus RNA-seq and Xenium spatial transcriptomics data generated in this study have been deposited in the Gene Expression Omnibus under the following accessions: adult right ventricular bulk RNA-seq, GSE345645; adult right ventricular single-nucleus RNA-seq, GSE345646; Xenium spatial transcriptomics, GSE345643; CX3CR1 lineage-traced cardiac macrophage bulk RNA-seq, GSE345518; and murine pulmonary artery banding single-nucleus RNA-seq, GSE345854. Pediatric single-nucleus RNA-seq data were generated by collaborators and are reported separately by Kadyrov et al. (Research Square, 2025; doi:10.21203/rs.3.rs-6689087/v1), through which they are available. Previously published data re-analysed here are available under accession GSE240941. Processed data objects underlying the figures are archived at Zenodo (DOI: 10.5281/zenodo.20115563). Source Data are provided with this paper for all main and Extended Data figures.

Research involving human participants, their data, or biological material

Policy information about studies with human participants or human data. See also policy information about sex, gender (identity/presentation), and sexual orientation and race, ethnicity and racism.

Reporting on sex and gender	Sex was obtained from clinical records for every subject and is reported per subject in Table S1. Sex was not a selection criterion; subjects were included on the basis of haemodynamic classification and tissue availability. Sex was explicitly accounted for in the adult bulk RNA-seq analysis: surrogate variable analysis was run with sex, age, race, batch and clinical covariates in the null model, so sex-associated variation is removed before differential testing. The single-nucleus (n=11), spatial (n=9) and pediatric (n=14) cohorts are too small to support sex-disaggregated analysis, and results from those cohorts are not stratified by sex.
Reporting on race, ethnicity, or other socially relevant groupings	Patient ethnicity is reported at group level in Tables S3 and S5 (Caucasian, Black, Other). Per-participant ethnicity has been withheld from Table S1 to limit the number of indirect identifiers carried in a per-participant table, in line with the editorial request to reduce these to three variables (sex, age and underlying diagnosis). Ethnicity was recorded for every adult subject and was included as a covariate in the surrogate variable analysis null model for the bulk RNA-seq differential expression.
Population characteristics	Table S1 reports, per participant, disease-state classification (NF, pRV, RVF), underlying diagnosis, age, sex, RNA integrity number and platform allocation. Haemodynamic parameters are reported per participant in Table S2, and the full clinical and demographic comparison between pressure-loaded and failing groups, including anthropometrics, renal function, comorbidities and medications, is reported at group level in Tables S3 and S5.
Recruitment	Prospective informed consent was obtained from transplant recipients, parents/guardians for subjects less than 18 years old with assent as indicated, or from next-of-kin in the case of deceased organ donors.
Ethics oversight	The study protocols were approved by the University of Pennsylvania IRB (study number 802781) and the Children's Hospital of Philadelphia IRB (study number 22-020464), as well as by the Gift-of-Life Donor program.

Note that full information on the approval of the study protocol must also be provided in the manuscript.

Field-specific reporting

Please select the one below that is the best fit for your research. If you are not sure, read the appropriate sections before making your selection.

☒ Life sciences ☐ Behavioural & social sciences ☐ Ecological, evolutionary & environmental sciences

For a reference copy of the document with all sections, see nature.com/documents/nr-reporting-summary-flat.pdf

Life sciences study design

All studies must disclose on these points even when the disclosure is negative.

Sample size	No statistical method was used to predetermine sample size and no power calculation was performed. All human cohorts were limited by the availability of non-renewable explanted tissue, and murine group sizes follow those established for this pulmonary artery banding model. The sample sizes are sufficient in that the primary comparisons remained significant after multiple-testing correction and the principal findings replicated across independent platforms and cohorts.<br><br>Adult bulk RNA-seq: all 142 subjects meeting the haemodynamic classification criteria (NF 29, pRV 78, RVF 35).<br>Adult single-nucleus RNA-seq: 11 subjects, limited by samples with sufficient RNA quality.<br>Xenium spatial transcriptomics: 9 sections, limited by tissue geometry suitable for sectioning.<br>Pediatric single-nucleus RNA-seq: 14 subjects, comprising all available single-ventricle and donor right ventricles.<br>Adult mitochondrial respirometry: NF 9, pRV 10, RVF 8.<br>Pediatric mitochondrial respirometry: donor 7, failing single-ventricle 9.

	Cardiomyocyte cross-sectional area histology: 12 subjects (17,864 cardiomyocytes).<br>Murine single-nucleus RNA-seq: 3 sham, 3 mild, 4 severe.<br>Murine flow cytometry: 7 sham, 7 mild, 8 severe.<br>Murine respirometry: 2 weeks post-surgery, 8 sham, 6 mild, 7 severe; 2 months post-surgery, 7 sham, 6 mild, 5 severe.<br>Murine resident-macrophage bulk RNA-sequencing: 3 sham and 5 pulmonary artery banded animals, each contributing paired left- and right-ventricular sorted populations (16 libraries).
Data exclusions	Pre-established quality-control criteria, defined in Methods, were applied. One adult snRNA-seq sample was excluded for failing sequencing quality control. One sham animal was excluded from the flow cytometry analysis for quality control. Nuclei and cells were filtered by the QC thresholds stated in Methods. No other data were excluded.
Replication	Reproducibility was addressed by replication across independent platforms and cohorts rather than by repeated runs of the same assay, because each human specimen is non-renewable and cannot be re-sampled. Every central transcriptional claim was required to be supported by at least two independent modalities among bulk RNA-seq (142 subjects), single-nucleus RNA-seq (11 adults) and Xenium spatial transcriptomics (9 sections), which derive from overlapping but non-identical subject sets.<br><br>All attempts at replication of the principal findings were successful. The cardiomyocyte decline in nuclear-encoded mitochondrial and OXPHOS transcripts was reproduced in bulk RNA-seq and in single-nucleus RNA-seq of the adult cohort, reproduced independently in the murine PAB model, and confirmed by an orthogonal non-transcriptional assay (high-resolution respirometry on isolated mitochondria). The myeloid and endothelial compartment shifts were reproduced between single-nucleus RNA-seq and Xenium spatial transcriptomics.<br><br>Two results did not reproduce across cohorts. First, the cardiomyocyte OXPHOS deficit seen in adult RVF and in the murine PAB model was not present in the pediatric single-ventricle cohort, where mitochondrial transcription was statistically flat and respiration was preserved. The pediatric right ventricle is therefore described as metabolically spared, and the deficit is not claimed to be universal. Second, fibroblast fold changes correlated poorly between the human and murine datasets (Extended Data Fig. 6), so the murine fibroblast findings are not presented as conserved.
Randomization	Human disease state (NF, pRV, RVF) is an intrinsic property of the explanted heart, not an assigned condition: subjects were classified from pre-specified haemodynamic criteria (right atrial pressure and the right atrial to pulmonary capillary wedge pressure ratio, defined in Methods) applied before any transcriptomic analysis, so allocation to experimental groups does not apply. For the murine studies, age-matched male mice of the same strain and cohort (9-week-old C57BL/6J for pulmonary artery banding; 8-week-old CX3CR1-CreER;Rosa26-tdTomato for the resident-macrophage bulk RNA-sequencing) were assigned to banding or sham surgery. RVF severity was not assigned by the investigators: mild versus severe RVF was determined post hoc from echocardiographic criteria (TAPSE, fractional area change and RV systolic function) measured after surgery.
Blinding	Blinding was not possible during tissue collection, library preparation or sequencing, because disease state is established at the point of collection and defines the analysis contrast. Operator influence was instead limited at the quantification stage. Histological fibrosis was measured by colour deconvolution with fixed thresholds, and cardiomyocyte cross-sectional area by automated segmentation, both applied identically to every section. Flow cytometry gates were drawn once per staining cohort and applied unchanged to every sample in that cohort. All transcriptomic group comparisons were run by scripted pipelines with fixed parameters and pre-specified models, so no per-sample operator decisions entered the analysis.

Behavioural & social sciences study design

All studies must disclose on these points even when the disclosure is negative.

Study description	*Briefly describe the study type including whether data are quantitative, qualitative, or mixed-methods (e.g. qualitative cross-sectional, quantitative experimental, mixed-methods case study).*
Research sample	*State the research sample (e.g. Harvard university undergraduates, villagers in rural India) and provide relevant demographic information (e.g. age, sex) and indicate whether the sample is representative. Provide a rationale for the study sample chosen. For studies involving existing datasets, please describe the dataset and source.*
Sampling strategy	*Describe the sampling procedure (e.g. random, snowball, stratified, convenience). Describe the statistical methods that were used to predetermine sample size OR if no sample-size calculation was performed, describe how sample sizes were chosen and provide a rationale for why these sample sizes are sufficient. For qualitative data, please indicate whether data saturation was considered, and what criteria were used to decide that no further sampling was needed.*
Data collection	*Provide details about the data collection procedure, including the instruments or devices used to record the data (e.g. pen and paper, computer, eye tracker, video or audio equipment) whether anyone was present besides the participant(s) and the researcher, and whether the researcher was blind to experimental condition and/or the study hypothesis during data collection.*
Timing	*Indicate the start and stop dates of data collection. If there is a gap between collection periods, state the dates for each sample cohort.*
Data exclusions	*If no data were excluded from the analyses, state so OR if data were excluded, provide the exact number of exclusions and the rationale behind them, indicating whether exclusion criteria were pre-established.*
Non-participation	*State how many participants dropped out/declined participation and the reason(s) given OR provide response rate OR state that no participants dropped out/declined participation.*
Randomization	*If participants were not allocated into experimental groups, state so OR describe how participants were allocated to groups, and if*

Randomization	*allocation was not random, describe how covariates were controlled.*

Ecological, evolutionary & environmental sciences study design

All studies must disclose on these points even when the disclosure is negative.

Study description	*Briefly describe the study. For quantitative data include treatment factors and interactions, design structure (e.g. factorial, nested, hierarchical), nature and number of experimental units and replicates.*
Research sample	*Describe the research sample (e.g. a group of tagged Passer domesticus, all Stenocereus thurberi within Organ Pipe Cactus National Monument), and provide a rationale for the sample choice. When relevant, describe the organism taxa, source, sex, age range and any manipulations. State what population the sample is meant to represent when applicable. For studies involving existing datasets, describe the data and its source.*
Sampling strategy	*Note the sampling procedure. Describe the statistical methods that were used to predetermine sample size OR if no sample-size calculation was performed, describe how sample sizes were chosen and provide a rationale for why these sample sizes are sufficient.*
Data collection	*Describe the data collection procedure, including who recorded the data and how.*
Timing and spatial scale	*Indicate the start and stop dates of data collection, noting the frequency and periodicity of sampling and providing a rationale for these choices. If there is a gap between collection periods, state the dates for each sample cohort. Specify the spatial scale from which the data are taken*
Data exclusions	*If no data were excluded from the analyses, state so OR if data were excluded, describe the exclusions and the rationale behind them, indicating whether exclusion criteria were pre-established.*
Reproducibility	*Describe the measures taken to verify the reproducibility of experimental findings. For each experiment, note whether any attempts to repeat the experiment failed OR state that all attempts to repeat the experiment were successful.*
Randomization	*Describe how samples/organisms/participants were allocated into groups. If allocation was not random, describe how covariates were controlled. If this is not relevant to your study, explain why.*
Blinding	*Describe the extent of blinding used during data acquisition and analysis. If blinding was not possible, describe why OR explain why blinding was not relevant to your study.*

Did the study involve field work? ☐ Yes ☐ No

Field work, collection and transport

Field conditions	*Describe the study conditions for field work, providing relevant parameters (e.g. temperature, rainfall).*
Location	*State the location of the sampling or experiment, providing relevant parameters (e.g. latitude and longitude, elevation, water depth).*
Access & import/export	*Describe the efforts you have made to access habitats and to collect and import/export your samples in a responsible manner and in compliance with local, national and international laws, noting any permits that were obtained (give the name of the issuing authority, the date of issue, and any identifying information).*
Disturbance	*Describe any disturbance caused by the study and how it was minimized.*

Reporting for specific materials, systems and methods

We require information from authors about some types of materials, experimental systems and methods used in many studies. Here, indicate whether each material, system or method listed is relevant to your study. If you are not sure if a list item applies to your research, read the appropriate section before selecting a response.

Materials & experimental systems

n/a	Involved in the study	
☐	☒	Antibodies
☒	☐	Eukaryotic cell lines
☒	☐	Palaeontology and archaeology
☐	☒	Animals and other organisms
☐	☒	Clinical data
☒	☐	Dual use research of concern
☒	☐	Plants

Methods

n/a	Involved in the study	
☒	☐	ChIP-seq
☐	☒	Flow cytometry
☒	☐	MRI-based neuroimaging

Antibodies

Antibodies used

Flow cytometry panel (murine RV immunophenotyping). Antibodies were used at the manufacturer-recommended amounts in a 100 uL staining volume:
CD45-BUV395 (clone 30-F11, BioLegend 103191), 0.25 ug per 1e6 cells
CCR2-BV421 (clone SA203G11, BioLegend 150605), 0.5 ug per 1e6 cells
Ly-6G-PerCP-Cy5.5 (clone 1A8, BioLegend 127615), 0.5 ug per 1e6 cells
CD11b-PE (clone M1/70, BioLegend 101207), 0.25 ug per 1e6 cells
CD64-PE-Cy7 (clone X54-5/7.1, BioLegend 139313), 0.5 ug per 1e6 cells
TREM2-APC (clone 237920, R&D Systems FAB17291A), 10 uL per 1e6 cells
I-A/I-E (MHC-II)-APC-Cy7 (clone M5/114.15.2, BioLegend 107627), 0.25 ug per 1e6 cells

FACS sorting for resident-macrophage bulk RNA-sequencing, at the manufacturer-recommended amounts:
CD45 (clone 30-F11, BioLegend 103132)
CD11b (clone M1/70, BioLegend 101223)
CD64 (clone X54-5/7.1, BioLegend 139323)

Immunofluorescence (cardiomyocyte cross-sectional area):
alpha-actinin, rabbit polyclonal (Abcam ab68167), 1:200
Wheat germ agglutinin-Alexa Fluor 488 (Cell Signaling Technology), 1:100 - a lectin, not an antibody
Donkey anti-rabbit IgG Alexa Fluor 568 secondary (Invitrogen/Thermo Fisher A10042), 1:500

Lot numbers were not recorded at the time of use and are therefore not available; supplier and catalogue number are given for every antibody above.

Validation

All antibodies were commercially sourced and used for the species and application for which the supplier validates them; catalogue numbers are listed above and each supplier provides validation data (species reactivity, application and representative staining) on the corresponding product page.

Flow cytometry and sorting antibodies. The clones used are established for murine cardiac myeloid phenotyping: 30-F11 (CD45), M1/70 (CD11b), 1A8 (Ly-6G), X54-5/7.1 (CD64), SA203G11 (CCR2) and M5/114.15.2 (I-A/I-E). The same clones were used for immunophenotyping and for FACS sorting. Specificity was additionally supported within our own data by the expected staining behaviour: Ly-6G resolved a discrete neutrophil population that was excluded from the myeloid gate; CD64 resolved a distinct macrophage population within the CD11b+ Ly-6G- gate; and CCR2, MHC-II and TREM2 each resolved separable positive and negative populations on CD64+ macrophages. Spectral spillover was corrected using single-stain compensation controls acquired in the same run. In the lineage-tracing experiment, the CD45+CD11b+CD64+ sort gate was further restricted to tdTomato+ cells, providing a genetic label independent of the antibody staining.

Immunofluorescence antibodies. Anti-alpha-actinin (Abcam ab68167) is validated by the supplier for immunohistochemistry on paraffin sections in mouse and human, and its performance was confirmed in situ by the expected sarcomeric striated staining restricted to cardiomyocytes; it was used to discriminate cardiomyocytes from non-cardiomyocytes during segmentation, so off-target staining would have been evident as mis-segmentation. The donkey anti-rabbit Alexa Fluor 568 secondary (Invitrogen A10042) is a species-specific secondary; no-primary controls showed no specific signal.

Eukaryotic cell lines

Policy information about cell lines and Sex and Gender in Research

Cell line source(s)

State the source of each cell line used and the sex of all primary cell lines and cells derived from human participants or vertebrate models.

Authentication

Describe the authentication procedures for each cell line used OR declare that none of the cell lines used were authenticated.

Mycoplasma contamination

Confirm that all cell lines tested negative for mycoplasma contamination OR describe the results of the testing for mycoplasma contamination OR declare that the cell lines were not tested for mycoplasma contamination.

Commonly misidentified lines
(See ICLAC register)

Name any commonly misidentified cell lines used in the study and provide a rationale for their use.

Palaeontology and Archaeology

Specimen provenance	*Provide provenance information for specimens and describe permits that were obtained for the work (including the name of the issuing authority, the date of issue, and any identifying information). Permits should encompass collection and, where applicable, export.*
Specimen deposition	*Indicate where the specimens have been deposited to permit free access by other researchers.*
Dating methods	*If new dates are provided, describe how they were obtained (e.g. collection, storage, sample pretreatment and measurement), where they were obtained (i.e. lab name), the calibration program and the protocol for quality assurance OR state that no new dates are provided.*

☐ Tick this box to confirm that the raw and calibrated dates are available in the paper or in Supplementary Information.

Ethics oversight	*Identify the organization(s) that approved or provided guidance on the study protocol, OR state that no ethical approval or guidance was required and explain why not.*

Note that full information on the approval of the study protocol must also be provided in the manuscript.

Animals and other research organisms

Policy information about studies involving animals; ARRIVE guidelines recommended for reporting animal research, and Sex and Gender in Research

Laboratory animals	Mus musculus; males only.<br>Pulmonary artery banding (PAB) and sham surgery: male C57BL/6J mice (Jackson Laboratory #000664), 9 weeks of age at surgery.<br>Resident-macrophage lineage tracing and bulk RNA-sequencing: male heterozygous Cx3cr1-CreER; Rosa26-tdTomato mice, bred in house from Cx3cr1-CreER (JAX #020940) and Rosa26-tdTomato (JAX #007914); tamoxifen chow from 3 weeks of age for one week, PAB or sham surgery at 8 weeks of age, harvested 2 weeks after surgery.<br>Other experiments were initiated at 6-10 weeks (typically 7-8 weeks) of age, with littermate controls. Only male mice were used, to remove sex as a biological variable.<br>Housing: University of Pennsylvania vivarium, 12:12 h light-dark cycle (lights on 07:00, off 19:00), ambient temperature 20-23 C, relative humidity 30-70%, with free access to normal chow and water.<br>Per-experiment group sizes are given in Table S7 and Table S8.
Wild animals	NA
Reporting on sex	Exclusively male mice were used, to exclude sex as a biological variable. Sex-dependence of the murine findings was therefore not assessed, and this is noted as a limitation.
Field-collected samples	NA
Ethics oversight	Mouse protocols were approved by the University of Pennsylvania IACUC (protocols 805255 and 805309)

Note that full information on the approval of the study protocol must also be provided in the manuscript.

Clinical data

Policy information about clinical studies
All manuscripts should comply with the ICMJE guidelines for publication of clinical research and a completed CONSORT checklist must be included with all submissions.

Clinical trial registration	NA
Study protocol	NA
Data collection	NA
Outcomes	NA

Dual use research of concern

Policy information about dual use research of concern

Hazards

Could the accidental, deliberate or reckless misuse of agents or technologies generated in the work, or the application of information presented in the manuscript, pose a threat to:

No	Yes	
☐	☐	Public health
☐	☐	National security
☐	☐	Crops and/or livestock
☐	☐	Ecosystems
☐	☐	Any other significant area

Experiments of concern

Does the work involve any of these experiments of concern:

No	Yes	
☐	☐	Demonstrate how to render a vaccine ineffective
☐	☐	Confer resistance to therapeutically useful antibiotics or antiviral agents
☐	☐	Enhance the virulence of a pathogen or render a nonpathogen virulent
☐	☐	Increase transmissibility of a pathogen
☐	☐	Alter the host range of a pathogen
☐	☐	Enable evasion of diagnostic/detection modalities
☐	☐	Enable the weaponization of a biological agent or toxin
☐	☐	Any other potentially harmful combination of experiments and agents

Plants

Seed stocks	NA
Novel plant genotypes	NA
Authentication	NA

ChIP-seq

Data deposition

☐ Confirm that both raw and final processed data have been deposited in a public database such as GEO.

☐ Confirm that you have deposited or provided access to graph files (e.g. BED files) for the called peaks.

Data access links<br>*May remain private before publication.*	*For "Initial submission" or "Revised version" documents, provide reviewer access links. For your "Final submission" document, provide a link to the deposited data.*
Files in database submission	*Provide a list of all files available in the database submission.*
Genome browser session<br>(e.g. UCSC)	*Provide a link to an anonymized genome browser session for "Initial submission" and "Revised version" documents only, to enable peer review. Write "no longer applicable" for "Final submission" documents.*

Methodology

Replicates	*Describe the experimental replicates, specifying number, type and replicate agreement.*
Sequencing depth	*Describe the sequencing depth for each experiment, providing the total number of reads, uniquely mapped reads, length of reads and whether they were paired- or single-end.*
Antibodies	*Describe the antibodies used for the ChIP-seq experiments; as applicable, provide supplier name, catalog number, clone name, and lot number.*
Peak calling parameters	*Specify the command line program and parameters used for read mapping and peak calling, including the ChIP, control and index files used.*

Data quality	*Describe the methods used to ensure data quality in full detail, including how many peaks are at FDR 5% and above 5-fold enrichment.*
Software	*Describe the software used to collect and analyze the ChIP-seq data. For custom code that has been deposited into a community repository, provide accession details.*

Flow Cytometry

Plots

Confirm that:

☒ The axis labels state the marker and fluorochrome used (e.g. CD4-FITC).

☒ The axis scales are clearly visible. Include numbers along axes only for bottom left plot of group (a 'group' is an analysis of identical markers).

☒ All plots are contour plots with outliers or pseudocolor plots.

☒ A numerical value for number of cells or percentage (with statistics) is provided.

Methodology

Sample preparation	Ventricles were digested as described in methods . The digested solution was filtered, rinsed in neutralization buffer, and then incubated in RBC lysis buffer.
Instrument	BD LSRFortessa, five-laser (355, 405, 488, 561, 640 nm); Penn Cytomics instrument "A3", serial R66093700193. FSC threshold 5,000. Nuclei for single-nucleus RNA-sequencing, and macrophages for the bulk RNA-sequencing validation, were sorted separately on a BD FACSAria III.
Software	Acquisition: BD FACSDiva v9.1. Gating and population statistics: FlowJo. Re-analysis of the exported gating workspaces, gate statistics and figure generation: R using flowWorkspace and ggcyto (CytoML import). Code: https://github.com/ikuznet1/RV_Atlas (helper_scripts/pab_flow).
Cell population abundance	Immunophenotyping was performed on unsorted ventricular digests, so no post-sort purity applies; all reported values are frequencies of the stated parent gate. For a representative Sham animal, cohort 1 yielded 3,588 CD45+ events of 1,000,000 recorded, of which 2,039 were CD64+ macrophages; cohort 2 yielded 37,537 of 6,062,444 recorded, of which 5,420 were CD64+ macrophages. Separately, for the resident-macrophage bulk RNA-sequencing, CD45+ CD11b+ CD64+ tdTomato+ cells were sorted on a BD FACSAria.
Gating strategy	Events were thresholded on FSC (5,000). Leukocytes were gated as CD45+ versus SSC-A on all recorded events, followed by an FSC-A/FSC-H singlet gate. Myeloid cells were gated as CD11b+ Ly-6G- (Ly-6G- excluding neutrophils); macrophages as CD64+ within the myeloid gate; resident (CCR2-) versus recruited (CCR2+) macrophages and their MHC-II status by a CCR2 x MHC-II quadrant gate on CD64+ cells; and TREM2+ macrophages within the macrophage gate. Positive/negative boundaries were placed on the internal negative population of each bivariate plot, with spectral spillover corrected using single-stain compensation controls acquired in the same run. Gates were drawn separately for each of the two staining cohorts and applied identically to every sample within a cohort; absolute cut-points therefore differ between cohorts, and all reported frequencies are normalized within experiment to that experiment's Sham median. The complete gating cascade for a representative animal from each cohort is provided in the Supplementary Information.

☒ Tick this box to confirm that a figure exemplifying the gating strategy is provided in the Supplementary Information.

Magnetic resonance imaging

Experimental design

Design type	*Indicate task or resting state; event-related or block design.*
Design specifications	*Specify the number of blocks, trials or experimental units per session and/or subject, and specify the length of each trial or block (if trials are blocked) and interval between trials.*
Behavioral performance measures	*State number and/or type of variables recorded (e.g. correct button press, response time) and what statistics were used to establish that the subjects were performing the task as expected (e.g. mean, range, and/or standard deviation across subjects).*

Acquisition

Imaging type(s) — *Specify: functional, structural, diffusion, perfusion.*

Field strength — *Specify in Tesla*

Sequence & imaging parameters — *Specify the pulse sequence type (gradient echo, spin echo, etc.), imaging type (EPI, spiral, etc.), field of view, matrix size, slice thickness, orientation and TE/TR/flip angle.*

Area of acquisition — *State whether a whole brain scan was used OR define the area of acquisition, describing how the region was determined.*

Diffusion MRI ☐ Used ☐ Not used

Preprocessing

Preprocessing software — *Provide detail on software version and revision number and on specific parameters (model/functions, brain extraction, segmentation, smoothing kernel size, etc.).*

Normalization — *If data were normalized/standardized, describe the approach(es): specify linear or non-linear and define image types used for transformation OR indicate that data were not normalized and explain rationale for lack of normalization.*

Normalization template — *Describe the template used for normalization/transformation, specifying subject space or group standardized space (e.g. original Talairach, MNI305, ICBM152) OR indicate that the data were not normalized.*

Noise and artifact removal — *Describe your procedure(s) for artifact and structured noise removal, specifying motion parameters, tissue signals and physiological signals (heart rate, respiration).*

Volume censoring — *Define your software and/or method and criteria for volume censoring, and state the extent of such censoring.*

Statistical modeling & inference

Model type and settings — *Specify type (mass univariate, multivariate, RSA, predictive, etc.) and describe essential details of the model at the first and second levels (e.g. fixed, random or mixed effects; drift or auto-correlation).*

Effect(s) tested — *Define precise effect in terms of the task or stimulus conditions instead of psychological concepts and indicate whether ANOVA or factorial designs were used.*

Specify type of analysis: ☐ Whole brain ☐ ROI-based ☐ Both

Statistic type for inference — *Specify voxel-wise or cluster-wise and report all relevant parameters for cluster-wise methods.*

(See Eklund et al. 2016)

Correction — *Describe the type of correction and how it is obtained for multiple comparisons (e.g. FWE, FDR, permutation or Monte Carlo).*

Models & analysis

n/a	Involved in the study
☐	☐ Functional and/or effective connectivity
☐	☐ Graph analysis
☐	☐ Multivariate modeling or predictive analysis

Functional and/or effective connectivity — *Report the measures of dependence used and the model details (e.g. Pearson correlation, partial correlation, mutual information).*

Graph analysis — *Report the dependent variable and connectivity measure, specifying weighted graph or binarized graph, subject- or group-level, and the global and/or node summaries used (e.g. clustering coefficient, efficiency, etc.).*

Multivariate modeling and predictive analysis — *Specify independent variables, features extraction and dimension reduction, model, training and evaluation metrics.*
