# Supplementary material for: Human adult and pediatric multimodal transcriptomic atlas of progression from pressure-loaded to failing right ventricle
Source: Nat Cardiovasc Res. 2026 Oct 9;5(11):1133–58. doi: 10.1038/s44161-026-00885-5 (PMC13753616; doi:10.1038/s44161-026-00885-5)
Supplement: Supplementary file 2 [file 44161_2026_885_MOESM2_ESM.pdf]

nature portfolio

Peer Review File

Human Adult and Pediatric Multimodal Transcriptomic Atlas of Progression from Pressure-Loaded to Failing Right Ventricle

Corresponding Author: Dr Zoltan Arany

This file contains all reviewer reports in order by version, followed by all author rebuttals in order by version.

Version 1:

Reviewer comments:

Reviewer #1

(Remarks to the Author)
Thank you for allowing me to review the manuscript by Kuznetsov et al in which the authors performed a deep transcriptomics based assessment of RV failure. The authors used bulkRNAseq, snRNAseq, and spatialomics to evaluate the molecular signature of RV failure. Overall, this is a much needed analysis but there are several areas that would be needed for improvement to make this a more valuable contribution, which I do believe it is.

1. I think for Figure 1, the authors should just perform deconvolution RNAseq rather than the module analysis that they did. The authors have the actual map of RV failure at the single nucleus level in FIgure 2, so why not? This would give a lot more data. I appreciate the limitations of deconvolution, but you can directly compare to your snRNAseq data. I appreciate the module system they employed, but it is a little more difficult to understand and not sure it really is more valuable.

2. The analysis of the spatial-omics data are relatively limited. Maybe that is because there were not as many differences. It may be useful to comment on the relative loss of CMs in snRNAseq that was not as drastic as observed in the spatial data.

3. The comparsion of RVF to LV failure is very nice. However, LV GDMT therapies don't work for RV failure so the authors should comment on this.

4. THe rodent data are not that useful. The authors only show RV dilation and no measures of dysfunction. There are plenty of data from rodents with pulmonary hypertension (bulkRNAseq, proteomics, metabolomics) showing mitochondrial derangements like the authors see in their transcriptomics data. Hard to say preclinical models are not that useful when these data exist. In the end, most RV failure in human HF is due to the presence of Group 2 PH. So a PH model is a better comparison.

5. There are human bulkRNAseq data from this journal looking at human PAH RV Failure. A discussion/comparison to those data would be useful. Again, PH drives RV failure in most left heart failure so this is a good comparison. In the manuscript, the authors show a very similar trend with mitochondrial derangements/inflammation/fibrosis.

6. The divergent mitochondrial response in pediatric and adult RVF is quite interesting. Do the authors have any tissue to evaluate mitochondrial respiratory in pediatric RVF.

Minor

Overall, the manuscript does not read that well to be honest. Maybe the authors are trying to put in too much data into one manuscript, but a theme is not well delineated.

(Remarks on code availability)

Reviewer #2

(Remarks to the Author)
Kuznetsov et al. combined a mixture of bulk and single-nucleus (sn) RNA-seq data in order to characterise the changes in gene expression that mark the transition from compensated to decompensated right heart failure (RHF). As the mechanisms of RHF appear to differ from those of the much better studied left heart failure (LHF) and treatment options are limited, identifying RHF-specific mechanisms is a relevant and widely interesting topic.
The authors investigated gene expression in transmural samples from the right ventricle of a large cohort of 142 patients, comprising non-failing controls (NF) and LHF patients with subsequent right heart disease in compensated (preserved, pRV) or failing (RVF) states, using bulk RNA-seq. To account for the expected heterogeneity of gene expression patterns across these samples and conditions, they employed weighted gene network correlation analysis (WGCNA) to identify gene regulatory modules that distinguish disease conditions. They then performed snRNA-seq on a smaller subset of 11 samples from these patients to determine changes in cell types, differentially expressed genes (DEGs) and cell states, followed by spatial transcriptomics on nine samples using the 10x Genomics platform. These data were then compared with snRNA-seq data from a paediatric patient cohort with congenital hypoplastic left heart syndrome (HLHS), as well as with a short-term mouse PAB model.
This tour de force results in the main and supplementary figures reporting a wealth of descriptive bioinformatics data sets that are difficult to follow, particularly for non-specialist readers. Furthermore, the underlying text is highly condensed and lacks necessary explanations of the approaches or rationales for data analysis. It also offers little insight or conclusions derived from the individual parts of the study.
Overall, this manuscript requires substantial rewriting and a reorganisation of the data analysis and presentation strategies. In my view, it would be much more meaningful to compare the extensive bulk RNA-seq analysis with existing RV gene expression studies, with the aim of integrating these data into consensus gene signatures that are characteristic of different types of RHF. These gene sets could then be tracked for their detailed cellular sources using the sn RNA-seq data sets with the aim to describe RHF cardiac niches.
Overall, this study does not meet the standards of a high-quality research paper, nor does it reach conclusions about interesting differentially expressed genes or cell types/cell states that go beyond current knowledge.
Specific points:
Fig. 1B: There appears to be little difference in gene expression between pRV and RVF. This is contrary to what is reported in the literature. Could this be due to patient treatment with multiple drugs (see Supplementary Table IV)? To what extent does the location of sample biopsies influence gene expression results? The authors need to provide a more detailed explanation of the patient cohort and consider confounding factors in their bioinformatics analysis.
Fig. 1C: The authors move straight away into WGCNA analysis without providing a general overview on the primary data. Therefore, it is very difficult to evaluate the quality of the data sets.
Fig. 1E: Gene module M29 is missing.
Fig. 2: A general overview on data quality (mapped reads, average number of genes / cell, srublet score number) is missing.

Fig. 2B: Although RYR2 expression in CM is very high, this gene is also expressed in all other cells. It would be more appropriate to choose a marker with higher and more exclusive expression in CM.
Fig. 2C: The decrease in cardiomyocytes in pRV and RHF is not novel and is also found in LHF. The authors should discuss this literature appropriately (DOI: 10.1038/s41586-022-04817-8).
Fig. 3: The spatial transcriptomic data is difficult to follow. They appear to be integrated from all donors? A much more detailed explanation and visualization is required.
Fig. 4E: There seems to be not much difference in the regulation of four genes in pRV and RF compared with controls. The authors should provide statistical results.
Fig. 5: How do the differences between RV and LV compare to similar comparisons from published studies (DOI: 10.1038/s44161-022-00113-w, DOI: 10.1038/s44161-024-00485-1, DOI: 10.1161/CIRCULATIONAHA.119.045401)
Fig. 6: Other studies have also reported an inflammatory component and an altered immune cell compartment. In my view, the interpretation of the glucocorticoid receptor target gene sets is an oversimplification and incorrect. An activated glucocorticoid receptor can bind to either a positive or negative glucocorticoid response element (GRE) in the genome, thereby inducing or repressing gene expression. Therefore, downregulation of genes with a proven or predicted GRE is not evidence of downregulation of the glucocorticoid system (see DOI: 10.1136/ard-2023-225371 for a recent review on this topic).
Fig. 8: The rationale for including the pediatric samples is unclear and there is no firm conclusion derived from the combined analysis.
Fig. 8D: Coefficients of correlation are missing
Text:
Introduction: Key literature on RH disease is missing (e.g. DOI: 10.1056/NEJMra2207410)
Results:
Page 4, lines 146 – 148: Where in Fig 3E are the known marker genes, spatial niches and cell types are located? This observation needs to be described in much more detail in the Results and Legends.
Page 4, lines 155-156: What does this mean: …Cross referencing these clusters with spatial niches demonstrated spatial pattering (Fig. S2G)?
Page 4, line 166: What are “nuclear synthesized genes”?
Page 5, line 228-229: What is the contribution of patient treatments to the M1 set of glucocorticoid-regulated genes? What is a pseudo-steroid deficient state?
Page 7, lines 308-310: What is the evidence to suggest that the mild RVF group may be protected?
Page 10, line 429: …, we also show upregulation…(missing word)
Page 10, line 434: What are pseudo-steroid resistant CCR2- rMacs?

Page 11, lines 487-490: This is a meaningless and trivial conclusion.

(Remarks on code availability)

Reviewer #3

(Remarks to the Author)
In their work, Kuznetsov et al. provide a transcriptomics atlas of right ventricular failure (RVF) progression in adults on the bulk, single-nucleus, and spatially resolved cellular levels. They identify several prominent changes, including a decrease in nuclear-encoded mitochondrial transcript expression in cardiomyocytes, increased expression of transcripts related to antigen presentation in myeloid cells, and an expansion of the endothelial cell compartment in the diseased samples. They compare their single-nucleus data to a mouse model of RVF, pediatric HLHS, and left ventricular failure (LVF), and provide a detailed landscape of shared and distinct characteristics between these conditions on the transcriptome level.

The manuscript presents a rich and multifaceted dataset, generated using multiple technological platforms and derived from complementary sample types, addressing a disease condition that has been understudied at the molecular level. As such, the data and analyses presented are highly relevant and are likely to attract interest within the cardiovascular field. The analysis is thorough, and the conclusions are, for the most part, well-supported by the accompanying figures and tables. Nonetheless, I have several general observations and comments (detailed below) that I believe should be addressed by the authors.

Overall, the authors were quite cautious in contextualizing their findings and in proposing hypotheses to explain the observed transcriptomic differences between disease stages or experimental models. While it is understandable that the study aims to serve as a foundation for future hypothesis generation, the inclusion of some author-driven interpretations or speculative hypotheses in the discussion would help better illustrate the study’s potential and guide subsequent research.

Additionally, while the analyses of the bulk and single-nucleus datasets are clearly described and well-integrated into the study, the spatial transcriptomics dataset (Xenium) appears underutilized. Its contribution to the central findings of the study remains unclear, as it is neither mentioned in the abstract nor acknowledged in the author contributions. Given the unique insights spatial transcriptomics can offer, I strongly encourage the authors to expand on this aspect of the work and provide a more in-depth assessment of spatial cell arrangements and interactions within the analyzed tissues, and their potential relevance to RVF progression.

The code is well organised overall and would be easy to reproduce with access to the study data. The authors used state-of-the-art R packages/methods for most of the analysis, with some great additions. The scripts being separated per figure and per panel make it very easy to follow through the manuscript. Some comments to allow for even better reproducibility are also provided below.

Major comments:

1. Please include the number of nuclei represented in all UMAPs, both within the figures and their corresponding legends. If the UMAPs display data from different experimental groups, provide versions of the plots with embeddings that highlight these groupings (as shown in Fig. S8A), to illustrate the effectiveness of data integration and to reveal any sample- or group-specific patterns.

2. Please consider providing dot plots of differentially expressed genes (DEGs) between the nuclear subclusters along with or instead of the currently included marker score enrichment. The former would be considerably more informative and easier to interpret than the complete DEG lists included in the manuscript, particularly for cell states that do not fall into biologically well-defined categories, such as cardiomyocyte subclusters. For endothelial and myeloid subclusters, please also include plots of key marker gene expression to support the provided annotations. Additionally, please expand the methods part of the manuscript with describing the annotation strategy of single-nucleus and Xenium-based cellular clusters.

3. In lines 90–92, the authors state that the majority of transcriptomic changes occur during the NF-to-pRV transition, with considerably fewer changes observed in the subsequent pRV-to-RVF transition. This suggests that many of the molecular alterations may be independent of RV functional decline. Given this, it remains unclear how the pronounced functional deterioration that characterizes the pRV-to-RVF transition is mediated at the molecular level. Do the authors have any insights or hypotheses regarding this?

4. How do the authors interpret the differentially expressed genes identified between pRV and RVF in the bulk RNA-seq data, as described in lines 101–108? Notably, SLITRK5 and GRIN2B are typically enriched in neuronal cell types - does this imply a change in the abundance of such cells between the two conditions? I would also question the relevance of SLITRK5 expression in AV node cells, as mentioned by the authors, in the context of this analysis.

5. Do the transcriptomic changes in genome-encoded mitochondrial components observed between NF, pRV, and RVF samples reflect alterations solely in mitochondrial function, or do they also impact mitochondrial abundance? Could the authors suggest experimental approaches to investigate this? This question is also relevant to the pediatric data, where ‘mitochondrial expansion’ is mentioned (line 386).

6. Regarding the Xenium analysis, were the tissue samples sectioned in a consistent orientation (e.g., similar sectioning planes of elongated cardiomyocytes)? If not, how reliable is the comparison between these datasets and different conditions? In Fig. 3C, there appears to be a striking difference in the density of annotated niches between the NF and RVF samples - could this disparity be attributed to biological variation or technical factors? Additionally, the niche nomenclature appears inconsistent; in some cases, it is based on the dominant cell type (which can be misleading as it might be mistaken for spatial single-cell clusters), while in others it refers to macroscopic tissue structures. This should be standardized or further clarified. It could also be helpful if the authors included a dot plot or a similar visualization showing the relative enrichment of different major cell types within the identified niches.

7. What filtering steps were included for the primary Xenium data and the segmented cell before cell label assignment? How were the spatial niches defined? Have the authors considered performing spatial gene imputation from the corresponding single-nucleus RNA-seq data for more in-depth, spatially informed analysis (e.g. ligand-receptor interactions)?

8. In Fig. 3B, please include the endocardial cluster as well. More generally, adding anatomical landmarks, such as the endocardium, and possibly a scale bar to the Xenium images would greatly aid their interpretation.

9. TMEM65, referenced in line 168, has also been reported to localize to the mitochondrial inner membrane (Nishimura et al., PeerJ, 2014). This subcellular localization is likely more pertinent to the mitochondrial patterns discussed in this paragraph than its alternative localization to the intercalated disc, mentioned by the authors.

10. The authors describe a pseudo-steroid deficient state in RVF monocytes, among other cell types. Do they suggest that these blood cells acquire this state after infiltrating the failing heart tissue, or could this reflect a systemic immunomodulatory effect associated with RVF?

11. Was the observed fibroblast-to-myofibroblast transition accompanied by fibrosis at the microscopic level in the pRV and RVF samples? Has this been previously documented in the literature, or could it be further explored in this study using specific histological stains, such as Masson's trichrome?

12. I could not find any information on the number of nuclei analyzed in the mouse and the pediatric single-nucleus datasets. Also, how many mice were used for the single-nucleus RNA-seq experiments?

13. Could the authors clarify what is meant in line 310 by describing the mild RVF group in the mouse model as 'protected' based on the single-cell data? In lines 484–490 of the discussion, they note important differences between the disease progression in the mouse RVF model and human pathology. From this, one might infer that the mild RVF group exhibits more adaptive tissue responses to the PAB insult compared to the severe group. It would be helpful if the authors could elaborate on this point in the main text, specifically in relation to the observations from the different mouse experimental groups.

14. In lines 349–350, the authors report induction of several cardiomyocyte-specific transcripts in myeloid cells in both pediatric and adult RVF samples. This finding is more likely attributable to contamination, such as ambient RNA (despite the authors' efforts to address ambient RNA cleanup as described in the methods) rather than reflecting a true biological phenomenon. Please review this observation and consider revising the statement accordingly.

15. In Fig. 5A and 5C, the enrichment pattern of module M2 appears to be opposite when comparing DCM and donor (control) samples. Could the authors clarify the difference between these two plots? It would be helpful to revise the figure legend to make this distinction more explicit.

16. In the description of the clustering analysis, please include the specific parameters used (e.g., resolution, number of principal components, nearest neighbors).

17. Please provide more detailed technical information on how the trajectory analysis was performed.

Minor comments:

18. Please provide a brief description of the Supplementary Data files for easier navigation.

19. In the abstract (lines 35-36), the authors state that the expansion of the endothelial cell compartment is unique to RVF compared to LVF, but I could not find any specific comment on this discrepancy within the main text. It would be helpful if the authors could clarify the basis for this claim and, if possible, offer any hypotheses to explain this observed discrepancy.

20. I recommend switching panels Fig. 1F–G with Fig. S1E–F between the main and supplementary figures. The latter, which illustrates gradual changes along the course of disease progression, appears more central to the study's main message. In contrast, the former, depicting a peculiar pattern for a gene module related to antigen presentation, is not thoroughly explained or contextualized and thus seems less critical.

21. There appears to be no legend provided for Fig. S3C. Please add a corresponding legend to facilitate interpretation of the panel.

22. Please add a statement to the ethics part of the methods on how the sensitive, poly(A)-capture-based sequencing data is handled.

23. In line 991, I assume the authors meant RV (and not LV) samples.

Comments on the code:

24. The authors did not share information about the package versions for reproducibility. Please consider adding at least the output of sessionInfo(). For such an atlas paper, which most likely will be reused by other scientists, adding environment infos such as a renv.lock file with renv package or a Docker container would be very useful for reuse of the data and further exploration of the dataset.

25. It seems like the current GitHub is not presenting the script for processing the snRNA-seq data. Please consider adding the processing scripts with loading the raw files, or 10x cellranger outputs (or similar) as well as filtering, doublets handling, normalisation and batch correction.

26. Some files are read using readRDS() such as scWGCNA_bulk2sn_projection.rds or bulk_heart_modules.csv. Please consider adding clearly where these files/objects come from and if generated by you, add the script for it (unless I missed it).

27. There are several mismatches regarding the numbering of figure panels between the scripts and the manuscript. Please consider updating the numbering in the scripts for future readers to avoid confusion.

28. Where some extra code is added, please make sure to add a title to specify that these parts of scripts are not used to generate figures.

(Remarks on code availability)
Please see my comments on the code above. The authors did not provide a proper README file, and in lack of the study data it was not possible to assess if it is possible to recreate the plots included in the manuscript. The evaluation is therefore based on reading the scripts, which overall appear well-organized and apart from certain parts related to data processing and generating the objects used for later analysis steps (see above), thorough.

Reviewer #4

(Remarks to the Author)
In this manuscript by Kuznetsov et al. the authors use a variety of transcriptomic techniques to probe the landscape of adult and pediatric human and mouse right ventricles that are healthy, pressure-loaded or failing. The authors do this to compare and contrast differences with age, species and disease of the right ventricle, which is grossly understudied compared to the left ventricle. It is an interesting cohort that is generally well-probed and comprehensive. As the authors point out this study is descriptive, but has the potential to fuel more mechanistic and hypothesis-driven studies.

This reviewer is not an expert in assessing the quality or validity of the extensive RNA-seq analyses, but can understand their interpretation, which generally seems founded. Other reviewers will undoubtedly provide more stringent review and assessment of the structural cell biology. However, this reviewer does have experience in cardiovascular immunology, preclinical models and macrophage biology. Although many observations are logical and intriguing, some are perplexing and require greater insight before publication can be considered in Nature Cardiovascular Research.

1. Greater methodological details of the pulmonary arterial banding model need to be provided. Although it is a common model, there is variation in the severity of its induction, which vary from lab to lab or experiment to experiment. Moreover, the authors should provide more functional and immunological data of this model, particularly due to the fact that much of the model seemingly does not recapitulate human disease. Although the model is well-described in the literature, it needs to be shown here so conclusions or hypotheses as to why their is discordance between mice and humans can be made. There is some description of this in the discussion, but more data are needed. At 2 weeks, what is the right ventricle ejection fraction of these mice, as well as other metrics such as diameters and volumes and blood pressure? By flow cytometry the inflammatory landscape of the heart should be compared to sham controls with particular emphasis on the macrophage subpopulations using CCR2, TREM2, MHC-II etc. Additionally, histology for cardiomyocyte hypertrophy and fibrosis could be used to compare the pathology of disease in mice and humans.

Why is expression of CCR2 low in this dataset, as the others point out in line 201? It is frequently used in both mouse and human studies at the transcript and protein level for segregating macrophage and monocyte populations. How does it compare to other published datasets?

It is unclear why the authors used CX3CR1-cre Rosa26-TdTomato mice in these experiments. In theory, all monocyte/macrophage lineage cells will be red following tamoxifen injection and thus it provides no more insight than the usage of a sorting CX3CR1+ cells for bulk seq. Caution should be used therefore in referring to the bulk seq as resident macrophages, particularly because no flow or sorting data are provided. It is also not clear how this is intended to "orthogonally validate" the discordance.

The authors make very interesting observations pertaining to gluco and mineralcorticoid receptors in humans with RVF.

However, this signature is not seen in mice. If the authors performed PAB in NR3C1-deficient mice, would the heart be protected or worsened?

2. The authors extensively rely on CCR2 for their subpopulations of macrophages. Generally, the conserved TLF (TIMD4, LYVE1 and FOLR2) signature can be used for resident embryonic macrophages across species and tissues (Dick...Epelman 2022 Sci Immunology;). These are specifically known to be conserved in humans and across most tissues. The authors mention these genes in the discussion and that they are perhaps expressed in a CCR2+ population which is highly unexpected. Can the authors use their dataset and compare it to those already published to ascertain the existence of this population and more completely describe its dynamics, location and transcriptional landscape during health and disease, aging, and in mice (PAB) versus humans (RVF). If RVF is uniquely changing the resident embryonic population to a more inflammatory subset in the absence of monocyte input that would have very interesting ramifications that would shape our understanding of ontogeny and macrophage heterogeneity and plasticity of responses. The analyses can also incorporate the authors' observations regarding MHC-II and what role they may be playing in heart failure, which is not completely delineated.

(Remarks on code availability)

Reviewer #5

(Remarks to the Author)
This study by Kuznetsov et al provides an important framework for understanding human right ventricular failure (RVF), integrating bulk, single-nucleus, and spatial transcriptomic approaches. In addition to its insights into human RVF, the study includes important comparative analyses of RV from pediatric single ventricle patients and from the mouse pulmonary artery banding (PAB) model of pressure overload-induced RVF. One of the most intriguing findings is the opposing regulation of mitochondrial gene programs where expression of nuclear encoded mitochondrial genes were found to be suppressed in adult RVF, but induced in both the PAB and pediatric samples. This raises questions about how age-, model-, or even species-specific differences might impact the expression of mitochondrial genes and mitochondrial function. One potential interpretation of the data is the unexpected possibility that mitochondrial function may actually be enhanced in the setting of pediatric single ventricle physiology or early-stage PAB. Given that the authors have established a assay to assess mitochondrial functon(respiration) using banked tissue, additional discussion of how transcriptional changes relate to mitochondrial function, and extending these functional assessments to at least the PAB model, would strengthen the conclusions and improve the overall completeness of an already exciting study.
Concerns:
1. The respirometry data provide important functional insights into mitochondrial function in RVF. The authors state that complex I+II-supported respiration is impaired in RVF. However, this may be a misinterpretation. Oxygen consumption with complex I substrates alone is not significantly reduced compared to NF controls, whereas respiration supported by complex II substrates, or combined complex I+II substrates, is significantly reduced. This pattern suggests that the primary functional deficits may lie downstream of complex II (possibly complex II, III or IV). Given that complex IV activity is impaired (based on TMPD/ascorbate-driven respiration), it is unclear from respirometry alone if RVF causes primary defects to complex II and III. More importantly, do the transcriptomic data, and particularly changes in expression of nuclear-encoded mitochondrial genes involved in respiratory chain/electron transfer support the functional data measured by respirometry (for example, are there changes to complex IV subunits or assembly factors?)? Additionally, does RVF lead to reduced respiratory chain complex assembly with particular complexes preferentially affected? Correlating transcriptional changes with functional data would help strengthen the mechanisms.
2. As noted above, the finding that mitochondrial gene expression is upregulated in PAB and pediatric HLHS RV samples was a surprising and really intriguing finding from the study. Given that depression of mitochondrial genes was associated with reduced respiration in adult RVF samples, is mitochondrial function enhanced in response to PAB and in the RV of pediatric single ventricle disease? Are there transcription factors regulating mitochondrial gene expression that are engaged in the PAB/pediatric single ventricle hearts that are suppressed in adult RVF?
3. I understand the rationale for looking at the 2 weeks post-PAB time point, but could this early timepoint reflect a compensatory period where mitochondrial function might paradoxically be maintained? Analysis of an expanded time course (perhaps more fulminant failure) correlating gene expression with mitochondrial function might be warranted as a more complete comparison with adult human RVF.

(Remarks on code availability)

Version 2:

Reviewer comments:

Reviewer #1

(Remarks to the Author)

I appreciate the opportunity to evaluate the revised version of the article entitled "Human Adult and Pediatric Single-Nucleus Transcriptomic Atlas of Progression from Pressure Loaded to Failing Right Ventricle." The authors did a very nice job and good faith effort to address my comments. In the end, I think the revised version is much better. I think this is a much needed addition to the literature and provides additional insight into RV biology. After this much work and effort, I think it is unfair to ask for additional work and think this is great. Congratulations.

(Remarks on code availability)

Reviewer #2

(Remarks to the Author)
The authors have carefully and thoroughly addressed all concerns raised and have substantially revised their manuscript. These changes have significantly improved both the data presentation and the discussion of relevant conclusions. I have no further comments, except for one incorrect citation: The statements in the discussion (lines 684–689) refer to reference 57 (Jurida et al., doi: 10.1038/s44161-024-00485-1) rather than to the paper by Boucherat et al. (ref. 58, doi: 10.1038/s44161-022-00113-w), which is discussed later, in lines 699–701.

(Remarks on code availability)

Reviewer #3

(Remarks to the Author)
I would like to thank the authors for the extensive amount of work they put into revising their manuscript. In general, they addressed all my specific concerns, and I believe that the new analyses and a more focused discussion of some of the biological observations substantially improve the work. I was also glad to see a more meaningful utilization of the Xenium dataset, and a more detailed description of the bioinformatic analysis. Also regarding the code, the authors did a good job in providing a more transparent and reproducible framework.

Below, I have some additional minor suggestions, more related to the format than the content, that do not affect my overall positive evaluation of the revised manuscript.

1. Since the manuscript integrates multiple datasets, the title could perhaps be revised from single-nucleus atlas to better reflect its broader scope.

2. Seemingly, there is a contradiction between the gradually increasing extent of histology-level fibrosis (Fig. S3M), the gain of ECM signalling (Fig. 4E), and the decreasing proportion of the fibrotic myocardial niche (Fig. 3G) along the NF->pRV->RVF axis. While this might be explained by shifts of histologically fibrotic or fibrosis-adjacent areas between spatially closely related niches, it would still make sense to add a comment to the related sentence in lines 276-279 to clarify this.

3. Please make sure that all abbreviations, including CTEPH and GDMT, are expanded at their first use in the manuscript, to help non-specialist readers.

4. In Fig. 3A, the UMAP presents data from segmented cells, not nuclei - please correct the in-panel label.

5. In Fig. 8A, it is not possible the separate the HLHS and adult RV datasets, and the in-panel n= text only includes the HLHS data. Please add an in-lay panel coloured by sample type to show successful integration of the two sample types, and include the number of RV numbers in the in-panel n= text.

6. While the restructuring of the manuscript has improved its overall narrative flow, the text has also become quite dense. As a result, it is sometimes difficult to determine which dataset specific statements refer to - for example, whether the subclustering of certain cell populations was performed on the snRNA-seq or Xenium data. Please make sure this is as clear as possible throughout the manuscript.

I would like to thank the authors again for the opportunity to evaluate their work.

(Remarks on code availability)
The updated code provides good structure, a clear README file, and enough information to enable reproducing the analysis, even though due to time restrictions, we did not re-run the analysis ourselves.

Reviewer #4

(Remarks to the Author)
This reviewer appreciates and acknowledges the extent of the appropriate revisions. I advise to accept.

(Remarks on code availability)

Reviewer #5

(Remarks to the Author)

(Remarks on code availability)

Open Access This Peer Review File is licensed under a Creative Commons Attribution 4.0 International License, which permits use, sharing, adaptation, distribution and reproduction in any medium or format, as long as you give appropriate credit to the original author(s) and the source, provide a link to the Creative Commons license, and indicate if changes were made.
In cases where reviewers are anonymous, credit should be given to 'Anonymous Referee' and the source.
The images or other third party material in this Peer Review File are included in the article's Creative Commons license, unless indicated otherwise in a credit line to the material. If material is not included in the article's Creative Commons license and your intended use is not permitted by statutory regulation or exceeds the permitted use, you will need to obtain permission directly from the copyright holder.
To view a copy of this license, visit https://creativecommons.org/licenses/by/4.0/

RV Paper, Reviewer Comments & Responses

EDITORIAL COMMENTS

1. Please include analysis of mitochondrial function in the PAB mouse model and in pediatric samples (R#1, R#3, and R#5).

We thank the reviewers and editors for this important suggestion. In response, we have now expanded our study to include direct functional assessment of mitochondrial respiration in both the murine pulmonary artery banding (PAB) model and pediatric RV samples (new **Figure 5H-J**).

Notably, we want to be transparent about an important correction prompted by reanalysis of the data. In our initial analysis, the apparent upregulation of mitochondrial transcripts in the PAB and pediatric RVs was an artifact of aggregating signal across pooled nuclei rather than computing per-sample (pseudobulk) summaries. When we re-ran the analysis with per-animal and per-patient pseudobulk aggregation and gene-set enrichment (the same framework applied to the adult cohort) the apparent induction did not hold: the direction reversed in PAB and attenuated to non-significance in pediatric (**Figure S6E-G; Figure 8F**). We have corrected this throughout the revised manuscript, and the corrected result changes our answer.

Using respirometry, we evaluated mitochondrial electron transport capacity in RV tissue from PAB mice. Similar to our observations in adult human RVF samples, we observed a reduction in mitochondrial respiratory capacity following PAB. These findings indicate that mitochondrial functional impairment is present in this pressure-overload model.

We also performed mitochondrial respirometry on pediatric RV samples (n=7 ped-NF vs n=9 ped-HLHS-palliated failing). In contrast to the adult RVF and murine PAB cohorts, pediatric HLHS-palliated failing RVs showed no significant deficit at any respiratory complex. Pediatric single-ventricle failure thus appears functionally distinct from adult acquired RVF, consistent with the developmental adaptive capacity of the pediatric heart to long-standing single-ventricle physiology.

Interestingly, these functional findings reveal a pattern across cohorts. Adult RVF cardiomyocytes show coordinated downregulation of nuclear-encoded mitochondrial transcripts paralleled by reduced respiratory capacity (transcripts and function both fall). Murine PAB cardiomyocytes show the a similar, but less pronounced transcriptional direction (mitochondrial transcripts supressed) and respiration is also impaired. Pediatric HLHS-palliated failing RVs show no differential transcriptional changes in mitochondrial transcripts together with preserved respiratory capacity.

These data show that mitochondrial respiratory dysfunction is a feature of adult and PAB-driven pressure-overload RVF but is notably absent in pediatric HLHS-palliated failing RVs. Across cohorts, mitochondrial transcriptional state and respiratory function move together, both reduced in adult and murine pressure overload and both spared in pediatric

single-ventricle RVF, with the key divergence falling between cohorts rather than between transcription and function.

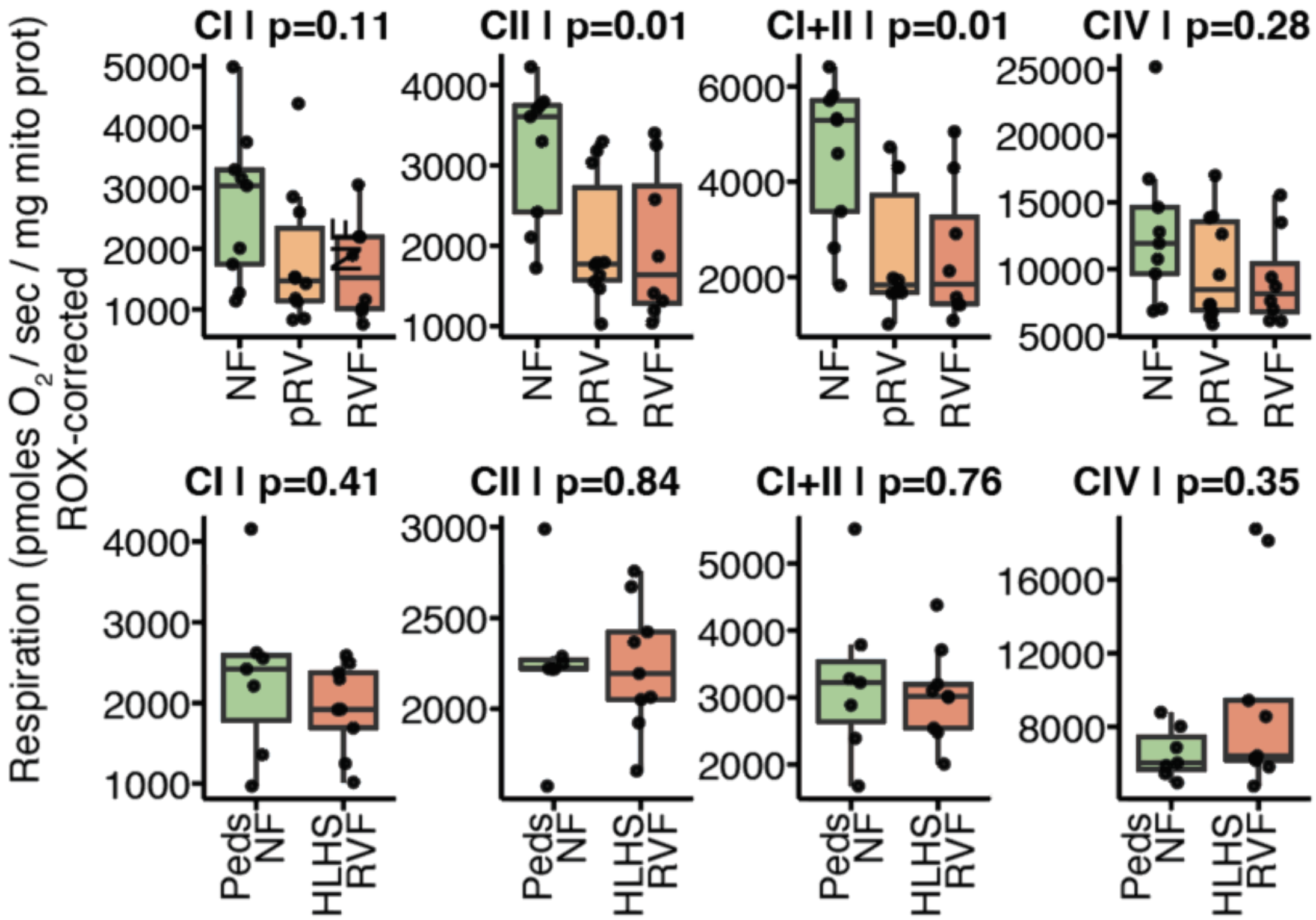

Figure 5H: Adult and pediatric respirometry. Oroboros 2k high-resolution respirometry on freeze/thaw-isolated RV mitochondria, ROX-corrected and normalized to starting mitochondrial protein mass (pmoles O_2 /sec/mg mitochondrial protein). Each dot is one patient. Adult cohort **(top)**: NF n=9, pRV n=10, RVF n=8; Kruskal–Wallis p-values shown. Pediatric cohort **(bottom)**: pediatric NF (n=7) vs HLHS-palliated failing (n=9); Mann–Whitney pairwise p-values.

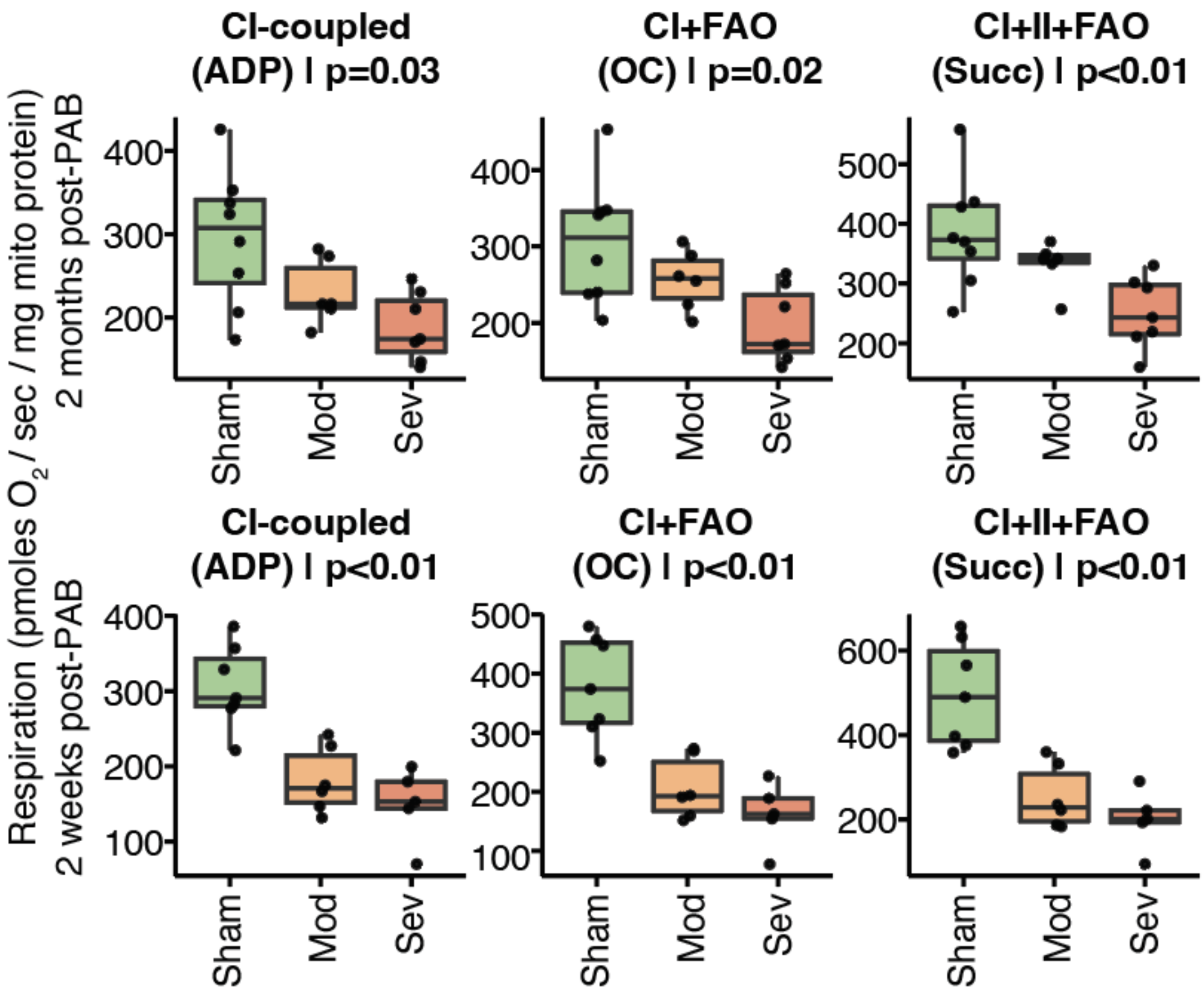

Figure 5I: PAB respirometry. Respirometry in PAB model at 2-month **(top)** and 2-week **(bottom)** time points vs sham, using a substrate-addition protocol (PM → ADP → cyt c → octanoyl-carnitine → succinate) on tissue homogenate. CI+II+FAO maximal coupled respiration (Succ-driven) is the headline measure. 2-week: Sham n=8, Mild n=6, Severe n=7. 2-month: Sham n=7, Mild n=6, Severe n=5. Each dot is one animal; Kruskal–Wallis p-values shown.

J

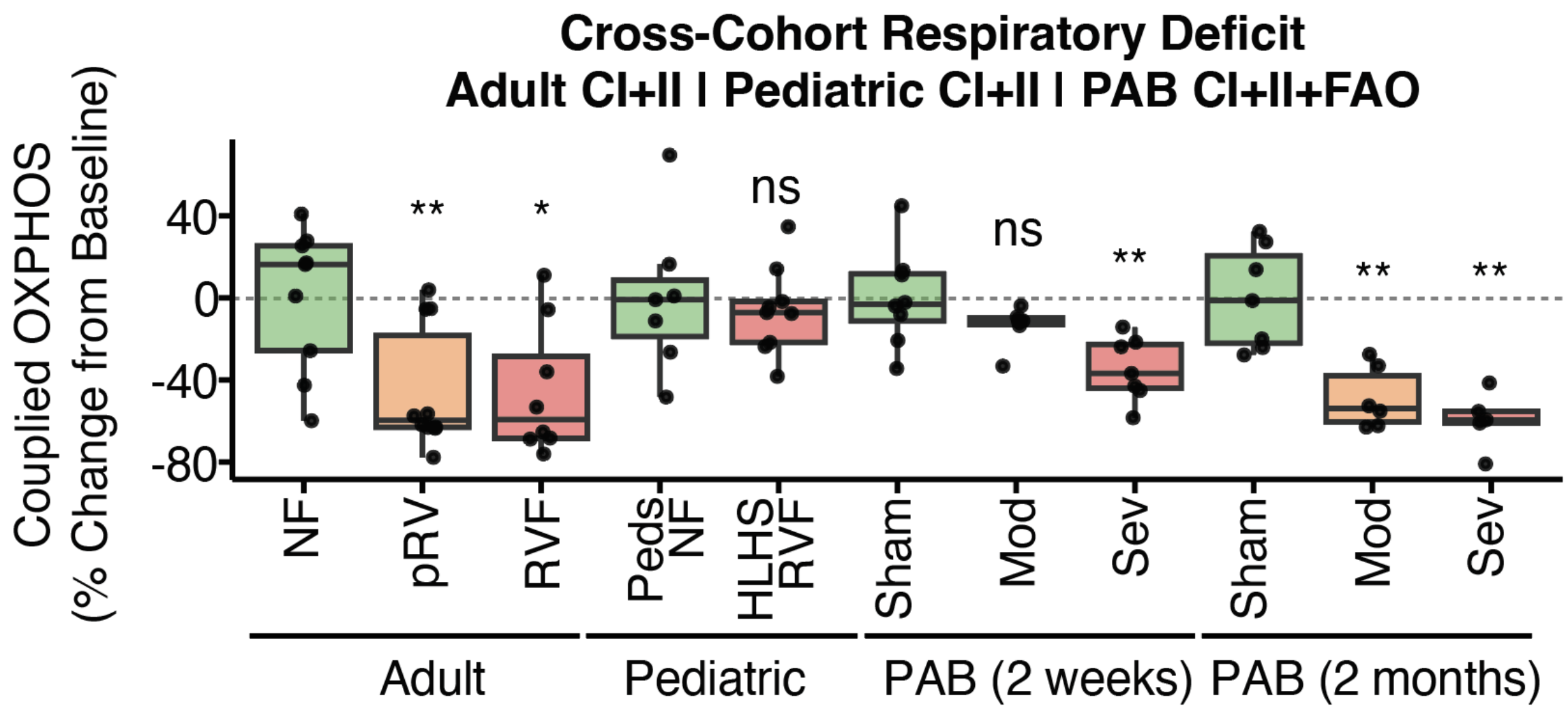

Figure 5J: Cross-cohort respirometry comparison. Cross-cohort summary of RV mitochondrial OXPHOS, normalized to within-cohort controls. Maximal coupled respiration is reduced in adult RVF and murine PAB but preserved in pediatric HLHS-palliated failing RV.

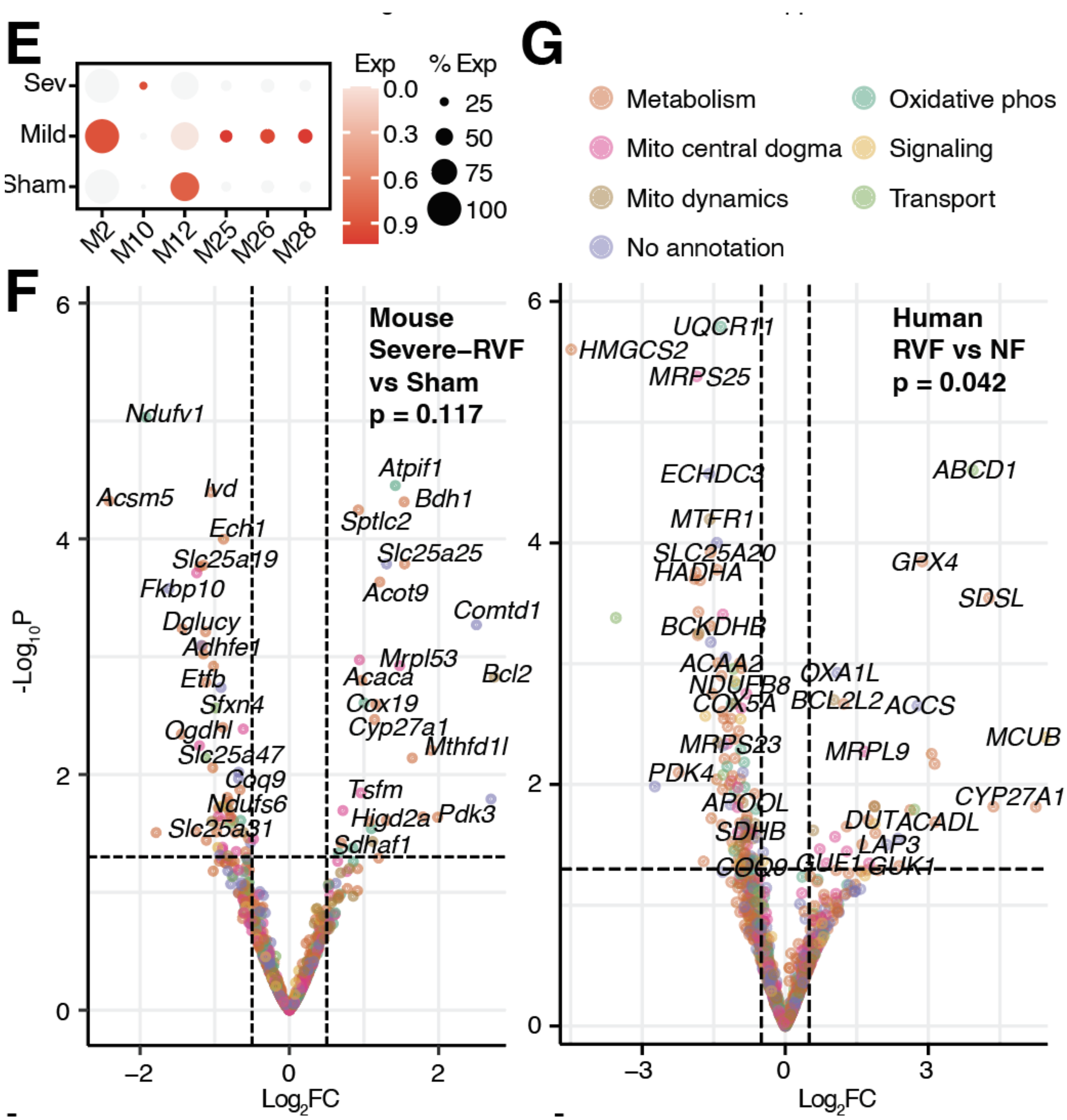

Figure S6E-G. Murine and human mitochondrial transcripts in RVF. (E) Dot plot of normalized *Seurat*-calculated expression score of corresponding WGCNA module genes in the mouse PAB cardiomyocytes by disease state. Previously observed trends in the human dataset are poorly conserved. **(F)** Per-mouse pseudobulk DESeq2 volcano of MitoCarta3.0 genes in murine RV CMs, severe-RVF vs NF (n = 4 vs 3) with apeglm log_2 fold-change shrinkage; points colored by MitoCarta3.0 top-level pathway. Per-mouse Wilcoxon p-value is shown for the NF vs (mild-RVF and severe-RVF) comparison. **(G)** Per-patient pseudobulk DESeq2 volcano of MitoCarta3.0 genes in human RV CMs, RVF vs NF (n = 3 vs 4) with apeglm log_2 fold-change shrinkage; points coloured by MitoCarta3.0 top-level pathway. Per-subject Wilcoxon p-value is shown for the NF vs (pRV and RVF) comparison.

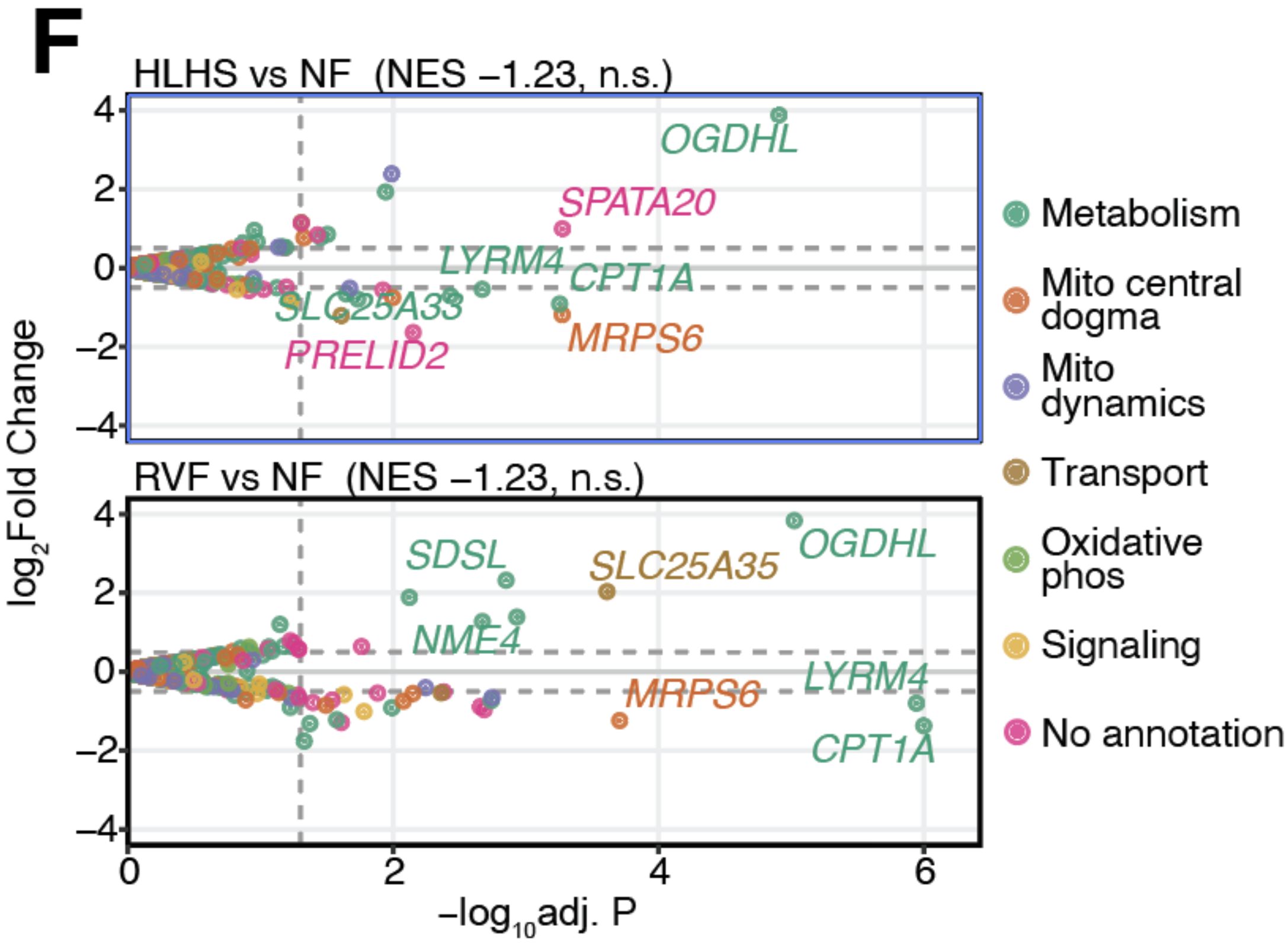

Figure 8F. Pediatric mitochondrial transcripts. MitoCarta3.0 gene-level volcano plots for pediatric cardiomyocytes: ped-HLHS (**top**) and ped-RVF (**bottom**) versus ped-NF. Per-patient pseudobulk DESeq2 (apeglm log_2FC shrinkage, FDR from the unshrunken Wald test; n = 4 ped-NF, 5 ped-HLHS, 5 ped-RVF). Each point is a MitoCarta3.0 gene colored by top-level MitoPathway category. Gene-set enrichment over the full ranking: ped-HLHS normalized enrichment score (NES) = −1.23, ped-RVF NES = −1.23. The shift is not significant at the patient level.

Changes in manuscript:

- Added mitochondrial respirometry across all three cohorts in new **Figure 5H–J**. **Figure 5H** (adult and pediatric RV respirometry), **Figure 5I** (murine PAB respirometry), and **Figure 5J** (cross-cohort summary across adult RVF, murine PAB, and pediatric HLHS), with corresponding text in the Results.
- Corrected the mitochondrial transcript analysis to per-sample (pseudobulk) aggregation with gene-set enrichment, and revised the Results, Discussion, and Abstract. Added **Figure S6F-G** and **Figure 8F** accordingly: murine PAB cardiomyocyte mitochondrial transcripts are now reported as coordinately suppressed (concordant with adult RVF), and pediatric transcripts as a minimal, non-significant change, replacing the earlier description of induction.

2. Please follow R#4 advice to expand and validate the findings regarding the transition of resident embryonic population to a more inflammatory state and the upregulation of MHCII-associated genes.

In response, we expanded our analyses and revised the interpretation of these results to more carefully address macrophage identity, activation state, and spatial context (**see Figure 6**).

First, we expanded the characterization of cardiac macrophage populations using established markers of resident macrophage identity. In addition to CCR2-based classification, we examined canonical resident macrophage markers including TIMD4, LYVE1, and FOLR2, which have been reported to identify embryonically derived tissue-resident macrophages across multiple tissues (**Figure 6B**).

Second, we further examined transcriptional programs associated with inflammatory macrophages and antigen presentation. Differential expression analysis and transcription factor enrichment consistently identified induction of MHCII-associated genes and CIITA-regulated transcriptional programs in RVF myeloid populations, supporting an increased antigen-presenting phenotype in the diseased RV (**Figure 6E, G-H**).

Third, we expanded the spatial analysis of macrophage populations using the Xenium dataset (**Figure 6I**). Inflammatory macrophages colocalized with monocytes at the tissue periphery, a spatial pattern that does not by itself distinguish phenotypic transition of resident macrophages from monocyte-derived recruitment; we now describe this finding cautiously and discuss its implications for the resident-vs-recruited question in the manuscript (**Figure 6I, S3L**).

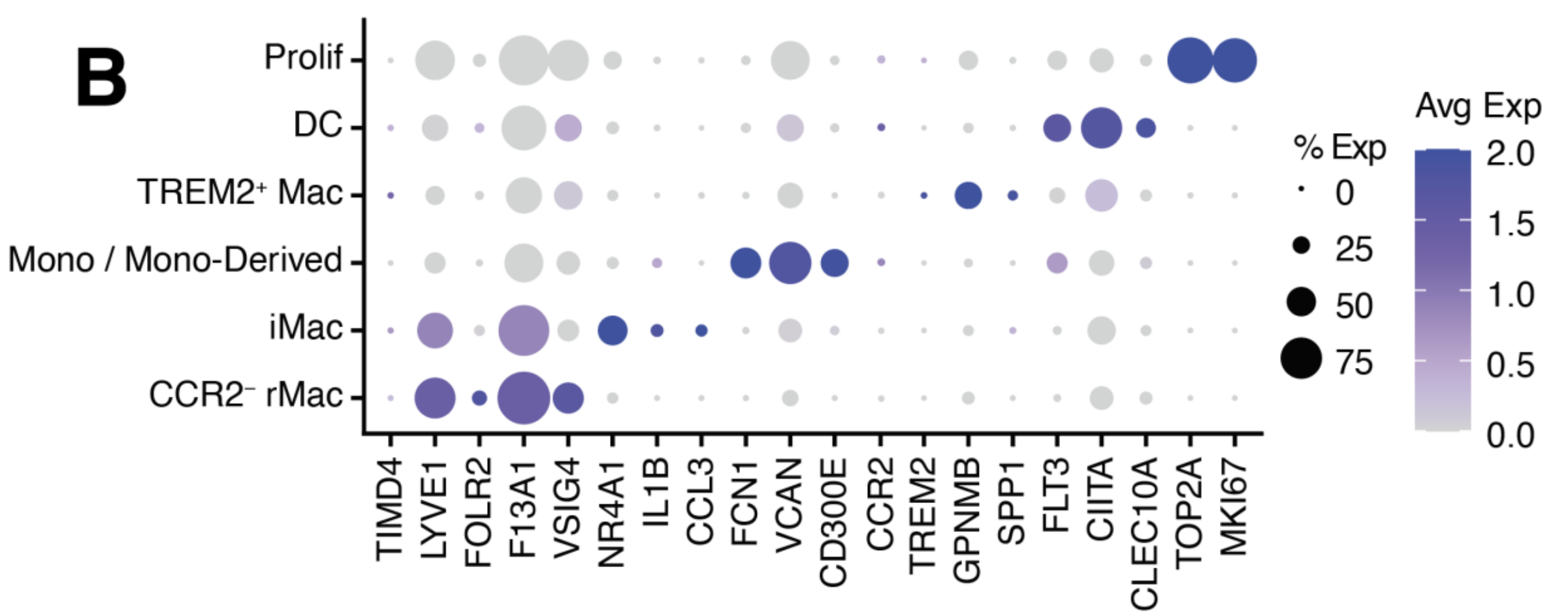

Figure 6B: Myeloid subtype marker genes. Dot plot of canonical marker genes per myeloid subtype. Mean expression (color) and percent of cells expressing (size).

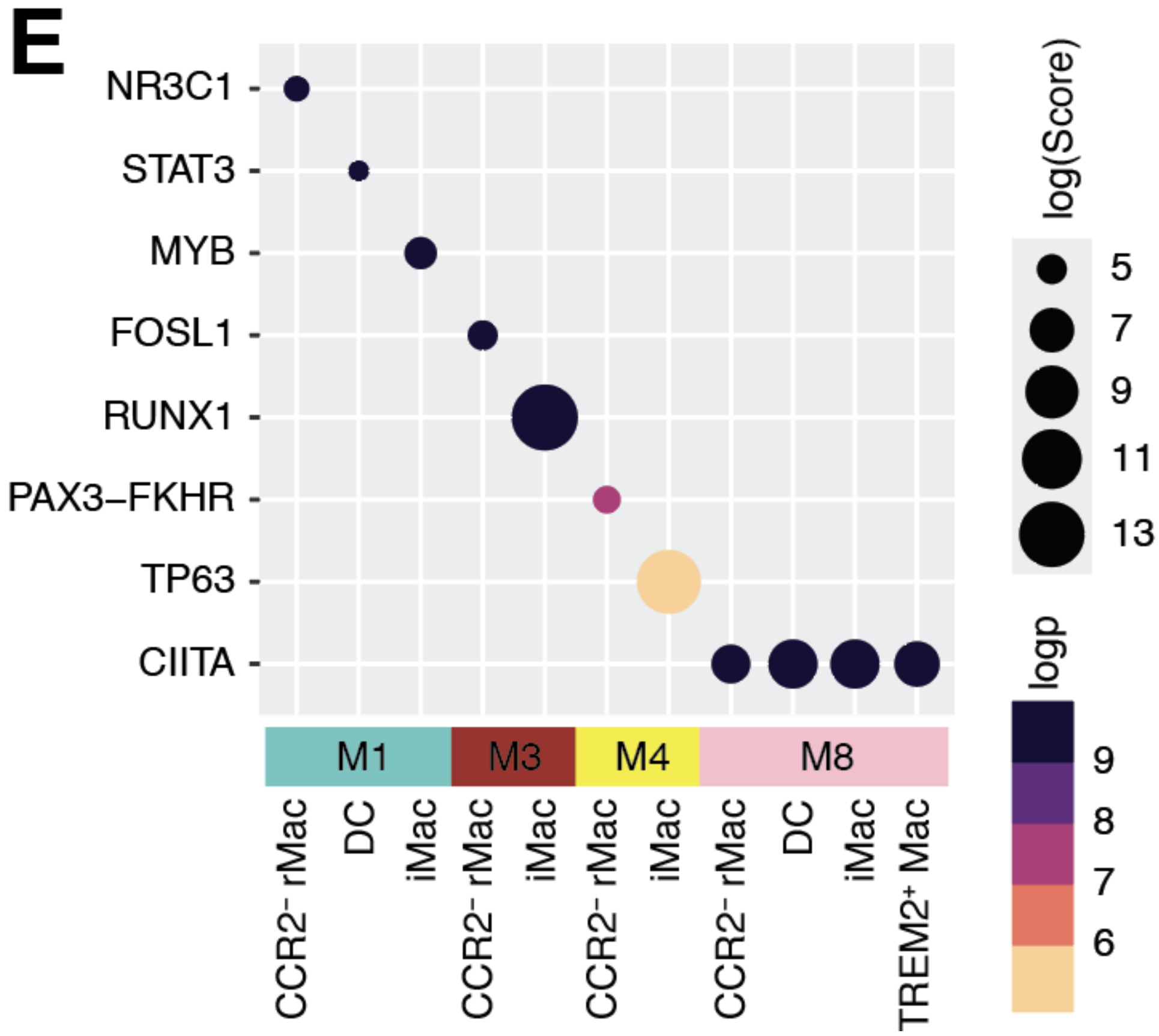

Figure 6E: Myeloid enrichments. ChEA transcription factor enrichment for module genes upregulated in RVF vs NF.

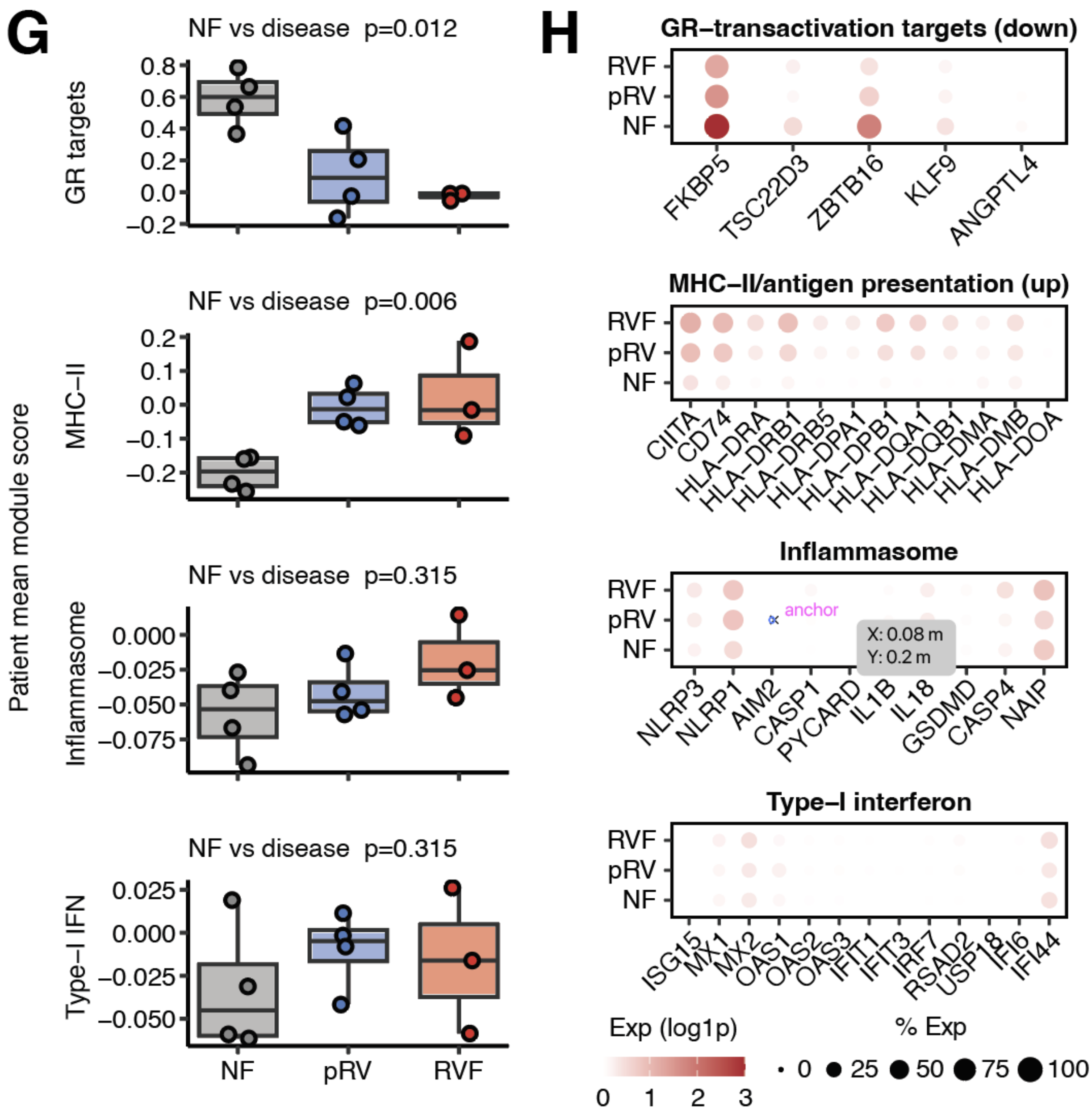

Figure 6 G & H: Myeloid programs. (G) Pseudobulk module-score violin plots for the four myeloid programs across NF/pRV/RVF: glucocorticoid-receptor (GR) transactivation, MHC-II/antigen-presentation, NLRP3 inflammasome, and type-I interferon across all myeloid subtypes. Pairwise Wilcoxon and pooled NF vs Disease (pRV+RVF) p-values shown. **(H)** Companion dot plots of canonical genes from each of the four programs shown across NF/pRV/RVF. Shared color (expression, white→red) and size (% expressed) legends.

149 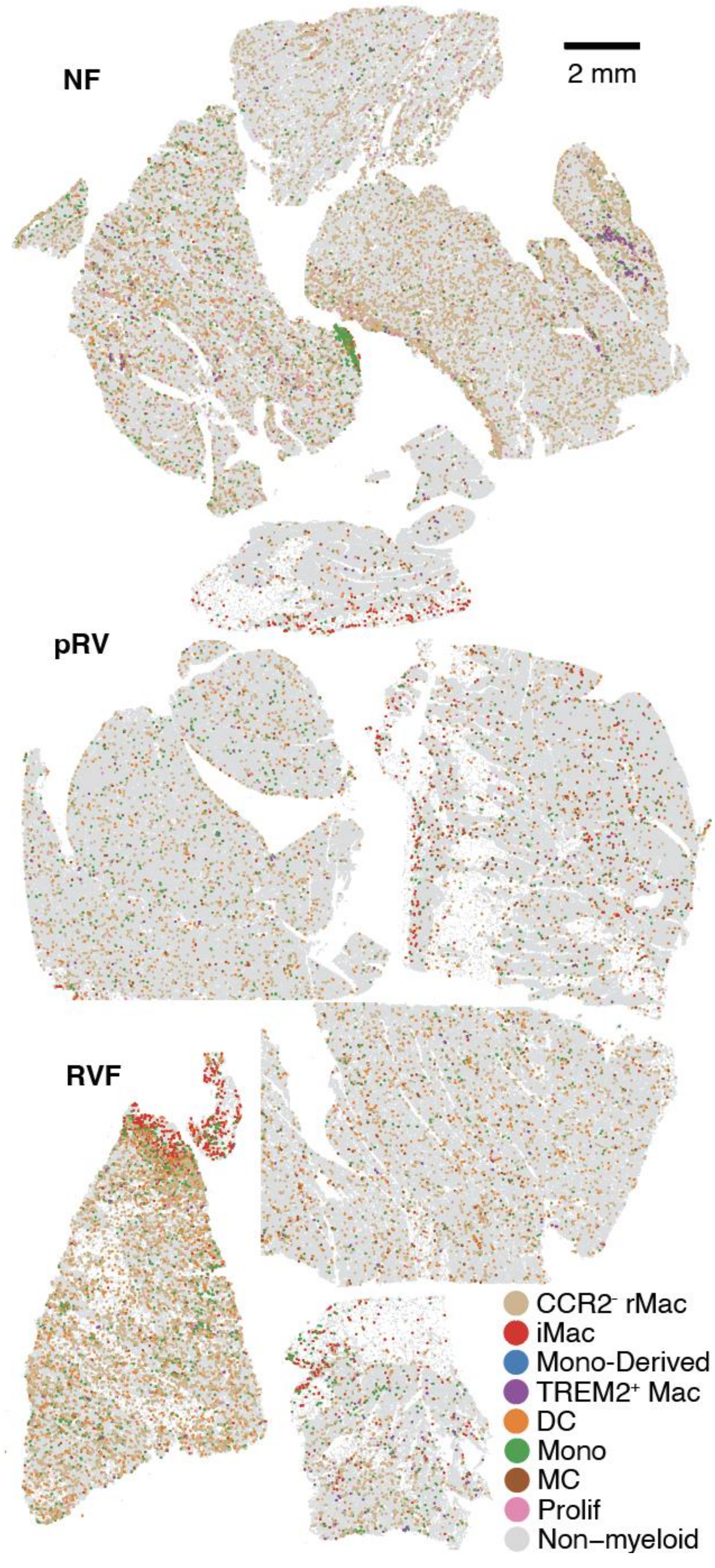
NF
2 mm
pRV
RVF
CCR2⁻ rMac
iMac
Mono-Derived
TREM2⁺ Mac
DC
Mono
MC
Prolif
Non–myeloid

Figure 6I: Spatial myeloid localization. Spatial localization of myeloid subtypes in the full Xenium dataset (625,305 cells). Non-myeloid cells are shown in light grey; CCR2⁻ rMacs and Macrophage C1q are combined.

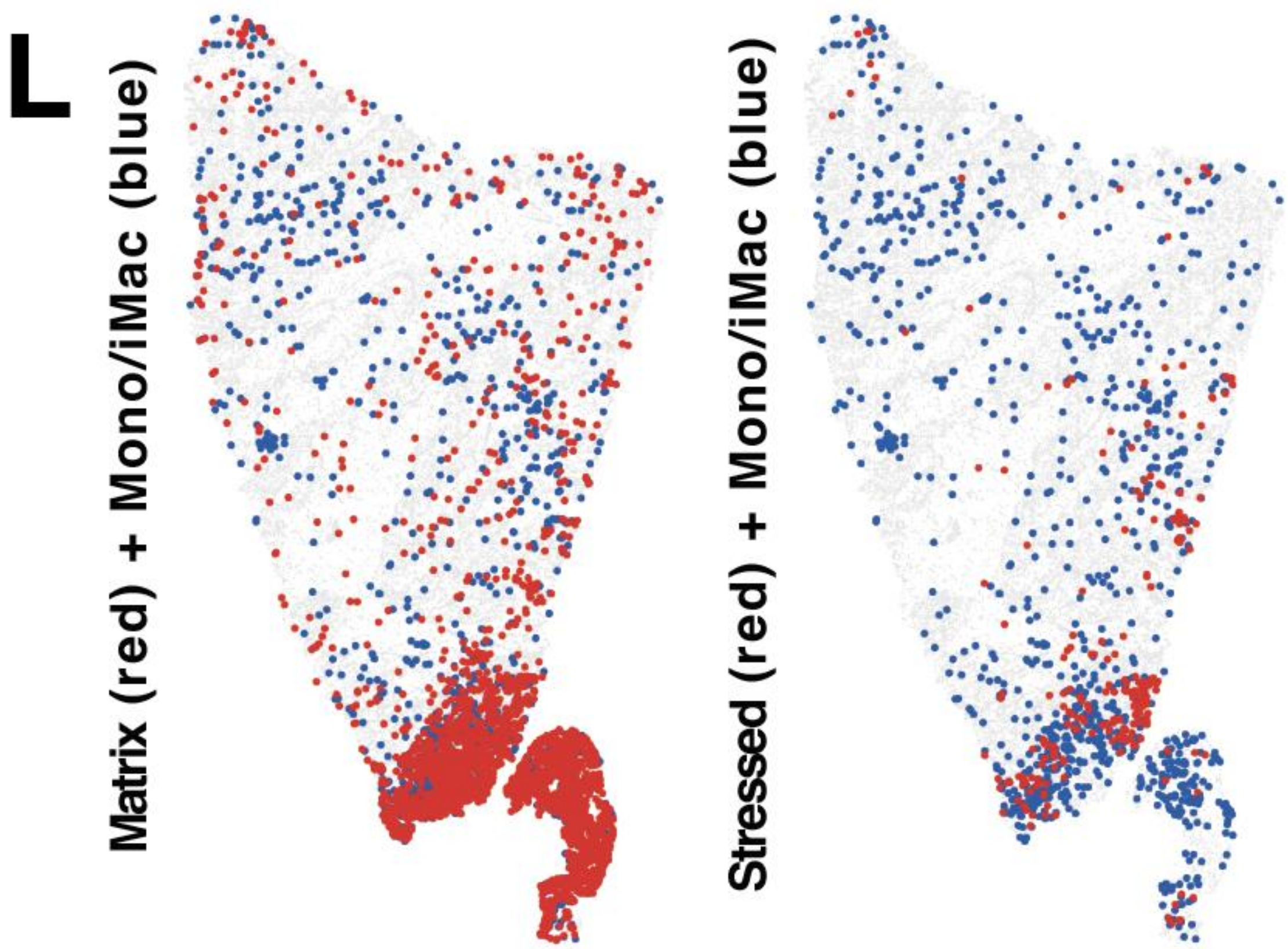

Figure S3L: Monocyte and iMac localization with matrix FBs and stressed FBs. Included here to demonstrate the localized nature of monocyte and iMac distribution. All baseline cells grey, iMac + Monocyte highlighted in blue, and either Xen FB Stressed (left) or Xen FB Matrix (right) highlighted in red

Fourth, to further explore potential drivers of this inflammatory state, we performed ligand–receptor interaction analysis integrating the snRNA-seq and spatial transcriptomic datasets.

Finally, in line with the spatial co-occurrence between iMacs and monocytes (**Figure 6I**) and the absence of imaging-based lineage tracing, we have softened our interpretation throughout. We now describe these changes as a shift in macrophage activation state rather than a definitive lineage transition, and we explicitly acknowledge that the relative contributions of resident-macrophage transition versus monocyte-derived recruitment cannot be resolved from the present data.

Together, these additional analyses provide stronger support for macrophage activation and antigen presentation programs in RVF while avoiding overinterpretation regarding macrophage ontogeny.

Changes in manuscript:

- Added characterization of $CCR2^{-}$ resident macrophages using the conserved TIMD4/LYVE1/FOLR2 (TLF) framework alongside CCR2-based classification (Results: myeloid populations; **Figure 6A–B**).
- Added Xenium spatial analysis of myeloid subtype localization (new **Figure 6I**), demonstrating co-occurrence of inflammatory macrophages and monocytes at the tissue periphery.
- Added ligand–receptor interaction analysis (CellChat) integrating snRNA-seq and Xenium spatial transcriptomics to identify candidate signaling drivers of RVF.
- Expanded analysis of MHCII / CIITA-regulated transcriptional programs in RVF myeloid populations, including enrichment of bulk WGCNA module M8 for CIITA targets in iMac and TLF-resident populations (**Figures 6E–H**).
- Revised text throughout to describe macrophage remodeling as a shift in activation state rather than a definitive lineage transition. Explicitly acknowledged that resident-macrophage activation versus monocyte-derived recruitment cannot be resolved without imaging-based lineage tracing.
- Expanded Discussion of the resident-vs-recruited macrophage question, the limits of the current dataset for resolving ontogeny, and the divergent macrophage response in the murine PAB model (loss of MHCII induction).

3. Please include functional validation to clarify the interpretation of the results related to the downregulation of the glucocorticoid response element (GRE), particularly regarding the activation or repression of GRE-associated genes (R#2). Additionally, consider using a pharmacological or genetic mouse model to understand whether these findings are recapitulated in the PAB model (R#4).

We agree with the reviewer that activation of the glucocorticoid receptor can lead to either transcriptional activation or repression depending on the specific GRE context. Accordingly, we have revised the manuscript to clarify that the observed downregulation of GRE-associated genes should not be interpreted as direct evidence of reduced glucocorticoid receptor signaling. Instead, we now describe this observation more conservatively as a relative reduction in the expression of genes previously identified as glucocorticoid-responsive, which may reflect altered glucocorticoid signaling, altered transcriptional context, or other regulatory mechanisms.

Second, we further expanded the description of the transcription factor enrichment analysis and clarified the interpretation of these results throughout the Results and Discussion sections. The revised text emphasizes that these findings highlight a potential role for altered glucocorticoid-axis regulation in human RVF that warrants targeted functional investigation in future studies.

Third, the reviewer suggested evaluating these findings in pharmacological or genetic mouse models. We discussed this point with the editors during the revision process and, given the scope of the current study and the extensive additional experimental work already undertaken during revision, it was agreed that generation of additional mouse models was not required for this revision. Moreover, our existing murine PAB dataset already allows assessment of whether the glucocorticoid-associated transcriptional signatures observed in human RVF are reproduced in this commonly used model. As reported in the manuscript, we found that NR3C1 target gene signatures do not show significant changes in the PAB dataset, indicating that this aspect of the human RVF transcriptional program is not recapitulated in the murine model, and thus experimental investigation of the GR program in this model may not be useful.

We have expanded the Results and Discussion sections to highlight this discrepancy and to further emphasize that murine PAB models only partially reproduce the transcriptional programs observed in human RVF.

Changes in manuscript:

- Revised interpretation of GRE-associated transcriptional signatures throughout the manuscript.
- Clarified that reduced expression of GRE-associated genes does not necessarily indicate reduced glucocorticoid receptor activity.
- Expanded discussion of glucocorticoid signaling and its potential role in RVF.
- Added clarification that NR3C1 target gene signatures are not altered in the murine PAB model, highlighting species/model differences.

4. Please provide analysis of mitochondrial function and gene expression of the PAB model at a later time point after the compensatory period (R#5).

We thank the reviewer for this important suggestion. To address the possibility that the early 2-week PAB time point reflects a compensatory phase rather than established RVF, we extended the PAB model to a later time point.

Specifically, we performed additional experiments in mice subjected to pulmonary artery banding for two months, a time point expected to represent a more chronic stage of pressure overload. We then repeated the mitochondrial functional assays using high-resolution respirometry on RV tissue from these animals.

These experiments revealed that mitochondrial respiratory capacity remained significantly reduced at the two-month time point, consistent with the findings observed at earlier stages of the model. This result indicates that mitochondrial dysfunction persists during chronic RV pressure overload and is not limited to an early compensatory phase.

Together with the newly added respirometry analyses in both the PAB model and pediatric RV samples (see Response to Editorial Comment 1), these data provide strong

functional support for mitochondrial dysfunction as a feature of RV remodeling across multiple contexts.

We note that the primary goal of this additional experiment was to determine whether mitochondrial functional impairment persists at later stages of disease. Because the transcriptomic analyses of the murine model were already extensively characterized in the original dataset and the revision focused on functional validation, we did not perform additional transcriptomic profiling at the later time point.

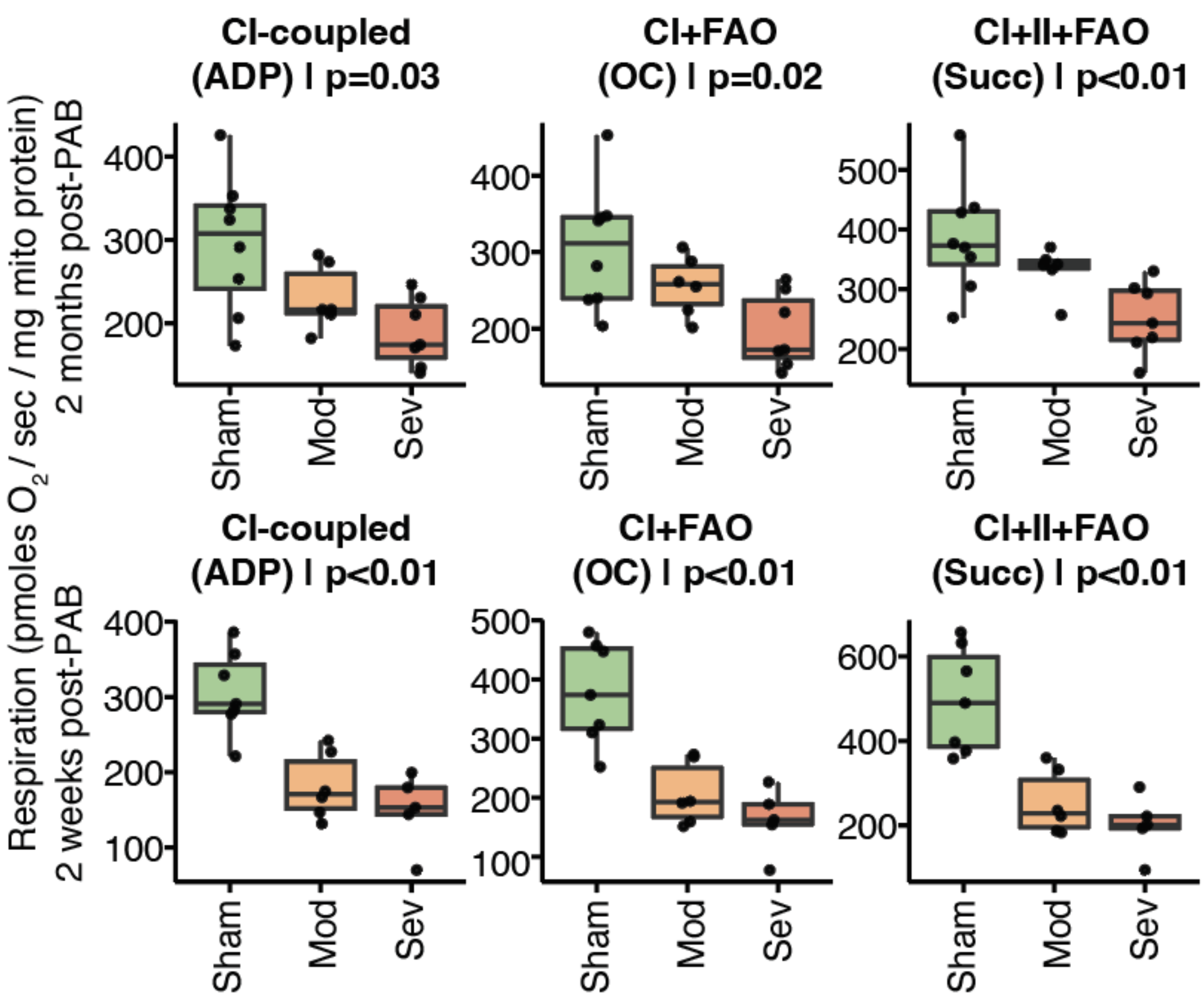

Figure 5I: PAB respirometry. Respirometry in PAB model at 2-month **(top)** and 2-week **(bottom)** time points vs sham, using a substrate-addition protocol (PM → ADP → cyt c → octanoyl-carnitine → succinate) on tissue homogenate. CI+II+FAO maximal coupled respiration (Succ-driven) is the headline measure. 2-week: Sham n=8, Mild n=6, Severe n=7. 2-month: Sham n=7, Mild n=6, Severe n=5. Each dot is one animal; Kruskal–Wallis p-values shown.

Changes in manuscript:

- Added mitochondrial respirometry analysis from two-month PAB RV samples.
- Updated the Results section describing mitochondrial function in the murine model.

- Expanded the Discussion to clarify that mitochondrial dysfunction persists during chronic pressure overload in the PAB model.

5. Please follow R#3 advice and add author-driven interpretations or speculative hypotheses in the discussion.

In the revised manuscript, we have expanded the Discussion to include several author-driven interpretations and hypotheses based on the transcriptional and functional findings reported in this study.

First, we expand on the relationship between mitochondrial transcription and respiratory function in RVF. We observe an interesting pattern across cohorts: adult and murine RVF cardiomyocytes show coordinated downregulation of nuclear-encoded mitochondrial transcripts paralleled by reduced respirometric capacity. Pediatric HLHS-palliated failing RVs show minimal, not significant changes in mitochondrial transcripts with preserved respiratory capacity. We discuss this divergence as evidence that mitochondria undergo coupled transcriptional and functional decline.

Second, we discuss potential mechanisms underlying the pro-inflammatory shift in cardiac macrophage populations. We frame this as a shift in macrophage activation state and explicitly acknowledge that the relative contributions of resident-macrophage transition versus monocyte-derived recruitment cannot be resolved from the present data, given the spatial co-occurrence of inflammatory macrophages and monocytes at the tissue periphery. Our spatial transcriptomic and ligand–receptor analyses suggest that stage-specific interactions between cardiomyocytes, endothelial cells, fibroblasts, and macrophages contribute to this remodeling.

Third, we expand our interpretation of the endothelial expansion observed in RVF, discussing potential adaptive and maladaptive roles of vascular remodeling during chronic pressure overload.

Together, these additions provide a more integrated conceptual framework linking the observed transcriptional programs to potential mechanisms of RV remodeling and failure.

Changes in manuscript:

- Expanded the Discussion section to include author-driven interpretations and hypotheses related to:
    - mitochondrial dysfunction in RVF
    - macrophage activation and antigen presentation
    - endothelial expansion and vascular remodeling
- Integrated findings from the spatial transcriptomic and ligand–receptor analyses into the Discussion.

6. Please address in full all technical and methodological concerns raised by R#2 and R#3.

We have substantially revised the analysis pipelines, figures, and Methods section to improve transparency, reproducibility, and interpretability of the datasets.

First, we expanded the overview of dataset quality and composition. We now explicitly report the number of nuclei represented in UMAP figures and corresponding legends. In addition, for the snRNA-seq integration we added a patient-colored inset (**Figure 2A**) to illustrate donor-consistent integration.

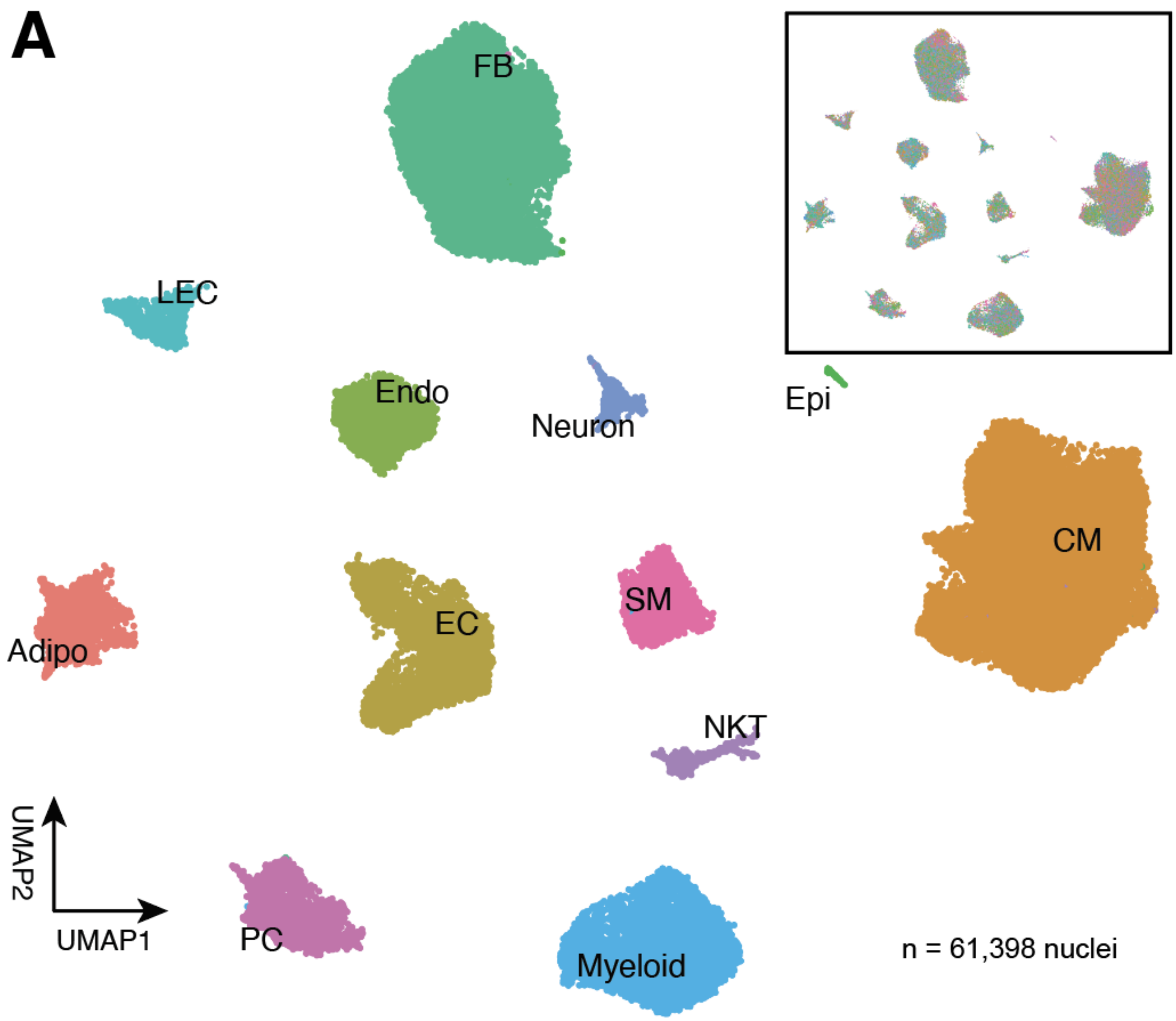

Figure 2A: snRNA-seq lineage UMAP. UMAP of 61,398 nuclei coloured by 12 major lineage clusters (CM, FB, EC, Endo, SM, Myeloid, NKT, PC, Adipo, Epi, LEC, Neuron); inset shows the same UMAP coloured by patient (n=11) to illustrate donor-consistent integration.

Second, we expanded the visualization of cell-type and subcluster annotations. We added dot plots of canonical marker genes supporting the annotation of cardiomyocyte,

endothelial, and myeloid subclusters (e.g. **Figure 6B**). These visualizations improve interpretability of the clustering results and directly address the reviewers' request for clearer annotation support.

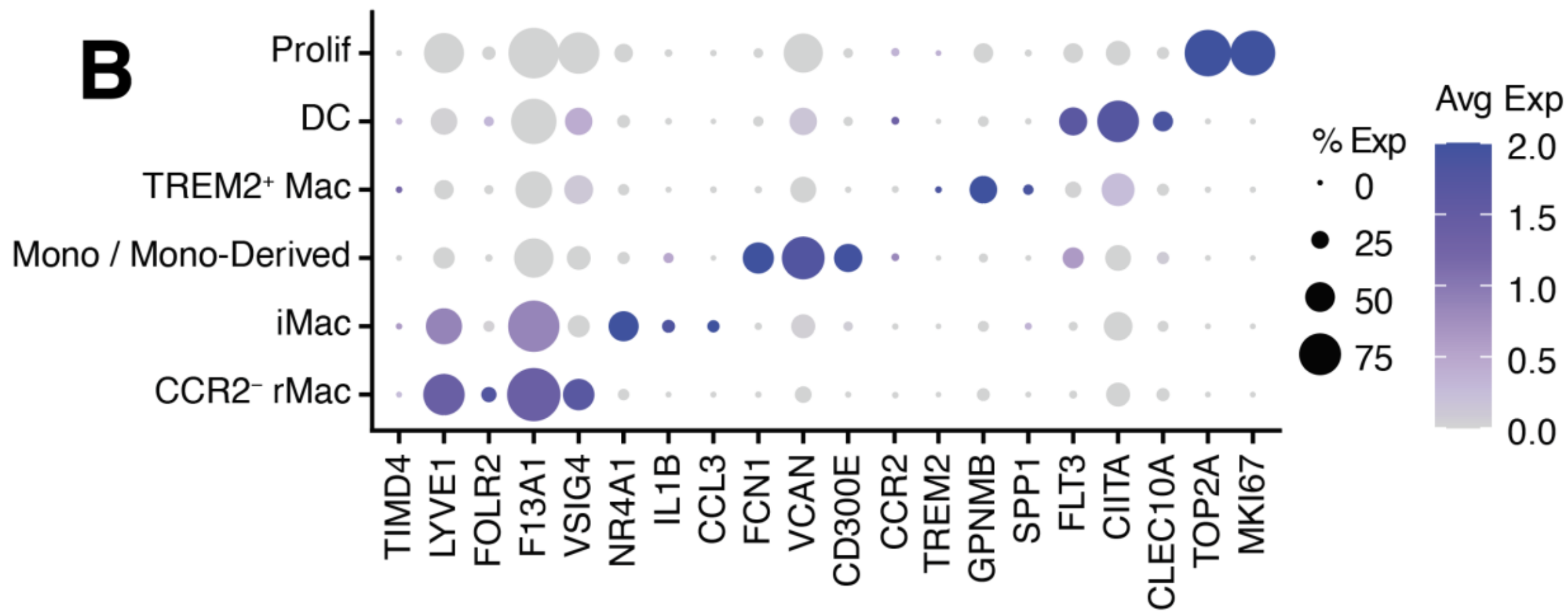

Figure 6B: Myeloid subtype marker genes. Dot plot of canonical marker genes per myeloid subtype. Mean expression (color) and percent of cells expressing (size).

Third, we expanded the description of clustering methods. The Methods section now explicitly reports clustering parameters, including the number of principal components used, nearest-neighbor construction, and clustering resolution. We have notably removed pseudotime trajectory analysis in this version.

Fourth, we clarified doublet detection. The Methods now describe the semi-supervised doublet identification strategy used during snRNA-seq preprocessing.

Fifth, we performed a major re-analysis of the Xenium spatial transcriptomic dataset to address several reviewer concerns regarding spatial analysis (see **Figure 3**). The dataset was reprocessed using an updated spatial analysis pipeline including:

Cell segmentation using Proseg (Baysor was evaluated but oversegmented), improving transcript assignment to individual cells.

Correction of diffusion-induced transcript contamination using SPLIT-based cellular de-mixing.

Gene expression imputation using SpaGE to integrate single-nucleus RNA-seq information into the spatial dataset.

Spatial cell-type assignment using RCTD, leveraging the snRNA-seq dataset as a reference.

These improvements substantially increased the number of confidently segmented cells and improved spatial cell-type identification and downstream spatial analyses.

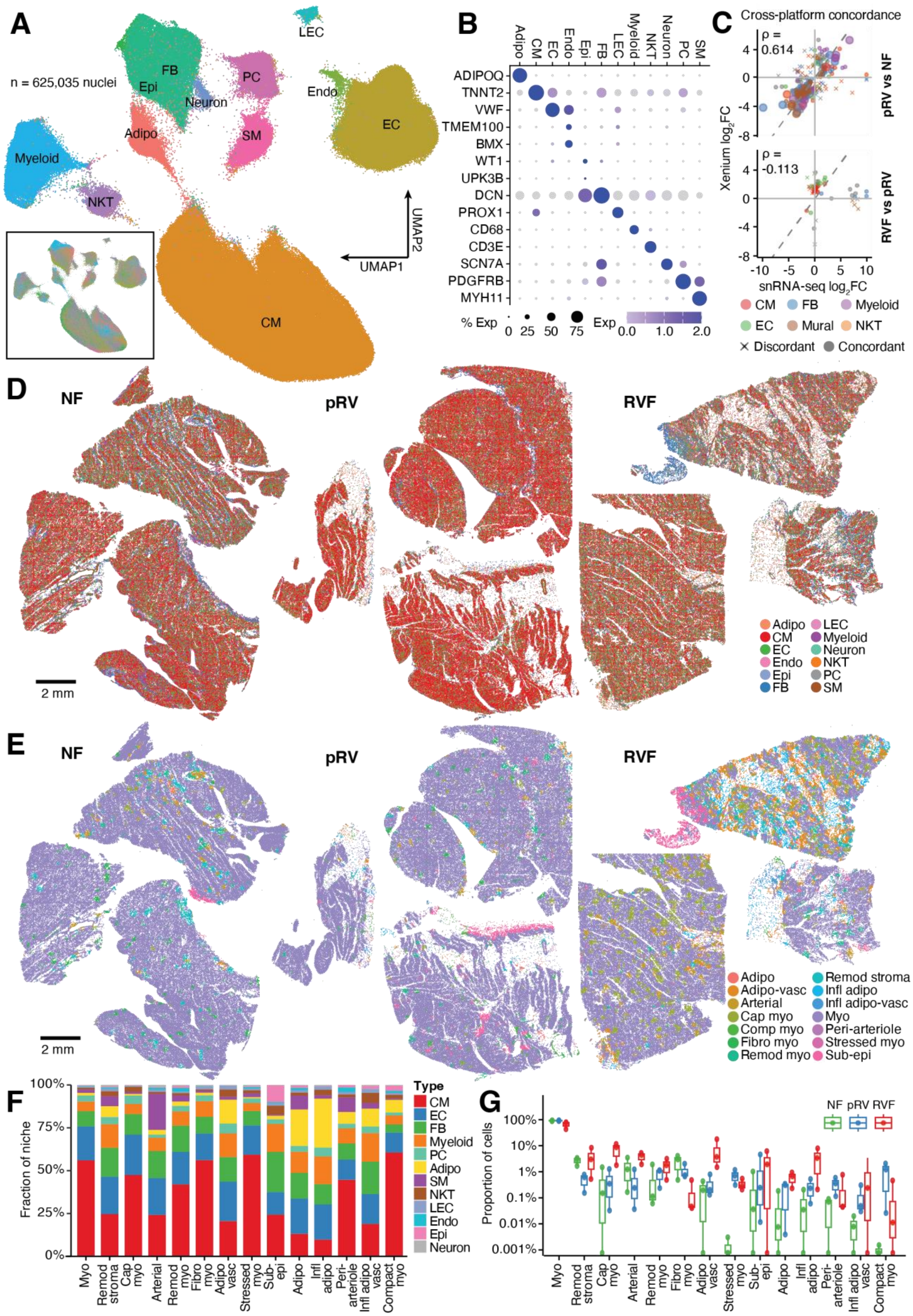

A
n = 625,035 nuclei
FB
Epi
Neuron
PC
LEC
Endo
EC
Adipo
SM
Myeloid
NKT
CM
UMAP1
UMAP2
B
ADIPOQ
TNNT2
VWF
TMEM100
BMX
WT1
UPK3B
DCN
PROX1
CD68
CD3E
SCN7A
PDGFRB
MYH11
% Exp
Exp
C
Cross-platform concordance
pRV vs NF
RVF vs pRV
Xenium log2FC
snRNA-seq log2FC
Discordant
Concordant
D
NF
pRV
RVF
2 mm
E
Adipo
Adipo-vasc
Arterial
Cap myo
Comp myo
Fibro myo
Remod myo
Remod stroma
Infl adipo
Infl adipo-vasc
Myo
Peri-arteriole
Stressed myo
Sub-epi
F
Fraction of niche
Type
Myeloid
Neuron
G
Proportion of cells
Stressed myo
Compact myo

Figure 3. Xenium spatial transcriptomic atlas of the right ventricle. (A) Xenium UMAP of 625,305 cells coloured by 12 lineage clusters; inset shows the same UMAP coloured by patient (n=9) to illustrate donor-consistent integration. **(B)** Dotplot of canonical lineage markers across the 12 Xenium lineages; dot colour encodes mean expression and dot size encodes the percentage of expressing cells. **(C)** Cross-platform concordance scatter of pseudobulk $\log_2$FC: snRNA-seq (x-axis) vs Xenium (y-axis) for matched lineages and contrasts, with points coloured by lineage and symbols separating concordant (●) from discordant (×) calls; Spearman r reported per comparison. **(D)** Spatial maps of all NF, pRV and RVF sections after Proseg resegmentation and RCTD annotation, coloured by lineage. Endocardial border and scale bars indicated where applicable. **(E)** Spatial maps of the same sections coloured by spatial niche assignment, illustrating the spatial organization of the 14 microenvironments resolved by BuildNicheAssay (k=100, CLR-transformed). **(F)** Stacked bar chart of niche composition by lineage (fraction-of-niche), summarizing the cellular identity of each microenvironment. Niche labels are abbreviated as: adipo (adipocyte-rich), adipo-vasc (adipo-vascular), arterial, cap myo (capillary myocardium), comp myo (compact myocardium), fibro myo (fibrotic myocardium), remod myo (remodeling myocardium), remod stroma (remodeling stroma), infl adipo (inflammatory adipocyte), infl adipo-vasc (inflammatory adipo-vascular), myo (myocardium), peri-arteriole, stressed myo (stressed myocardium) and sub-epi (sub-epicardial). **(G)** Niche frequency boxplots across NF, pRV and RVF. Y-axis is showed on a log-scale.

Sixth, we clarified aspects of tissue preparation and spatial sampling. Xenium samples were sectioned transmurally across the right ventricular wall, ensuring comparable tissue orientation across samples.

Seventh, we improved reproducibility of the analysis code. The public analysis repository has been substantially updated to include: a detailed README file, explicit instructions for reproducing analyses, instructions for constructing a Conda environment containing the required software dependencies. The updated repository is available at:https://github.com/ikuznet1/RV_Atlas

Finally, we added a Supplementary Data index table describing the contents of all supplementary datasets to facilitate navigation of the data resources.

Together, these revisions substantially improve the transparency, reproducibility, and interpretability of the study while addressing the technical and methodological concerns raised by the reviewers.

7. Additionally, it is our strong editorial opinion that you should follow R#1 and R#2 suggestions to improve the manuscript's accessibility and clarity by revising and reorganizing the presentation of data and analyses.

We thank the editors for this suggestion. In response, we have revised and reorganized the manuscript to improve clarity and accessibility of the presented analyses. In particular, we streamlined the presentation of the bulk RNA-seq, single-nucleus RNA-seq, and

spatial transcriptomic analyses, clarified the logical flow of the Results section, and expanded explanatory text where necessary to better guide readers through the analytical framework. We believe these revisions substantially improve the readability and accessibility of the manuscript.

Reviewer #1 (Remarks to the Author):

Thank you for allowing me to review the manuscript by Kuznetsov et al in which the authors performed a deep transcriptomics based assessment of RV failure. The authors used bulkRNAseq, snRNAseq, and spatialomics to evaluate the molecular signature of RV failure. Overall, this is a much needed analysis but there are several areas that would be needed for improvement to make this a more valuable contribution, which I do believe it is.

*1. I think for **Figure 1,** the authors should just perform deconvolution RNAseq rather than the module analysis that they did. The authors have the actual map of RV failure at the single nucleus level in FIgure 2, so why not? This would give a lot more data. I appreciate the limitations of deconvolution, but you can directly compare to your snRNAseq data. I appreciate the module system they employed, but it is a little more difficult to understand and not sure it really is more valuable.*

We thank the reviewer for this suggestion. In response, we explored multiple approaches for deconvolution of the bulk RNA-seq dataset using the single-nucleus RNA-seq data as a reference.

Specifically, we tested two widely used deconvolution methods: BayesPrism and BisqueRNA. However, in our dataset these approaches did not produce consistent or biologically interpretable results. In particular, the inferred cell-type proportions were not concordant between methods and did not reliably reproduce the disease-associated shifts in cell populations observed in the single-nucleus RNA-seq dataset. Additionally, the recovered cell-type–specific gene expression profiles were inconsistent with those derived directly from the snRNA-seq analysis.

These limitations likely reflect challenges inherent to deconvolution of complex cardiac tissue using relatively small single-nucleus reference datasets and heterogeneous bulk samples. Because the inferred results were inconsistent across methods and could not be validated against the single-nucleus data, we elected not to include these analyses in the manuscript.

Importantly, our study already directly resolves cell-type–specific transcriptional changes using snRNA-seq profiling of RV samples, allowing us to map gene modules identified in the bulk dataset to specific cell populations with high confidence. We therefore retained the WGCNA-based framework for analyzing the bulk dataset, which provides robust identification of coordinated transcriptional programs across the large cohort of bulk samples. The full deconvolution code for both methods is provided in the repository for transparency.

2. The analysis of the spatial-omics data are relatively limited. Maybe that is because there were not as many differences. It may be useful to comment on the relative loss of CMs in snRNAseq that was not as drastic as observed in the spatial data.

We thank the reviewer for this observation. In the revised manuscript, we have substantially expanded the spatial transcriptomic analysis (new **Figure 3**). The Xenium dataset was fully reprocessed using an updated pipeline comprising Proseg-based resegmentation, SPLIT ambient RNA correction, RCTD cell-type annotation using our snRNA-seq atlas as reference, and SpaGE gene expression imputation. This enabled confident recovery of all 12 major cell types and subclustering into 58 subtypes across the 12 lineages, pseudobulk DESeq2 differential expression analysis per subtype across disease states, and spatially-informed ligand–receptor interaction analysis using CellChat.

Regarding the relative loss of cardiomyocytes: the snRNA-seq data show a progressive decrease in cardiomyocyte nuclear proportion from NF to pRV to RVF. In the Xenium data, interpretation of cardiomyocyte proportions is more nuanced. We observed fewer cardiomyocyte nuclei in RVF compared to NF, consistent with the snRNA-seq trend. However, in pRV, cardiomyocyte nuclear counts were higher, perhaps reflecting cardiomyocyte hypertrophy with increased nuclei per cell rather than an expansion in cardiomyocyte number or relecting an artifact from cell/nuclei segmentation. These observations highlight the importance of distinguishing nuclear counts from cell counts when interpreting proportional shifts, particularly in the context of cardiomyocyte hypertrophy during compensated pressure overload. We have now substantiated this hypertrophy framing with direct measurement: WGA staining of human RV sections (NF n=4, pRV n=4, RVF n=4; 17,864 CMs segmented via CellProfiler) confirms progressive cardiomyocyte hypertrophy across NF→pRV→RVF (per-patient median minimum Feret diameter: NF 55.8 µm; pRV 73.9 µm [+32%, BH-adjusted p = 0.011]; RVF 102.5 µm [+84%, $p < 10^{-5}$]; pRV vs RVF p = 0.026; cell-level linear mixed model; new **Figure S2I**; **Data S16**). We have discussed these cross-platform differences and the supporting CM-size histology in the revised manuscript.

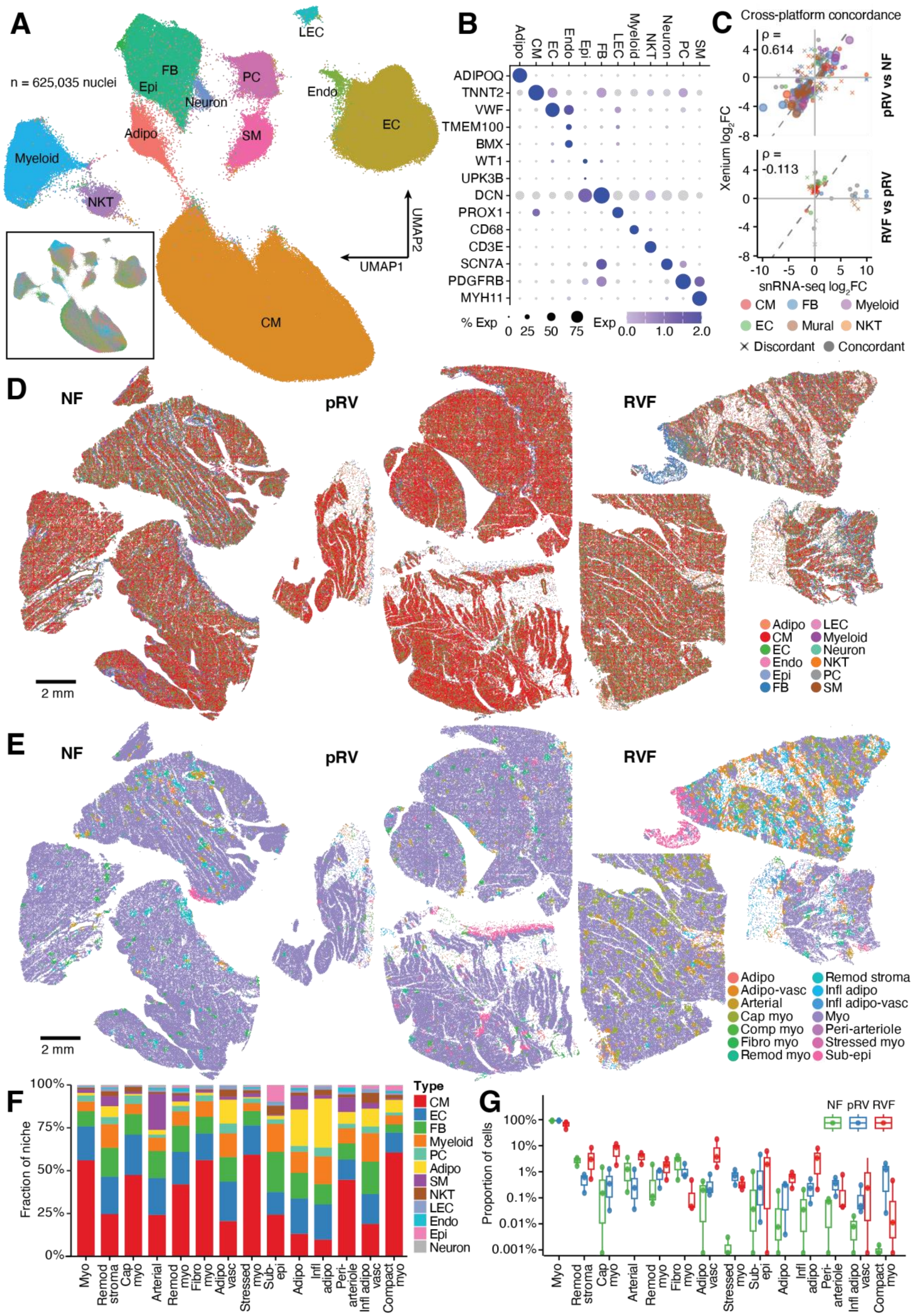
A
n = 625,035 nuclei
FB
Epi
Neuron
PC
LEC
Endo
EC
Adipo
SM
Myeloid
NKT
CM
UMAP1
UMAP2
B
ADIPOQ
TNNT2
VWF
TMEM100
BMX
WT1
UPK3B
DCN
PROX1
CD68
CD3E
SCN7A
PDGFRB
MYH11
% Exp
Exp
C
Cross-platform concordance
pRV vs NF
RVF vs pRV
Xenium log2FC
snRNA-seq log2FC
CM FB Myeloid
EC Mural NKT
Discordant Concordant
D
NF
pRV
RVF
2 mm
E
Remod stroma
Infl adipo
Infl adipo-vasc
Myo
Peri-arteriole
Stressed myo
Sub-epi
Adipo-vasc
Arterial
Cap myo
Comp myo
Fibro myo
Remod myo
F
Fraction of niche
Type
G
Proportion of cells
Compact myo

Figure 3. Xenium spatial transcriptomic atlas of the right ventricle. (A) Xenium UMAP of 625,305 cells coloured by 12 lineage clusters; inset shows the same UMAP coloured by patient (n=9) to illustrate donor-consistent integration. **(B)** Dotplot of canonical lineage markers across the 12 Xenium lineages; dot colour encodes mean expression and dot size encodes the percentage of expressing cells. **(C)** Cross-platform concordance scatter of pseudobulk $\log_2$FC: snRNA-seq (x-axis) vs Xenium (y-axis) for matched lineages and contrasts, with points coloured by lineage and symbols separating concordant (●) from discordant (×) calls; Spearman r reported per comparison. **(D)** Spatial maps of all NF, pRV and RVF sections after Proseg resegmentation and RCTD annotation, coloured by lineage. Endocardial border and scale bars indicated where applicable. **(E)** Spatial maps of the same sections coloured by spatial niche assignment, illustrating the spatial organization of the 14 microenvironments resolved by BuildNicheAssay (k=100, CLR-transformed). **(F)** Stacked bar chart of niche composition by lineage (fraction-of-niche), summarizing the cellular identity of each microenvironment. Niche labels are abbreviated as: adipo (adipocyte-rich), adipo-vasc (adipo-vascular), arterial, cap myo (capillary myocardium), comp myo (compact myocardium), fibro myo (fibrotic myocardium), remod myo (remodeling myocardium), remod stroma (remodeling stroma), infl adipo (inflammatory adipocyte), infl adipo-vasc (inflammatory adipo-vascular), myo (myocardium), peri-arteriole, stressed myo (stressed myocardium) and sub-epi (sub-epicardial). **(G)** Niche frequency boxplots across NF, pRV and RVF. Y-axis is showed on a log-scale.

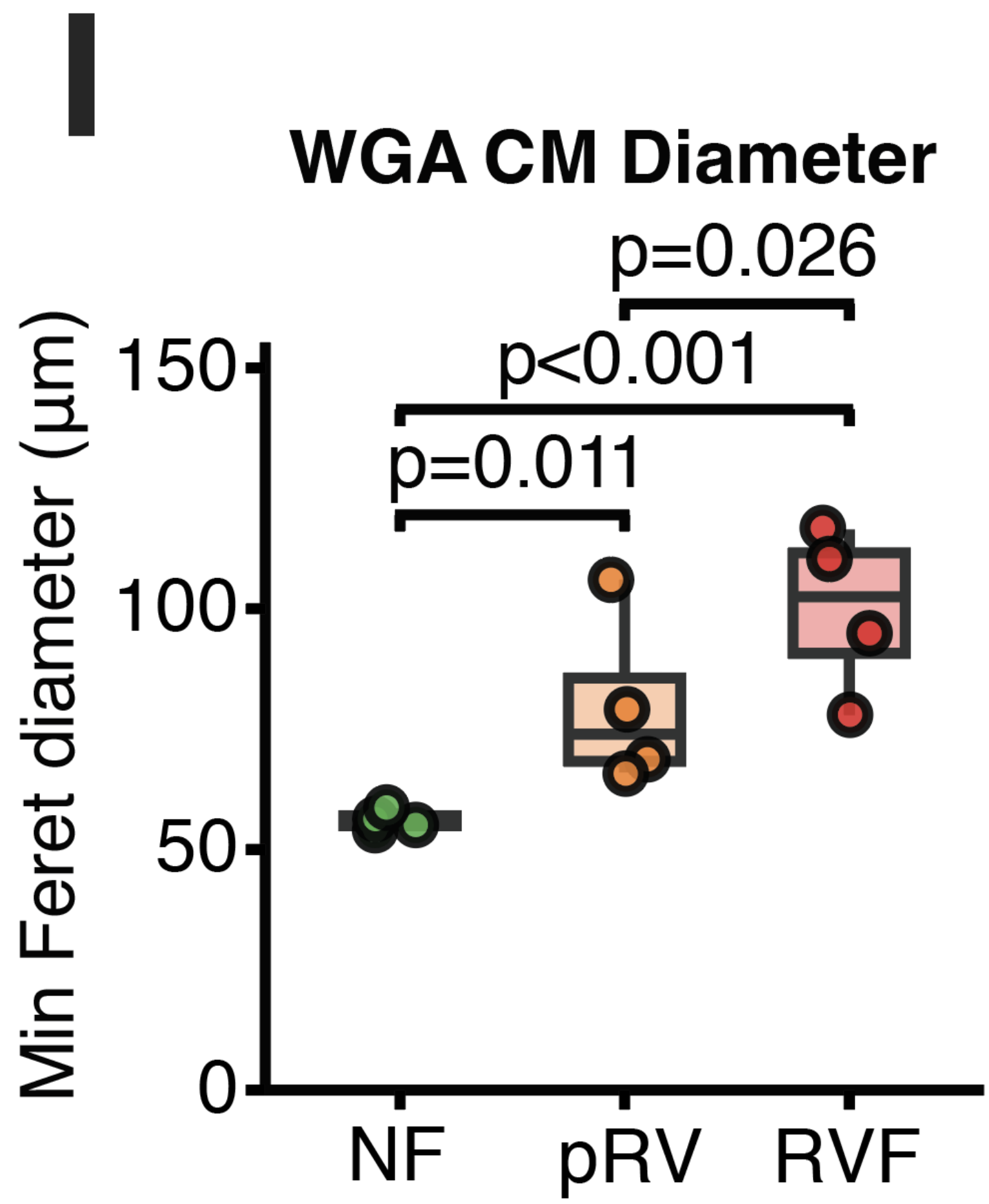

Figure S2I: WGA cardiomyocyte diameter. Cardiomyocyte minimum Feret diameter (proxy for CM cross-sectional area) measured by wheat-germ agglutinin (WGA) staining of human RV sections (NF n=4, pRV n=4, RVF n=4; 17,864 CMs total). Boxplot of per-patient median minimum Feret; each dot is one patient.

Changes in manuscript:

- Substantially expanded the Xenium spatial transcriptomic analysis with updated resegmentation (Proseg), ambient RNA correction (SPLIT), cell-type annotation (RCTD), gene imputation (SpaGE), pseudobulk DESeq2, and ligand–receptor interaction analysis (CellChat).
- Added discussion of cross-platform differences in cardiomyocyte proportional changes between snRNA-seq and Xenium.

3. The comparsion of RVF to LV failure is very nice. However, LV GDMT therapies don't work for RV failure so the authors should comment on this.

We agree, and indeed, the lack of efficacy of LV-directed guideline-directed medical therapies (GDMT) for RVF was a critical clinical motivation for our study. As noted in the Introduction, neurohormonal blockade, a cornerstone of LVF therapy, has little efficacy in RVF, underscoring the need for RV-specific therapeutic targets. In the revised

manuscript, we have further emphasized this point in the Discussion, highlighting that the distinct transcriptional programs identified in this study, including RV-specific changes in mitochondrial gene regulation, myeloid activation, and endothelial remodeling, may help explain the limited efficacy of LV-directed therapies and point toward novel RV-specific therapeutic strategies.

Changes in manuscript:

- Expanded the Discussion to further emphasize the clinical relevance of RV-specific transcriptional programs in the context of the failure of LV-directed GDMT for RVF.

4. The rodent data are not that useful. The authors only show RV dilation and no measures of dysfunction. There are plenty of data from rodents with pulmonary hypertension (bulkRNAseq, proteomics, metabolomics) showing mitochondrial derangements like the authors see in their transcriptomics data. Hard to say preclinical models are not that useful when these data exist. In the end, most RV failure in human HF is due to the presence of Group 2 PH. So a PH model is a better comparison.

We note that Reviewers 3 and 5 requested additional PAB characterization, so we have substantially expanded the functional characterization of the PAB model in the revised manuscript. In addition to the originally reported echocardiographic data, we now include additional morphometric and echocardiographic parameters demonstrating RV dysfunction, mitochondrial respirometry confirming decreased respiratory capacity at both 2-week and 2-month time points (**Figure 5I**), flow cytometric analysis of cardiac macrophage populations (CCR2, TREM2, MHCII; **Figure S5H**), and Sirius Red fibrosis histology (**Figure S6I**).

Regarding the choice of model: we agree that pulmonary hypertension is a major driver of RVF in the context of left heart failure, and we acknowledge the existing literature demonstrating mitochondrial derangements in PH models. Importantly, PAB does model the downstream hemodynamic consequence of PH, namely, RV pressure overload, and we have clarified this point in the revised manuscript. Our goal was to isolate the effects of RV pressure overload from the systemic and pulmonary vascular changes inherent to PH models, which confound interpretation of RV-specific transcriptional responses.

That said, we agree that comparison to human PAH data is valuable and have now included a direct comparison of our bulk RNA-seq findings to the recently published PAH RV transcriptomic dataset (see Response to Comment 5 below).

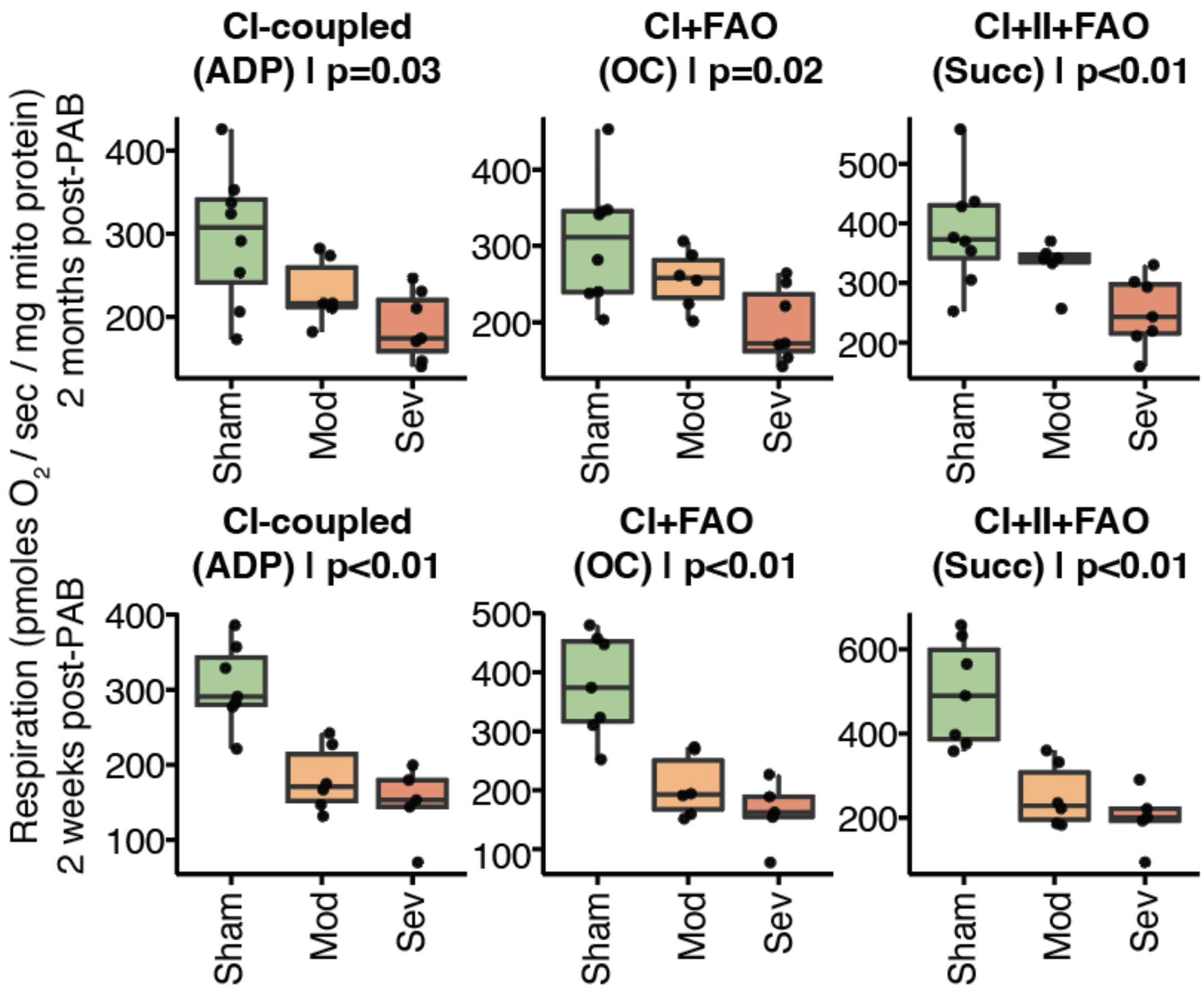

Figure 5I: PAB respirometry. Respirometry in PAB model at 2-month **(top)** and 2-week **(bottom)** time points vs sham, using a substrate-addition protocol (PM → ADP → cyt c → octanoyl-carnitine → succinate) on tissue homogenate. CI+II+FAO maximal coupled respiration (Succ-driven) is the headline measure. 2-week: Sham n=8, Mild n=6, Severe n=7. 2-month: Sham n=7, Mild n=6, Severe n=5. Each dot is one animal; Kruskal–Wallis p-values shown.

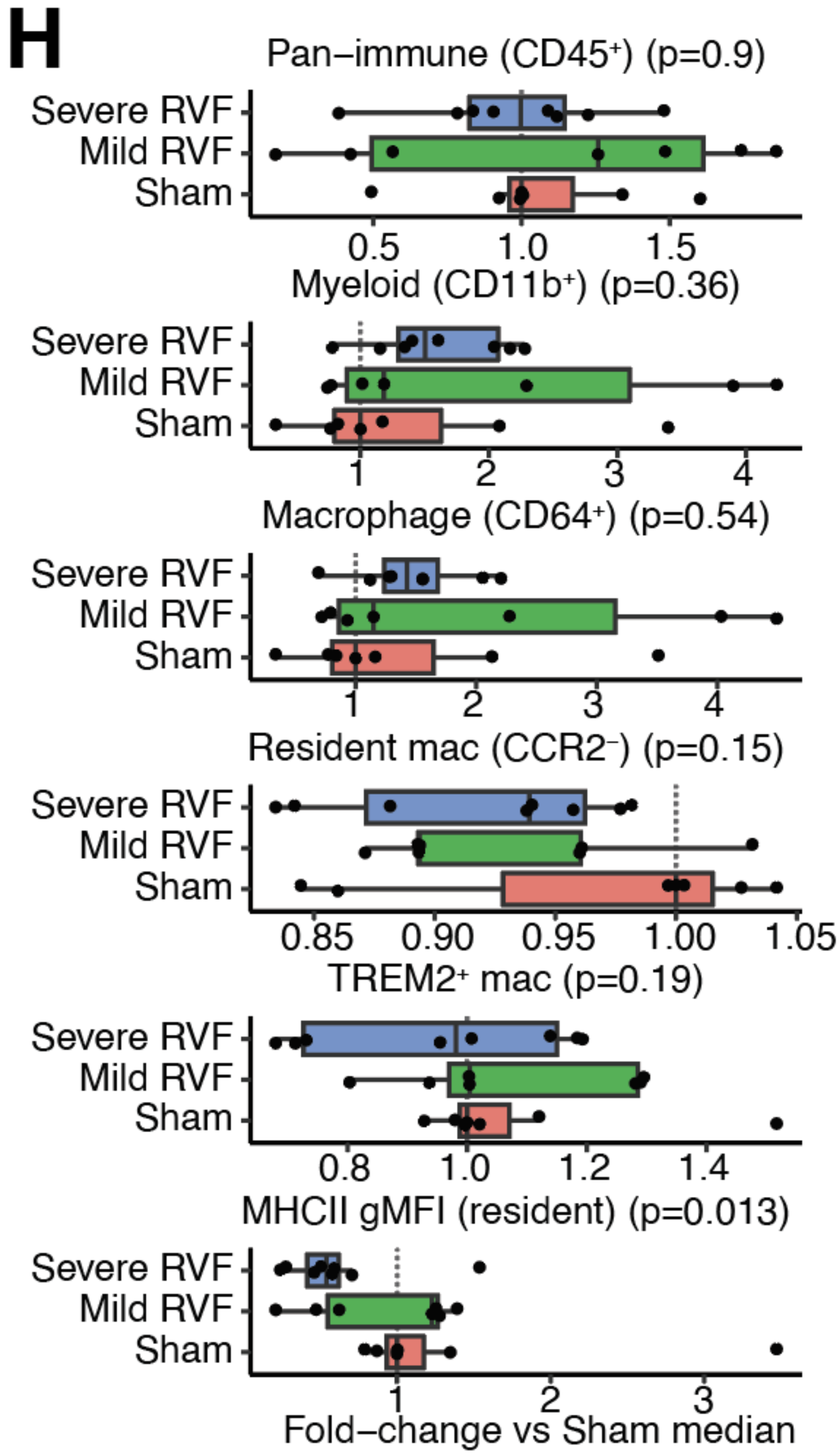

Figure S5H: Flow-cytometry validation of cardiac immune populations in the murine PAB model (Sham/Mild RVF/Severe RVF). Per-mouse fold-change relative to the within experiment Sham median for leukocytes ($CD45^+$), myeloid cells ($CD11b^+Ly\text{-}6G^-$), macrophages ($CD64^+$), $CCR2^-$ resident macrophages, $TREM2^+$ macrophages, and MHC-II geometric mean fluorescence intensity (gMFI) on $CCR2^-$ residents; n = 7/7/8 mice. Log-scale linear-trend p across severity is shown. Antibody clones and catalogue numbers are listed in Methods.

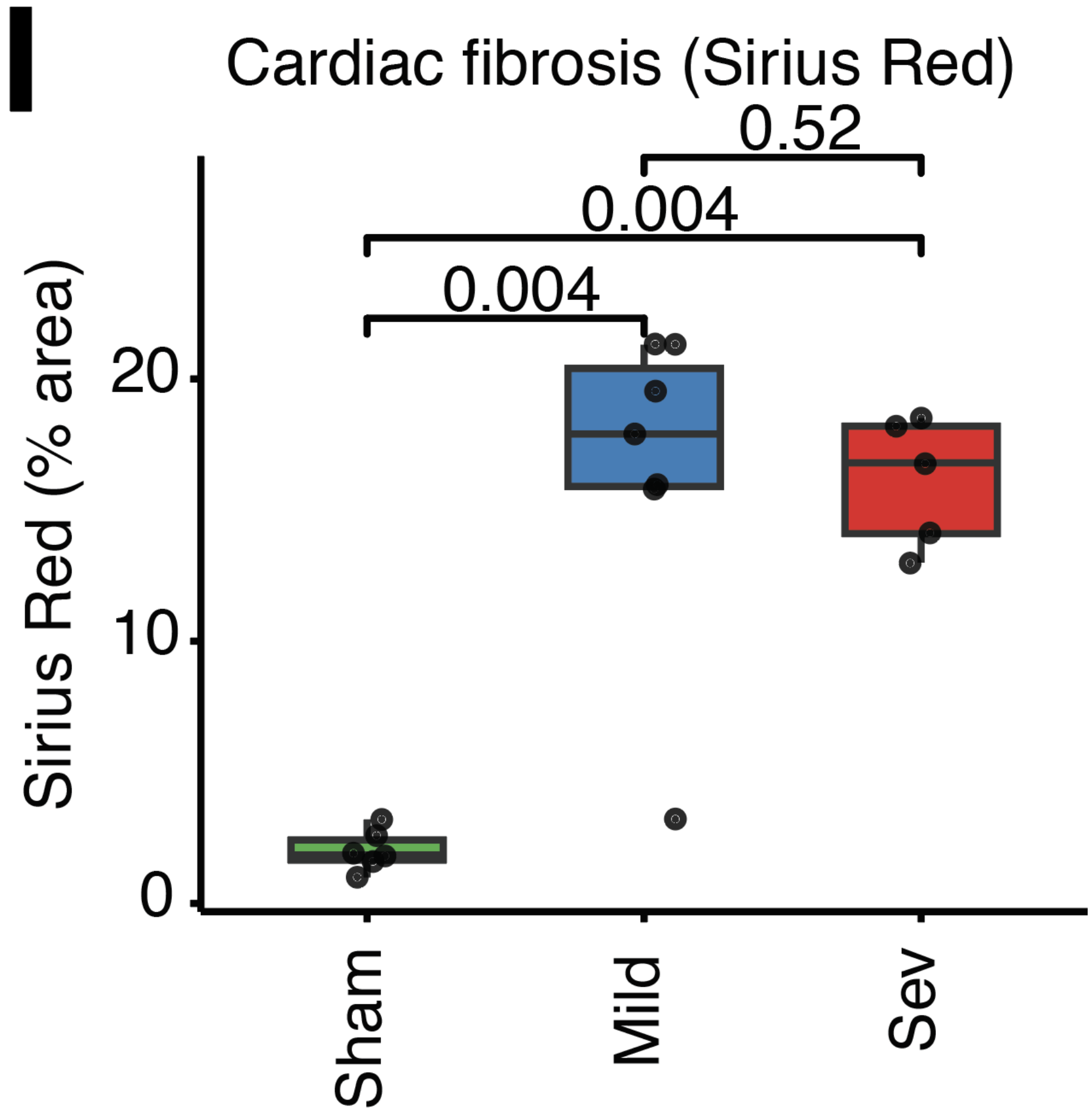

Figure S6I: Cardiac fibrosis after PAB. Cardiac fibrosis quantified as Sirius Red percent area per animal across PAB stages (Sham n = 6, Mild n = 7, Sev n = 5; Wilcoxon).

Changes in manuscript:

- Added additional echocardiographic and morphometric parameters for the PAB model.
- Added mitochondrial respirometry data at 2-week and 2-month PAB time points (**Figure 5I**).
- Added flow cytometric analysis of cardiac macrophage populations in PAB mice (**Figure S5H**).
- Added Sirius Red fibrosis histology of PAB RV tissue (**Figure S6I**).
- Clarified in the Discussion that PAB models the hemodynamic consequence of PH (RV pressure overload) while isolating RV-specific effects.

5. There are human bulkRNAseq data from this journal looking at human PAH RV Failure. A discussion/comparison to those data would be useful. Again, PH drives RV failure in most left heart failure so this is a good comparison. In the manuscript, the authors show a very similar trend with mitochondrial derangements/inflammation/fibrosis.

We thank the reviewer for this suggestion. In response, we performed a systematic comparison of our bulk RNA-seq data with the recently published PAH-associated RV failure transcriptomic dataset (Khassafi et al., *Nature Cardiovascular Research* 2023).

To assess transcriptional concordance with PAH-associated RVF, we compared differentially expressed genes (DEGs) between our cohort and the published PAH dataset. In the RVF versus NF comparison, we identified substantial overlap: 235 concordantly downregulated and 214 concordantly upregulated genes between the two datasets (**Figure 1F; Data S5**). Pathway enrichment of concordantly downregulated genes revealed shared suppression of respiratory electron transport, Complex I biogenesis, and aerobic respiration, confirming that mitochondrial transcriptional decline is a conserved feature of RVF regardless of whether the pressure overload originates from pulmonary arterial hypertension or left heart failure (**Figure 1F**). Concordantly upregulated genes were enriched for interferon alpha-beta signaling and antiviral response pathways, indicating a shared inflammatory/innate immune activation signature.

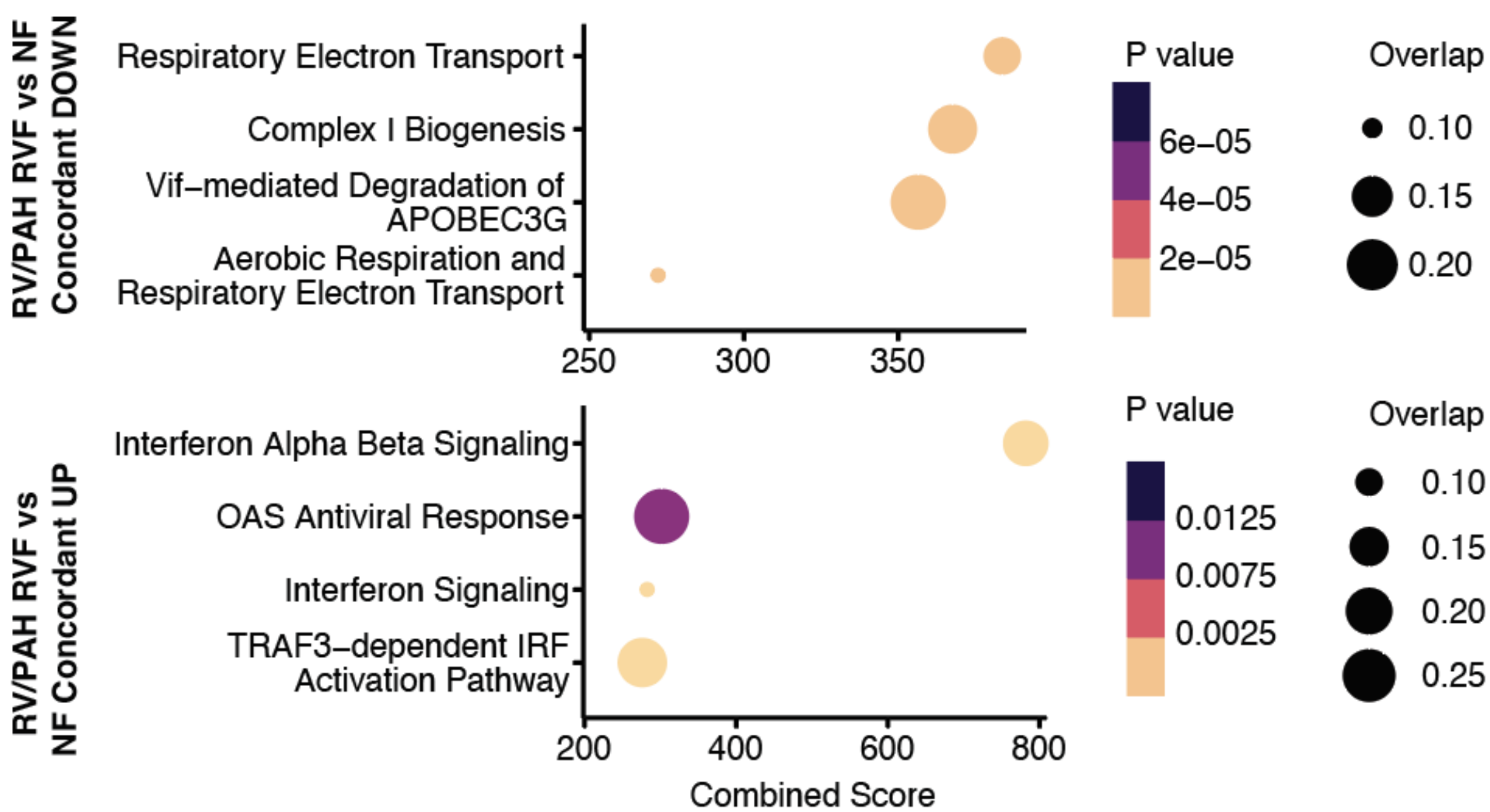

Figure 1F: PAH cross-cohort concordance. Cross-cohort concordance dotplots between cardiomyopathy RVF (RVF vs NF) and pulmonary arterial hypertension for concordantly down- and up-regulated pathways.

We also identified divergent gene sets between the two cohorts (**Figure S1I**). For example, complement genes were downregulated in our cohort but upregulated in PAH. These differences likely reflect the distinct upstream drivers of RVF in PAH (pulmonary vascular disease with genetic/environmental factors) versus LVF-associated RVF (afterload plus underlying cardiomyopathy).

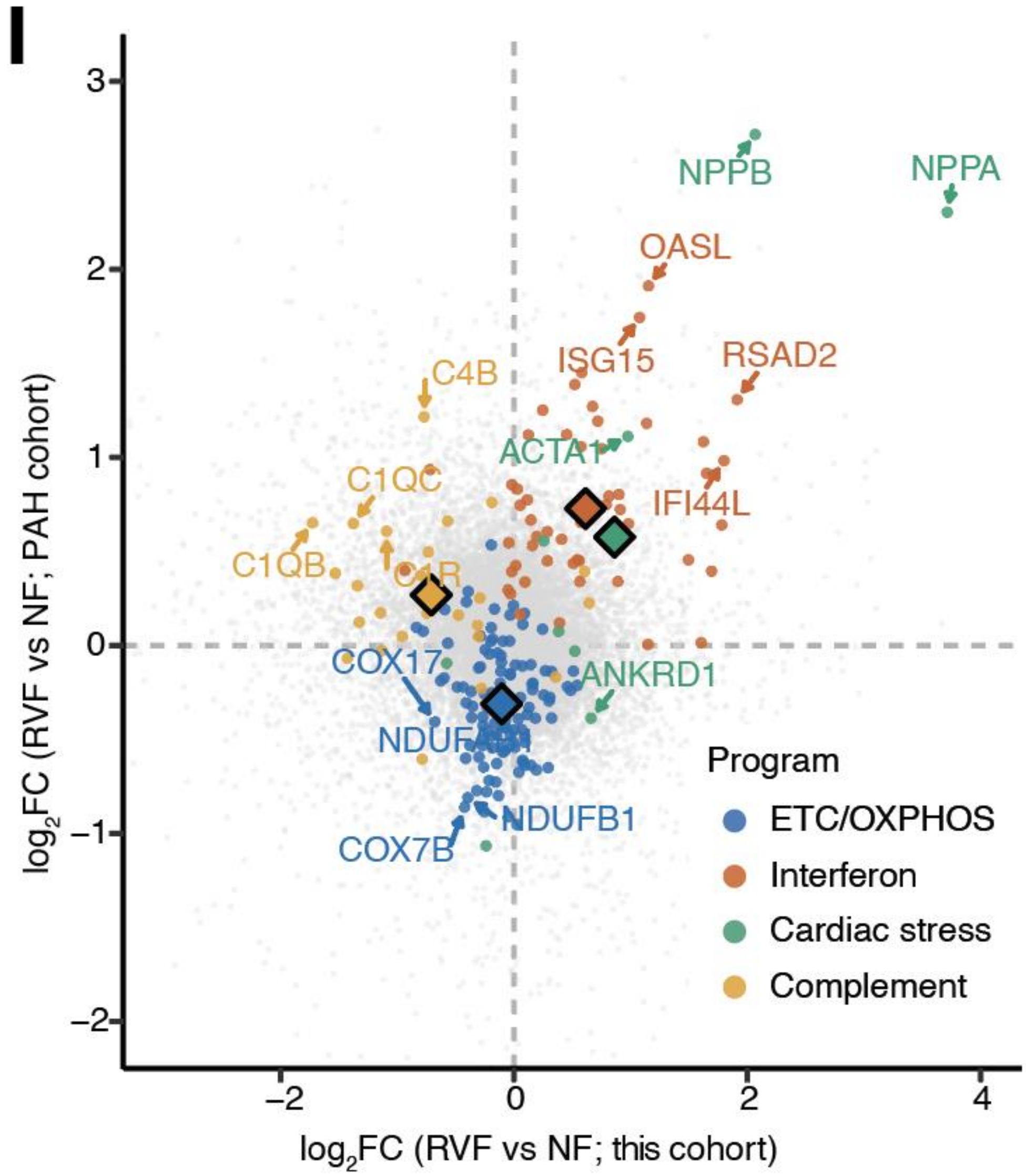

Figure S1I: Cross-cohort comparison of RV-failure transcriptomes. Each gene is plotted by its $\log_2$fold change (RVF vs NF) in our RV cohort versus a PAH-associated RV-failure cohort (GSE240941), colored by membership in four programs (ETC/OXPHOS, interferon, cardiac stress, complement). Diamonds mark program centroids. Interferon and cardiac-stress programs are concordantly up-regulated and ETC/OXPHOS concordantly down-regulated across both cohorts, whereas complement is divergent (down in our RVF, up in PAH), indicating etiology-specific responses. Program gene membership is provided in Data S5.

Changes in manuscript:

- Added **Figure 1F** and **S1I** comparing bulk RNA-seq findings with the PAH RV transcriptomic dataset (Khassafi et al., *Nat Cardiovasc Res* 2023).
- Expanded the Discussion to contextualize shared and divergent transcriptional programs between PAH-associated and LVF-associated RVF.

6. The divergent mitochondrial response in pediatric and adult RVF is quite interesting. Do the authors have any tissue to evaluate mitochondrial respiratory in pediatric RVF.

We thank the reviewer, and we do have pediatric tissue, which allowed us to address this functionally. We also note a correction prompted by re-examining these data: the apparent upregulation of mitochondrial transcripts in the PAB and pediatric RVs in our original submission was an artifact of pooled-nucleus aggregation. On per-sample (pseudobulk) reanalysis with gene-set enrichment, the PAB signal reversed to coordinated suppression and the pediatric signal was statistically insignificant in terms of directionality. We detail this in our response to Reviewer #5 (comment #2) and have corrected it throughout the manuscript.

We performed mitochondrial respirometry on pediatric RV samples (n=7 ped-NF vs n=9 ped-HLHS-palliated failing). In contrast to the adult RVF and murine PAB cohorts, pediatric HLHS-palliated failing RVs showed no significant respiratory deficit at any complex. This functional preservation runs in parallel with the lack of differential change in mitochondrial gene expression we observe in pediatric CMs (unlike adult RVF, where transcripts are suppressed and respiration is impaired). Together, the data place pediatric single-ventricle associated RV failure as a functionally distinct phenotype from adult acquired RVF, consistent with the developmental adaptive capacity of the pediatric heart to long-standing single-ventricle physiology.

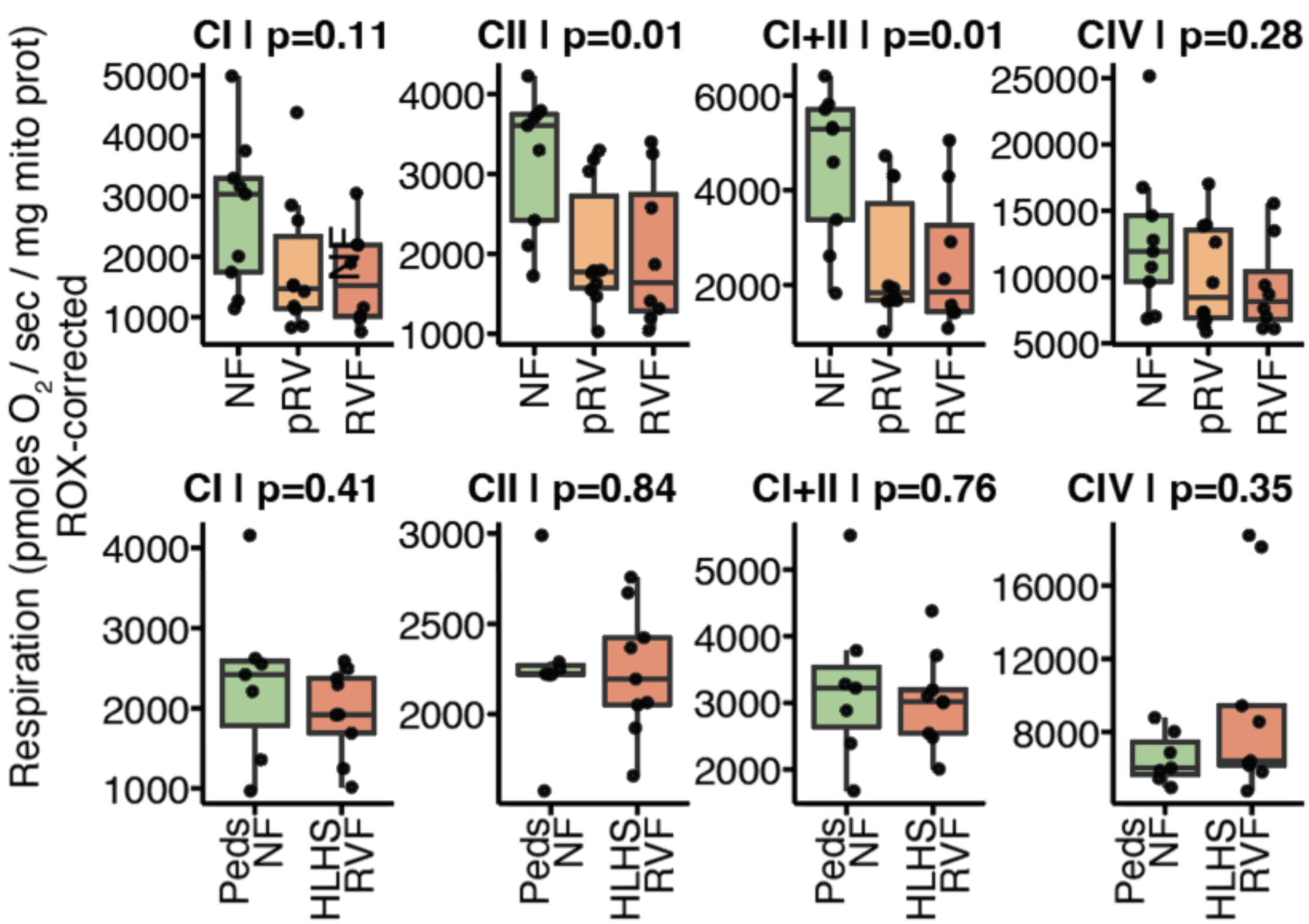

Figure 5H: Adult and pediatric respirometry. Oroboros 2k high-resolution respirometry on freeze/thaw-isolated RV mitochondria, ROX-corrected and normalized to starting

mitochondrial protein mass (pmoles O_2 /sec/mg mitochondrial protein). Each dot is one patient. Adult cohort **(top)**: NF n=9, pRV n=10, RVF n=8; Kruskal–Wallis p-values shown. Pediatric cohort **(bottom)**: pediatric NF (n=7) vs HLHS-palliated failing (n=9); Mann–Whitney pairwise p-values.

Changes in manuscript:

- Added mitochondrial respirometry data for pediatric RV samples.

Minor: Overall, the manuscript does not read that well to be honest. Maybe the authors are trying to put in too much data into one manuscript, but a theme is not well delineated.

We thank the reviewer for this candid feedback. In the revised manuscript, we have reorganized the presentation and substantially revised the text to improve readability and thematic coherence. The revised manuscript is now structured around a central narrative: the progressive, multi-lineage transcriptional remodeling of the right ventricle from compensated pressure overload to overt failure. We have streamlined the presentation of individual datasets to emphasize how each contributes to this overarching theme, improved transitions between sections, and expanded explanatory text where necessary to guide non-specialist readers through the analytical framework.

Reviewer #2 (Remarks to the Author):

Kuznetsov et al. combined a mixture of bulk and single-nucleus (sn) RNA-seq data in order to characterise the changes in gene expression that mark the transition from compensated to decompensated right heart failure (RHF). As the mechanisms of RHF appear to differ from those of the much better studied left heart failure (LHF) and treatment options are limited, identifying RHF-specific mechanisms is a relevant and widely interesting topic.

The authors investigated gene expression in transmural samples from the right ventricle of a large cohort of 142 patients, comprising non-failing controls (NF) and LHF patients with subsequent right heart disease in compensated (preserved, pRV) or failing (RVF) states, using bulk RNA-seq. To account for the expected heterogeneity of gene expression patterns across these samples and conditions, they employed weighted gene network correlation analysis (WGCNA) to identify gene regulatory modules that distinguish disease conditions. They then performed snRNA-seq on a smaller subset of 11 samples from these patients to determine changes in cell types, differentially expressed genes (DEGs) and cell states, followed by spatial transcriptomics on nine samples using the 10x Genomics platform. These data were then compared with snRNA-seq data from a paediatric patient cohort with congenital hypoplastic left heart syndrome (HLHS), as well as with a short-term mouse PAB model.

This tour de force results in the main and supplementary figures reporting a wealth of descriptive bioinformatics data sets that are difficult to follow, particularly for non-specialist readers. Furthermore, the underlying text is highly condensed and lacks necessary explanations of the approaches or rationales for data analysis. It also offers little insight or conclusions derived from the individual parts of the study.

Overall, this study does not meet the standards of a high-quality research paper, nor does it reach conclusions about interesting differentially expressed genes or cell types/cell states that go beyond current knowledge.

We apologize for the difficult access to our manuscript, as indeed we are including large amounts of data into a single manuscript. We have now substantially rewritten and reorganized the manuscript to improve clarity and accessibility (see Response to Editorial Comment 7). In particular, we streamlined the presentation of the bulk RNA-seq, single-nucleus RNA-seq, and spatial transcriptomic analyses, clarified the logical flow of the Results section, and expanded explanatory text where necessary to better guide readers through the analytical framework. We have also expanded the Methods section to include additional technical details for all analyses, including clustering parameters, Xenium reanalysis pipeline, and mitochondrial respirometry protocols (see Response to Editorial Comment 6). We believe these revisions substantially improve the readability and accessibility of the manuscript.

1. Fig. 1B: There appears to be little difference in gene expression between pRV and RVF. This is contrary to what is reported in the literature. Could this be due to patient

treatment with multiple drugs (see Supplementary Table IV)? To what extent does the location of sample biopsies influence gene expression results? The authors need to provide a more detailed explanation of the patient cohort and consider confounding factors in their bioinformatics analysis.

We thank the reviewer for this question. It concerns two distinct comparisons that bear separate treatment: the modest separation between pRV and RVF and the large separation between non-failing and diseased tissue. Medication and sampling affect each differently.

The pRV-versus-RVF similarity is unlikely to be a medication or sampling artifact. The limited separation is reproducible rather than platform- or cohort-specific: it is concordant across our bulk, single-nucleus, and spatial data, and in the bulk cohort, which at n=142 (pRV n=78, RVF n=35) is well powered to resolve modest, consistent group differences, we recover 2,509 differentially expressed genes across NF→pRV but only 5 across pRV→RVF. This asymmetry is the empirical basis for our two-phase model, and it is not unique to our data: in an independent human end-stage heart-failure cohort, Garry et al. (Pulm Circ 2025; PMID 40276472) likewise found compensated and failing RV poorly separated on unsupervised analysis of the global transcriptome (their Figure 2A). Medication is an unlikely source of the similarity. Among medications tabulated (**Tables S3, S5**), only two differed at nominal significance between pRV and RVF: higher thyroid-replacement and lower beta-blocker use in RVF. There were no differences in ACE/ARB, digoxin, diuretic, or milrinone use. Logically, medications that differ between these two groups would be expected to introduce transcriptional differences, not erase them. That the groups nonetheless converge argues for a biological interpretation. We note that mineralocorticoid-receptor antagonists are tabulated within the diuretic category, so balanced diuretic use does not formally establish balanced MRA exposure.

Sampling location is minimized by design. We did not use regional or positional biopsies. Every human sample is a full-thickness transmural RV section taken at explantation. Sampling-region heterogeneity, a recognized variance source in RV studies relying on positional biopsies (particularly those mixing free-wall and septal tissue) is therefore minimized by construction, which if anything increases confidence that the limited pRV-vs-RVF separation reflects biology rather than sampling site.

The medication question bears most directly on the larger non-failing-versus-diseased separation, which we address candidly. Non-failing human RV tissue is available essentially only from organ donors, who are not on chronic heart-failure therapy, whereas both pRV and RVF derive from LVF transplant recipients on guideline-directed medical therapy. Medication exposure, and the donor critical-illness state, is therefore collinear with the NF-versus-diseased contrast, and we are transparent that this is a structural limitation of the tissue source rather than one any analysis fully removes. In particular, we do not claim that surrogate variable analysis resolves it: because SVA preserves the contrast of interest by construction, it cannot disentangle a confound collinear with disease state. Our confidence that the NF-versus-diseased programs

reflect disease biology rests instead on three orthogonal lines of evidence. First, and most directly, our murine pulmonary-artery-banding model carries none of these confounds — inbred animals, an untreated sham control, controlled afterload, no transplant or donor state — and it reproduces specific programs we identify in the human NF→pRV transition: expansion of the endothelial compartment, cardiomyocyte fatty-acid-oxidation decline and fetal-gene induction, and reduced maximal mitochondrial respiration. Programs that recur in an untreated model cannot be artifacts of human medication. The murine model does not reproduce every human program, as the myeloid antigen-presentation response in particular diverges. We restrict this argument to the programs that replicate. Second, the core human signatures are not pharmacological programs of any drug in the cohort: coordinated loss of glucocorticoid-receptor target genes, erosion of resident-macrophage identity, and suppression of nuclear-encoded mitochondrial and oxidative-phosphorylation genes are not effects of beta-blockade, thyroid replacement, RAAS blockade, digoxin, diuretics, or milrinone, and the chronic heart-failure agents that are present (beta-blockers, RAAS inhibitors) promote reverse remodeling, biasing against the failure phenotype rather than generating it. Third, independent human RV-failure datasets with differing treatment regimens recover overlapping disease biology.

Finally, we have expanded the cohort description. Demographic and clinical variables for the bulk and single-nucleus cohorts are now more fully presented in the Results and Methods and summarized in the supplementary tables.

2. Fig. 1C: The authors move straight away into WGCNA analysis without providing a general overview on the primary data. Therefore, it is very difficult to evaluate the quality of the data sets.

We thank the reviewer for this helpful suggestion. In response, we start by reporting results of direct differential expression analyses from pairwise analyses of NF, pRV, and RVF, which are further detailed in the Supplementary Data. This expanded overview of the primary data highlight the overlapping transcriptomic signatures of pRV and RVF and the 276 genes with stepwise changes from NF to pRV and RVF. This clearer overview of the primary data also provides a stronger foundation to support pursuing WGCNA.

3. Fig. 1E: Gene module M29 is missing.

We thank the reviewer for noting this. Fig 1E only displays modules which demonstrated statistically significant differences between disease states. This has been clarified in the legend.

4. Fig. 2: A general overview on data quality (mapped reads, average number of genes / cell, scrublet score number) is missing.

We thank the reviewer for this suggestion. In the revised manuscript we expanded the description of the single-nucleus RNA-seq dataset and added additional quality metrics in the Supplementary Data (e.g. **Data S6**) and in the Figures and associated captions.

Specifically, the revised captions and Methods report total nuclei recovered (61,398 nuclei across 11 patients for the adult cohort; 55,420 nuclei for the murine PAB cohort; 209,604 nuclei for the pediatric cohort), sequencing depth (target 75,000–100,000 reads per nucleus on a NovaSeq 6000 S4), and average detected genes per nucleus (2,912 in the adult cohort). The Methods section also expands the description of the semi-supervised doublet identification strategy used during snRNA-seq preprocessing.

These additions allow readers to more easily evaluate the quality of the single-nucleus dataset.

5. Fig. 2B: Although RYR2 expression in CM is very high, this gene is also expressed in all other cells. It would be more appropriate to choose a marker with higher and more exclusive expression in CM.

While we agree that RYR2 expression can be detected at low levels in other cell types in single-nucleus datasets, RYR2 is widely used as a canonical marker of cardiomyocytes in cardiac single-cell and single-nucleus transcriptomic studies.

For example, RYR2 has been used as a cardiomyocyte marker in several prior studies of human cardiac transcriptomics (e.g., Koenig et al., 2022, human, **Figure 1,** PMID: 35959412; Tucker et al., 2020, human, **Supplement 2, Table IV**, PMID: 32403949; Miao et al., 2024, pig, **Figure 2,** PMID: 38868189). Low-level detection of RYR2 transcripts in non-cardiomyocyte populations is commonly observed in single-nucleus sequencing datasets and is thought to reflect cytoplasmic RNA contamination during nuclear isolation. We further note that we have previously described cardiac endothelial cells express myofibrillar genes (Yucel et. al, 2020, PMID: 33315013), suggesting that some of the observed gene expression may not be artifactual.

Importantly, cardiomyocytes in our dataset show markedly higher RYR2 expression than other cell types, supporting its use as a robust cardiomyocyte marker. We therefore retained RYR2 in the figure.

6. Fig. 2C: The decrease in cardiomyocytes in pRV and RHF is not novel and is also found in LHF. The authors should discuss this literature appropriately (DOI: 10.1038/s41586-022-04817-8).

We thank the reviewer for this helpful suggestion and agree that changes in cardiomyocyte abundance during heart failure have been described in studies of the left ventricle. In particular, single-nucleus transcriptomic studies of failing left ventricles have reported similar reductions in the relative proportion of cardiomyocytes accompanied by expansion of non-myocyte populations (as highlighted by the reviewer).

We have now incorporated discussion of this literature in the revised manuscript. Importantly, our study focuses specifically on the right ventricle, which remains comparatively understudied at the single-cell level. While the observed decrease in

cardiomyocyte representation parallels findings in LV failure, our dataset provides a detailed characterization of these changes across stages of right ventricular remodeling and failure, including comparison of preserved RV function (pRV) and RVF states. These analyses therefore extend previous LV-focused studies and help clarify whether similar cellular remodeling patterns occur in the right ventricle.

7. Fig. 3: The spatial transcriptomic data is difficult to follow. They appear to be integrated from all donors? A much more detailed explanation and visualization is required.

We thank the reviewer for this important feedback. In response, we substantially revised and expanded the spatial transcriptomic analysis to improve clarity, interpretability, and biological insight.

First, we performed a major re-analysis of the Xenium dataset using an updated spatial analysis pipeline (**Figure 3**). Specifically, we reprocessed the data using Proseg for cell segmentation (Baysor was evaluated as an alternative but oversegmented cells), applied SPLIT-based de-mixing to correct for diffusion-associated transcript contamination, performed gene expression imputation using SpaGE, and conducted cell-type annotation using RCTD with the snRNA-seq dataset as a reference. These improvements increased the number of confidently segmented cells and improved spatial assignment of transcripts to individual cells.

Second, we improved the presentation and visualization of the spatial data. We clarified how spatial datasets were handled across samples and expanded figure legends and main text descriptions to better guide the reader through the spatial analyses. We also added additional visualizations illustrating spatial distribution of cell types and niches, as well as improved annotation of spatial clusters.

Third, we expanded the description of spatial niche identification and interpretation. We now provide a more detailed explanation of how spatial niches were defined and how they relate to underlying cell-type composition.

Finally, we integrated the spatial transcriptomic data more directly with the snRNA-seq dataset (e.g. see **Figure 6I, S2E**). In particular, we incorporated ligand–receptor interaction analysis and improved mapping between spatial niches and transcriptional cell states, allowing for a more mechanistic interpretation of spatial organization in RV remodeling.

Together, these revisions substantially improve the clarity and interpretability of the spatial transcriptomic data and strengthen its contribution to the overall study.

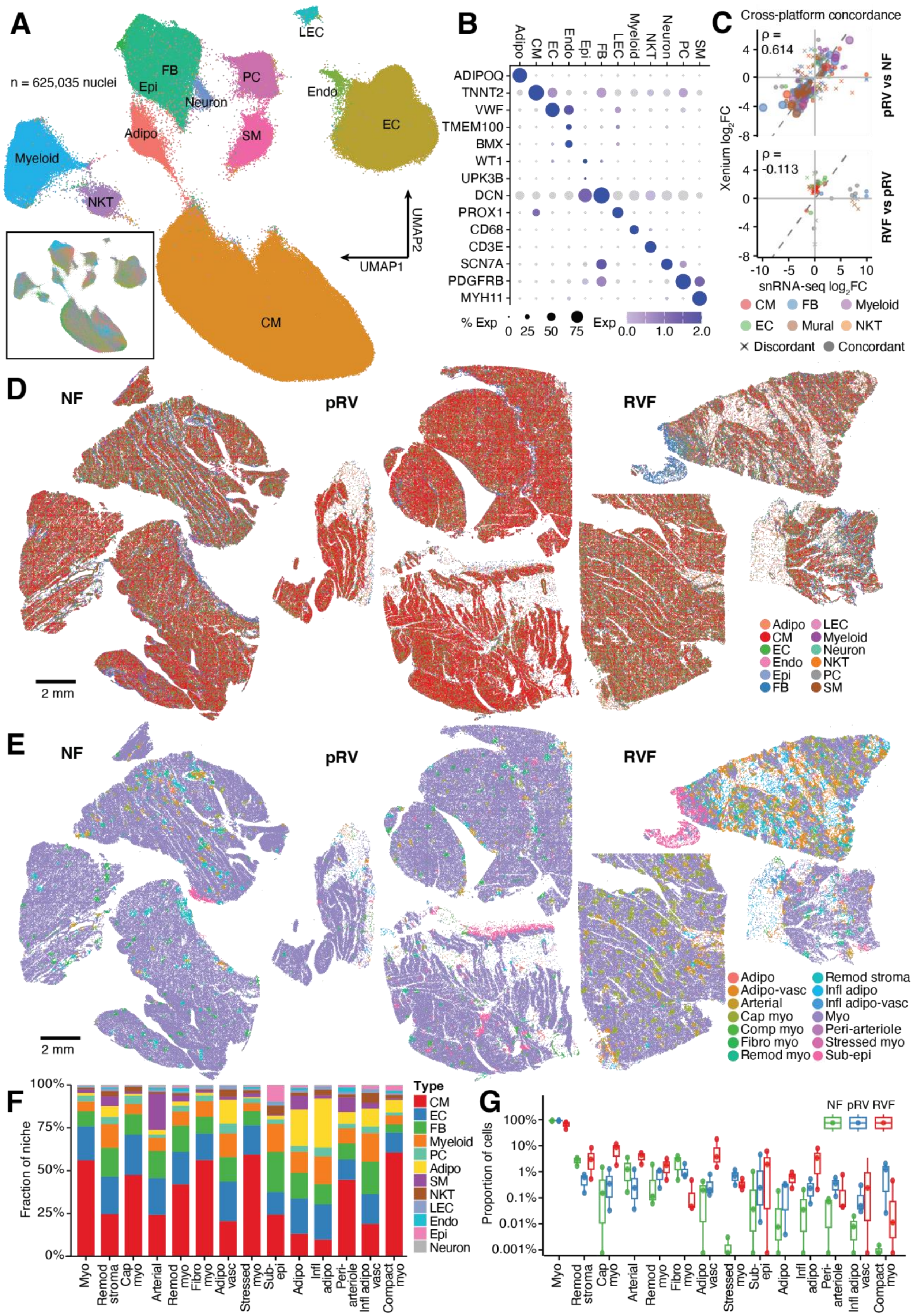
A
n = 625,035 nuclei
LEC
FB
Epi
Neuron
PC
Endo
EC
Adipo
SM
Myeloid
NKT
CM
UMAP1
UMAP2
B
Adipo CM EC Endo Epi FB LEC Myeloid NKT Neuron PC SM
ADIPOQ
TNNT2
VWF
TMEM100
BMX
WT1
UPK3B
DCN
PROX1
CD68
CD3E
SCN7A
PDGFRB
MYH11
% Exp 0 25 50 75
Exp 0.0 1.0 2.0
C
Cross-platform concordance
ρ = 0.614
pRV vs NF
ρ = -0.113
RVF vs pRV
Xenium log2FC
snRNA-seq log2FC
CM FB Myeloid EC Mural NKT
× Discordant ● Concordant
D
NF
pRV
RVF
2 mm
Adipo CM EC Endo Epi FB LEC Myeloid Neuron NKT PC SM
E
NF
pRV
RVF
2 mm
Adipo Adipo-vasc Arterial Cap myo Comp myo Fibro myo Remod myo Remod stroma Infl adipo Infl adipo-vasc Myo Peri-arteriole Stressed myo Sub-epi
F
Fraction of niche
100% 75% 50% 25% 0%
Type
CM EC FB Myeloid PC Adipo SM NKT LEC Endo Epi Neuron
Myo
Remod stroma
Cap myo
Arterial
Remod myo
Fibro myo
Adipo vasc
Stressed myo
Sub-epi
Adipo
Infl adipo
Peri-arteriole
Infl adipo vasc
Compact myo
G
Proportion of cells
100% 10% 1% 0.1% 0.01% 0.001%
NF pRV RVF
Myo
Remod stroma
Cap myo
Arterial
Remod myo
Fibro myo
Adipo vasc
Stressed myo
Sub-epi
Adipo
Infl adipo
Peri-arteriole
Infl adipo vasc
Compact myo

Figure 3. Xenium spatial transcriptomic atlas of the right ventricle. (A) Xenium UMAP of 625,305 cells coloured by 12 lineage clusters; inset shows the same UMAP coloured by patient (n=9) to illustrate donor-consistent integration. **(B)** Dotplot of canonical lineage markers across the 12 Xenium lineages; dot colour encodes mean expression and dot size encodes the percentage of expressing cells. **(C)** Cross-platform concordance scatter of pseudobulk $\log_2$FC: snRNA-seq (x-axis) vs Xenium (y-axis) for matched lineages and contrasts, with points coloured by lineage and symbols separating concordant (●) from discordant (×) calls; Spearman r reported per comparison. **(D)** Spatial maps of all NF, pRV and RVF sections after Proseg resegmentation and RCTD annotation, coloured by lineage. Endocardial border and scale bars indicated where applicable. **(E)** Spatial maps of the same sections coloured by spatial niche assignment, illustrating the spatial organization of the 14 microenvironments resolved by BuildNicheAssay (k=100, CLR-transformed). **(F)** Stacked bar chart of niche composition by lineage (fraction-of-niche), summarizing the cellular identity of each microenvironment. Niche labels are abbreviated as: adipo (adipocyte-rich), adipo-vasc (adipo-vascular), arterial, cap myo (capillary myocardium), comp myo (compact myocardium), fibro myo (fibrotic myocardium), remod myo (remodeling myocardium), remod stroma (remodeling stroma), infl adipo (inflammatory adipocyte), infl adipo-vasc (inflammatory adipo-vascular), myo (myocardium), peri-arteriole, stressed myo (stressed myocardium) and sub-epi (sub-epicardial). **(G)** Niche frequency boxplots across NF, pRV and RVF. Y-axis is showed on a log-scale.

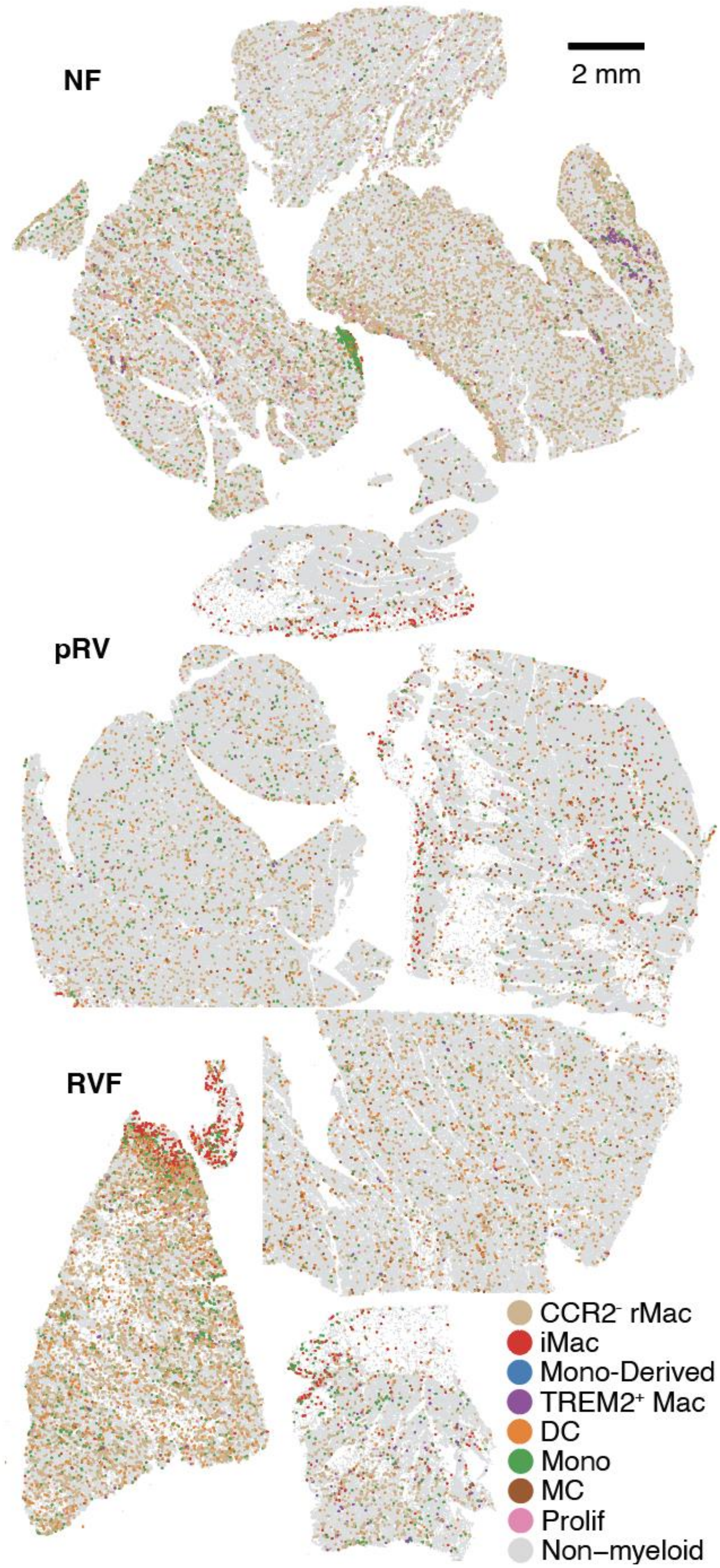
NF
2 mm
pRV
RVF
CCR2⁻ rMac
iMac
Mono-Derived
TREM2⁺ Mac
DC
Mono
MC
Prolif
Non–myeloid

966

Figure 6I: Spatial myeloid localization. Spatial localization of myeloid subtypes in the full Xenium dataset (625,305 cells). Non-myeloid cells are shown in light grey; CCR2^{-} rMacs and Macrophage C1q are combined.

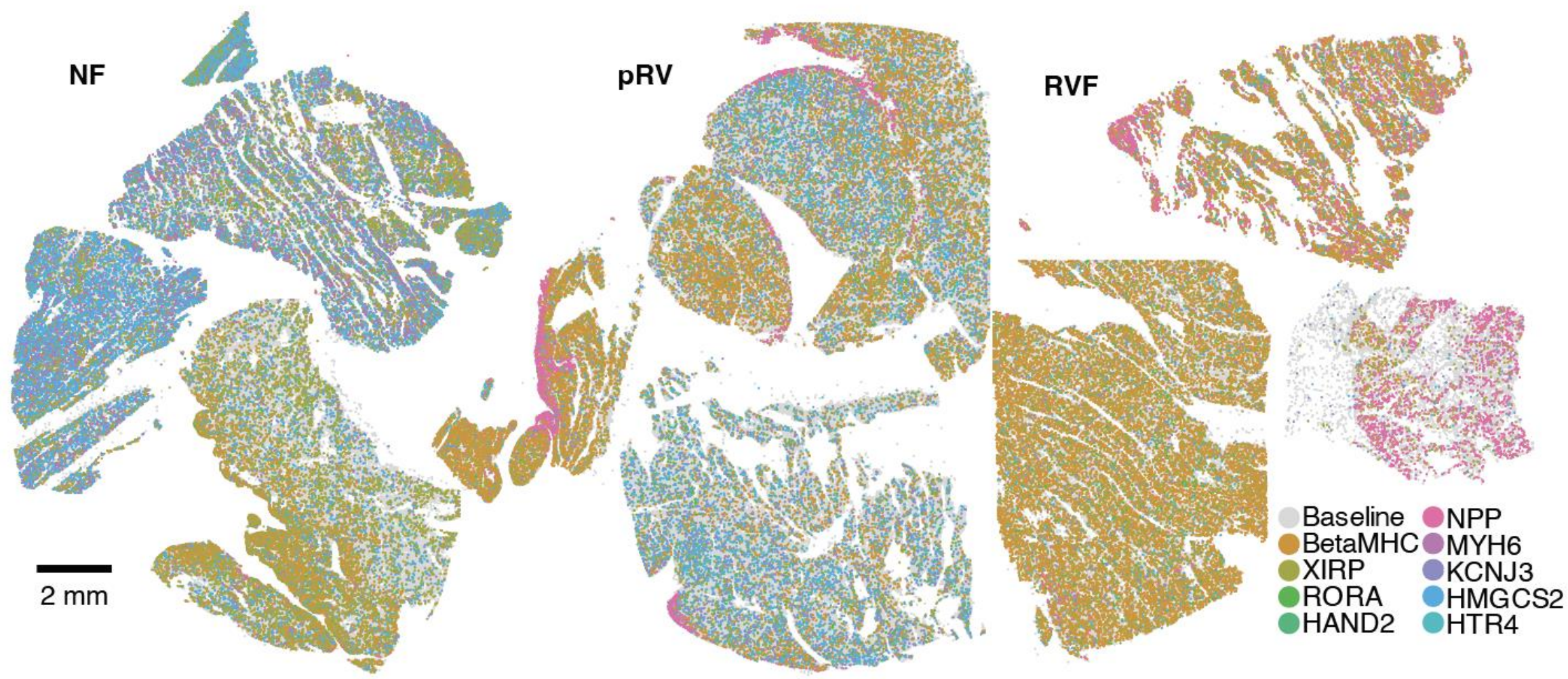

Figure S2E: Spatial distribution of cardiomyocyte subtypes. Spatial distribution of cardiomyocyte subclusters, as visualized on the Xenium spatial data.

8. Fig. 4E: There seems to be not much difference in the regulation of four genes in pRV and RF compared with controls. The authors should provide statistical results.

We thank the reviewer for this observation regarding TNNI3/MYH7/BMPR2/TNNI3K, which is well-founded. Our formal sample-level pseudobulk DESeq2 analysis of the snRNA-seq cardiomyocyte data supports the reviewer's impression: at the CM lineage level, none of the four genes reach significance for NF vs pRV, NF vs RVF, or pRV vs RVF. At the CM-subtype level, only isolated comparisons approach significance (MYH7 and BMPR2).

In the reorganized manuscript, we have therefore replaced the dedicated violin panel for TNNI3/MYH7/BMPR2/TNNI3K with an integrated description in the Results that explicitly distinguishes their statistical status: TNNI3 and TNNI3K are reported as Phase 1 bulk DEGs, while BMPR2 and MYH7 are reported as subthreshold members of module M12 (manuscript Results, M12 description). The complete per-gene pseudobulk DESeq2 statistics (log2 fold-change, p-value, BH-adjusted padj) for all three pairwise contrasts at the CM lineage level are provided in **Data S8** (snRNA-seq coarse-grained, 12 major cell types).

9. Fig. 5: How do the differences between RV and LV compare to similar comparisons from published studies (DOI: 10.1038/s44161-022-00113-w, DOI: 10.1038/s44161-024-00485-1, DOI: 10.1161/CIRCULATIONAHA.119.045401)

We thank the reviewer. To make the cross-chamber comparison explicit, our matched RV-vs-LV contrast is anchored on the failing-LV/DCM snRNA-seq atlas of Koenig et al. (*Nat Cardiovasc Res* 2022; PMID: 35959412), processed at matched modality and resolution (**Figure S4**). Across this comparison, the failing RV and failing LV share most of their transcriptional program — coordinated nuclear-encoded mitochondrial/OXPHOS suppression, cytoskeletal/contractile (M2) remodeling, fibroblast subtype structure and activation, and MHC-II/antigen-presentation induction are all largely conserved across ventricles. The RV-vs-LV differences we resolve are comparatively focal: loss of glucocorticoid-receptor (GR) transactivation-target expression and the absence of inflammasome induction are RV-specific, whereas failing LV myeloid cells retain GR-target expression and show inflammasome induction (**Figure S4I**).

Placing this against the studies the reviewer cites:

Tucker et al. (*Circulation* 2020; PMID 32403949) profiles both ventricles, and it provides the most direct point of comparison. In the non-failing heart, Tucker found the RV and LV to be nearly transcriptionally identical with only 24 differentially expressed genes between the two ventricles, compared with >1,000 DEGs for each atrial-vs-ventricular contrast. Therefore, chamber identity in the healthy heart is dominated by the atrial-vs-ventricular axis, not RV-vs-LV. Our findings are concordant with and extend this: the failing RV and LV likewise share the great majority of their program, so the focal RV-specific features we identify (GR-axis loss, absent inflammasome induction) are disease-emergent rather than reflections of baseline chamber identity, since at baseline the two ventricles differ by only a handful of (sarcomeric) genes. This both validates our approach against an independent four-chamber reference and localizes where the RV genuinely diverges from the LV in disease.

Jurida et al. (*Nat Cardiovasc Res* 2024; PMID 39196177) is a matched RV-vs-LV comparison, the closest model-based analog to our analysis, contrasting the failing RV (rat pulmonary-artery banding, PAB) with the failing LV (aortic banding, AOB), cross-validated in human CTEPH. Its findings align with ours on several axes. At baseline the two ventricles are nearly identical (91% of expressed cardiomyocyte genes shared), mirroring Tucker's 24-gene human RV-vs-LV difference and reinforcing that RV-specific disease programs are not baseline chamber identity. In disease, Jurida finds the RV mounts a distinct and stronger response than the LV (121 RV-specific vs 10 LV-specific deregulated genes in compensation) and defines a conserved rat→human RVF signature of >50 secreted/extracellular-matrix factors. Our fibroblast Phase-2 matricellular program overlaps this signature, which we extend with disease-stage (Phase 1 vs 2) resolution and cell-type assignment. One apparent difference is itself informative: Jurida (and the CTEPH/PAH data it integrates) concludes that right- and left-ventricular failure are largely distinct transcriptional responses, whereas we find that the failing RV and LV share a substantial core program. This most plausibly reflects differences in etiology. Our cohort is predominantly RVF secondary to LVF, in which the RV experiences the same cardiomyopathic and systemic milieu as the LV, whereas the PAB/CTEPH model isolated RV afterload. The same etiology axis underlies the RV-specific divergence we observe

against the Khassafi et al. PAH RV dataset (*Nat Cardiovasc Res* 2023; PMID 39196250; used in **Figure 1F & Figure S1I**).

Boucherat et al. (*Nat Cardiovasc Res* 2022; PMID 39196085) is the one cited study that does not make an RV-vs-LV comparison: it profiles RV tissue only (donor control, compensated and decompensated PAH RV) integrated with plasma proteomics. There is no LV arm, and its comparisons are RV disease-stage and RV-tissue-vs-plasma. It identifies LTBP-2, a secreted matrix protein, as a circulating biomarker of RV dysfunction in PAH. We relate to it on the RV-failure/biomarker axis: the fibrosis-related RV transcripts in our atlas (and Boucherat's own RV matrix proteins: LTBP-2, COL6A3, COL18A1, TNC) are candidate tissue correlates of that circulating signal.

Together, the matched human cross-chamber comparison localizes the RV-specific features. Two independent RV-vs-LV references, the four-chamber human atlas (Tucker) and the rat PAB-vs-AOB model (Jurida), both show the ventricles are near-identical at baseline, so these features are disease-emergent and the etiology-resolved comparisons (Jurida/CTEPH, Khassafi/PAH, Boucherat/LTBP-2) indicate that LVF-secondary RV failure shares more with LVF than isolated-afterload RV failure does, while converging on a conserved fibrotic program.

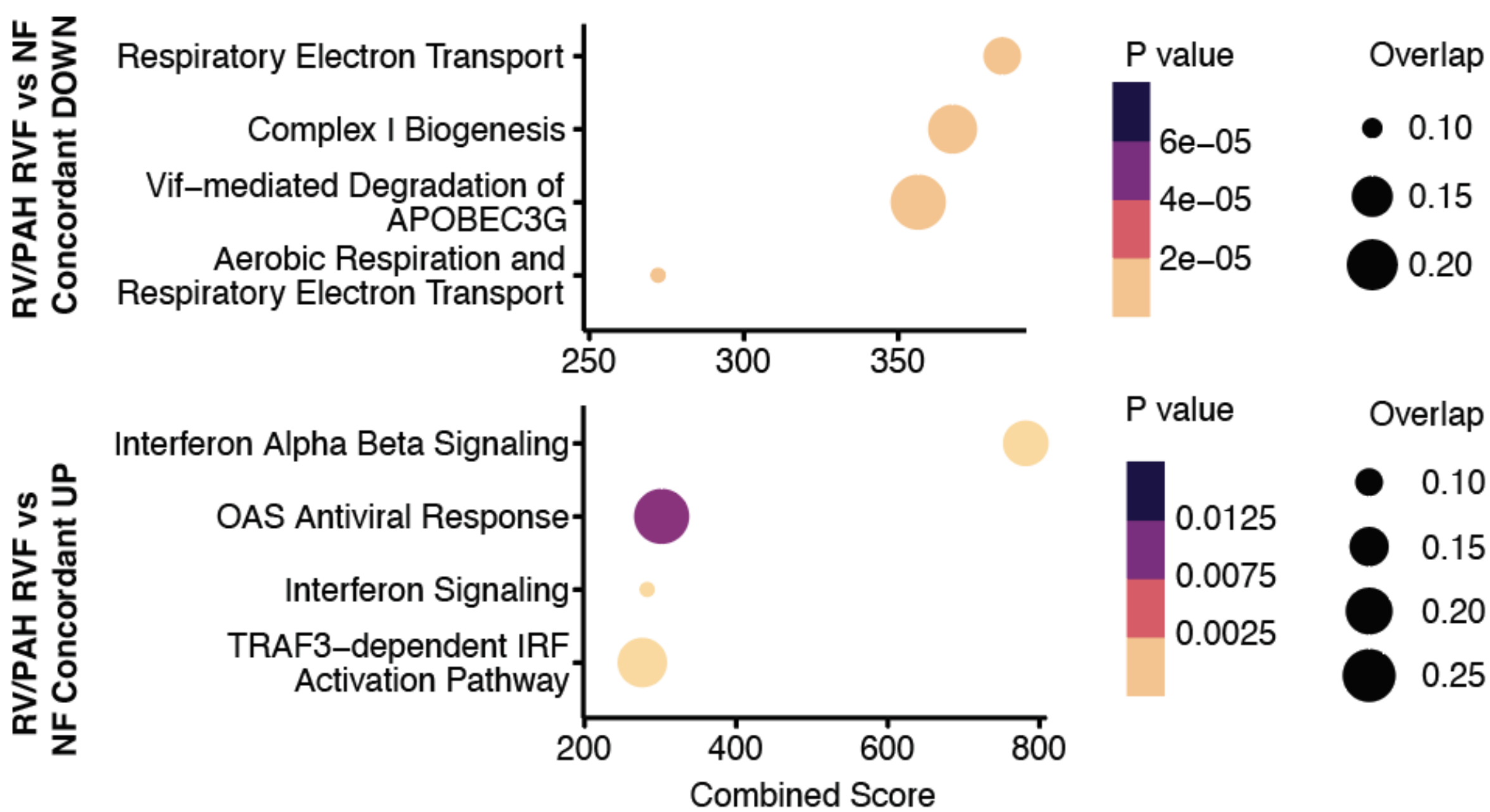

Figure 1F: PAH cross-cohort concordance. Cross-cohort concordance dotplots between RVF (RVF vs NF) and pulmonary arterial hypertension for concordantly down- and up-regulated pathways.

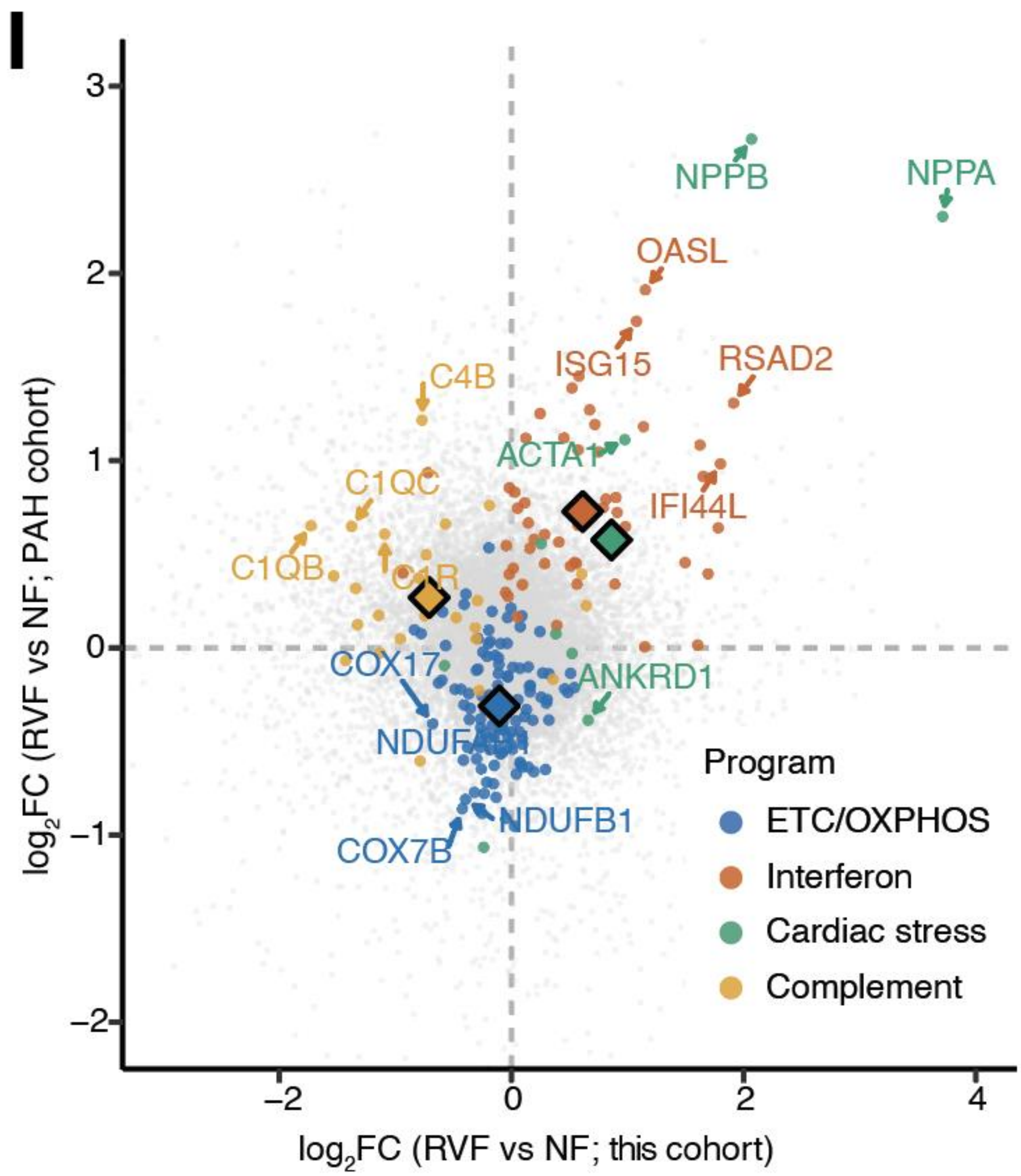

Figure S1I: Cross-cohort comparison of RV-failure transcriptomes. Each gene is plotted by its log_2fold change (RVF vs NF) in our RV cohort versus a PAH-associated RV-failure cohort (GSE240941), colored by membership in four programs (ETC/OXPHOS, interferon, cardiac stress, complement). Diamonds mark program centroids. Interferon and cardiac-stress programs are concordantly up-regulated and ETC/OXPHOS concordantly down-regulated across both cohorts, whereas complement is divergent (down in our RVF, up in PAH), indicating etiology-specific responses. Program gene membership is provided in Data S5.

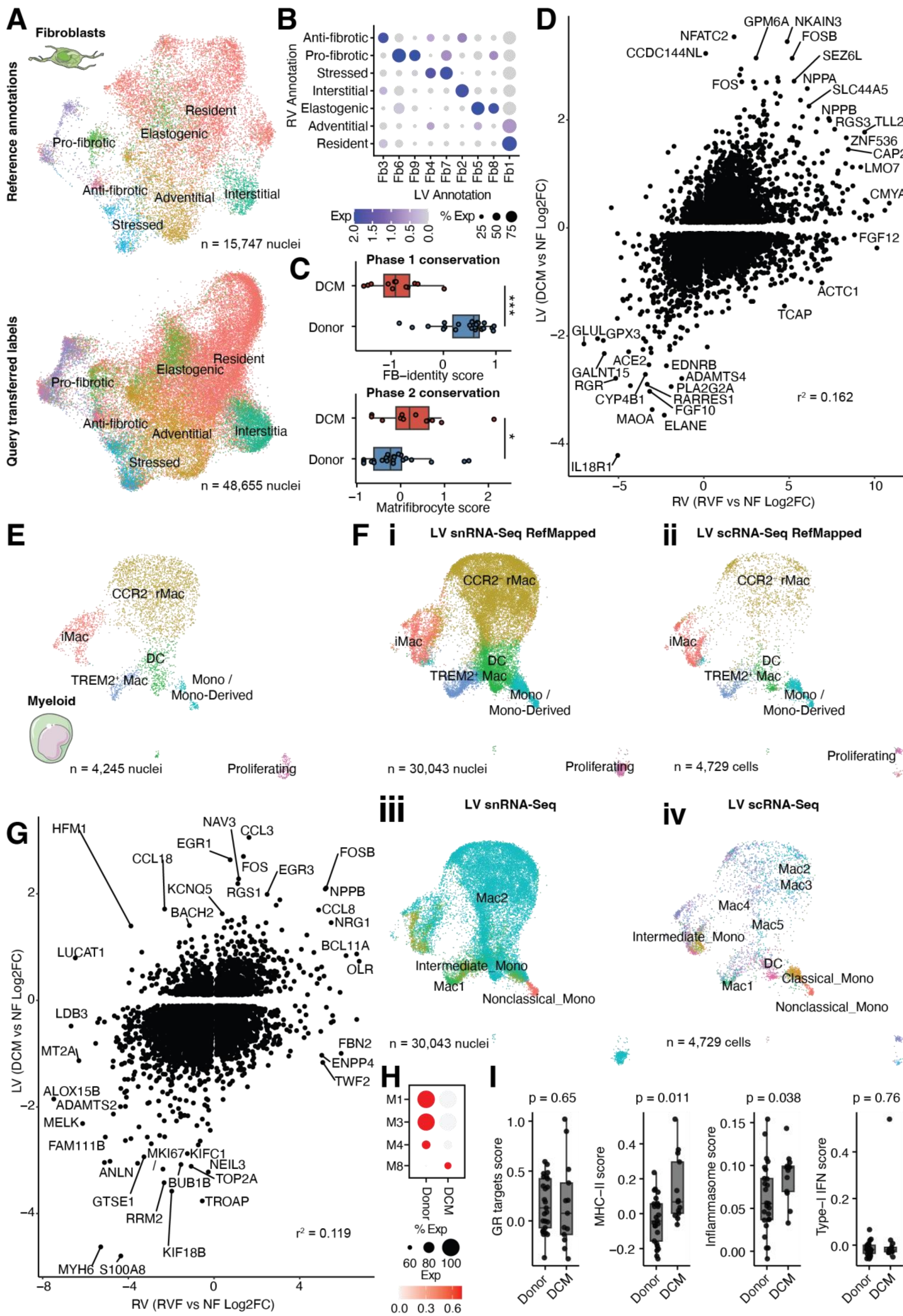
A
Fibroblasts
Reference annotations
Resident
Pro-fibrotic
Elastogenic
Anti-fibrotic
Adventitial
Interstitial
Stressed
n = 15,747 nuclei
Query transferred labels
Resident
Elastogenic
Pro-fibrotic
Anti-fibrotic
Adventitial
Interstitia
Stressed
n = 48,655 nuclei
B
RV Annotation
Anti-fibrotic
Pro-fibrotic
Stressed
Interstitial
Elastogenic
Adventitial
Resident
Fb3 Fb6 Fb9 Fb4 Fb7 Fb2 Fb5 Fb8 Fb1
LV Annotation
Exp
2.0 1.5 1.0 0.5 0.0
% Exp
25 50 75
C
Phase 1 conservation
DCM
Donor
***
FB-identity score
Phase 2 conservation
DCM
Donor
*
Matrifibrocyte score
D
LV (DCM vs NF Log2FC)
RV (RVF vs NF Log2FC)
GPM6A NKAIN3
NFATC2
FOSB
CCDC144NL
SEZ6L
FOS
NPPA
SLC44A5
NPPB
RGS3 TLL2
ZNF536
CAP2
LMO7
CMYA5
FGF12
ACTC1
TCAP
GLUL
GPX3
ACE2
GALNT15
RGR
EDNRB
ADAMTS4
PLA2G2A
CYP4B1
RARRES1
FGF10
MAOA
ELANE
IL18R1
r² = 0.162
E
CCR2⁻ rMac
iMac
DC
TREM2⁺ Mac
Mono / Mono-Derived
Myeloid
n = 4,245 nuclei
Proliferating
F i
LV snRNA-Seq RefMapped
CCR2⁻ rMac
iMac
DC
TREM2⁺ Mac
Mono / Mono-Derived
n = 30,043 nuclei
Proliferating
ii
LV scRNA-Seq RefMapped
CCR2⁻ rMac
iMac
DC
TREM2⁺ Mac
Mono / Mono-Derived
n = 4,729 cells
Proliferating
iii
LV snRNA-Seq
Mac2
Intermediate_Mono
Mac1
Nonclassical_Mono
n = 30,043 nuclei
iv
LV scRNA-Seq
Mac2
Mac3
Mac4
Mac5
Intermediate_Mono
DC
Classical_Mono
Mac1
Nonclassical_Mono
n = 4,729 cells
G
LV (DCM vs NF Log2FC)
RV (RVF vs NF Log2FC)
HFM1
NAV3
CCL3
EGR1
CCL18
FOS
EGR3
FOSB
KCNQ5
RGS1
NPPB
BACH2
CCL8
NRG1
BCL11A
LUCAT1
OLR
LDB3
FBN2
MT2A
ENPP4
TWF2
ALOX15B
ADAMTS2
MELK
FAM111B
MKI67
KIFC1
NEIL3
ANLN
TOP2A
BUB1B
GTSE1
TROAP
RRM2
KIF18B
MYH6
S100A8
r² = 0.119
H
M1
M3
M4
M8
Donor
DCM
% Exp
60 80 100
Exp
0.0 0.3 0.6
I
p = 0.65
GR targets score
p = 0.011
MHC-II score
p = 0.038
Inflammasome score
p = 0.76
Type-I IFN score
Donor
DCM

Figure S4. Left ventricular failure (DCM) comparison. (A) UMAP of integrated human RV fibroblast lineages and cell sub-types **(top)**, as well as reference mapped LV data **(bottom)**. **(B)** Fibroblast subtype concordance between ventricles. **(C)** Conservation of fibroblast Phase 1 and Phase 2 programs in LV snRNA-seq (Koenig et al. 2022; Donor vs DCM, per-patient pseudobulk; CPM, log1p, per-gene z-scored across patients, mean across genes in module). **(top)** Phase 1: Koenig donor-FB identity score (*GPX3, PID1, TGFBR3, ACSM3, APOD*; expected to erode, i.e. down in DCM). **(bottom)** Phase 2: Fu 2018 matrifibrocyte commitment score (*COMP, CILP, CHAD*; expected to rise, i.e. up in DCM). Each dot is one patient; box = IQR, whiskers = 1.5× IQR. Donor vs DCM tested by Wilcoxon rank-sum. * $p < 0.05$; ** $p < 0.01$; *** $p < 0.001$. **(D)** Scatter plot of $\log_2$ fold changes for the human LV fibroblast (DCM vs NF) vs human RV fibroblast (RVF vs NF) comparison by gene. Fold changes are well correlated between datasets. **(E)** UMAP of integrated human RV myeloid lineages and cell sub-types. **(F)** Reference mapping of single-nuclei **(i)** and single-cell **(ii)** LV myeloid dataset onto human RV dataset with corresponding RV annotations. Original LV annotations are also shown for single-nuclei **(iii)** and single-cell **(iv)** LV data. **(G)** Cross-chamber concordance scatter for the four myeloid bulk-WGCNA modules (M1, M3, M4, M8). For each module, per-gene $\log_2$FC was computed by Seurat FindMarkers in the RV myeloid object and in the LV (Koenig 2022) myeloid object, restricted to genes detected on both platforms. Notable genes are labeled. **(H)** Bulk-WGCNA myeloid modules (M1, M3, M4, M8) projected onto the LV (Koenig 2022) myeloid atlas via Seurat AddModuleScore. Dot plot of mean per-cell module score (color) and percent expressing (size) by LV myeloid subtype × disease state (Donor vs DCM), allowing the four RV-derived myeloid programs to be assessed for cross-chamber conservation. **(I)** Per-patient pseudobulk module-score box plots in LV myeloid cells snRNA-seq for the four RV-derived myeloid programs: GR transactivation, MHC-II/antigen presentation, NLRP3 inflammasome, and type-1 interferon. Each dot = one patient (per-patient mean module score over LV myeloid nuclei). Donor vs DCM tested by Wilcoxon rank-sum.

10. Fig. 6: Other studies have also reported an inflammatory component and an altered immune cell compartment. In my view, the interpretation of the glucocorticoid receptor target gene sets is an oversimplification and incorrect. An activated glucocorticoid receptor can bind to either a positive or negative glucocorticoid response element (GRE) in the genome, thereby inducing or repressing gene expression. Therefore, downregulation of genes with a proven or predicted GRE is not evidence of downregulation of the glucocorticoid system (see DOI: 10.1136/ard-2023-225371 for a recent review on this topic).

We thank the reviewer for this important conceptual point and agree that our original interpretation was overly simplistic. The glucocorticoid receptor can indeed bind to both positive and negative GREs, resulting in either transcriptional activation or repression depending on the specific genomic context. Accordingly, we have revised the manuscript to clarify that the observed downregulation of GRE-associated genes should not be interpreted as direct evidence of reduced glucocorticoid receptor signaling. Instead, we now describe this observation more conservatively as a relative reduction in the expression of genes previously identified as glucocorticoid-responsive, which may reflect

altered glucocorticoid signaling, altered transcriptional context, or other regulatory mechanisms.

Importantly, our expanded pseudobulk DESeq2 analysis at the sub-cell-type level identified FKBP5, a direct transcriptional target and negative-feedback regulator of the glucocorticoid receptor, as significantly downregulated in 11 of 24 non-cardiomyocyte subtypes tested in pRV versus NF (Phase 1; CM uniquely attenuated; **Data S9**). This tissue-wide loss of FKBP5 provides more specific evidence for altered glucocorticoid signaling in RVF than the module-level GRE enrichment analysis presented in the original manuscript, and is discussed in the revised text.

Changes in manuscript:

- Revised interpretation of GRE-associated transcriptional signatures throughout the manuscript.
- Added FKBP5 pan-tissue downregulation as evidence for altered glucocorticoid signaling.

11. Fig. 8: The rationale for including the pediatric samples is unclear and there is no firm conclusion derived from the combined analysis.

The rationale for including the pediatric cohort is to determine whether the transcriptional programs identified in adult RVF are generalizable to RVF arising from a fundamentally different etiology, congenital single-ventricle physiology in hypoplastic left heart syndrome (HLHS). This comparison is clinically relevant because pediatric RVF in the setting of HLHS remains a major cause of morbidity and mortality with limited therapeutic options.

In the revised manuscript, we have clarified this rationale and strengthened the comparative analysis. We now highlight several key findings from the pediatric comparison: (1) shared features with adult RVF, including myeloid MHCII induction and endothelial cell expansion; and (2) important divergences, including no transcriptional change (rather than downregulation) of nuclear-encoded mitochondrial genes in pediatric cardiomyocytes, preserved (rather than reduced) mitochondrial respiratory capacity, and venous (rather than capillary) EC expansion. These shared and divergent features are now discussed in the context of a two-phase model of RV remodeling that may apply across etiologies.

12. Fig. 8D: Coefficients of correlation are missing

We thank the reviewer for pointing this out. Correlation coefficients have now been added to the corresponding figure panels.

13. Introduction: Key literature on RH disease is missing (e.g. DOI: 10.1056/NEJMra2207410)

We thank the reviewer for this suggestion. We have incorporated the referenced review and additional key literature on right heart disease into the Introduction.

14. Page 4, lines 146 – 148: Where in Fig 3E are the known marker genes, spatial niches and cell types are located? This observation needs to be described in much more detail in the Results and Legends.

We thank the reviewer for this feedback. In the revised manuscript, the spatial transcriptomic analysis has been substantially expanded with updated figures that more clearly identify cell types, spatial niches, and marker gene expression within the Xenium data. Figure legends have been expanded to describe the spatial features highlighted in each panel.

15. Page 4, lines 155-156: What does this mean: …Cross referencing these clusters with spatial niches demonstrated spatial pattering (Fig. S2G)?

We agree this sentence was unclear. We have revised the text to more clearly explain that spatial niche analysis revealed non-random spatial organization of cell types, with specific cell-type combinations co-localizing in distinct tissue regions. The revised manuscript provides a more detailed description of the niche identification methodology and the biological interpretation of the observed spatial patterns.

16. Page 4, line 166: What are "nuclear synthesized genes"?

We agree this terminology was imprecise. The intended meaning was nuclear-encoded mitochondrial genes, genes encoded in the nuclear genome that produce proteins targeted to the mitochondria. We have corrected this terminology in the revised manuscript.

17. Page 5, line 228-229: What is the contribution of patient treatments to the M1 set of glucocorticoid-regulated genes? What is a pseudo-steroid deficient state?

We thank the reviewer for this important question. Importantly, none of the patients in our cohort were receiving exogenous glucocorticoid therapy at the time of tissue collection. **Table S3** and **Table S5** list other relevant medications. Using nominal p values, RVF patients were more likely to be on thyroid replacement therapy and less likely to be on a beta blocker. There were no differences in the use of ACE/ARB, digoxin, diuretics, or milrinone. MRAs, like spironolactone, were grouped under the diuretic category. While we cannot rule out indirect effects of other medications on glucocorticoid-responsive gene expression, the absence of exogenous steroid use in our cohort supports the interpretation that the observed transcriptional changes reflect disease-associated rather than treatment-related alterations.

Regarding terminology, we have revised the manuscript to avoid the term "pseudo-steroid deficient state," which we agree may be misleading. Instead, we now describe this finding as a tissue-wide reduction in glucocorticoid-responsive gene expression, most clearly exemplified by the pan-tissue downregulation of FKBP5 (a direct GR target gene), which suggests altered glucocorticoid signaling in the failing right ventricle. We have also

softened the interpretation to acknowledge that this observation is hypothesis-generating and that the direction of glucocorticoid receptor activity (activation vs. repression) cannot be inferred from target gene expression alone.

18. Page 7, lines 308-310: What is the evidence to suggest that the mild RVF group may be protected?

We agree with the reviewer that the term "protected" was speculative and not supported by the data. We have revised the manuscript to use more neutral language, describing the mild RVF group as exhibiting transcriptional signatures that more closely resemble the compensated (pRV) state rather than characterizing these as "protective."

19. Page 10, line 429: …, we also show upregulation…(missing word)

We thank the reviewer for catching this. The missing text has been corrected in the revised manuscript.

20. Page 10, line 434: What are pseudo-steroid resistant CCR2- rMacs?

We agree this terminology was unclear. As discussed in our response regarding the glucocorticoid findings, we have revised the manuscript to avoid the term "pseudo-steroid resistant." This population is now described as CCR2- resident macrophages exhibiting reduced expression of glucocorticoid-responsive genes, including FKBP5.

21. Page 11, lines 487-490: This is a meaningless and trivial conclusion.

We thank the reviewer for this feedback. We have removed this concluding statement and replaced it with a more substantive summary that highlights the key translational implications of the study.

Reviewer #3 (Remarks to the Author):

In their work, Kuznetsov et al. provide a transcriptomics atlas of right ventricular failure (RVF) progression in adults on the bulk, single-nucleus, and spatially resolved cellular levels. They identify several prominent changes, including a decrease in nuclear-encoded mitochondrial transcript expression in cardiomyocytes, increased expression of transcripts related to antigen presentation in myeloid cells, and an expansion of the endothelial cell compartment in the diseased samples. They compare their single-nucleus data to a mouse model of RVF, pediatric HLHS, and left ventricular failure (LVF), and provide a detailed landscape of shared and distinct characteristics between these conditions on the transcriptome level.

The manuscript presents a rich and multifaceted dataset, generated using multiple technological platforms and derived from complementary sample types, addressing a disease condition that has been understudied at the molecular level. As such, the data and analyses presented are highly relevant and are likely to attract interest within the cardiovascular field. The analysis is thorough, and the conclusions are, for the most part, well-supported by the accompanying figures and tables. Nonetheless, I have several general observations and comments (detailed below) that I believe should be addressed by the authors.

Overall, the authors were quite cautious in contextualizing their findings and in proposing hypotheses to explain the observed transcriptomic differences between disease stages or experimental models. While it is understandable that the study aims to serve as a foundation for future hypothesis generation, the inclusion of some author-driven interpretations or speculative hypotheses in the discussion would help better illustrate the study's potential and guide subsequent research.

Additionally, while the analyses of the bulk and single-nucleus datasets are clearly described and well-integrated into the study, the spatial transcriptomics dataset (Xenium) appears underutilized. Its contribution to the central findings of the study remains unclear, as it is neither mentioned in

The code is well organised overall and would be easy to reproduce with access to the study data. The authors used state-of-the-art R packages/methods for most of the analysis, with some great additions. The scripts being separated per figure and per panel make it very easy to follow through the manuscript. Some comments to allow for even better reproducibility are also provided below.

Major comments:

1. Please include the number of nuclei represented in all UMAPs, both within the figures and their corresponding legends. If the UMAPs display data from different experimental groups, provide versions of the plots with embeddings that highlight these groupings (as shown in Fig. S8A), to illustrate the effectiveness of data integration and to reveal any sample- or group-specific patterns.

We thank the reviewer for this suggestion. In the revised manuscript, the major lineage-integration UMAPs now explicitly report the total number of nuclei or cells represented (e.g., **Figure 2A**: 61,398 adult RV nuclei; **Figure 3A**: 625,305 Xenium cells; **Figure 8A**: 209,604 pediatric and 61,398 adult nuclei in co-embedding; **Figure S5A**: 55,420 mouse PAB nuclei). Subclustering UMAPs (CM, FB, EC, myeloid, mural) inherit these totals from the parent lineage panel; the corresponding subtype-level nuclei counts are tabulated in **Data S6 and S7**. In addition, for the snRNA-seq integration we added a patient-colored inset (**Figure 2A**) to illustrate donor-consistent integration.

2. Please consider providing dot plots of differentially expressed genes (DEGs) between the nuclear subclusters along with or instead of the currently included marker score enrichment. The former would be considerably more informative and easier to interpret than the complete DEG lists included in the manuscript, particularly for cell states that do not fall into biologically well-defined categories, such as cardiomyocyte subclusters. For endothelial and myeloid subclusters, please also include plots of key marker gene expression to support the provided annotations. Additionally, please expand the methods part of the manuscript with describing the annotation strategy of single-nucleus and Xenium-based cellular clusters.

We thank the reviewer for this excellent suggestion. In the revised manuscript, we have added dot plots of marker genes supporting the annotation of cardiomyocyte, endothelial, fibroblast, and myeloid subclusters (e.g. **Figure 6B**). These visualizations substantially improve interpretability of the clustering results and directly address the reviewer's request for clearer annotation support.

We have also expanded the Methods section to describe the annotation strategy for both the snRNA-seq and Xenium datasets. For the snRNA-seq data, subclusters were annotated based on marker gene expression identified by FindAllMarkers, cross-referenced with published cardiac cell atlases. For the Xenium spatial data, cell-type annotation was performed using RCTD with the snRNA-seq dataset as a reference, followed by subclustering within each cell type using Harmony-corrected embeddings to mitigate patient-level batch effects. Subcluster annotations were assigned using the same marker-based approach as the snRNA-seq analysis.

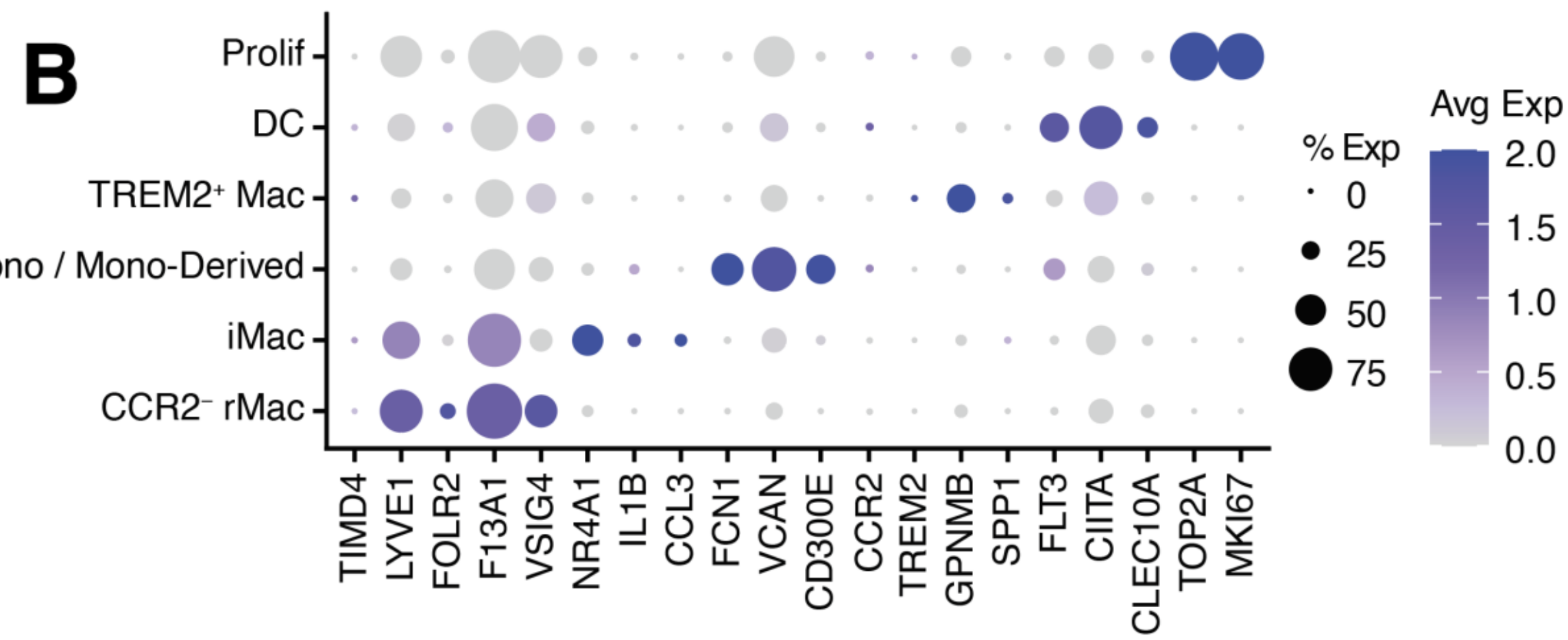

Figure 6B: Myeloid subtype marker genes. Dot plot of canonical marker genes per myeloid subtype. Mean expression (color) and percent of cells expressing (size).

3. In lines 90–92, the authors state that the majority of transcriptomic changes occur during the NF-to-pRV transition, with considerably fewer changes observed in the subsequent pRV-to-RVF transition. This suggests that many of the molecular alterations may be independent of RV functional decline. Given this, it remains unclear how the pronounced functional deterioration that characterizes the pRV-to-RVF transition is mediated at the molecular level. Do the authors have any insights or hypotheses regarding this?

We thank the reviewer for this thoughtful question, which prompted us to interpret the two-phase asymmetry more rigorously.

We first emphasize that the relative paucity of pRV→RVF transcriptional change is a reproducible biological feature of the data, not a sampling artifact. The asymmetry is concordant across all three platforms, including the bulk cohort, which at n=142 (pRV n=78, RVF n=35) is well powered to detect even modest, consistent whole-tissue shifts, yet recovers only 5 lineage-level DEGs across the pRV→RVF transition versus 2,509 across NF→pRV. That the most highly powered modality shows the most pronounced quiescence argues against limited statistical power as the explanation. We now state explicitly that ~95% of platform-resolved differential expression is front-loaded into the NF→pRV transition.

A low genome-wide DEG count does not, however, imply an absence of molecular drivers of decompensation. Rather, the Phase-2 program is focal and lineage-restricted, and is therefore diluted in whole-tissue and whole-lineage averaging. This is evident within the data: single-nucleus profiling, despite a far smaller cohort (n=11), recovers more Phase-2 DEGs than the genome-wide bulk analysis (61 versus 5 lineage-level), and spatial profiling, with its substantially larger cell counts, recovers a still higher per-gene detection rate at this transition. The relationship between cohort size and Phase-2 recovery thus runs opposite to what a power-limitation model would predict, and is instead the expected

signature of a cell-type-restricted program resolved only with cellular and spatial resolution. We accordingly frame the compensated-to-decompensated transition as a transcriptional set-point shift followed by focal, lineage-specific programs.

These Phase-2 programs are themselves coherent candidate mediators of functional deterioration, even where the aggregate count is modest. In cardiomyocytes, decompensation engages a BMP/TGF-β superfamily program (BMPR2, BMPR1A, FSTL3, BAMBI) superimposed on a progressive, broadly consistent decline of nuclear-encoded oxidative-phosphorylation modules (M10/M25/M26), the latter with a functional correlate in the reduced maximal respiration we measure in adult RVF. In the endothelium, Phase 2 resolves as an angiogenic-destabilization and EndMT program (ANGPT2, MYC, ADAMTS1, SNAI1) with erosion of capillary identity (CA4), validated cross-platform. In fibroblasts, matrifibrocyte commitment (COMP, CILP, CHAD) rises specifically at this transition, focal to activated/matricellular subsets, against a backdrop of cumulative inflammatory induction in the myeloid compartment. Vascular destabilization, matrix maturation, and continued metabolic decline thus provide a plausible molecular basis for the functional deterioration of decompensation that a simple genome-wide DEG count does not capture.

Finally, our transcriptomic approach cannot capture changes in translation or post-translational modification, which may contribute disproportionately to the pRV→RVF functional transition relative to the initial molecular response and which would be invisible to the analyses presented here.

4. How do the authors interpret the differentially expressed genes identified between pRV and RVF in the bulk RNA-seq data, as described in lines 101–108? Notably, SLITRK5 and GRIN2B are typically enriched in neuronal cell types - does this imply a change in the abundance of such cells between the two conditions? I would also question the relevance of SLITRK5 expression in AV node cells, as mentioned by the authors, in the context of this analysis.

We thank the reviewer for this question. SLITRK5 and GRIN2B are indeed primarily expressed in neuronal populations. In our snRNA-seq dataset, these genes are enriched in the neuronal cluster, consistent with their known expression profiles. Their differential expression between pRV and RVF in the bulk RNA-seq data therefore likely reflects changes in the abundance or transcriptional state of neuronal populations within the RV tissue. We have removed this point in the revised manuscript. Regarding SLITRK5 expression in AV node cells, we agree that this reference was tangential and have removed it from the revised text.

5. Do the transcriptomic changes in genome-encoded mitochondrial components observed between NF, pRV, and RVF samples reflect alterations solely in mitochondrial function, or do they also impact mitochondrial abundance? Could the authors suggest experimental approaches to investigate this? This question is also relevant to the pediatric data, where 'mitochondrial expansion' is mentioned (line 386).

We thank the reviewer for this thoughtful question. The transcriptomic alterations we observe likely reflect a combination of changes in mitochondrial function and abundance, which cannot be fully distinguished by RNA-based approaches alone. In adult RVF, the coordinated decline in nuclear-encoded ETC transcripts is paralleled by reduced respirometric capacity (**Figure 5H**), consistent with functional impairment; whether this also reflects reduced mitochondrial mass per cardiomyocyte is not directly addressed by our current dataset. Notably, the prior point about pediatric mitochondrial expansion was an artifact of normalization. After proper normalization we no longer observe any change in pediatric mitochondrial transcripts. Furthermore, we see no evidence of impaired respirometric capacity in pediatric failing palliated HLHS hearts.

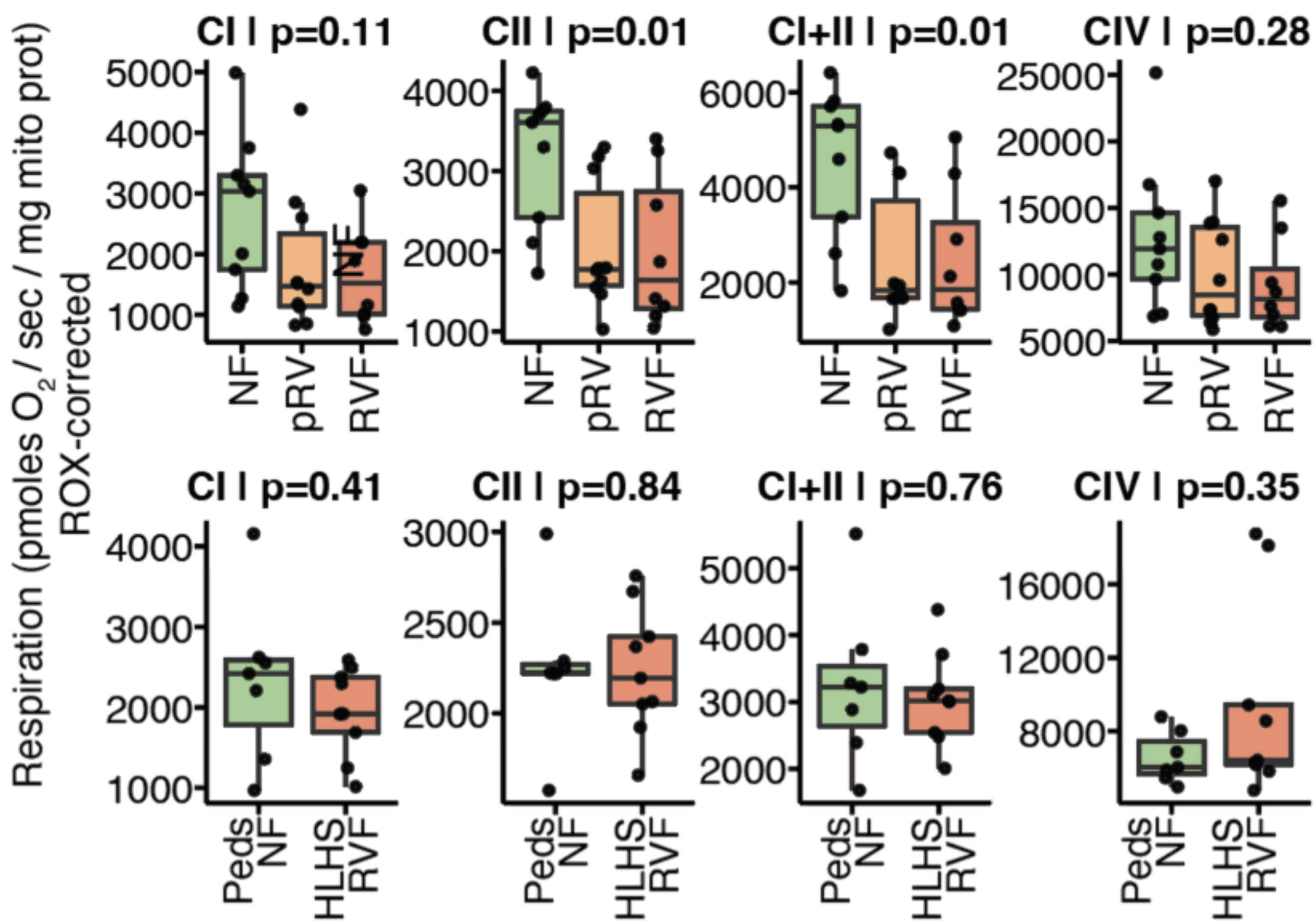

Figure 5H: Adult and pediatric respirometry. Oroboros 2k high-resolution respirometry on freeze/thaw-isolated RV mitochondria, ROX-corrected and normalized to starting mitochondrial protein mass (pmoles O_2 /sec/mg mitochondrial protein). Each dot is one patient. Adult cohort **(top)**: NF n=9, pRV n=10, RVF n=8; Kruskal–Wallis p-values shown. Pediatric cohort **(bottom)**: pediatric NF (n=7) vs HLHS-palliated failing (n=9); Mann–Whitney pairwise p-values.

Investigating the question of mitochondrial abundance will require complementary measurements that are beyond the scope of the current study. Specifically, we propose: (i) mitochondrial DNA copy-number quantification by qPCR as a measure of organelle abundance; (ii) citrate synthase activity assays as an enzymatic surrogate of mitochondrial mass; (iii) electron microscopy for direct quantification of mitochondrial

morphology and number per cardiomyocyte; and (iv) mitochondrial protein quantification (e.g., OXPHOS complex subunits by Western blot or mass spectrometry).

6. Regarding the Xenium analysis, were the tissue samples sectioned in a consistent orientation (e.g., similar sectioning planes of elongated cardiomyocytes)? If not, how reliable is the comparison between these datasets and different conditions? In Fig. 3C, there appears to be a striking difference in the density of annotated niches between the NF and RVF samples - could this disparity be attributed to biological variation or technical factors? Additionally, the niche nomenclature appears inconsistent; in some cases, it is based on the dominant cell type (which can be misleading as it might be mistaken for spatial single-cell clusters), while in others it refers to macroscopic tissue structures. This should be standardized or further clarified. It could also be helpful if the authors included a dot plot or a similar visualization showing the relative enrichment of different major cell types within the identified niches.

We thank the reviewer for these important questions regarding the spatial analysis.

First, regarding tissue orientation: all Xenium samples were sectioned transmurally across the right ventricular free wall, ensuring comparable tissue orientation across samples. We have clarified this in the revised Results section.

Second, regarding differences in niche density between NF and RVF samples: these differences reflect both biological variation (changes in cellular composition and spatial organization during disease progression) and technical factors (differences in tissue section area and cell density). We now discuss this in the revised manuscript.

Third, regarding niche nomenclature: in the revised manuscript, niches are framed explicitly as spatial microenvironments resolved by BuildNicheAssay (k=100 nearest spatial neighbors, CLR-transformed proportions), distinct from cell-type or cell-state clusters; the Figure 3 legend and Methods describe niches as the 14 microenvironments emerging from this procedure. We have adopted descriptive labels that abbreviate the dominant cellular composition or anatomical context of each niche, as detailed in the Figure 3F legend: adipo (adipocyte-rich), adipo-vasc (adipo-vascular), arterial, cap myo (capillary myocardium), comp myo (compact myocardium), fibro myo (fibrotic myocardium), remod myo (remodeling myocardium), remod stroma (remodeling stroma), infl adipo (inflammatory adipocyte), infl adipo-vasc (inflammatory adipo-vascular), myo (myocardium), peri-arteriole, stressed myo (stressed myocardium), and sub-epi (sub-epicardial). To enable readers to inspect the cellular composition of each niche directly, Figure 3F now shows a stacked bar chart of niche composition by lineage (fraction-of-niche), summarizing the lineage identity of each microenvironment.

7. What filtering steps were included for the primary Xenium data and the segmented cell before cell label assignment? How were the spatial niches defined? Have the authors considered performing spatial gene imputation from the corresponding single-nucleus RNA-seq data for more in-depth, spatially informed analysis (e.g. ligand-receptor interactions)?

We thank the reviewer for these important questions.

Regarding filtering: we have expanded the Methods section to describe the filtering steps applied to the Xenium data prior to cell-type assignment. In the revised analysis, data were reprocessed using Proseg for cell segmentation, which assigns transcripts to cells based on transcript density and cell morphology. Following segmentation, SPLIT-based cellular de-mixing was applied to correct for diffusion-associated ambient RNA contamination, and cells with low transcript counts were filtered. Cell-type labels were then assigned using RCTD with the snRNA-seq dataset as a reference.

Regarding spatial niches: niches were defined using the Seurat BuildNicheAssay function, which identifies spatial neighborhoods based on the local composition of cell types surrounding each cell. We have expanded the description of this methodology in the revised manuscript.

Regarding spatial gene imputation and ligand–receptor analysis: in response to the reviewer's suggestion, we performed gene expression imputation using SpaGE, which leverages shared principal vectors between the Xenium and snRNA-seq datasets to infer the expression of genes not present in the original Xenium panel. Using the combined panel and imputed gene expression, we then performed spatially-informed ligand–receptor interaction analysis using CellChat. This analysis revealed a gain of extracellular-matrix (collagen, laminin, thrombospondin) signaling on top of a stable cell-contact adhesion baseline. These results are now presented in the revised manuscript.

8. In Fig. 3B, please include the endocardial cluster as well. More generally, adding anatomical landmarks, such as the endocardium, and possibly a scale bar to the Xenium images would greatly aid their interpretation.

We thank the reviewer for this suggestion. We have updated the figure to include all 12 cell types. We have also annotated the endocardium, as well as included scale bars to improve interpretability of the spatial images.

9. TMEM65, referenced in line 168, has also been reported to localize to the mitochondrial inner membrane (Nishimura et al., PeerJ, 2014). This subcellular localization is likely more pertinent to the mitochondrial patterns discussed in this paragraph than its alternative localization to the intercalated disc, mentioned by the authors.

We thank the reviewer for this helpful suggestion. We notably removed discussion of *TMEM65* in the latest version of the manuscript.

10. The authors describe a pseudo-steroid deficient state in RVF monocytes, among other cell types. Do they suggest that these blood cells acquire this state after infiltrating the failing heart tissue, or could this reflect a systemic immunomodulatory effect associated with RVF?

We thank the reviewer for this thoughtful question. The observation that glucocorticoid-responsive gene expression (particularly FKBP5) is reduced across virtually all cell types in the RV, including resident populations (cardiomyocytes, fibroblasts, endothelial cells, pericytes, resident macrophages) and infiltrating populations (monocytes, T cells), raises the question of whether this reflects a local tissue-level phenomenon or a systemic immunomodulatory effect. We cannot definitively distinguish between these possibilities with the current dataset, as we did not profile circulating immune cells. We have added this discussion to the revised manuscript and note that profiling circulating monocytes in matched patients would be an informative future study.

11. Was the observed fibroblast-to-myofibroblast transition accompanied by fibrosis at the microscopic level in the pRV and RVF samples? Has this been previously documented in the literature, or could it be further explored in this study using specific histological stains, such as Masson's trichrome?

We thank the reviewer for this excellent suggestion. In response, we performed collagen-specific histology on both human RV samples (Masson's trichrome and Sirius Red on NF, pRV, RVF) and murine PAB RV tissue (Sirius Red, at 2-week time point). These analyses confirmed progressive fibrosis from NF→pRV→RVF in human RV (**Supplementary Figure S3M;** +5.9% collagen area per stage, significant linear trend) and progressive fibrosis across PAB stages in mouse RV (**Supplementary Figure S6I**), consistent with the transcriptional evidence of fibroblast activation (ACTA2 induction) and myofibroblast transition observed in both the snRNA-seq and Xenium datasets. These histological data are now presented in the revised manuscript and provide important orthogonal validation of the transcriptomic findings.

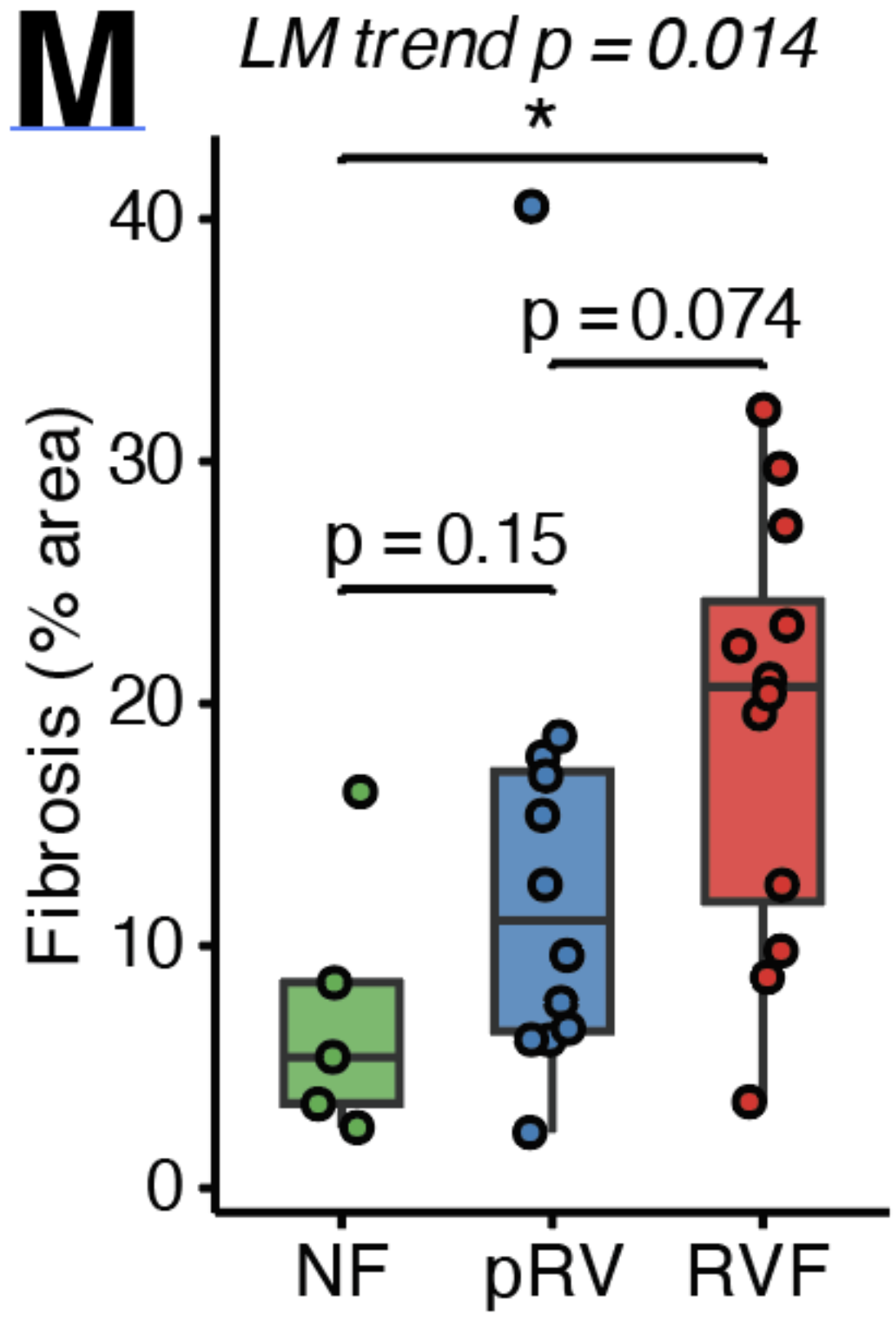

Figure S3M: Per-patient fibrosis quantification. Adult RV trichrome / Sirius red fibrosis (% area) by disease state (n = 5 NF, 12 pRV, 12 RVF) boxplot with pairwise Wilcoxon brackets. Significant linear model with ordered group as continuous predictor is shown.

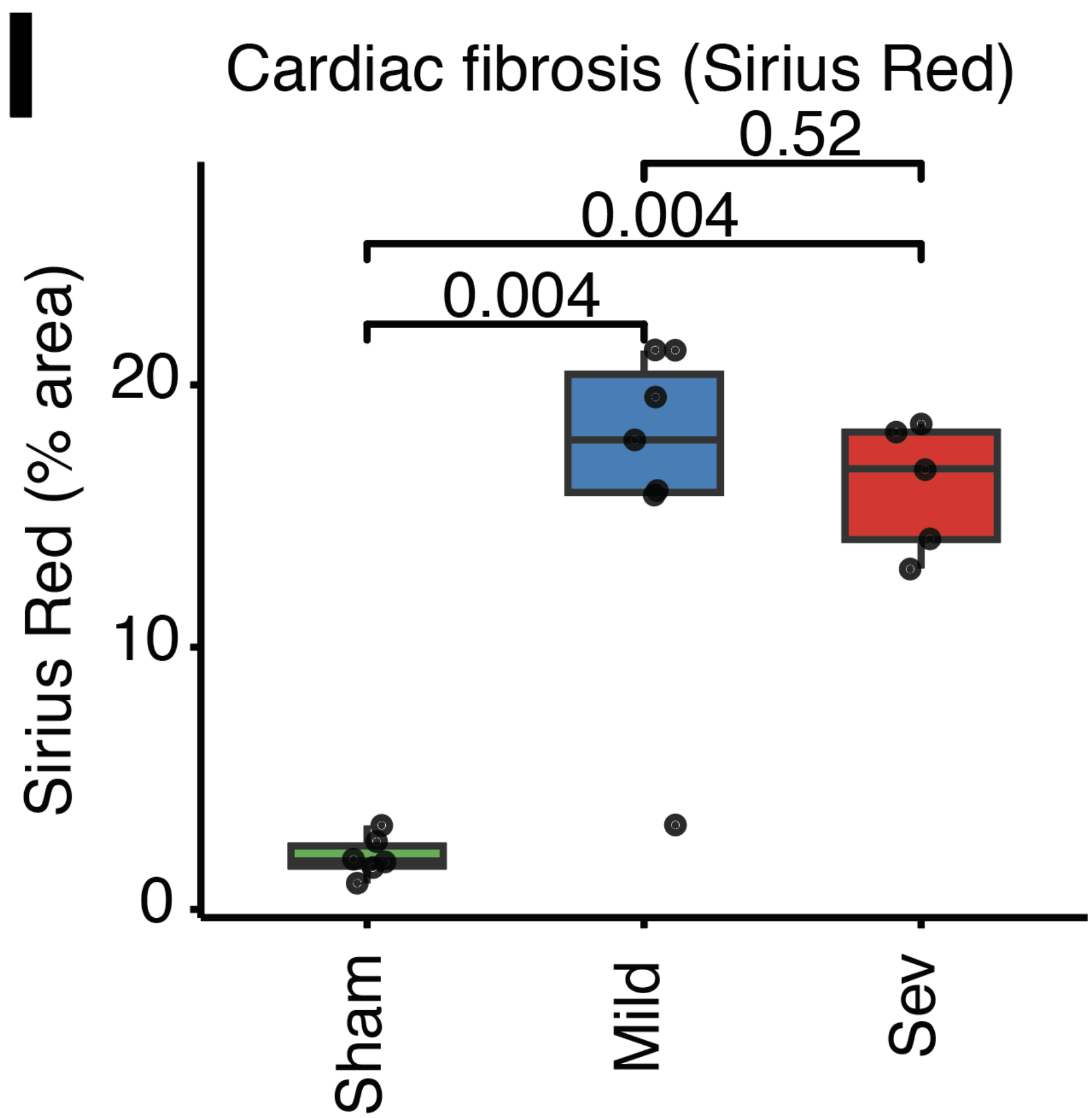

Figure S6I: PAB Sirius Red fibrosis. Cardiac fibrosis quantified as Sirius Red percent area per animal across PAB stages (Sham n = 6, Mild n = 7, Sev n = 5; Wilcoxon).

12. I could not find any information on the number of nuclei analyzed in the mouse and the pediatric single-nucleus datasets. Also, how many mice were used for the single-nucleus RNA-seq experiments?

We thank the reviewer for pointing this out. The revised manuscript and figure captions now explicitly report cohort sizes and total nuclei: the murine PAB snRNA-seq cohort comprised n=3 sham, n=3 PAB-mild RVF, and n=4 PAB-severe RVF mice (55,420 nuclei total; **Figure S5A; Table S7**). The pediatric snRNA-seq cohort comprised n=4 pediatric controls and n=10 HLHS-palliated patients (n=5 Fontan without RVF, n=4 Fontan with RVF, n=1 Glenn with RVF), totaling 209,604 nuclei (**Figure 8A**). These details are now included in the Results section and Supplementary Tables.

13. Could the authors clarify what is meant in line 310 by describing the mild RVF group in the mouse model as 'protected' based on the single-cell data? In lines 484–490 of the discussion, they note important differences between the disease progression in the mouse RVF model and human pathology. From this, one might infer that the mild RVF group exhibits more adaptive tissue responses to the PAB insult compared to the severe

group. It would be helpful if the authors could elaborate on this point in the main text, specifically in relation to the observations from the different mouse experimental groups.

We agree with the reviewer that the term "protected" was speculative and potentially misleading. In the revised manuscript, we have replaced this language with more neutral descriptions, now characterizing the mild-RVF group as a distinct transcriptional state rather than a linear intermediate. We have also expanded the Results to clarify that these observations may reflect differences in the severity or chronicity of pressure overload rather than a discrete protective mechanism, which better contextualize the mouse experimental groups in relation to the human disease spectrum.

14. In lines 349–350, the authors report induction of several cardiomyocyte-specific transcripts in myeloid cells in both pediatric and adult RVF samples. This finding is more likely attributable to contamination, such as ambient RNA (despite the authors' efforts to address ambient RNA cleanup as described in the methods) rather than reflecting a true biological phenomenon. Please review this observation and consider revising the statement accordingly.

We thank the reviewer for raising this concern. We agree that ambient RNA contamination is the most parsimonious explanation for the detection of cardiomyocyte-specific transcripts in myeloid cells, particularly given the technical challenges of nuclear isolation. Interestingly, however, we have previously addressed this question specifically in the context of cardiac endothelial cells, where we also saw cardiomyocyte-specific transcripts (Yucel et. al., 2020, PMID: 33315013). In that previously published study, to differentiate between RNA contamination and endothelial-inherent gene expression, we evaluated the chromatin state with ATAC-Seq. Surprisingly, we found that the chromatin of cardiomyocyte-specific genes (e.g. sarcomeric genes) was open in cardiac endothelial cells. Thus we concluded that cardiac endothelial cells maintain open chromatin and expression of cardiomyocyte myofibrillar genes. The same may be true in myeloid cells, but our single-nucleus data cannot distinguish this from ambient contamination. In the reorganized Results section of the revised manuscript, we no longer feature this observation as a substantive biological finding. The Methods section (under "Single-nucleus RNA sequencing: ambient RNA and noise removal") now explicitly acknowledges that residual cytoplasmic RNA contamination during nuclear isolation can produce low-level off-target detection of canonical cell-type markers (e.g., *RYR2* in non-cardiomyocyte populations), and cell-type assignment relies on relative enrichment over a panel of cluster-defining markers rather than on individual cross-cell-type signals. We thank the reviewer for prompting this clarification.

15. In Fig. 5A and 5C, the enrichment pattern of module M2 appears to be opposite when comparing DCM and donor (control) samples. Could the authors clarify the difference between these two plots? It would be helpful to revise the figure legend to make this distinction more explicit.

We thank the reviewer for pointing out this apparent discrepancy. Previous Fig. 5A (now 8G) showed module eigengene scores computed on the full dataset, while Fig. 5C (now

8I) showed module scores computed after subsetting to specific cell types. The apparent reversal in M2 enrichment reflects the difference in cellular composition between the two analyses, when scored across all cell types, the module signal is diluted by non-cardiomyocyte populations, whereas the cardiomyocyte-specific analysis captures the cell-type–specific pattern.

16. In the description of the clustering analysis, please include the specific parameters used (e.g., resolution, number of principal components, nearest neighbors).

We thank the reviewer for this suggestion. We have expanded the Methods section to explicitly report clustering parameters, including the number of principal components used for dimensionality reduction, the number of nearest neighbors for graph construction, and the clustering resolution for each analysis.

17. Please provide more detailed technical information on how the trajectory analysis was performed.

We thank the reviewer for this suggestion. We have removed the trajectory analysis from the revised manuscript.

Minor comments:

18. Please provide a brief description of the Supplementary Data files for easier navigation.

We thank the reviewer for this suggestion. We have added a Supplementary Data index table that describes the contents of each supplementary data file to facilitate navigation.

19. In the abstract (lines 35-36), the authors state that the expansion of the endothelial cell compartment is unique to RVF compared to LVF, but I could not find any specific comment on this discrepancy within the main text. It would be helpful if the authors could clarify the basis for this claim and, if possible, offer any hypotheses to explain this observed discrepancy.

We thank the reviewer and agree that the original abstract overstated this point. The abstract has been revised to describe the endothelial-compartment expansion in pRV/RVF without asserting uniqueness relative to LVF. Indeed, endothelial expansion has been reported in ischemic LV cardiomyopathy (Simonson et. al., *Cell Reports 2023*; PMID: 36790929), and we have no head-to-head RV-versus-LV comparison that would establish RV-specificity. We now develop the cross-chamber comparison in the Discussion and frame the expansion as a hypothesis-generating observation.

In the revised Discussion we frame the EC compartment expansion observed across pRV and RVF as unusual, but not unique, among published heart-failure transcriptomic studies, and explicitly position it as a hypothesis-generating observation rather than a definitive RV-versus-LV claim. Specifically, we hypothesize that EC expansion reflects

coronary microvascular adaptation to RV-specific hemodynamics, a thin-walled chamber perfused throughout systole as well as diastole, driving compensatory capillary/arterial expansion to maintain wall oxygenation. We further note that this expansion has distinct subtype composition across cohorts (adult MECOM-positive arterial induction and pediatric venous-dominated expansion), reflecting different developmental contexts, and that the Phase-2 angiopoietin-axis destabilization signature (ANGPT2, MYC, ADAMTS1) suggests this initially compensatory vascular response transitions toward destabilization at decompensation. Dedicated experimental designs will be needed to test these mechanistic interpretations.

20. I recommend switching panels Fig. 1F–G with Fig. S1E–F between the main and supplementary figures. The latter, which illustrates gradual changes along the course of disease progression, appears more central to the study's main message. In contrast, the former, depicting a peculiar pattern for a gene module related to antigen presentation, is not thoroughly explained or contextualized and thus seems less critical.

We thank the reviewer for this suggestion. We considered this reorganization carefully. In the revised manuscript, **Figure 1E** now shows WGCNA module-score violins across NF/pRV/RVF for key modules (capturing disease-state progression including the M4 biphasic pattern), and the PAH cross-cohort comparison (**Figure 1F; S1I**) was added as new panels in response to multiple reviewers' requests for cross-etiology context. The bulk DEG heatmaps illustrating gradual increasing and decreasing genes have been retained in **Figure S1C–D** where they provide direct gene-level support to the module-level summary in the main figure. We hope this organization satisfies the spirit of the suggestion while preserving the cross-etiology PAH comparison as a high-value main-figure panel.

21. There appears to be no legend provided for Fig. S3C. Please add a corresponding legend to facilitate interpretation of the panel.

We thank the reviewer for catching this. A legend has been added in the revised manuscript.

*22. Please add a statement to the ethics part of the methods on how the sensitive, poly**(A)**-capture-based sequencing data is handled.*

We thank the reviewer for this important point. We have added a statement to the Ethics section describing how sequencing data are handled, including data storage, access controls, and compliance with relevant data privacy regulations.

23. In line 991, I assume the authors meant RV (and not LV) samples.

We thank the reviewer for catching this typographical error. This has been corrected to "RV" in the revised manuscript.

Comments on the code:

24. The authors did not share information about the package versions for reproducibility. Please consider adding at least the output of sessionInfo(). For such an atlas paper, which most likely will be reused by other scientists, adding environment infos such as a renv.lock file with renv package or a Docker container would be very useful for reuse of the data and further exploration of the dataset.

We thank the reviewer for highlighting the importance of reproducibility for this study.

In response, we have updated the analysis repository to provide more complete information on the computational environment used for all analyses. Specifically, we have added detailed documentation describing software dependencies and included explicit instructions for generating a Conda environment that reproduces the analysis environment used in this study. The repository README now provides step-by-step guidance for setting up this environment and running the analysis workflows. The Conda specification in the repository pins R version, BioConductor channel, and the set of R packages required to reproduce the analyses.

The updated codebase is available at:https://github.com/ikuznet1/RV_Atlas

We agree that containerized environments (e.g., Docker) or renv-based workflows may further facilitate reproducibility and will consider these approaches for future work.

25. It seems like the current GitHub is not presenting the script for processing the snRNA-seq data. Please consider adding the processing scripts with loading the raw files, or 10x cellranger outputs (or similar) as well as filtering, doublets handling, normalisation and batch correction.

We thank the reviewer for this important suggestion regarding reproducibility.

In response, we have expanded the GitHub repository to include scripts for processing the single-nucleus RNA-seq data from raw inputs through downstream analysis. Specifically, we have added scripts covering key preprocessing steps, including:

- loading raw data (e.g., CellRanger outputs),
- quality control and filtering,
- ambient RNA correction,
- doublet detection and removal,
- normalization and scaling (SCTransform),
- dimensionality reduction and clustering,
- and batch correction/integration (Harmony).

These additions provide a more complete and transparent description of the snRNA-seq analysis pipeline and enable end-to-end reproducibility of the dataset processing.

The updated codebase is available at:https://github.com/ikuznet1/RV_Atlas

26. Some files are read using readRDS() such as scWGCNA_bulk2sn_projection.rds or bulk_heart_modules.csv. Please consider adding clearly where these files/objects come from and if generated by you, add the script for it (unless I missed it).

We thank the reviewer for highlighting this point regarding reproducibility and clarity of intermediate data objects.

In response, we have updated both the code repository and data availability to clearly document the origin of these files. Specifically, the referenced .rds and .csv files represent intermediate objects generated as part of the analysis pipeline (e.g., WGCNA module assignments and projections between bulk and single-nucleus datasets).

We have now:

- added scripts to the repository that explicitly generate these intermediate objects from the primary data, and
- made these files directly available via Zenodo, ensuring that users can either reproduce them from raw data or access them directly for convenience.

Additionally, we updated the README to clearly indicate where these files are generated within the pipeline and how they are used in downstream analyses.

The updated codebase is available at:https://github.com/ikuznet1/RV_Atlas

- Associated data objects are available via Zenodo: [10.5281/zenodo.20115563](https://doi.org/10.5281/zenodo.20115563)

27. There are several mismatches regarding the numbering of figure panels between the scripts and the manuscript. Please consider updating the numbering in the scripts for future readers to avoid confusion.

We thank the reviewer for pointing this out. We have corrected the figure panel numbering in the scripts to ensure consistency with the revised manuscript and to improve clarity for future users of the codebase.

28. Where some extra code is added, please make sure to add a title to specify that these parts of scripts are not used to generate figures.

We thank the reviewer for pointing this out. We have commented the pertinent parts of the code.

Reviewer #3 (Remarks on code availability):

Please see my comments on the code above. The authors did not provide a proper README file, and in lack of the study data it was not possible to assess if it is possible to recreate the plots included in the manuscript. The evaluation is therefore based on reading the scripts, which overall

We thank the reviewer for pointing this out. We have added a README file. The data needed to replicate the figures is uploaded to Zenodo: [10.5281/zenodo.20115563](https://doi.org/10.5281/zenodo.20115563).

Reviewer #4 (Remarks to the Author):

In this manuscript by Kuznetsov et al. the authors use a variety of transcriptomic techniques to probe the landscape of adult and pediatric human and mouse right ventricles that are healthy, pressure-loaded or failing. The authors do this to compare and contrast differences with age, species and disease of the right ventricle, which is grossly understudied compared to the left ventricle. It is an interesting cohort that is generally well-probed and comprehensive. As the authors point out this study is descriptive, but has the potential to fuel more mechanistic and hypothesis-driven studies.

This reviewer is not an expert in assessing the quality or validity of the extensive RNA-seq analyses, but can understand their interpretation, which generally seems founded. Other reviewers will undoubtedly provide more stringent review and assessment of the structural cell biology. However, this reviewer does have experience in cardiovascular immunology, preclinical models and macrophage biology. Although many observations are logical and intriguing, some are perplexing and require greater insight before publication can be considered in Nature Cardiovascular Research.

1. Greater methodological details of the pulmonary arterial banding model need to be provided. Although it is a common model, there is variation in the severity of its induction, which vary from lab to lab or experiment to experiment. Moreover, the authors should provide more functional and immunological data of this model, particularly due to the fact that much of the model seemingly does not recapitulate human disease. Although the model is well-described in the literature, it needs to be shown here so conclusions or hypotheses as to why their is discordance between mice and humans can be made. There is some description of this in the discussion, but more data are needed. At 2 weeks, what is the right ventricle ejection fraction of these mice, as well as other metrics such as diameters and volumes and blood pressure? By flow cytometry the inflammatory landscape of the heart should be compared to sham controls with particular emphasis on the macrophage subpopulations using CCR2, TREM2, MHC-II etc. Additionally, histology for cardiomyocyte hypertrophy and fibrosis could be used to compare the pathology of disease in mice and humans.

We thank the reviewer for this thoughtful and important comment regarding characterization of the pulmonary artery banding (PAB) model.

First, we have expanded the Methods section to provide additional detail on the PAB procedure, including experimental conditions and timing, to improve clarity and reproducibility. We agree that PAB severity can vary between laboratories, and the revised Methods now specify strain (C57BL/6J), age, anesthesia, surgical approach, banding technique, sham approach, perioperative care, and the two harvest time points (2-week and 2-month, with RV remodeling confirmed by echocardiography. Per-mouse echocardiographic outcomes in **Table S8**). The 2-week and 2-month cohorts are treated as independent and not pooled in downstream statistical analyses.

Second, in response to the request for additional functional characterization, we have expanded the description of the cardiac phenotype of the PAB model, including additional

echocardiographic and morphometric parameters describing RV remodeling and dysfunction.

Third, we have incorporated additional analyses of mitochondrial function in the PAB model, including both early (2-week) and extended (2-month) time points (see Response to Editorial Comment 4; **Figure 5I**). These data demonstrate persistent mitochondrial dysfunction across disease stages and strengthen comparisons to human RVF.

Fourth, we performed flow cytometric analysis of cardiac immune populations in PAB and sham mice to more directly characterize the inflammatory landscape. These analyses included assessment of macrophage subpopulations using markers such as CCR2, TREM2, and MHC-II, and provide orthogonal validation of the immune changes suggested by the single-nucleus RNA-seq data. The results of these analyses have now been incorporated into the revised manuscript (**Figure S5H**).

Fifth, we performed Sirius Red histology on RV tissue from PAB and sham mice to quantify fibrotic remodeling, a key pathological feature of pressure-overload RVF. Quantification confirmed progressive collagen deposition across PAB stages (Sham, Mild, Sev; **Figure S6I**), complementing the transcriptomic and respirometry analyses and supporting the relevance of the PAB model. We have also added direct cardiomyocyte hypertrophy histology for the human cohort: WGA-stained FFPE human RV sections (NF n=4, pRV n=4, RVF n=4; 17,864 CMs segmented via CellPose) show progressive cardiomyocyte hypertrophy across NF→pRV→RVF (per-patient median min Feret diameter: NF 55.8 µm; pRV 73.9 µm [+32%, BH $p = 0.011$]; RVF 102.5 µm [+84%, $p < 10^{-5}$]; pRV vs RVF $p = 0.026$; cell-level linear mixed model with random intercept by patient; new **Figure S2I**; **Data S16**). WGA staining was not performed on the murine PAB cohort; PAB structural-remodeling claims continue to rely on fibrosis quantification (**Figure S6I**) and the established hypertrophic phenotype of the PAB model.

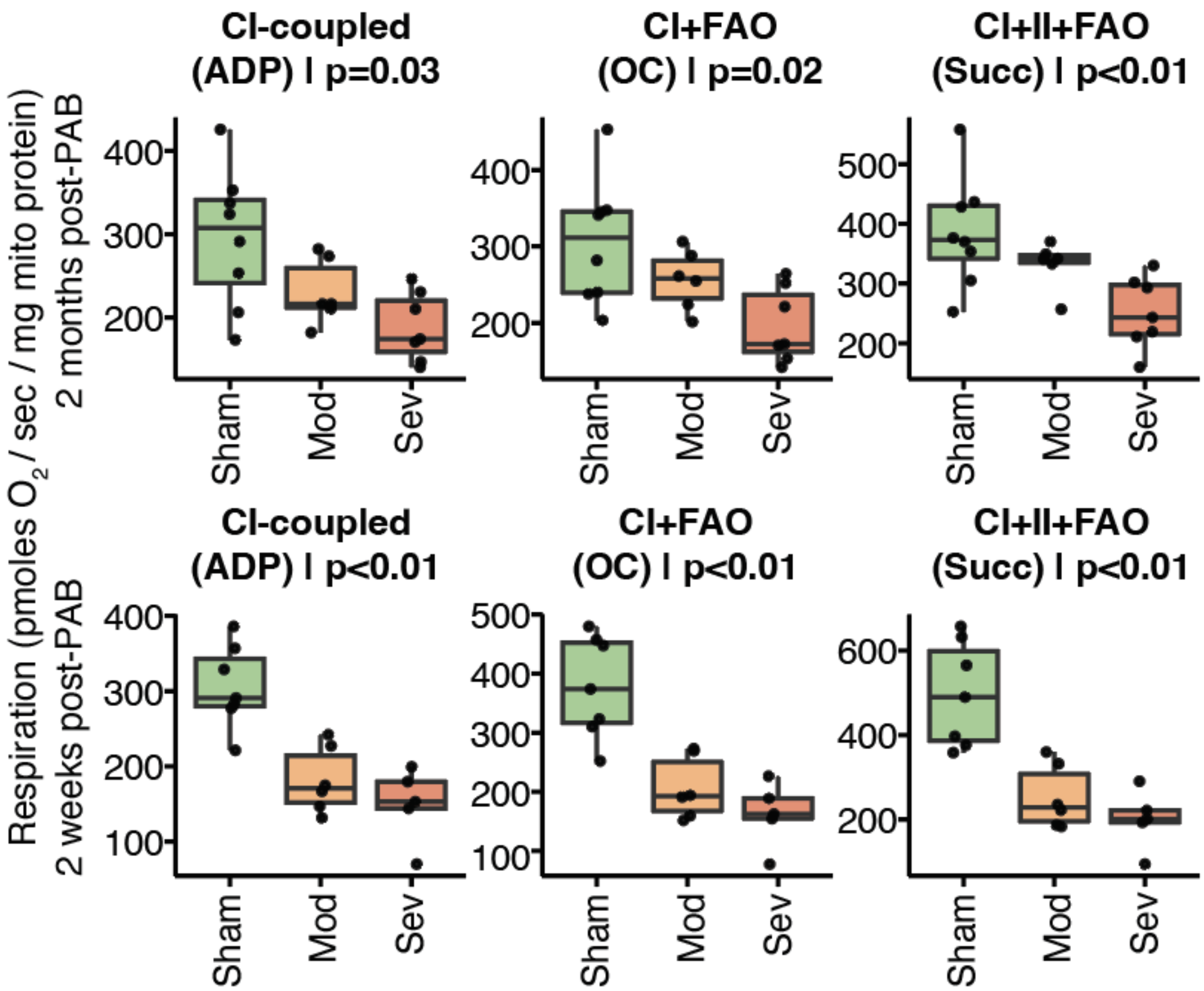

Figure 5I: PAB respirometry. Respirometry in PAB model at 2-month **(top)** and 2-week **(bottom)** time points vs sham, using a substrate-addition protocol (PM → ADP → cyt c → octanoyl-carnitine → succinate) on tissue homogenate. CI+II+FAO maximal coupled respiration (Succ-driven) is the headline measure. 2-week: Sham n=8, Mild n=6, Severe n=7. 2-month: Sham n=7, Mild n=6, Severe n=5. Each dot is one animal; Kruskal–Wallis p-values shown.

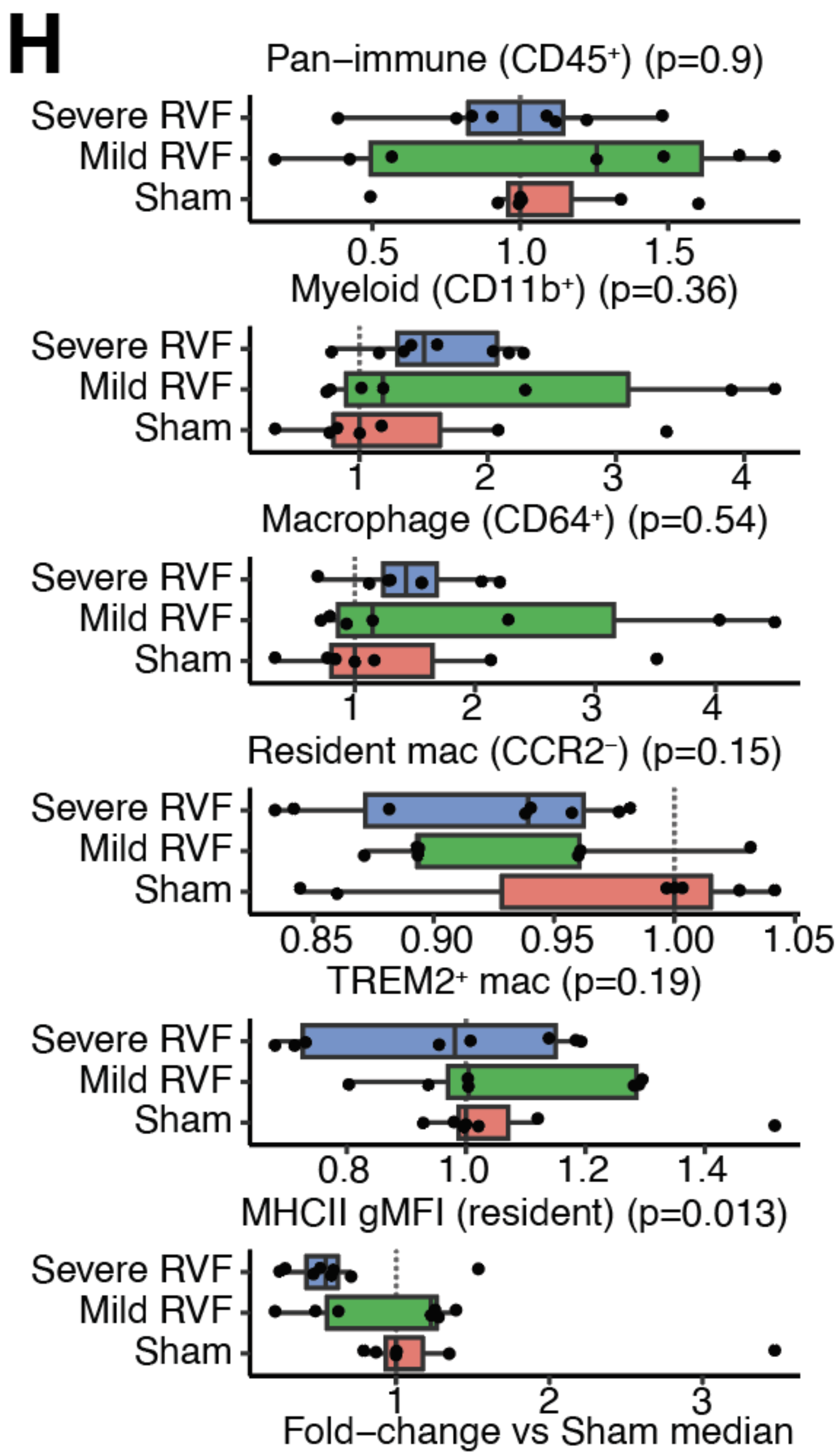

Figure S5H: Flow-cytometry validation of cardiac immune populations in the murine PAB model (Sham/Mild RVF/Severe RVF). Per-mouse fold-change relative to the within experiment Sham median for leukocytes ($CD45^+$), myeloid cells ($CD11b^+Ly\text{-}6G^-$), macrophages ($CD64^+$), $CCR2^-$ resident macrophages, $TREM2^+$ macrophages, and MHC-II geometric mean fluorescence intensity (gMFI) on $CCR2^-$ residents; n = 7/7/8 mice. Log-scale linear-trend p across severity is shown. Antibody clones and catalogue numbers are listed in Methods.

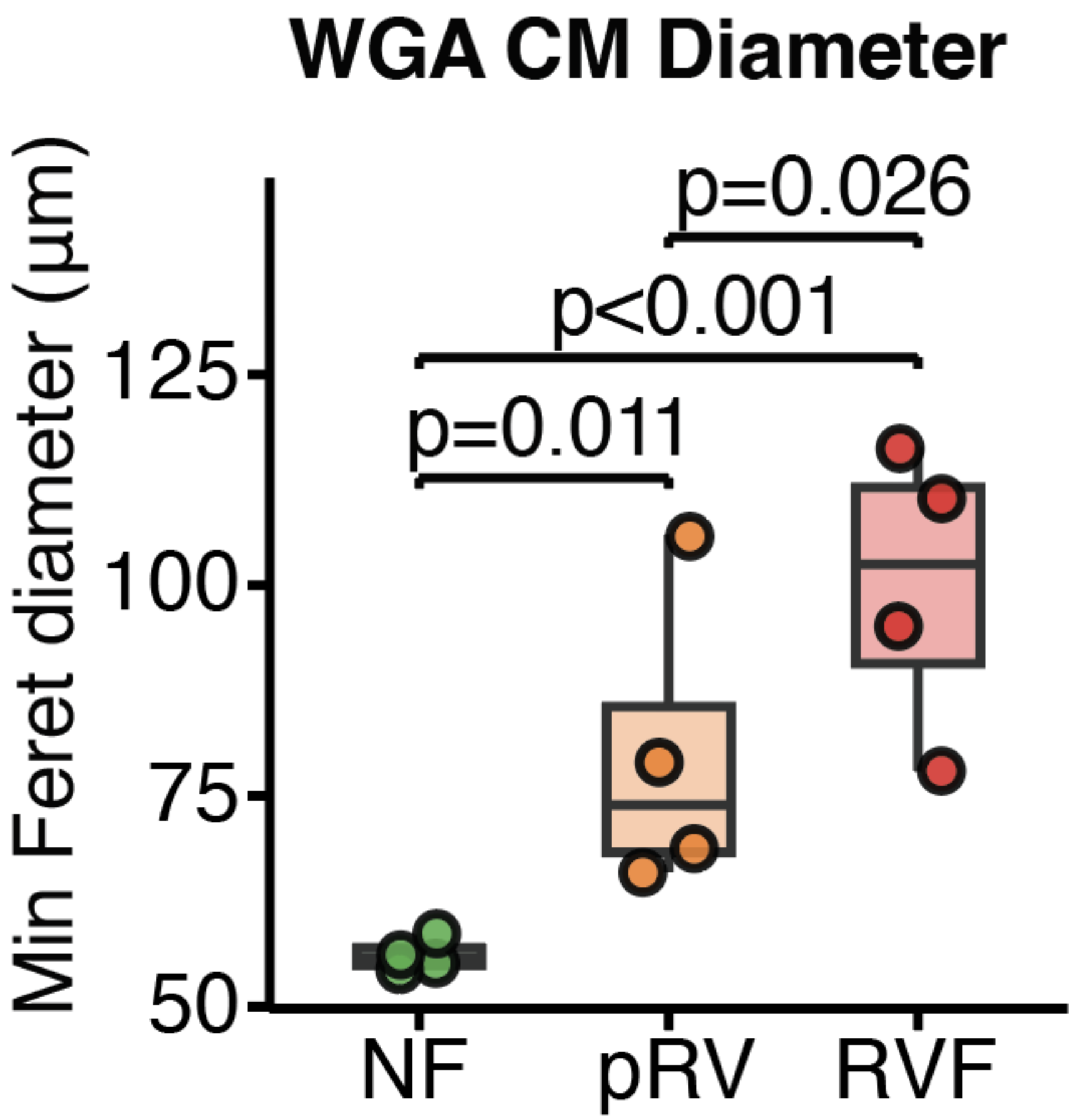

Figure S2I: WGA cardiomyocyte diameter. Cardiomyocyte minimum Feret diameter (proxy for CM cross-sectional area) measured by wheat-germ agglutinin (WGA) staining of human RV sections (NF n=4, pRV n=4, RVF n=4; 17,864 CMs total). Boxplot of per-patient median minimum Feret; each dot is one patient.

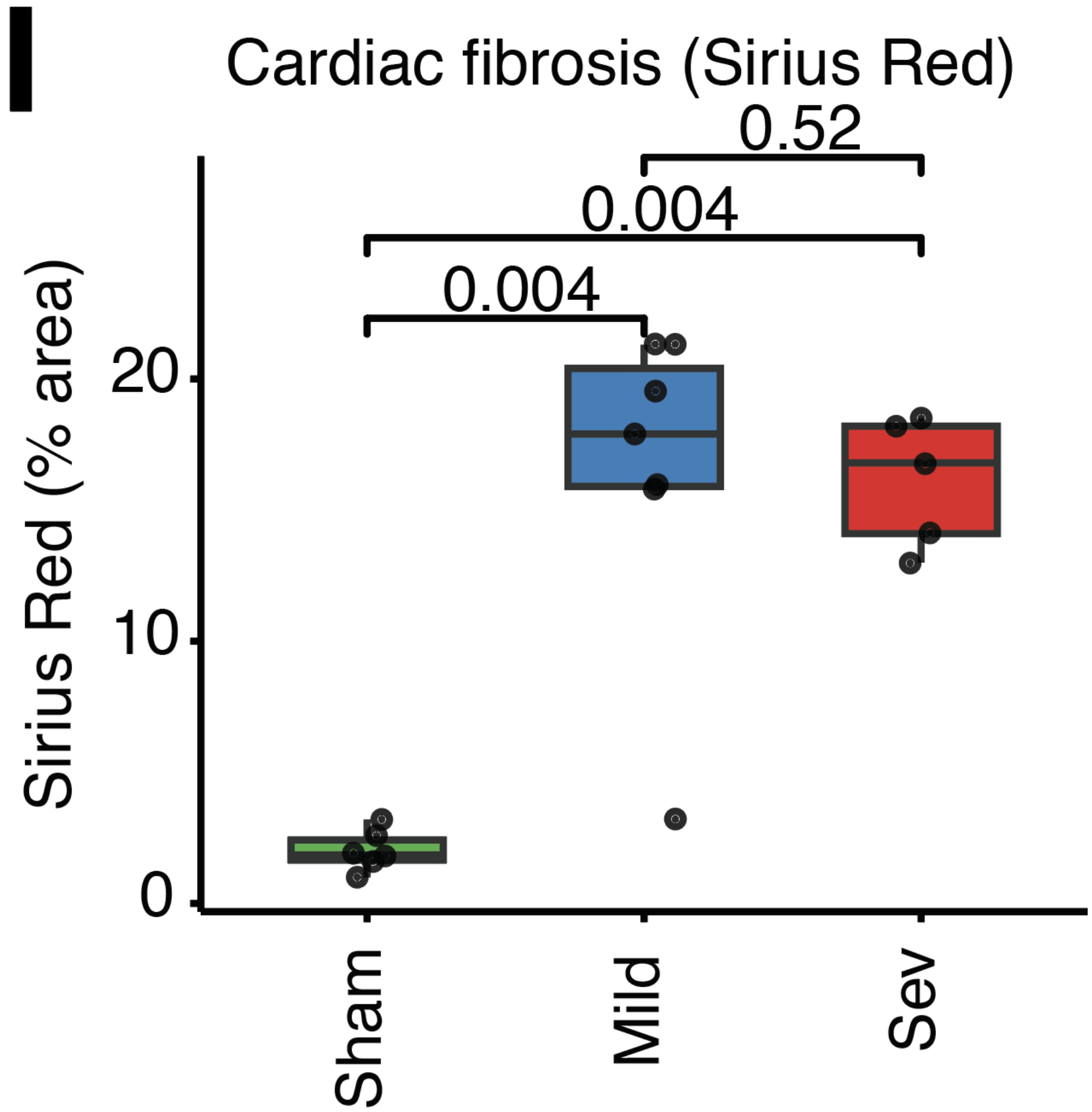

Figure S6I: PAB Sirius Red fibrosis. Cardiac fibrosis quantified as Sirius Red percent area per animal across PAB stages (Sham n = 6, Mild n = 7, Sev n = 5; Wilcoxon).

Together, these additions provide a more comprehensive functional and immunological characterization of the PAB model.

Finally, we have expanded the Discussion to more clearly address the discordance between murine PAB and human RVF, emphasizing that differences in disease chronicity, hemodynamic load, and species-specific biology likely contribute to the observed transcriptional differences.

We note that while there is variation in the severity of PAB induction across laboratories, this model is well-established and widely used to study RV pressure overload. In the revised manuscript, we provide additional functional characterization including echocardiographic parameters (TAPSE, fractional area change, and RV end-diastolic area; **Table S8**) at both 2-week and 2-month time points, demonstrating progressive RV remodeling and dysfunction. These data are now clearly presented alongside the transcriptomic analyses to allow readers to assess the degree of RV dysfunction in our specific implementation of the model.

Why is expression of CCR2 low in this dataset, as the others point out in line 201? It is frequently used in both mouse and human studies at the transcript and protein level for segregating macrophage and monocyte populations. How does it compare to other published datasets?

We thank the reviewer for raising this important point.

We note that detection of certain transcripts, particularly those expressed at relatively low levels, can be limited in droplet-based single-nucleus RNA-seq platforms such as 10x Genomics due to well-described dropout effects. This phenomenon disproportionately affects genes with modest expression levels.

Importantly, low or variable detection of CCR2 transcripts in cardiac macrophages has also been observed in prior single-cell transcriptomic studies of the human heart (e.g., Koenig et al., 2022), suggesting that this is not unique to our dataset. CCR2 expression in cardiac macrophages is often modest at the transcript level and may therefore be particularly susceptible to dropout in single-nucleus RNA-seq data.

To address this, we relied on combinatorial marker expression and transcriptional signatures, rather than CCR2 expression alone, to define macrophage subpopulations. This approach is consistent with prior studies of cardiac macrophage heterogeneity.

It is unclear why the authors used CX3CR1-cre Rosa26-TdTomato mice in these experiments. In theory, all monocyte/macrophage lineage cells will be red following tamoxifen injection and thus it provides no more insight than the usage of a sorting CX3CR1+ cells for bulk seq. Caution should be used therefore in referring to the bulk seq as resident macrophages, particularly because no flow or sorting data are provided. It is also not clear how this is intended to "orthogonally validate" the discordance.

We thank the reviewer for this important comment and appreciate the opportunity to clarify the rationale for this experimental approach.

The *CX3CR1*-CreER; Rosa26-tdTomato system was used in a pulse–chase fate mapping strategy to distinguish long-lived resident macrophages from short-lived circulating monocyte-derived populations. Specifically, mice were administered tamoxifen at 3 weeks of age for one week to label CX3CR1-expressing cells, followed by a 4-week washout period prior to pulmonary artery banding (PAB). During this interval, short-lived circulating monocytes are replaced and lose tdTomato labeling, whereas long-lived tissue-resident macrophages retain the label. This approach has been previously validated, demonstrating near-complete turnover of circulating monocytes during the washout period (e.g., Dick et. al., *Nature Immunol.* 2019*;* PMID: 30538339; their Figure 3).

At the time of tissue collection, tdTomato^{+} cells therefore represent a population highly enriched for long-lived, resident cardiac macrophages. To further refine this population,

cells were isolated using flow cytometry based on $CD45^{+}$ $CD11b^{+}$ $CD64^{+}$ gating, which enriches for macrophages and excludes most non-myeloid cells.

We agree with the reviewer that without this temporal separation, CX3CR1-based labeling alone would not distinguish monocyte-derived from resident macrophages. We have clarified this point in the revised manuscript.

With respect to “orthogonal validation,” the purpose of this experiment was to independently assess transcriptional changes in a purified population of resident cardiac macrophages using bulk RNA-seq, separate from the single-nucleus RNA-seq dataset. This approach allowed us to confirm that key transcriptional signatures (e.g., MHCII-related gene expression changes) observed in the snRNA-seq data are also detectable in an independently isolated macrophage population, thereby providing complementary validation across experimental modalities.

We have clarified the lineage tracing protocol in the revised manuscript. Briefly, *CX3CR1*-CreER-Rosa26-tdTomato mice were administered tamoxifen chow starting at 3 weeks of age for one week. At 8 weeks of age, mice underwent pulmonary artery banding or sham surgery and were sacrificed 2 weeks later. The resultant 4-week tamoxifen washout period ensured that short-lived blood monocyte populations turned over and lost tdTomato labeling (approaching 0% in Dick et. al., *Nature Immunol.* 2019*;* PMID: 30538339). Macrophage populations were further enriched by CD11b, CD45, and CD64 gating. The isolated cells therefore represent a long-lived, resident cardiac macrophage population.

The authors make very interesting observations pertaining to gluco and mineralcorticoid receptors in humans with RVF. However, this signature is not seen in mice. If the authors performed PAB in NR3C1-deficient mice, would the heart be protected or worsened?

We thank the reviewer for this stimulating question. Whether NR3C1 deficiency would be protective or detrimental in the setting of PAB is an interesting question that our transcriptomic data alone cannot resolve. The absence of altered NR3C1 target gene signatures in the murine PAB model is one of several notable discordances between mouse and human RVF that we have now highlighted more explicitly in the Discussion. We agree that genetic or pharmacological perturbation of glucocorticoid signaling in the PAB model would be a valuable experiment to determine the functional role of this pathway in RV remodeling, and we discuss this as a direction for future mechanistic studies. Given the scope of the current revision and the extensive additional experiments already performed, generation of additional genetic mouse models was not feasible for this revision, as discussed with the editors.

2. The authors extensively rely on CCR2 for their subpopulations of macrophages. Generally, the conserved TLF (TIMD4, LYVE1 and FOLR2) signature can be used for resident embryonic macrophages across species and tissues (Dick...Epelman 2022 Sci Immunology;). These are specifically known to be conserved in humans and across most tissues. The authors mention these genes in the discussion and that they are perhaps

expressed in a CCR2+ population which is highly unexpected. Can the authors use their dataset and compare it to those already published to ascertain the existence of this population and more completely describe its dynamics, location and transcriptional landscape during health and disease, aging, and in mice (PAB) versus humans (RVF). If RVF is uniquely changing the resident embryonic population to a more inflammatory subset in the absence of monocyte input that would have very interesting ramifications that would shape our understanding of ontogeny and macrophage heterogeneity and plasticity of responses. The analyses can also incorporate the authors' observations regarding MHC-II and what role they may be playing in heart failure, which is not completely delineated.

We thank the reviewer for this important and detailed comment regarding macrophage heterogeneity and ontogeny.

In the revised manuscript, we have substantively expanded the characterization of the cardiac $CCR2^{-}$ resident macrophage compartment using the conserved TLF framework (TIMD4, LYVE1, FOLR2) recommended by the reviewer. We now annotate the resident pool with the explicit TLF marker set and discuss heterogeneity within the resident population in the framework of Dick et al. (*Sci Immunol* 2022; PMID 34995099). However, we have not claimed definitive sub-population resolution: initial high-resolution subclustering of $CCR2^{-}$ residents identified three transcriptionally distinguishable subsets (enriched for HPGDS, a broader F13A1/CD209/VSIG4 signature, or RBPJ), but these subsets showed no disease-specific expansion (all differences <10% of total resident macrophages) and were combined into a single $CCR2^{-}$ resident population for downstream analyses. Formal validation of the TLF^{+} /intermediate/$MHC\text{-}II^{hi}$ framework awaits larger cohorts and complementary protein-level readouts.

In the Xenium spatial dataset, where greater cell numbers enabled finer subclustering, we identified additional myeloid subtypes including Macrophage C1q and Mast Cells. Pseudobulk DESeq2 analysis across disease states revealed resident macrophages as the most transcriptionally responsive myeloid population, with progressive loss of tissue-resident identity markers (VSIG4, CD163) during the NF-to-pRV transition and cumulative inflammatory de-repression across NF→RVF (CD83 induction, MHCII induction including HLA-DRB1, and NR4A immediate-early family activation in residents).

These data are consistent with phenotypic remodeling of resident macrophages toward a more inflammatory and antigen-presenting state, but we now also explicitly note the spatial co-occurrence of iMacs and monocytes at the tissue periphery (**Figure 6I**), which leaves open a contribution from monocyte-derived recruitment versus the possibility of a monocyte-induced transition of $CCR2^{-}$ resident macrophages to a pro-inflammatory state. We have revised the manuscript to describe these changes as shifts in macrophage activation state rather than definitive lineage transitions, and we acknowledge that formal lineage tracing would be required to resolve whether the inflammatory macrophage population arises from resident macrophage reprogramming, monocyte infiltration, or both.

We have expanded the Discussion to integrate these findings with the MHCII induction observed across platforms and to contextualize them within the broader literature on macrophage ontogeny and plasticity in cardiac disease.

2079

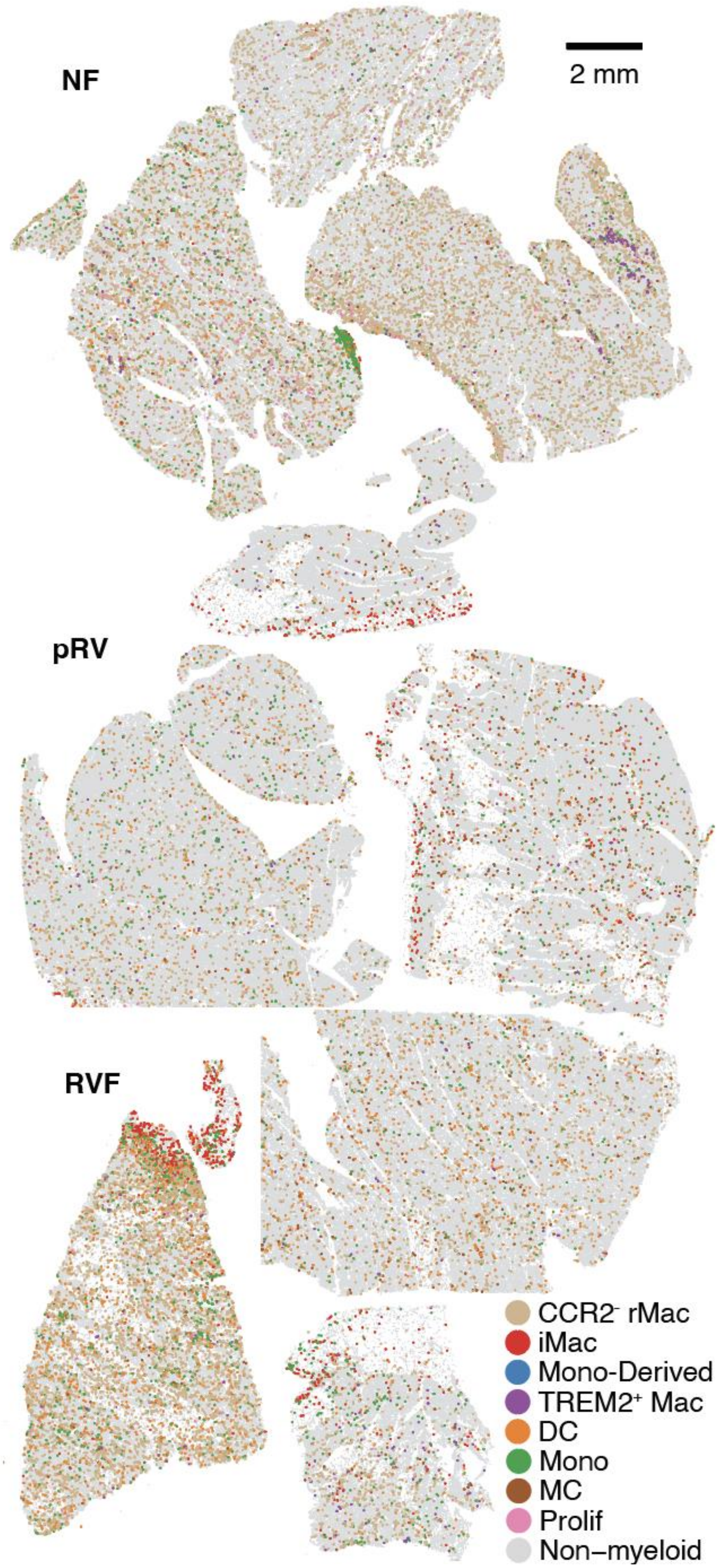
NF
2 mm
pRV
RVF
CCR2⁻ rMac
iMac
Mono-Derived
TREM2⁺ Mac
DC
Mono
MC
Prolif
Non–myeloid

Figure 6I: Spatial myeloid localization. Spatial localization of myeloid subtypes in the full Xenium dataset (625,305 cells). Non-myeloid cells are shown in light grey; $CCR2^{-}$ rMacs and Macrophage C1q are combined.

Reviewer #5 (Remarks to the Author):

This study by Kuznetsov et al provides an important framework for understanding human right ventricular failure (RVF), integrating bulk, single-nucleus, and spatial transcriptomic approaches. In addition to its insights into human RVF, the study includes important comparative analyses of RV from pediatric single ventricle patients and from the mouse pulmonary artery banding (PAB) model of pressure overload-induced RVF. One of the most intriguing findings is the opposing regulation of mitochondrial gene programs where expression of nuclear encoded mitochondrial genes were found to be suppressed in adult RVF, but induced in both the PAB and pediatric samples. This raises questions about how age-, model-, or even species-specific differences might impact the expression of mitochondrial genes and mitochondrial function. One potential interpretation of the data is the unexpected possibility that mitochondrial function may actually be enhanced in the setting of pediatric single ventricle physiology or early-stage PAB. Given that the authors have established a assay to assess mitochondrial functon(respiration) using banked tissue, additional discussion of how transcriptional changes relate to mitochondrial function, and extending these functional assessments to at least the PAB model, would strengthen the conclusions and improve the overall completeness of an already exciting study.

Concerns:

1. The respirometry data provide important functional insights into mitochondrial function in RVF. The authors state that complex I+II-supported respiration is impaired in RVF. However, this may be a misinterpretation. Oxygen consumption with complex I substrates alone is not significantly reduced compared to NF controls, whereas respiration supported by complex II substrates, or combined complex I+II substrates, is significantly reduced. This pattern suggests that the primary functional deficits may lie downstream of complex II (possibly complex II, III or IV). Given that complex IV activity is impaired (based on TMPD/ascorbate-driven respiration), it is unclear from respirometry alone if RVF causes primary defects to complex II and III. More importantly, do the transcriptomic data, and particularly changes in expression of nuclear-encoded mitochondrial genes involved in respiratory chain/electron transfer support the functional data measured by respirometry (for example, are there changes to complex IV subunits or assembly factors?)? Additionally, does RVF lead to reduced respiratory chain complex assembly with particular complexes preferentially affected? Correlating transcriptional changes with functional data would help strengthen the mechanisms.

We thank the reviewer for this detailed and insightful comment regarding the interpretation of the respirometry data. We agree that the pattern of respiratory chain impairment warrants more careful discussion.

Respirometry interpretation: In the revised manuscript (**Figure 5H**) we have clarified that Complex I–supported respiration alone is not significantly reduced, whereas Complex II–supported and combined Complex I+II respiration are significantly reduced (Kruskal–Wallis), and Complex IV activity (TMPD/ascorbate-driven) trends downward without

reaching significance. As the reviewer notes, this places the dominant functional deficit at or downstream of Complex II.

Transcriptome–function correlation: To test whether the transcriptional data localize the functional deficit, we annotated nuclear-encoded subunits and assembly factors of each respiratory-chain complex (CI, NDUF; CII, SDH; CIII, UQCR/CYC1; CIV, COX subunits and assembly factors; CV, ATP5) and examined their expression across disease states in cardiomyocyte pseudobulk (snRNA-seq, n=11) and bulk RV tissue (n=142). In the cardiomyocyte compartment, the dominant source of the tissue respirometry signal, nuclear-encoded ETC transcripts showed a modest, coordinate downregulation (median log2FC ≈ −0.22) without preferential targeting of any single complex; COX (Complex IV) subunit and assembly-factor transcripts were not selectively reduced. Bulk tissue showed a smaller overall decline (median log2FC ≈ −0.08) with a relatively COX-weighted pattern, but bulk aggregates all cardiac cell types and is confounded by the cell-composition shifts that accompany RVF (notably endothelial expansion and relative cardiomyocyte loss). We therefore do not read the bulk per-complex pattern as cardiomyocyte-intrinsic.

Critically, neither compartment reproduces the complex-specific functional pattern at the transcript level: respirometry localizes the deficit to Complex II/Complex I+II, whereas the cardiomyocyte transcriptional decline is broad and complex-agnostic. The functional specificity is thus not accounted for by subunit or assembly-factor transcription, and the magnitude of the functional deficit exceeds the modest transcriptional change. Together these implicate post-transcriptional and assembly-level mechanisms, impaired complex assembly, heme/cofactor availability, or substrate limitation, rather than reduced subunit transcription, as the proximate driver of the respiratory impairment.

2. As noted above, the finding that mitochondrial gene expression is upregulated in PAB and pediatric HLHS RV samples was a surprising and really intriguing finding from the study. Given that depression of mitochondrial genes was associated with reduced respiration in adult RVF samples, is mitochondrial function enhanced in response to PAB and in the RV of pediatric single ventricle disease? Are there transcription factors regulating mitochondrial gene expression that are engaged in the PAB/pediatric single ventricle hearts that are suppressed in adult RVF?

We thank the reviewer for this question, and we want to be transparent about an important correction it prompted. In our initial analysis, the apparent upregulation of mitochondrial transcripts in the PAB and pediatric RVs was an artifact of aggregating signal across pooled nuclei rather than computing per-sample (pseudobulk) summaries. When we re-ran the analysis with per-animal and per-patient pseudobulk aggregation and gene-set enrichment (the same framework applied to the adult cohort) the apparent induction did not hold: the direction reversed in PAB and attenuated to non-significance in pediatric. We have corrected this throughout the revised manuscript (**Figures 5H–J**; **S6F–G**; **8F**), and the corrected result changes our answer.

Regarding mitochondrial function: On the corrected analysis, transcriptional and respiratory changes are concordant across all three settings rather than dissociated:

- *Adult RVF*: coordinated suppression of nuclear-encoded mitochondrial transcripts with significantly reduced Complex I+II coupled respiration (**Figure 5H**).
- *Murine PAB*: cardiomyocyte mitochondrial transcripts are coordinately suppressed (MitoCarta GSEA NES = −1.8, FDR < 10^{-17}; Figure S6F), concordant in direction with adult RVF though milder at the per-animal level, accompanied by reduced maximal coupled (CI+II+FAO) respiration at both the 2-week and 2-month timepoints (**Figure 5I**).
- *Pediatric HLHS-palliated failing RV*: mitochondrial transcripts show only a modest, non-significant downregulation, directionally consistent with adult but far milder (**Figure 8F**), and respiration is preserved at every complex (Complex I −9.7%, I+II −2.3%, II +4.1%, IV +42.4%; all Mann–Whitney NS; **Figure 5H**).

To the reviewer's specific question, mitochondrial function is not enhanced in either setting. In PAB it is reduced, tracking the transcriptional decline; in the pediatric single-ventricle RV it is preserved but not enhanced. The non-significant complex-level point estimates (including the +42% Complex IV) are underpowered in this small, palliation-heterogeneous cohort, and preserved maximal ETC capacity does not exclude the substrate-level impairment (i.e. reduced CPT activity, altered TCA-cycle flux reported in explanted single-ventricle hearts by Garcia et al., *JACC Basic Transl. Sci. 2023*; PMID: 37034285). We therefore describe the pediatric RV as metabolically spared from the coordinated ETC transcriptional-and-functional collapse of adult RVF, rather than metabolically enhanced.

The corrected picture strengthens rather than weakens the association the reviewer notes between mitochondrial transcript levels and respiratory capacity: that coupling holds in both adult and murine pressure overload, and the pediatric RV is the informative exception in which neither readout is appreciably perturbed. We note that in both adult and PAB the magnitude of the functional deficit exceeds the modest transcriptional decline (median log2FC ≈ −0.22), consistent with an additional post-transcriptional contribution (impaired complex assembly, altered mitochondrial dynamics, or substrate limitation) layered on top of transcriptional suppression.

Regarding transcription factors: This correction also reframes the reviewer's transcription-factor question. Because PAB mitochondrial transcripts are suppressed (concordant with adult) rather than induced, the data do not support a compensatory biogenesis-TF program uniquely engaged in PAB and lost in adult RVF. The more parsimonious interpretation is a shared, milder suppression. In the adult cohort, the suppressed regulators are the estrogen-related receptor/PGC-1α axis: at the single-nucleus CM pseudobulk level, ESRRA and PPARGC1A were significantly downregulated versus NF, with ESRRG and PPARA trending down concordantly (**Figure 5G**), and ESRRA was concordantly suppressed across NF→pRV→RVF in snRNA-seq (**Figure 5F**). The remaining regulators (NRF1, PPARD, NFE2L2) were directionally mixed and subthreshold.

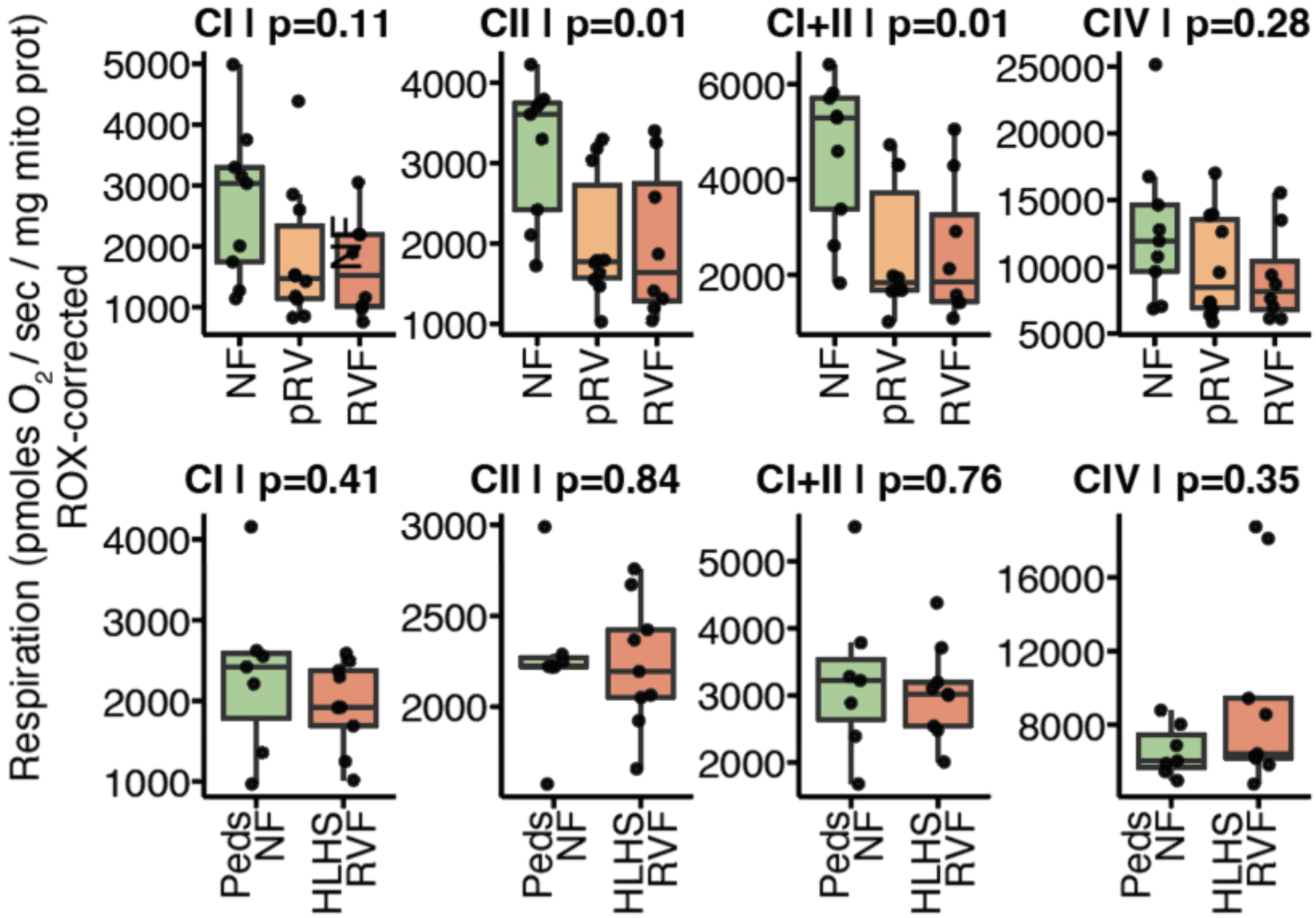

Figure 5H: Adult and pediatric respirometry. Oroboros 2k high-resolution respirometry on freeze/thaw-isolated RV mitochondria, ROX-corrected and normalized to starting mitochondrial protein mass (pmoles O_2 /sec/mg mitochondrial protein). Each dot is one patient. Adult cohort **(top)**: NF n=9, pRV n=10, RVF n=8; Kruskal–Wallis p-values shown. Pediatric cohort **(bottom)**: pediatric NF (n=7) vs HLHS-palliated failing (n=9); Mann–Whitney pairwise p-values.

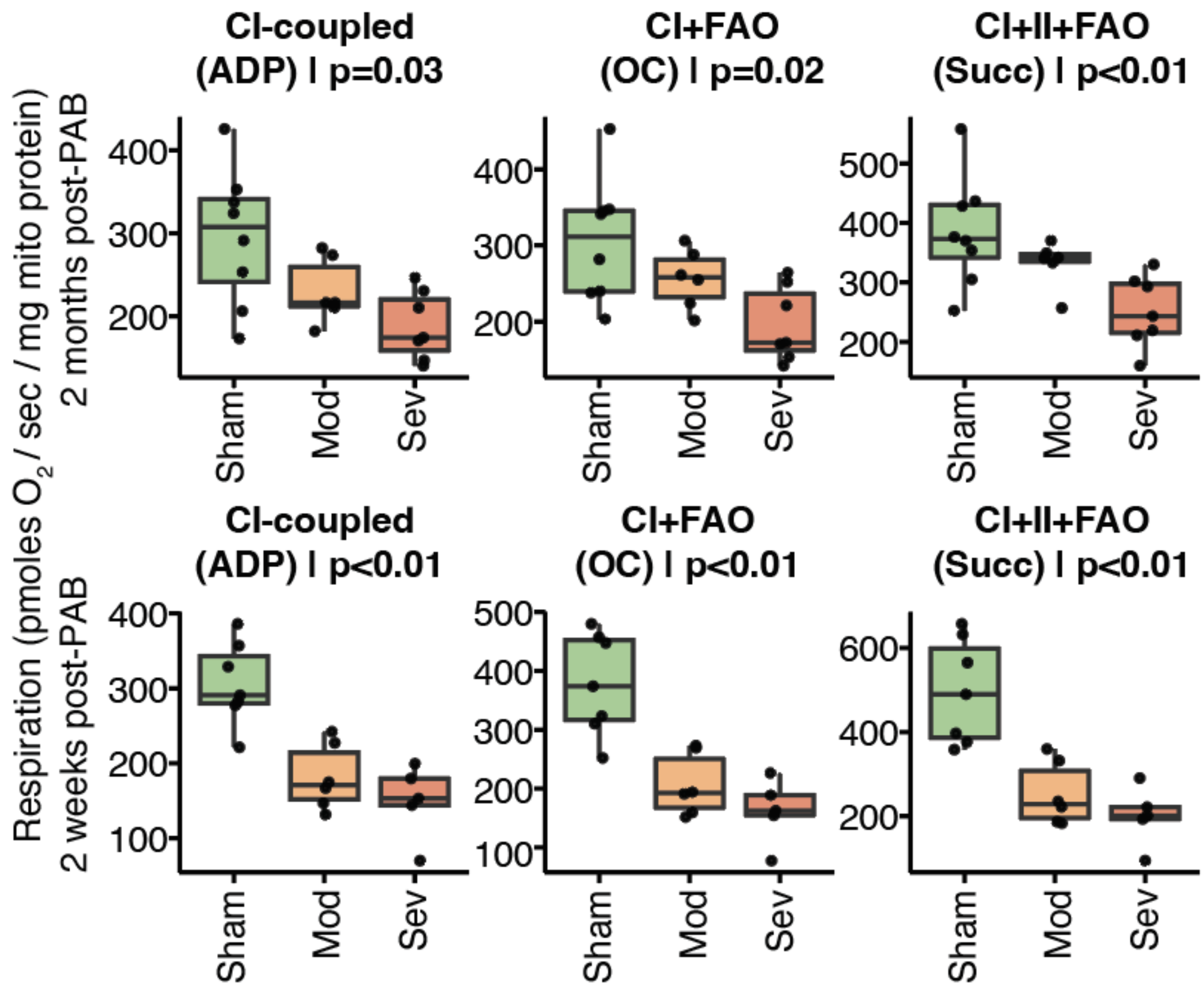

Figure 5I: PAB respirometry. Respirometry in PAB model at 2-month **(top)** and 2-week **(bottom)** time points vs sham, using a substrate-addition protocol (PM → ADP → cyt c → octanoyl-carnitine → succinate) on tissue homogenate. CI+II+FAO maximal coupled respiration (Succ-driven) is the headline measure. 2-week: Sham n=8, Mild n=6, Severe n=7. 2-month: Sham n=7, Mild n=6, Severe n=5. Each dot is one animal; Kruskal–Wallis p-values shown.

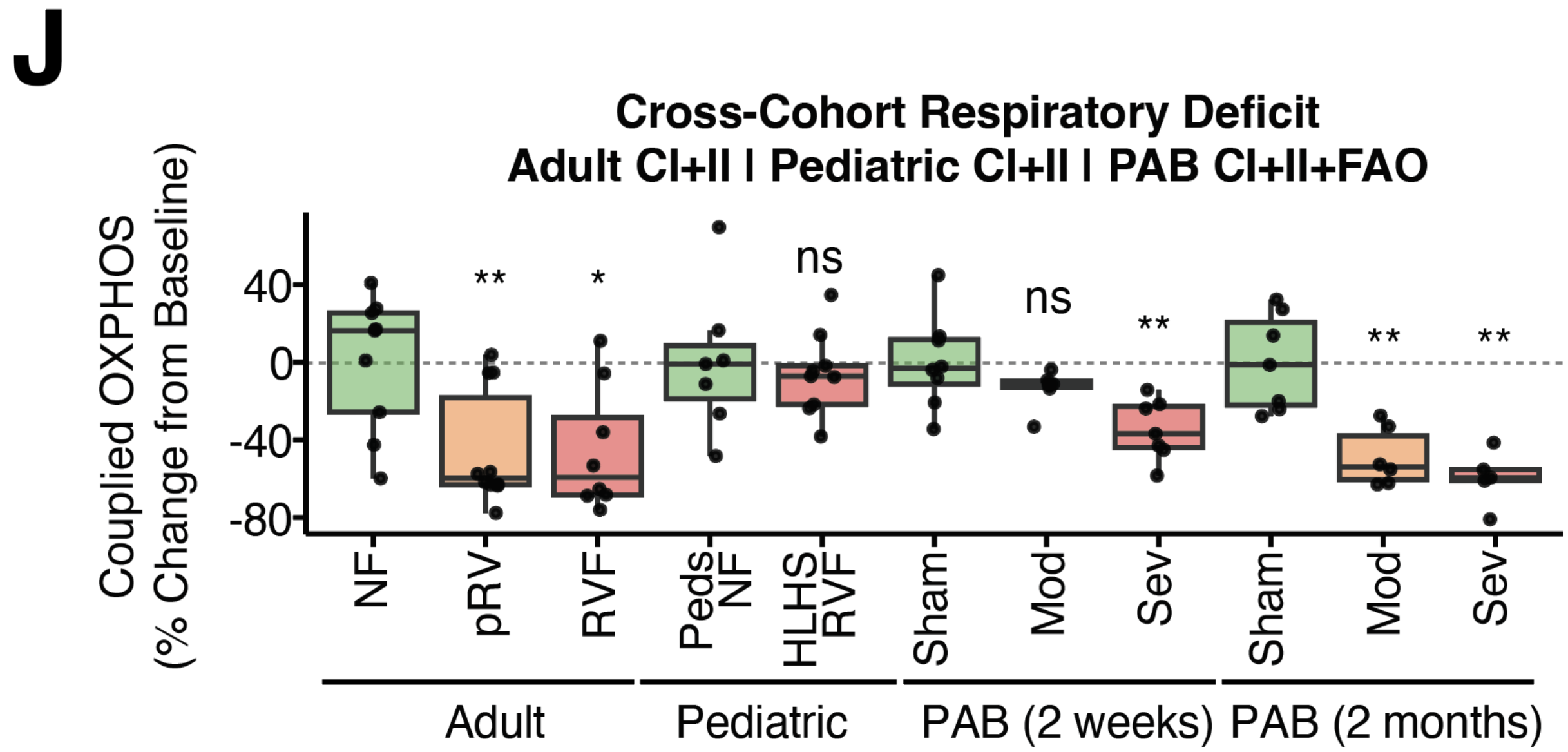

Figure 5J: Cross-cohort respirometry comparison. Cross-cohort summary of RV mitochondrial OXPHOS, normalized to within-cohort controls. Maximal coupled respiration is reduced in adult RVF and murine PAB but preserved in pediatric HLHS-palliated failing RV.

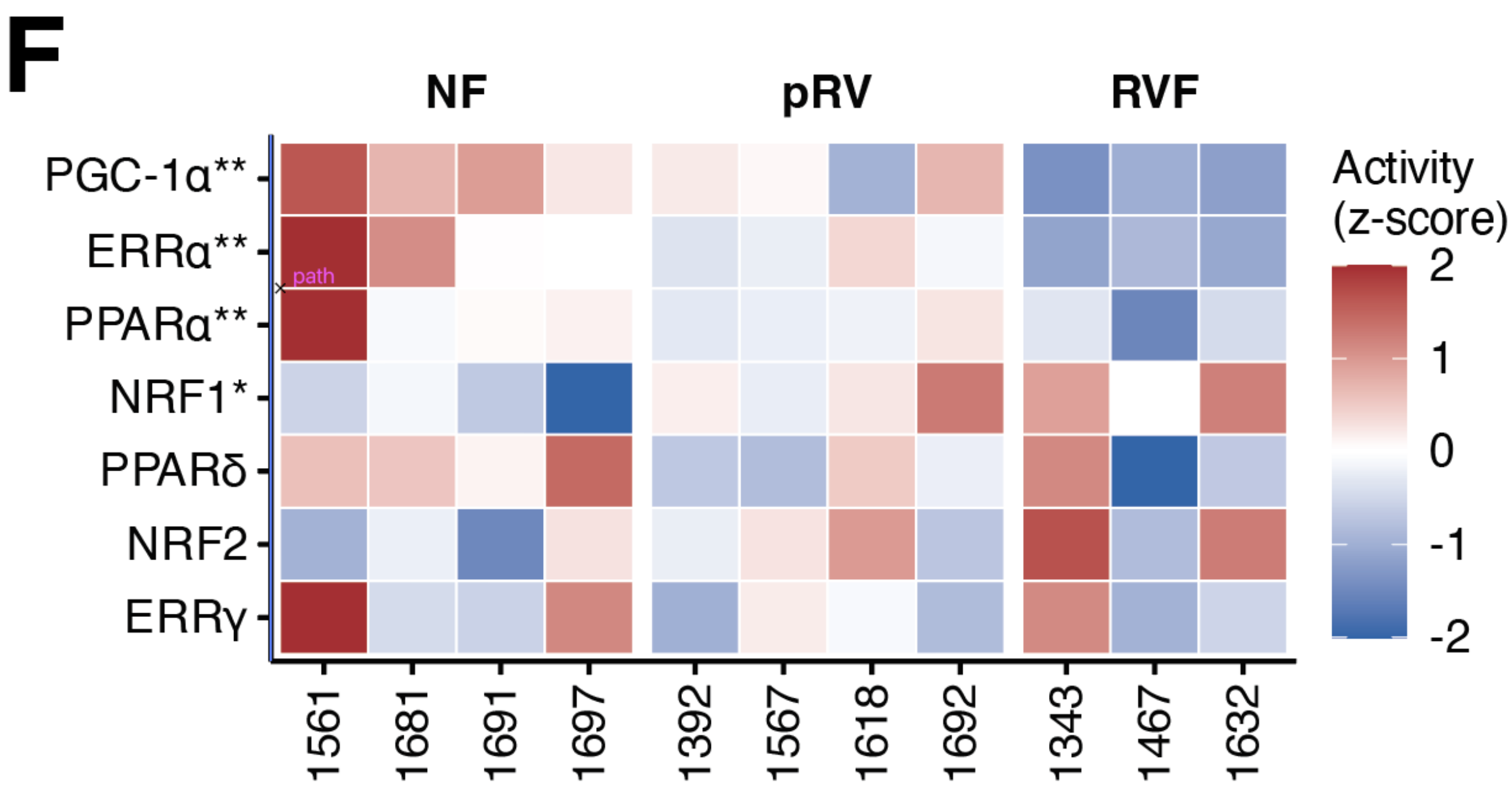

Figure 5F: Mitochondrial TF activity. CollecTRI/decoupleR transcription factor activity for mitochondrial regulators inferred from CM snRNA-seq.

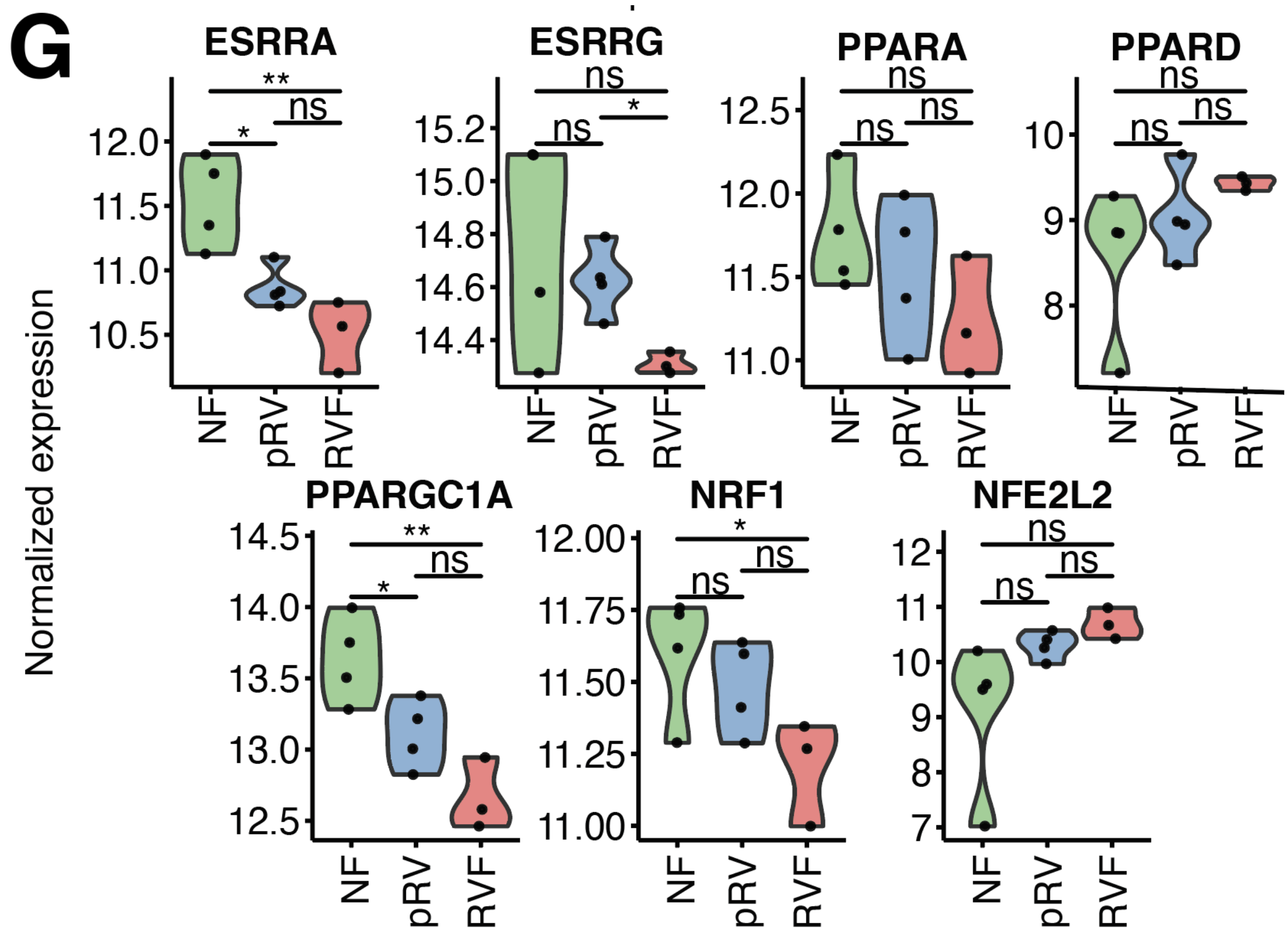

Figure 5G: TF expression violins. snRNA-seq pseudo-bulk violin plots of ESRRA, ESRRG, PPARA, PPARGC1A expression in cardiomyocytes.

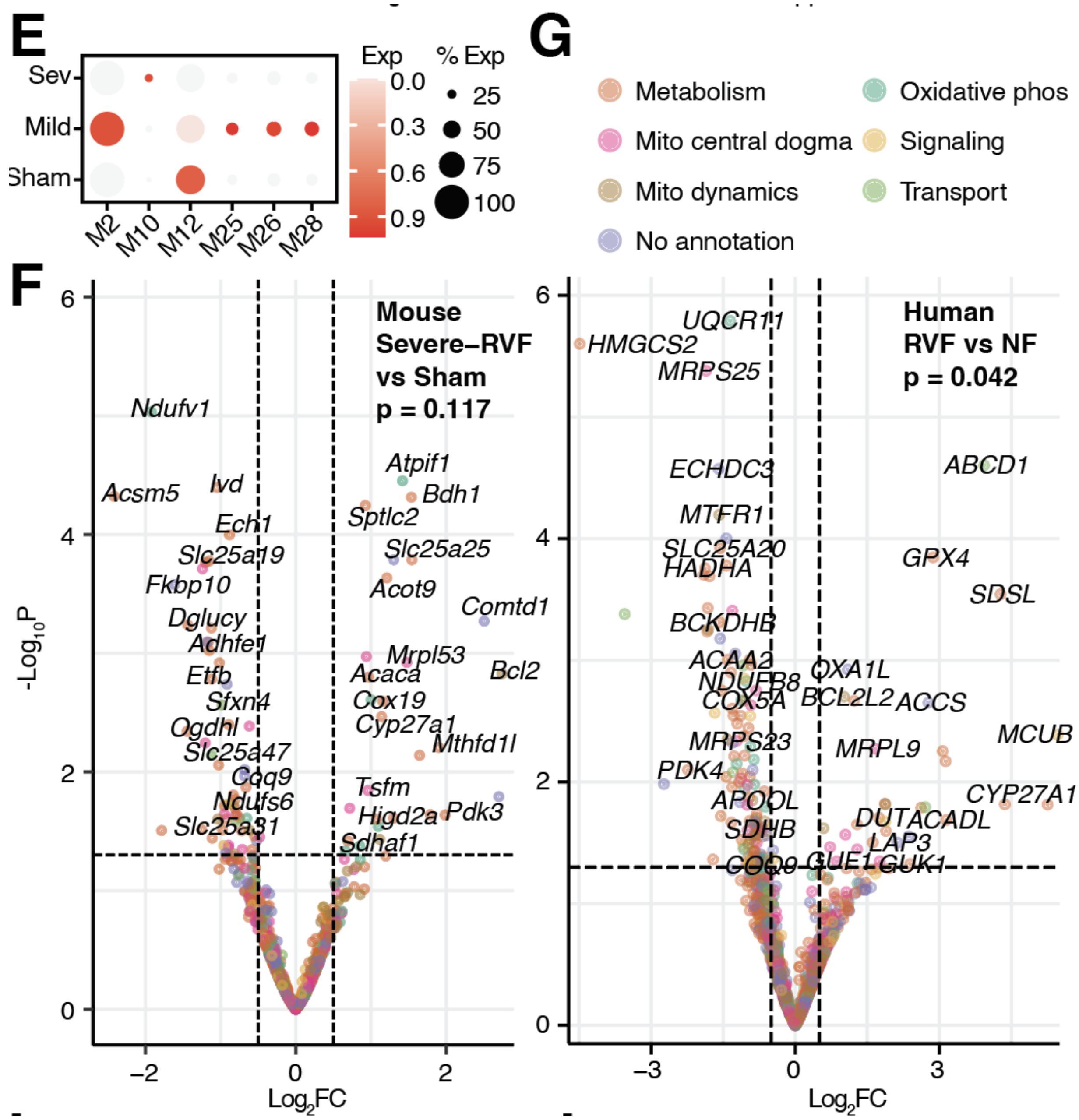

Figure S6E-G. Murine and human mitochondrial transcripts in RVF. (E) Dot plot of normalized *Seurat*-calculated expression score of corresponding WGCNA module genes in the mouse PAB cardiomyocytes by disease state. Previously observed trends in the human dataset are poorly conserved. **(F)** Per-mouse pseudobulk DESeq2 volcano of MitoCarta3.0 genes in murine RV CMs, severe-RVF vs NF (n = 4 vs 3) with apeglm $\log_2$ fold-change shrinkage; points colored by MitoCarta3.0 top-level pathway. Per-mouse Wilcoxon p-value is shown for the NF vs (mild-RVF and severe-RVF) comparison. **(G)** Per-patient pseudobulk DESeq2 volcano of MitoCarta3.0 genes in human RV CMs, RVF vs NF (n = 3 vs 4) with apeglm $\log_2$ fold-change shrinkage; points coloured by MitoCarta3.0 top-level pathway. Per-subject Wilcoxon p-value is shown for the NF vs (pRV and RVF) comparison.

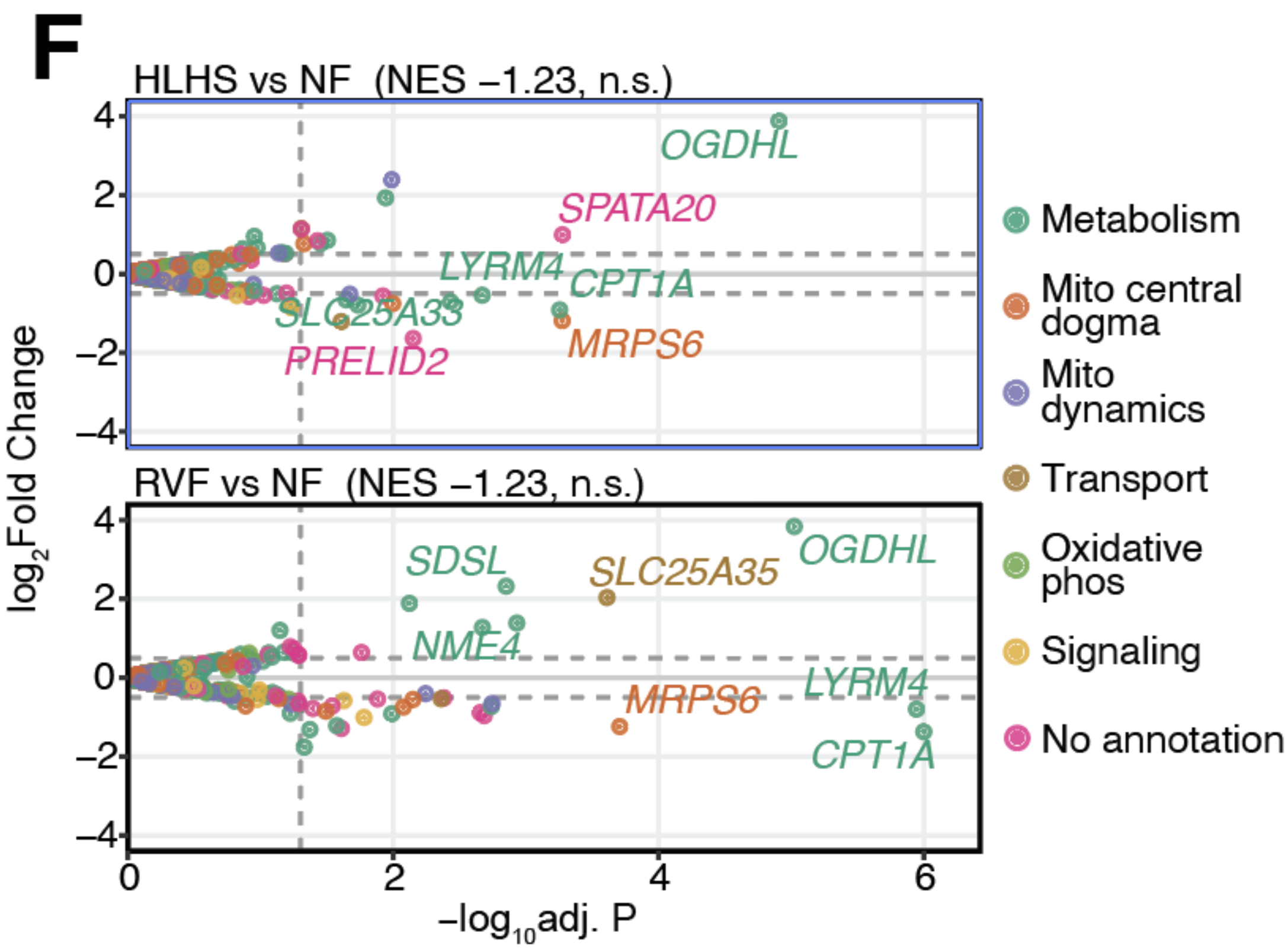

Figure 8F. Pediatric mitochondrial transcripts. MitoCarta3.0 gene-level volcano plots for pediatric cardiomyocytes: ped-HLHS (**top**) and ped-RVF (**bottom**) versus ped-NF. Per-patient pseudobulk DESeq2 (apeglm log_2FC shrinkage, FDR from the unshrunken Wald test; n = 4 ped-NF, 5 ped-HLHS, 5 ped-RVF). Each point is a MitoCarta3.0 gene colored by top-level MitoPathway category. Gene-set enrichment over the full ranking: ped-HLHS normalized enrichment score (NES) = −1.23, ped-RVF NES = −1.23. The shift is not significant at the patient level.

Changes in manuscript:

- Added analysis of mitochondrial transcription factor activity across disease states (**Figure 5F**: CollecTRI/decoupleR-ULM TF activity for mitochondrial regulators in CM snRNA-seq).
- Added respirometry data for PAB and pediatric RV samples (Figure 5H–J). On corrected per-sample pseudobulk analysis, murine PAB shows coordinate suppression of mitochondrial transcripts (MitoCarta GSEA NES = −1.8; Figure S6F) with reduced respiration — a coupled decline concordant with adult RVF — whereas pediatric HLHS-palliated failing RVs show largely unchanged mitochondrial transcripts (modest, non-significant downregulation) with preserved respiration, indicating metabolic sparing rather than compensation.
- Expanded Discussion to address the relationship between mitochondrial gene expression and respiratory function, with concordant (coupled decline) in adult and murine RVF and spared in pediatric, and the role of the ESRRA/PPARGC1A (ERRα/PGC-1α) biogenesis program.

3. I understand the rationale for looking at the 2 weeks post-PAB time point, but could this early timepoint reflect a compensatory period where mitochondrial function might paradoxically be maintained? Analysis of an expanded time course (perhaps more fulminant failure) correlating gene expression with mitochondrial function might be warranted as a more complete comparison with adult human RVF.

We thank the reviewer for raising this important concern regarding the temporal dynamics of the PAB model.

To address whether the 2-week time point reflects a compensatory phase, we extended the PAB model to a 2-month time point and performed echocardiographic assessment and mitochondrial respirometry at both stages. At 2 weeks post-PAB, echocardiography confirmed RV pressure overload with evidence of early functional impairment (reduced TAPSE and fractional area change). At 2 months, PAB animals demonstrated progressive RV dysfunction with further reductions in systolic function, consistent with established RVF rather than compensated pressure overload.

Critically, mitochondrial respirometry revealed that respiratory capacity was reduced at both the 2-week and 2-month time points compared to sham controls (**Figure 5J**). The persistence of mitochondrial dysfunction across both early and late stages of the model indicates that mitochondrial impairment is not limited to an early compensatory period but is a sustained feature of RV pressure overload.

These findings, combined with the respirometry data from adult human RVF and pediatric RV samples, demonstrate that mitochondrial dysfunction is a conserved feature between adult humans and mice, although is notably not replicated in the pediatric HLHS cohort. The extended time course strengthens the comparison with adult human RVF and supports the conclusion that mitochondrial functional impairment accompanies RV remodeling from early pressure overload through established failure.

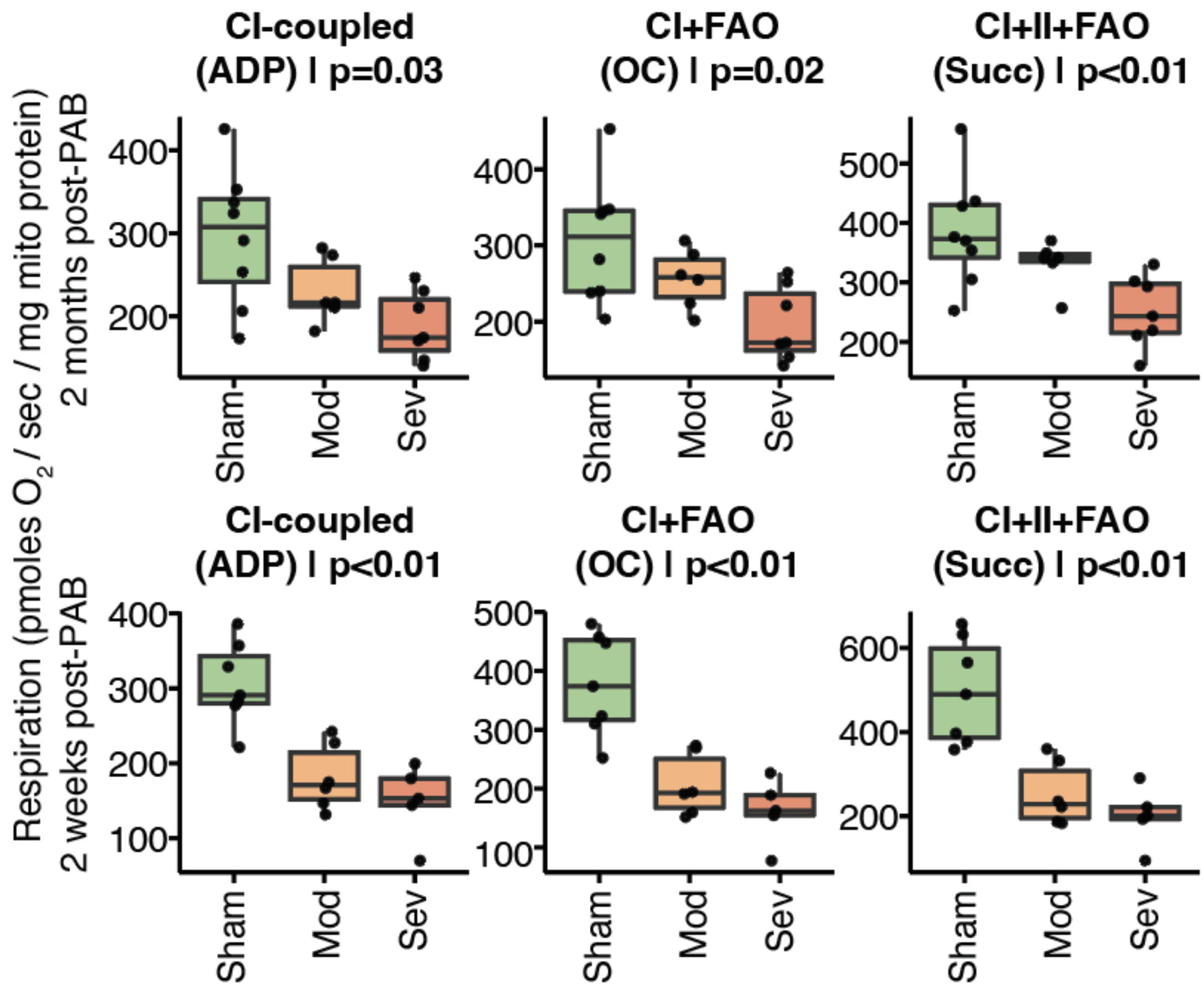

Figure 5I: PAB respirometry. Respirometry in PAB model at 2-month **(top)** and 2-week **(bottom)** time points vs sham, using a substrate-addition protocol (PM → ADP → cyt c → octanoyl-carnitine → succinate) on tissue homogenate. CI+II+FAO maximal coupled respiration (Succ-driven) is the headline measure. 2-week: Sham n=8, Mild n=6, Severe n=7. 2-month: Sham n=7, Mild n=6, Severe n=5. Each dot is one animal; Kruskal–Wallis p-values shown.

Changes in manuscript:

- Added 2-month PAB echocardiographic and respirometry data demonstrating progressive RV dysfunction and persistent mitochondrial impairment.
- Added corresponding figure panels comparing 2-week and 2-month PAB outcomes.
- Revised Results to discuss both early and late PAB time points in the context of disease progression.

Point-by-Point Response to Reviewers' Comments

Manuscript NCVR-2025-04-0552B

Human Adult and Pediatric Multimodal Transcriptomic Atlas of Progression from Pressure-Loaded to Failing Right Ventricle

We thank the editor and the four referees for their careful reading of the revised manuscript and for their positive assessment. We are grateful for the constructive comments across both rounds of review, which have substantially strengthened the work. Below we respond point by point to the remaining requests. Reviewer comments are reproduced in italics; our responses follow in plain text. All line numbers refer to the previously submitted version unless otherwise noted.

Reviewer #1 (Remarks to the Author):

I appreciate the opportunity to evaluate the revised version of the article entitled "Human Adult and Pediatric Single-Nucleus Transcriptomic Atlas of Progression from Pressure Loaded to Failing Right Ventricle." The authors did a very nice job and good faith effort to address my comments. In the end, I think the revised version is much better. I think this is a much needed addition to the literature and provides additional insight into RV biology. After this much work and effort, I think it is unfair to ask for additional work and think this is great. Congratulations.

We thank the reviewer for this generous assessment and for the constructive comments in the first round of review, which materially improved the manuscript — in particular the addition of direct mitochondrial functional assessment across all three cohorts. No further changes were requested.

Reviewer #2 (Remarks to the Author):

The authors have carefully and thoroughly addressed all concerns raised and have substantially revised their manuscript. These changes have significantly improved both the data presentation and the discussion of relevant conclusions. I have no further comments, except for one incorrect citation: The statements in the discussion (lines 684–689) refer to reference 57 (Jurida et al., doi: 10.1038/s44161-024-00485-1) rather than to the paper by Boucherat et al. (ref. 58, doi: 10.1038/s44161-022-00113-w), which is discussed later, in lines 699–701.

We thank the reviewer for catching this error. On re-examining the passage we found that the erroneous citation occurred at two adjacent sentences within the flagged block rather than at one. Both now correctly cite reference 57 (Jurida et al., 2024). We have verified that reference 58 (Boucherat et al., 2022) remains correctly cited at the later plasma LTBP-2 biomarker sentence, as the reviewer notes.

Changes in manuscript:

- Corrected two in-text citations in the Discussion (previously ref. 58, now ref. 57, Jurida et al. 2024) at the rat PAB/AB cross-species comparison.
- Verified that ref. 58 (Boucherat et al. 2022) remains correctly cited at the plasma LTBP-2 biomarker sentence in the following paragraph.
- Re-verified all 108 in-text citation call-outs against the numbered bibliography. No further mismatches.

Reviewer #3 (Remarks to the Author):

I would like to thank the authors for the extensive amount of work they put into revising their manuscript. In general, they addressed all my specific concerns, and I believe that the new analyses and a more focused discussion of some of the biological observations substantially improve the work. I was also glad to see a more meaningful utilization of the Xenium dataset, and a more detailed description of the bioinformatic analysis. Also regarding the code, the authors did a good job in providing a more transparent and reproducible framework.

Below, I have some additional minor suggestions, more related to the format than the content, that do not affect my overall positive evaluation of the revised manuscript.

We thank the reviewer for the constructive engagement across both rounds of review and for these final suggestions, all of which we have adopted.

1. Since the manuscript integrates multiple datasets, the title could perhaps be revised from single-nucleus atlas to better reflect its broader scope.

We agree. The original title emphasized a single modality while the study integrates bulk RNA-seq, single-nucleus RNA-seq, and Xenium spatial transcriptomics, together with mitochondrial respirometry and histology, across adult human, pediatric human, and murine cohorts. We have revised the title to:

"Human Adult and Pediatric Multimodal Transcriptomic Atlas of Progression from Pressure-Loaded to Failing Right Ventricle"

We elected to use "multimodal" rather than "multi-omic" because the three transcriptomic modalities we integrate (bulk, single-nucleus, and imaging-based spatial) sample the same molecular layer, and we did not wish to imply chromatin or proteomic profiling that we did not perform. We have also hyphenated "pressure-loaded" as a compound adjective.

Changes in manuscript:

- Revised the manuscript title as above; updated the title in the cover letter, the response letter, and the public code repository README.

2. Seemingly, there is a contradiction between the gradually increasing extent of histology-level fibrosis (Fig. S3M), the gain of ECM signalling (Fig. 4E), and the decreasing proportion of the fibrotic myocardial niche (Fig. 3G) along the NF->pRV->RVF axis. While this might be explained by shifts of histologically fibrotic or fibrosis-adjacent areas between spatially closely related niches, it would still make sense to add a comment to the related sentence in lines 276-279 to clarify this.

We thank the reviewer for raising this, and we agree the apparent tension deserves explicit comment. The reviewer's own proposed explanation is, we believe, the correct one, and we have added it to the text. Three considerations reconcile the observations:

First, niche abundance is a relative compositional measure. Each segmented cell is assigned to exactly one of 14 niches on the basis of its k=100 nearest-neighbour cell-type composition, so the niche fractions are constrained to sum to one. Expansion of other niches in RVF (the stressed myocardium niche reached nominal significance, and the adipose-vascular, capillary-rich myocardium, arterial, and inflamed adipose stroma niches all trended upward) necessarily compresses the fractions assigned to the remaining niches, including fibrotic myocardium, independently of any change in absolute fibrotic burden.

Second, and most importantly, niches are defined by cellular neighbourhood composition, not by extracellular matrix content. As fibrosis progresses, regions that were previously classified as fibrotic myocardium acquire stressed cardiomyocyte, myeloid, and vascular neighbours and are reassigned to spatially adjacent niches, precisely the shift the reviewer proposes. A histologically more fibrotic ventricle can therefore contain a smaller fraction of cells meeting the narrow neighbourhood definition of the fibrotic niche. This is compounded by the fact that matrix-dominated scar is relatively acellular and thus contributes few segmented cells to any cell-based compositional measure. Increased collagen deposition per unit area does not translate into more cells assigned to a fibrotic niche.

Third, the ECM signalling gain in Figure 4E is a measure of inferred signalling activity per cell, not of compartment size. Intensifying matrix signalling within a constant or contracting cellular compartment is fully consistent with rising histological fibrosis.

We would also note that no niche abundance change survived multiple-comparison correction at n=3 per group, and the fibrotic myocardium decrease is a non-significant trend. We have made this explicit at the relevant sentence so the observation is not over-read.

Changes in manuscript:

- Added clarifying text to the niche-abundance paragraph (previously lines 276–279) stating that the lower fibrotic-myocardium share in RVF should not be read as less fibrosis, that niche proportions are compositional and describe how section area is partitioned rather than how much matrix each neighbourhood contains, and that the rising histologic collagen burden (Figure S3M), the pRV→RVF gain of ECM signalling (Figure 4E) and the matrifibrocyte commitment trend (Figure S3G) are compatible with fibrotic remodeling intensifying within existing neighbourhoods while the stressed-myocardium and vascular niches expand their share.
- Expanded the Figure 3G legend to state that niche frequencies are compositional. Each segmented cell is assigned to exactly one of the 14 niches, so the fractions sum to one within a sample and a fall in one niche's share can reflect expansion of others. We also restate that no niche abundance shift survived multiple-comparison correction at n=3 per group.
- The sentence noting that no niche survived multiple-comparison correction was already present in the previous version.

3. Please make sure that all abbreviations, including CTEPH and GDMT, are expanded at their first use in the manuscript, to help non-specialist readers.

We have expanded CTEPH and GDMT at first use, and we swept the main text for any other abbreviation introduced without expansion. This identified WGA, which was defined in the Methods and in a figure legend but used unexpanded at its first appearance in the Results. It is now expanded there.

CTEPH → chronic thromboembolic pulmonary hypertension (CTEPH)

GDMT → guideline-directed medical therapy (GDMT)

WGA → wheat-germ agglutinin (WGA)

We confirmed that DEG, HLHS, GRE, PCWP, IQR, ETC and TLF are already expanded at or immediately adjacent to their first use — DEG, for example, is introduced as "DE genes (DEGs)".

The sweep identified ten further abbreviations used without expansion at first appearance, which are now expanded there:

Abbreviation	Expansion
TAPSE	tricuspid annular plane systolic excursion
FAC	fractional area change
DCM	dilated cardiomyopathy
RAAS	renin–angiotensin–aldosterone system
TCA	tricar

We have retained ECM, MHC, NS and similar terms in abbreviated form as standard usage for this readership.

Changes in manuscript:

- Expanded CTEPH, GDMT, WGA, TAPSE, FAC, DCM, RAAS, TCA, FAO, CPT, RIN, GSEA and NES at first use in the main text.
- Verified that DEG, HLHS, GRE, PCWP, IQR, ETC and TLF are already defined at first appearance and require no change.

4. In Fig. 3A, the UMAP presents data from segmented cells, not nuclei - please correct the in-panel label.

The reviewer is correct. The Xenium data are Proseg-resegmented cells, and the figure legend already read "625,305 cells." The in-panel annotation was inconsistent with it. We have corrected the in-panel label to read cells.

Changes in manuscript:

- Corrected the in-panel label in Figure 3A from "nuclei" to "cells" so that the panel, the legend, and the Methods are consistent in describing the Xenium data as segmented cells.

5. In Fig. 8A, it is not possible the separate the HLHS and adult RV datasets, and the in-panel n= text only includes the HLHS data. Please add an in-lay panel coloured by sample type to show successful integration of the two sample types, and include the number of RV numbers in the in-panel n= text.

We agree and have made both changes. Figure 8A now carries an inset UMAP colored by dataset of origin (pediatric HLHS versus adult RV), mirroring the patient-colored inset already used in Figure 3A, which demonstrates that the two datasets co-embed across shared cell types rather than separating by cohort. The in-panel n= text now reports both datasets: pediatric HLHS n=148,206 nuclei and adult RV n=61,398 nuclei (total n=209,604 nuclei). In preparing this panel we identified an error in the previous Figure 8A legend, which reported n=209,604 for the HLHS dataset alone. That value is in fact the combined total of the two co-embedded datasets. The legend has been corrected, and we thank the reviewer, since the request for a dataset-of-origin panel is what surfaced it.

Changes in manuscript:

- Added an inset to Figure 8A coloured by dataset of origin (pediatric HLHS vs adult RV) to demonstrate successful integration of the two sample types.
- Updated the caption to correct numbers (HLHS n=148,206; adult RV n=61,398; total n=209,604 nuclei).
- Updated the Figure 8 legend to describe the new inset.

6. While the restructuring of the manuscript has improved its overall narrative flow, the text has also become quite dense. As a result, it is sometimes difficult to determine which dataset specific statements refer to - for example, whether the subclustering of certain cell populations was performed on the snRNA-seq or Xenium data. Please make sure this is as clear as possible throughout the manuscript.

We appreciate this point and agree that dataset provenance had become difficult to track in places. We have named the source dataset explicitly at the subclustering statements the reviewer highlights, so that snRNA-seq–derived and Xenium-derived subclusters are identified as such.

Changes in manuscript:

- Named the source dataset explicitly at the Xenium subclustering statement (58 imputed subtypes across the 12 Xenium lineages) and at the FB–myeloid spatial colocalization analysis.

I would like to thank the authors again for the opportunity to evaluate their work.

We thank the reviewer once more for the thorough and constructive review.

Reviewer #3 (Remarks on code availability):

The updated code provides good structure, a clear README file, and enough information to enable reproducing the analysis, even though due to time restrictions, we did not re-run the analysis ourselves.

We thank the reviewer for evaluating the code.

Reviewer #4 (Remarks to the Author):

This reviewer appreciates and acknowledges the extent of the appropriate revisions. I advise to accept.

We thank the reviewer for this assessment and for the comments in the first round of review, which strengthened the manuscript.
